# Parallel synthesis of 5′-amino-5′-deoxy-adenosine derivatives for focused chemical space exploration and their application as methyltransferase inhibitors

**DOI:** 10.1039/d5md00376h

**Published:** 2025-08-12

**Authors:** Sabrina N. Hoba, Marvin Schwickert, Luis Kammerer, Mark Sabin, Annabelle C. Weldert, Zarina Nidoieva, J. Laurenz Meidner, Fabian Barthels, Tanja Schirmeister, Christian Kersten

**Affiliations:** a Institute of Pharmaceutical and Biomedical Sciences, Johannes Gutenberg University Mainz Staudinger Weg 5 55128 Mainz Germany kerstec@uni-mainz.de; b Institute for Quantitative and Computational Bioscience, Johannes Gutenberg-University Mainz BioZentrum l, Hanns-Dieter-Hüsch Weg 15 55128 Mainz Germany

## Abstract

Parallel syntheses and their throughput capabilities are powerful tools for the rapid generation of molecule libraries, making them highly beneficial for accelerating hit identification in early-stage drug discovery. Utilizing chemical spaces and virtual libraries enhances time and cost efficiency, enabling the faster exploitation of chemically diverse compounds. In this study, a parallel synthesis method for rapidly generating a 5′-amino-5′-deoxy adenosine-based amide and sulfonamide library of 42 compounds is described with high yields and purity, which is economical and ecological due to the reduced requirements for extensive purification. Methyltransferases recently emerged as promising drug targets. The adenosine-derived library was screened using a fluorescence polarization (FP) assay against model enzymes human DNMT2 and METTL3/14, and SARS-CoV-2 nsp14/10, resulting in the identification of three compounds binding with nanomolar affinity to nsp14/10 and three compounds binding METTL3/14 with low micromolar affinity. To demonstrate the accessibility of a broad variety of adenosine derivatives, a focused virtual chemical space of 25 241 5′-amino-5′-deoxy adenosine amides and sulfonamides, which are accessible *via* the described synthetic procedure, was generated. This chemical space was further investigated for potential biological applications through virtual screening against nsp14/10 which led to the identification of four additional ligands with low micromolar affinities.

## Abstract 2

Parallel and virtual synthesis of 5′-amino-5′-deoxy-adenosine amides and sulfonamides allows the rapid generation of molecule libraries for hit identification and early SAR exploration of methyltransferase inhibitors.

## Graphical abstract

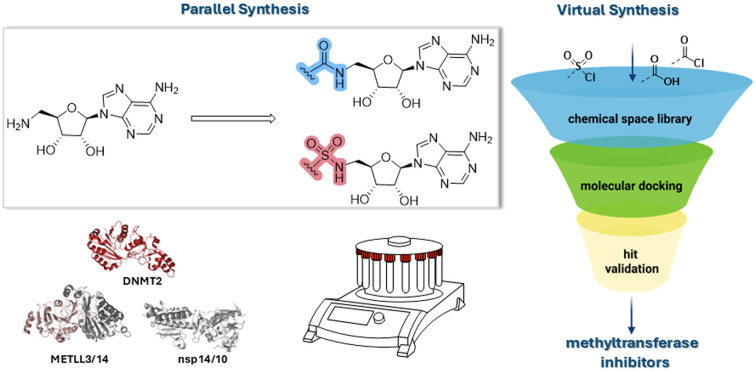

## Introduction

Over the last decades, parallel syntheses and combinatorial chemistry have evolved significantly, enabling the generation of large libraries of chemically diverse molecules for hit identification, structure–activity relationship (SAR) studies, lead discovery, and optimization.^1–3^ Parallel synthesis, either *via* solid support or in solution, is often advantageous over traditional synthesis, allowing the highly efficient simultaneous synthesis of multiple compounds with significantly reduced time and cost.^4–7^ These high-throughput capabilities accelerate the screening of libraries against new biological targets, facilitating the identification of potential lead compounds and providing detailed SAR information.^3^ This process also enables faster iterations in design-make-test-analyse (DMTA) cycles, contributing to the development of more effective and selective drug candidates for the treatment of human diseases.^6,8^ Moreover, automation further improves reproducibility and minimizes human error, thus making it invaluable for chemical biology, medicinal chemistry, and drug discovery.^9^ Likewise, computer-aided drug design (CADD) methods have gained immense importance over the past decades and nowadays are a key driving force for an accelerated drug discovery process due to reduced resources, time, and costs.^10–12^ The utilization of large virtual libraries and chemical spaces enables a fast exploration of chemically diverse compounds.^13–15^ Besides ‘in-stock’ molecules like those consolidated in the original ZINC database,^16^ ‘make-on-demand’ molecule libraries demonstrated high potential. To date, several databases of commercially available compounds are accessible containing up to trillions of molecules.^17^ However, proprietary libraries are reported to even cover up to 10^26^ molecules.^18^ Among the largest public databases for small molecules are Enamine REadily AccessibLe (REAL) Space,^15^ GalaXi,^19^ and eXplore^17^ or their combined implementation in the ZINC22 database.^20,21^ So far, these ultra-large screening libraries have successfully revealed novel lead compounds for disease-related enzymes.^10,13,22–27^ Structure-based virtual screenings are the most used approach, utilizing an established protein structure of the target of interest. However, screening an enumerated library exceeding hundreds of millions of molecules requires an enormous amount of computing power or cloud capacities limiting their broad application.^28^ Therefore, structurally or synthetically focused chemical spaces can be a more practical, time- and resource-efficient starting point, when a certain structural motif is already known.^29–31^

**Fig. 1:**
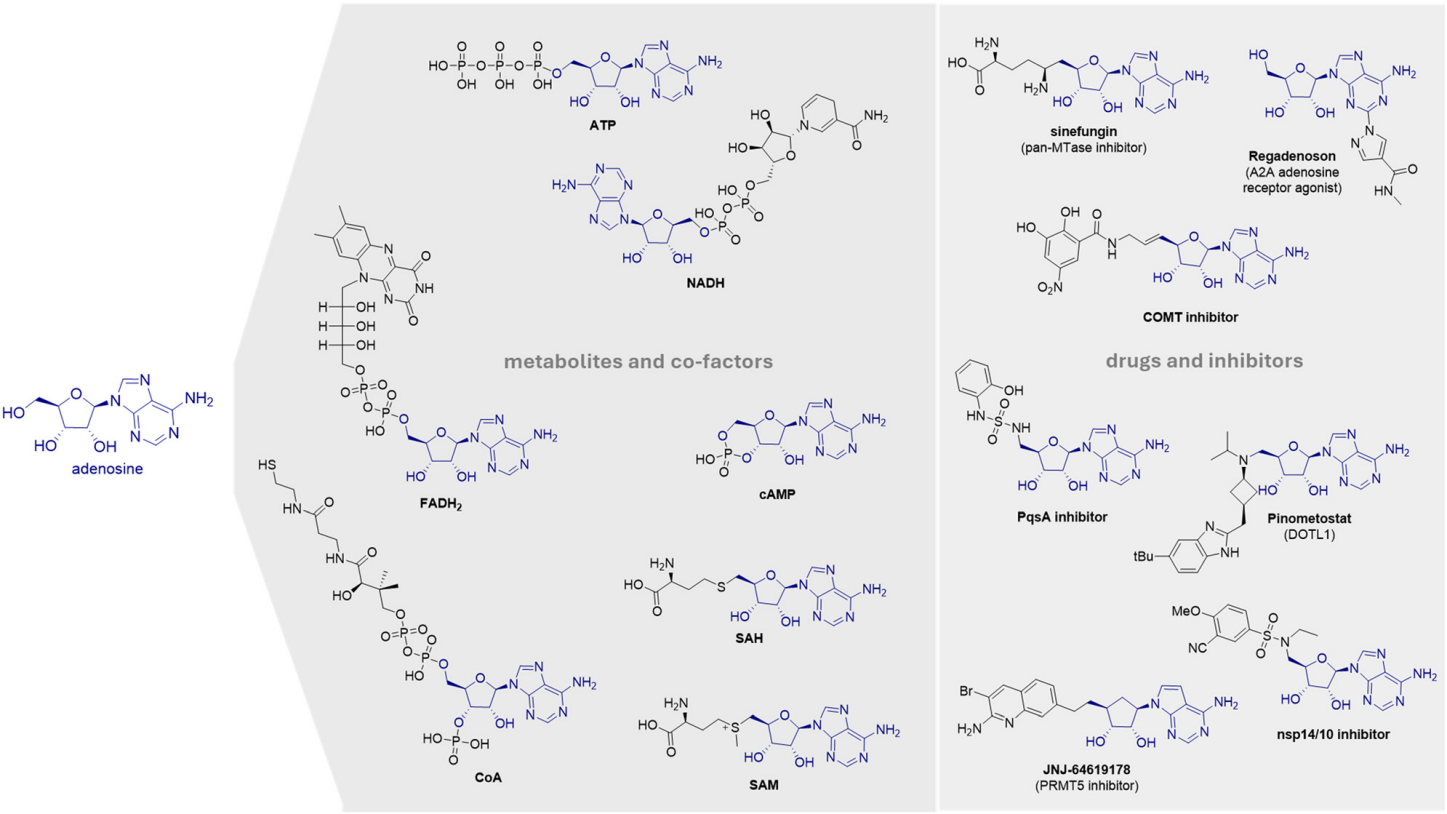
Examples for naturally metabolites and co-factors, and drugs and inhibitors containing the structural motif of adenosine (ATP = adenosine triphosphate, NADH = nicotinamide adenine dinucleotide, FADH_2_ = flavin adenine dinucleotide, CoA = coenzyme A, SAH = *S*-adenosyl-l-homocysteine, SAM = *S*-adenosyl-l-methionine).

Nucleosides, especially the naturally occurring, ubiquitous metabolite adenosine or molecules containing an adenosine scaffold, play fundamental roles in the human body and are involved in numerous critical biological pathways.^32^ Adenosine is involved in several extracellular and intracellular processes, including cell signalling *via* the adenosine receptors (AR), energy metabolism, and DNA methylation.^33^ Adenosine has been linked to several diseases, such as inflammation, ischemia, cardiovascular diseases,^34^ bacterial infections,^35^ and cancer.^36^ Coenzymes (SAM, SAH, NAD^+^/NADH, NADP^+^/NADPH, FAD/FADH_2_, CoAs), along with nucleic acids (RNA, DNA), second messengers (cAMP), and energy molecules (ATP, ADP, AMP) all share the adenosine scaffold, highlighting its relevance in a variety of fundamental physiological processes.^32^ These molecules are utilized by different protein families, including enzymes and receptors, most notably kinases, SAM-dependent methyltransferases, and adenosine receptors (Fig. 1). Adenosine was first approved by the FDA in 1989 for the treatment of supraventricular tachyarrhythmias.^34^ Since then, other adenosine-containing drugs such as regadenoson,^37^ fludarabine,^38^ remdesivir,^39^ or pinometostat^40^ have also been approved. In recent years, research focusing on adenosine-like inhibitors has emerged as a promising area in drug development. JNJ-646119178, a protein arginine methyltransferase 5 (PRMT5) inhibitor, is currently undergoing clinical trials as an anticancer drug,^41^ while other compounds such as sinefungin, a *pan*-MTase inhibitor,^42–47^ or Cladribin, an immunosuppressive agent,^48^ have also demonstrated their biological effects. Although adenosine analogues are still widely used in inhibitor development, targeting those specific pathways remains challenging due to the lack of selectivity among molecules that share the same scaffold. Still, exploration of diverse substitution patterns and SAR around the adenosine core can yield important insights about affinity and selectivity determining features supporting decision-making in drug development.^42^ Recently, methyltransferases (MTases) have received increased attention in drug development due to their involvement in pathologic processes such as cancer. Adenosine-substituted derivatives are promising inhibitors against the sleeping sickness,^49^ and as antitumor agents,^50^ while building block connection at 5′ position *via* an amide or sulfonamide functionality led to potent inhibitors of salicyl-AMP ligase (MbtA),^51^ anthranilyl-CoA synthetase PqsA^52^ as well as catechol-*O*-methyltransferase (COMT),^53^ and most recently of the SARS-CoV-2 nsp14 guanine-*N*^7^ MTase, among others.^54–57^

In this study, a parallel synthesis methodology to rapidly generate a chemically diverse substitution pattern at adenosine scaffolds resulting in a library of 42 compounds with good yields and high purity without the requirement of extensive purification despite a single extraction step is described. In 2014, Moukha-Chafiq and Reynolds established a similar strategy under the Pilot Scale Library Program of the NIH Roadmap Initiative by generating a library of 81 adenosine antibiotic-like derivatives.^58^ However, this approach relies on the time-consuming and expensive purification process, which hampers the throughput capability of this method, giving our method a practical advantage. Moreover, our library represents a small, focused compound library for a biologically relevant chemical space of adenosine analogues, enabling scientists to further explore and apply the potential activity for various biological targets. In this study, SAM-dependent methyltransferases were selected as a test case target class.

## Results and discussion

### Design and synthesis

**Scheme 1:**
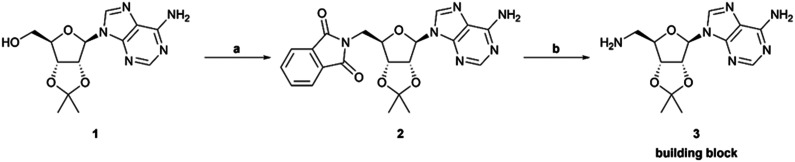
Synthesis of the main building block 3. Reagents and conditions: (a) phthalimide, PPh_3_, DIAD, THF, 0 °C to rt, 18 h, quant. (b) N_2_H_4_·H_2_O, EtOH, 80 °C, 4 h, 73%.

**Scheme 2:**
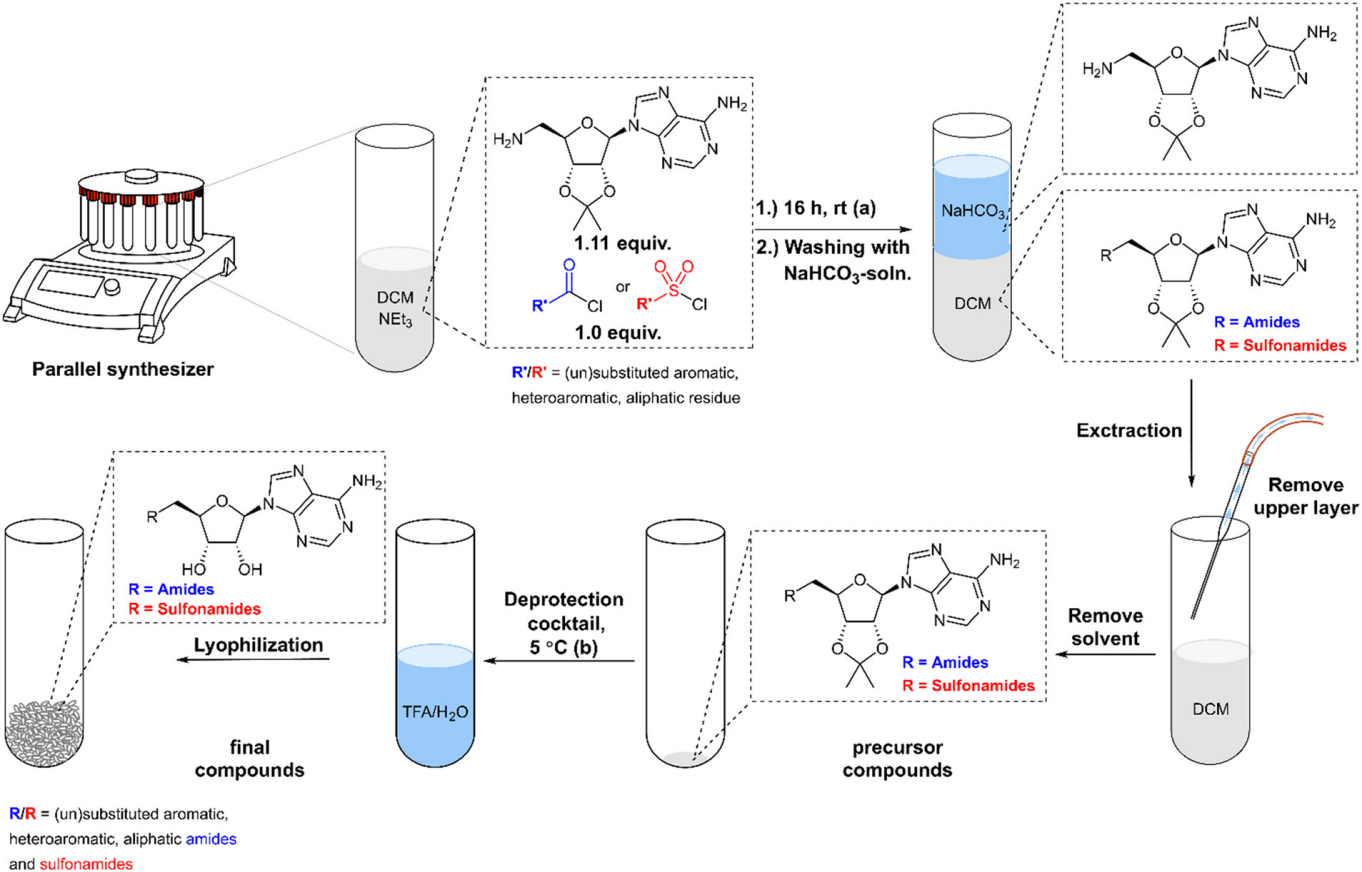
General Workflow of the established parallel synthesis method. Reagents: (a) TEA, DCM, rt, 18 h. (b) TFA:H_2_O (14 vol.%), 5 °C.

**Fig. 2:**
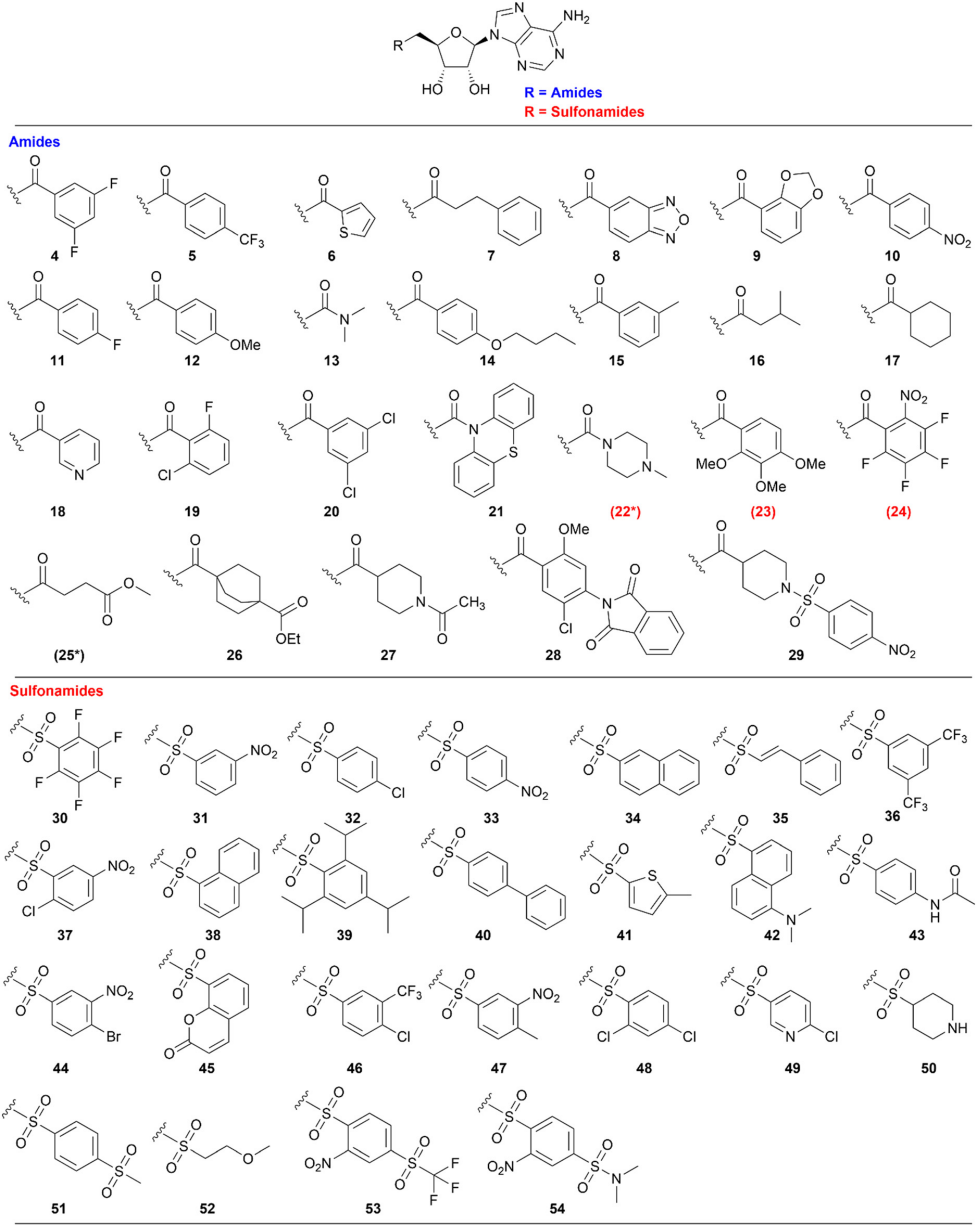
Structures of adenosine-based library obtained by the parallel synthesis. 
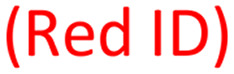
: no conversion. 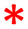
Conversion to the amide, but no final compound obtained. Occurred side products are listed in the SI (Fig. S2 and S3).

For the parallel synthesis of the desired amides and sulfonamides, 5′-amino-5′-deoxy-2’,3′-*O*-isopropylideneadeno-sine 3 was used as the main building block (BB). The synthetic procedure of the primary amine building block 3 was recently described for the synthesis of SAH-analogue DNMT2 inhibitors.^42^ In the latter study, the solubility of the respective amine 3 was identified in aqueous bicarbonate-solution, which allows the separation of this precursor from the constitutive reaction mixtures simply through extraction. By utilizing this finding, a synthesis protocol to generate amides and sulfonamides was established without further purification except this single extraction step. To obtain the primary amine 3, 2’,3′-*O*-isopropylideneadenosine 1 was converted in a Mitsunobu reaction to the respective phthalimide 2. A hydrazinolysis of the phthalimide 2 further revealed building block 3 (Scheme 1). By using the primary amine 3 as a precursor, a parallel synthesis method was established utilizing a parallel synthesizer. The instrument used in this study allows the generation of 16 derivatives simultaneously with the advantage that no further purification is required, and all steps can be carried out inside the tube (Scheme 2). For the synthesis of the amides and sulfonamides, the main scaffold 3 and the respective acyl and sulfonyl chlorides were therefore agitated in the parallel synthesizer for 18 h at room temperature. To allow complete conversion of the acyl/sulfonyl chlorides, the amine was used in excess (1.11 equiv.). The organic phase was then washed five times with saturated aqueous NaHCO_3_-solution and once with water, which completely removed the residual amine 3. This was specifically done by vacuuming the upper layer using a Pasteur pipette connected to a small hose. In the following step, the organic solvent was removed by distillation. The residue was treated with aqueous TFA solution (14 vol%) at 5 °C (cold room) until full removal of the protecting group was detected by LC-MS. After lyophilization, the final product was obtained as the respective trifluoroacetate salt. Utilizing this method, 51 in-house acyl and sulfonyl chlorides were used to create a diverse library of (un)substituted aromatic, heteroaromatic and aliphatic amides and sulfonamides (Fig. 2). Only 2 out of 51 reactions did not show conversion to the respective amides/sulfonamides (23, 24, labelled red) which resulted in a synthesis success of 96% with this parallel synthesis method with yields mostly above 40% (Table S1). Moreover, out of these 51 compounds, 44 compounds exhibit a purity higher than ≥95% and can be considered as successfully synthesized. These 86% of the compounds could be screened immediately for their biological effect without further purification. This demonstrates that the parallel synthesis of these amide and sulfonamide-based adenosine derivatives is a robust synthesis method giving fast access to an adenosine-based chemical space for SAR exploration. However, some structural motifs exhibited side reactions in the synthesis. LC-MS purity measurements revealed traces of a double acylated derivative for 7 leading to only 84% purity. After flash column chromatography only a reduced yield of 21% but with a purity of 99% was obtained. In general, the primary aromatic amine of the adenine exhibits low reactivity under these synthesis conditions, so a protecting group is not necessary. For compound 22, conversion to the respective amide *via* LC-MS was observed. However, through extraction with the saturated bicarbonate solution, phase transition of the precursor derivative (Scheme 2) into the aqueous phase was observed, failing to yield the desired final product. The calculated log *P*-value^59^ for the ribose-protected adenosine precursor of 22, (Table S2) of −1.5 (log *D*_7.0_ = −3.4)^60^ explains the prevalence of the respective compound for the aqueous phase. Except for compound 22, all calculated log *P*-values of the protected adenosine derivatives exhibit values higher than zero (Table S2), enabling their application in this parallel synthesis. Some substituted aromatic derivatives are known for their increased electrophilicity induced by the substituent's electronegativity,^61,62^ like pentafluoro-substitutions or NO_2_ substituents. Here, a reduced purity of 86% of the final compound 30 was identified, which could be related to the reactive character of the substituted benzene. Exhibiting a similar structural motif and reactivity as 30, however, the reaction did not display conversion for the synthesis of compound 24. One further limitation of this method is the incorporation of acid-labile structural motifs, like acetyl- or other acid-labile protecting groups. Due to the acid-based deprotection step, compound 43 could only be obtained in 66% acetylated and 33% deacetylated products. Also compound 25 underwent full methyl ester cleavage and LC-MS analysis showed the free acid as well as the formation of a succinimide ring closure (Fig. S2 and S3). In comparison, ethyl esters are more stable under these conditions and compound 26 only showed cleavage to the respective acid with less than 30% (Fig. S3). Moreover, it is important that the deprotection step is carried out at 5 °C or lower, otherwise a cleavage of the C–N glycosidic bond between adenine and ribose was observed in LC-MS, independently of the structural motif of the amides or sulfonamides (Fig. S1). With the increase of lipophilicity thus higher log *P* values, solubility in the deprotection cocktail is limited. Thus, a prolonged deprotection time is necessary to remove the isopropylidene protecting group. To be suitable for our method, several requirements of the BBs and reaction products must be fulfilled. Amine BBs like 3 must have a log *P* < 0 and contain no free acid or alcohol. Otherwise, phase separation into the aqueous phase is hampered. Similarly, for acid chlorides and sulfonyl chlorides, log *P* should not exceed 3.6 to allow phase separation of these BBs after hydrolysis and deprotonation. However, the incorporation of alcohols and acids is possible if suitable protecting groups are used for the acid-labile deprotection. Moreover, the amine should exhibit reactivity towards sulfonyl and acyl chlorides, thus featuring some nucleophilicity. In this regard, two amines fulfilling the requirements, morpholine and *N*-mehylpiperazine were selected as starting materials and were treated with the parallel method using two acyl and sulfonyl chlorides, respectively (Scheme S1). LC-MS analysis showed the occurrence of the respective amides and sulfonamides in the organic phase and no prevalence of the starting materials. These results indicated that an expanded scope of the parallel synthesis method can be created by changing the starting material beyond building block 3. To further explore the scope and limitations of this method, a secondary amine 55 was synthesized using 3 and acetone in a reductive amination (Scheme S2). Exemplarily, two acyl and sulfonyl chlorides were selected as BBs in the parallel synthesis with this secondary amine, respectively (56, 57). LC-MS analysis after liquid–liquid extraction revealed no phase transfer of the secondary amine 55 into the aqueous phase (Fig. S4). The calculated log *P* of 0.92 explains the prevalence of the respective compound for the organic phase. This shows that the synthesis is robust, but the liquid–liquid extraction has its limitations for lipophilicity of the BBs (acid/acid chloride/sulfonyl chloride log *P* ≤ 3.6), amines ≤ 0, and final compounds (after deprotection log *P* ≥ −1.8 ∼log *P* ≥ 0 prior deprotection). Taken together, a parallel synthesis method for adenosine-based amides and sulfonamides was established, which revealed a library of tool compounds with diverse substitution patterns containing both electron-rich and -poor aromatic, heteroaromatic, aliphatic and basic moieties. Only some strong electron-rich residues (23, OMe, +M-effect), some electron-poor ones with reactive residues (24, 30) and acid-labile groups (25, 26, 43) limit the scope of acetyl and sulfonyl chloride building blocks (BBs). The physicochemical properties (log *P*, log *D*_7.0_, p*K*_a_, TPSA) of the BBs, the protected adenosine-intermediates and the final compounds were calculated to correlate physicochemical properties to synthetic accessibility (yield). BBs of 13 and 22 exhibit log *P* values below 1.0 leading to a lower lipophilicity of the intermediates (close to 0), thus increasing their likelihood to be extracted into the aqueous phase (Table S2). Despite that, for BBs with a log *P* value higher than 1.0, there is no evident correlation between the lipophilicity and the yield. Furthermore, high TPSA values demonstrated to have no considerable effect on this method (Table S2, Fig. S5). Final compounds cover a wide range of TPSA and log *P* values (Fig. S5). Compared to a similar, already described procedure in literature,^58^ our synthesis was also carried out in a solution-phase manner using the primary amine 3 and sulfonyl chlorides. However, instead of carboxylic acids and coupling reagents, acetyl chlorides were used which reduces the common side effect of epimerization, if a stereocenter is prevalent. Moreover, utilizing reduced amount of chemicals, only amine, base, solvent and the acyl/sulfonyl chlorides, the established method can consider to be more economical (high-yields, efficient, inexpensive reagents, no labor-intensive workups, fast) and ecological (reduced solvent, minimal waste generation, energy efficient (rt)).^63^ This includes the omitted purification step, which is usually carried out with the high resource and solvent requirement of HPLC or flash column chromatography. This not only reduces material and costs but is also more time-efficient and the obtained compounds are suitable for direct biological evaluation. For comparison, four amides and sulfonamides (4, 6, 41 and 48) were selected, synthesized and purified exemplarily *via* HPLC and the solvent consumption was recorded. Each compound consumes appr. 900 mL of solvent, depending on the HPLC system and column size used (Table S3). Moreover, with the omitted purification step, scalability is simplified. Theoretically, the throughput of this method can be further increased using automated liquid–liquid extraction^64–66^ or parallel synthesizer with higher throughput^66,67^ as described previously for other reactions. However, this was not explicitly tested within this study.

### Biological evaluation – MTase application cases

**Fig. 3:**
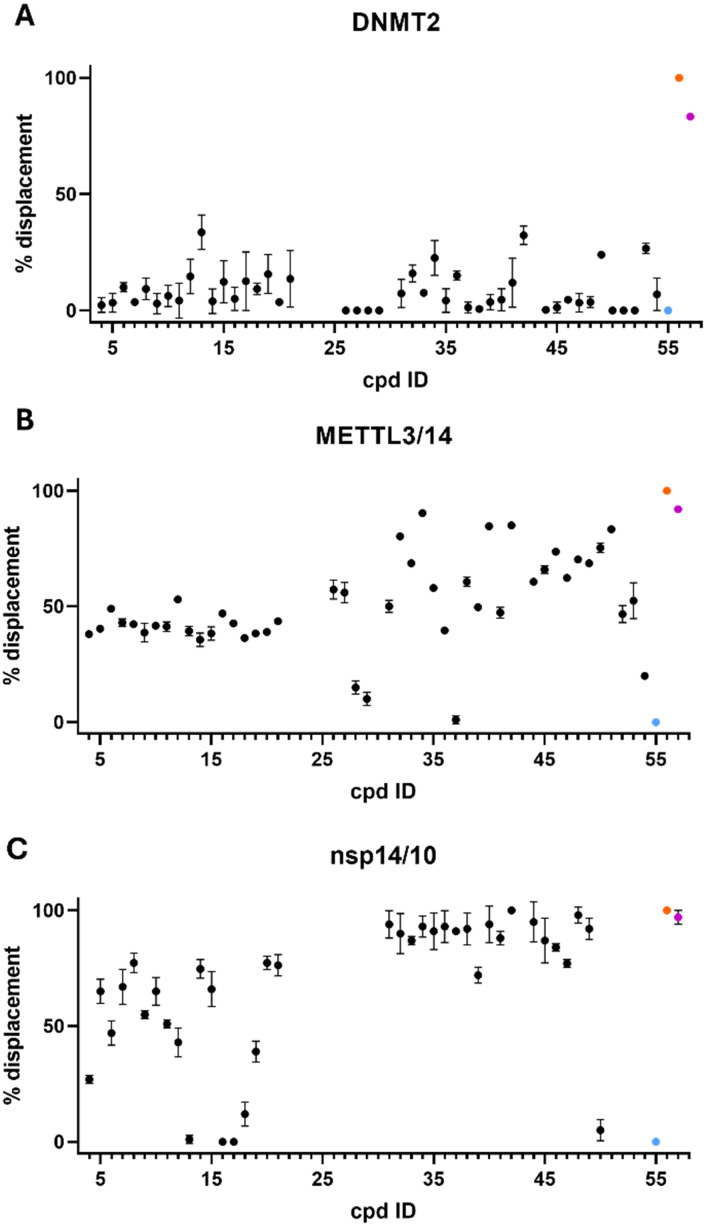
Scatter plots for the displacement (in %) of FTAD through the adenosine-based compounds (100 μM) derived from the parallel synthesis. Results shown for FTAD displacement out of the SAM-binding site of DNMT2 (A), METTL3/14 (B) and nsp14/10 (C). 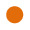 free probe (FTAD); 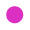 SAH/SFG; 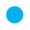 probe–enzyme complex. Each datapoint represents the mean ± SD of triplicate determination.

**Fig. 4:**
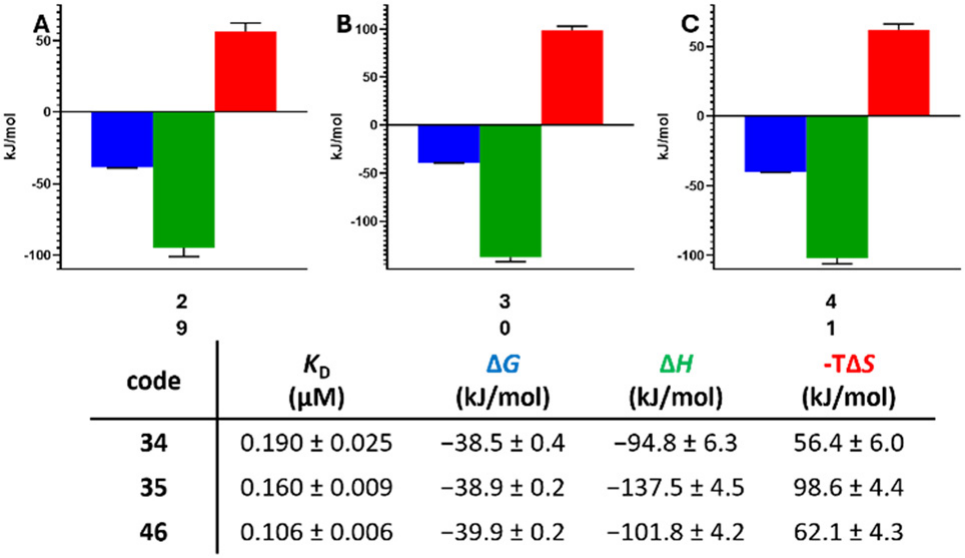
Thermodynamic signature plots from ITC experiments of compounds A) 34, B) 35 and C) 46. Experimentally derived binding affinity (*K*_D_), stoichiometry (*n*) and thermodynamic properties (Δ*G*, Δ*H*, −*T*Δ*S*) of compounds 34, 35 and 46 binding to nsp14/10 (mean ± standard deviation from duplicate measurements) (Fig. S15–17, Table S5).

To assess the biological activity of the synthesized adenosine-based library on potential targets, three different SAM-dependent MTases, DNMT2, METTL3/14, and SARS-CoV-2 nsp14/10, were selected as model systems to investigate the binding affinities of the obtained compound-library.^42,68,69^ These enzymes are also reported to bind 5-FAM-triazolyl-adenosyl-diaminobutyric acid (FTAD), a 5-FAM labelled SAH analogue that binds to the SAM binding site, and has been commonly reported as a displacement probe for various targets, such as histone methyltransferase 1 (MLL1),^70^*O*-methyltransferase BCDIN3D,^71^ or the flaviviral NS5 methyltransferase,^72^ but also for nsp14/10 (*K*_D_ = 1.29 ± 0.33 μM),^73^ DNMT2 (*K*_D_ = 6.4 ± 0.7 μM)^74^ and METTL3/14 (*K*_D_ = 1.1 ± 0.1 μM).^74^ Therefore, a fluorescence polarization displacement assay was established to distinguish between binders and non-binders within the adenosine-based library. To evaluate their binding on the respective targets, the percentage of FTAD displacement was determined by using the free probe (FTAD) as a positive control indicating 100% displacement, and the FTAD–enzyme complex as a negative control for 0% displacement. SAH or sinefungin (SFG) and DMSO were used in each measurement as the positive and negative control, respectively. All compounds were screened at a concentration of 100 μM (Fig. 3). For DNMT2, no or only low displacement of the FTAD-probe was observed for all compounds. This is in line with previous findings, showing that the amino acid side chain of SAM-derivatives, which is not present in the presented library (Fig. 2), is crucial for binding to DNMT2.^42^ The FP-assay for METTL3/14 revealed displacements above 45% for all tested compounds, some of which even displace FTAD more than 80%. Three compounds (34, 40, 42) showed a displacement similar to SFG (92%). Hence, these compounds were selected for affinity-determination displaying *K*_D_-values of 12.9 ± 2.0 μM, 16.1 ± 4.6 μM, and *K*_D_-value of 22.5 ± 1.4 μM, respectively (Fig. S13). As a *caveat*, compound 42 showed increased total fluorescence intensities due to the incorporated dansyl residue, leading to fluorescence interferences which influences the determined binding affinity of this compound (Table S4). Hence, this method revealed three compounds with potency equal to previously published inhibitors.^68^ For nsp14/10 a displacement for most of the compounds above 50% was observed and more than half of the molecules showed a displacement between 80–100%. Compounds with a displacement of more than 80% were selected and retested at lower concentrations (10 μM and 1 μM) to allow better discrimination (Fig. S6). Compounds 34, 35 and 46 were still exhibiting a displacement of 100% at 1 μM concentration. Thus, these compounds were selected for *K*_D_-determination *via* FP-assay. Previous studies already discovered compound 34 to bind and inhibit nsp14/10 in the nanomolar range.^75^ Additionally, compounds 31–33, 44 and 47 were identified to inhibit in the low micromolar range, exhibiting the same or a similar sub-structure as previously reported, affine inhibitors and therefore were not followed up.^54^ Herein, the compounds showed a binding affinity of less than 1 μM, which is below the limit for accurate *K*_D_-determination at the given enzyme concentration of 1 μM. A microscale thermophoresis (MST)-displacement assay was used in a second, more sensitive approach in which FTAD was also used as the fluorescence probe, but enzyme concentration could be reduced to 0.13 μM. Still, high affinity of compounds 34, 35 and 42 prohibited accurate *K*_D_-determination (Fig. S10–S12). Thus, the binding affinities of these compounds were determined by isothermal titration calorimetry (ITC), yielding *K*_D_ values of 0.19, 0.16 and 0.11 μM, respectively (Fig. 4 and S15–S17). Binding is dominated by enthalpy Δ*H* of up to −137.5 kJ mol^−1^ (35), partially compensated by an entropy penalty (Fig. 4, Table S5). The binding stoichiometry (*n*) of 0.4–0.6 indicates that probably not all protein is capable of ligand binding, properly due to misfolding (Table S5). Based on these findings, compounds featuring a sulfonamide displayed a better binding to both METTL3/14 and nsp14/10, compared to the amide series. This is indicated in the screening scatter plots, showing a clear cut at compound 30 (Fig. 3B/C). One could speculate that the different geometries of amides and sulfonamides cause this different binding behaviour. Especially nsp14/10 has been demonstrated to be targetable by adenosine-based sulfonamides.^56,76^

### Focused chemical space generation by virtual synthesis

**Fig. 5:**
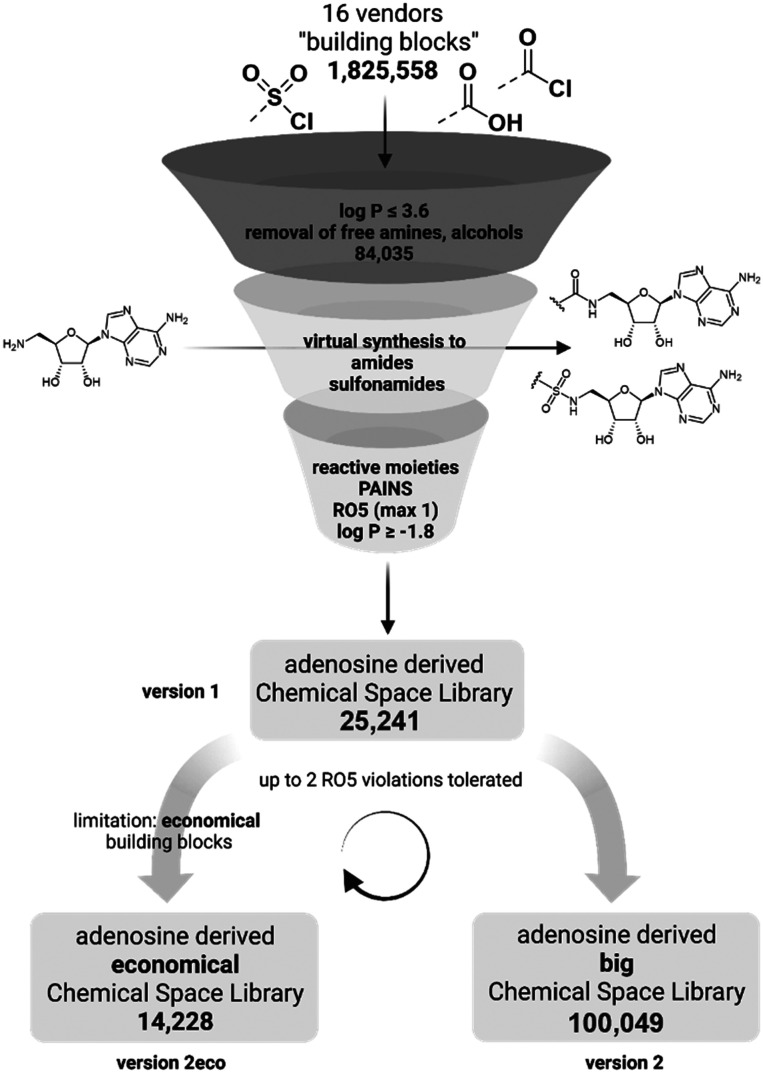
Workflow of chemical space generation by virtual synthesis of selected building blocks to generate a focused adenosine-based library.

The described synthesized and tested molecules are representatives of a much larger, but still very focussed, chemical space of adenosine derivatives. This chemical space can be accessed *via* the same fast and easy synthetic procedure and is virtually provided in SMILES format in the SI (SI, versions 1, 2 and 2eco). It can be used for virtual screening purposes against targets where competition with coenzymes like ATP (kinases), SAM (methyl transferases), NAD (oxidoreductases) or CoAs (specific transferases) and alike is desired. Starting from 16 chemical vendor catalogues (Table S6), BBs containing carboxylic acids, acyl and sulfonyl chlorides (1 825 558 BBs) were extracted and filtered for their lipophilicity log *P* <+3.5 and functional group properties, so their physicochemical properties are compatible with the extraction step (Scheme 2). However, a BB with a log *P* > 4 was still suitable for the extraction step (Table S2). The remaining 84 035 BBs were virtually reacted with 5′-amino-5′-deoxyadenosine to amides and sulfonamides, respectively. The obtained products were again filtered for their compatibility with the purification procedure (log *P* ≥ 1.8) and to be drug-like (one or fewer violations of the Lipinski rule of five [RO5]^77^ for oral bioavailability), non-reactive and no pan-assay interference compounds (PAINS).^78,79^ The resulting chemical space consists of 25 241 molecules (Fig. 5). All chemical space compounds are expected to be easily accessible from the commercially available BBs *via* the synthetic procedure described herein. Notably, this number can be easily expanded, if a single BB protection and final product deprotection step is performed which will also give access to molecules containing carboxylic acids, thiols, and additional amine- or hydroxy-functionalities. However, these procedures were not experimentally validated, and it is to be noted that the addition of the respective moieties to the already very polar sub-structure of 5′-amino-5′-deoxyadenosine amides and sulfonamides will lead to additional violations of the RO5 for H-bond donors or H-bond acceptors. Computational diversity analyses of the spaces were conducted by calculating the physicochemical properties such as TPSA, log *P*, logD, HBA, HBD, number of rotatable bonds and molecular weight of the respective libraries (Tables S7, S18–20) showing a broad distribution of properties despite the common building block 3. Likewise, similarity analysis using the iSIM method^80^ with RDKit fingerprints, resulted in median molecular similarities of 0.79, 0.78 and 0.78 for space1, space2 and space2eco, respectively (Table S8). Due to the common adenosine substructure, these values are higher compared to unbiased libraries^81^ and structural diversity solely originates from the used acid, acid chloride and sulfonyl chloride BBs. Still, these sub-structures can cover structurally different moieties with varying physicochemical properties.

### Virtual screening and hit validation

**Fig. 6:**
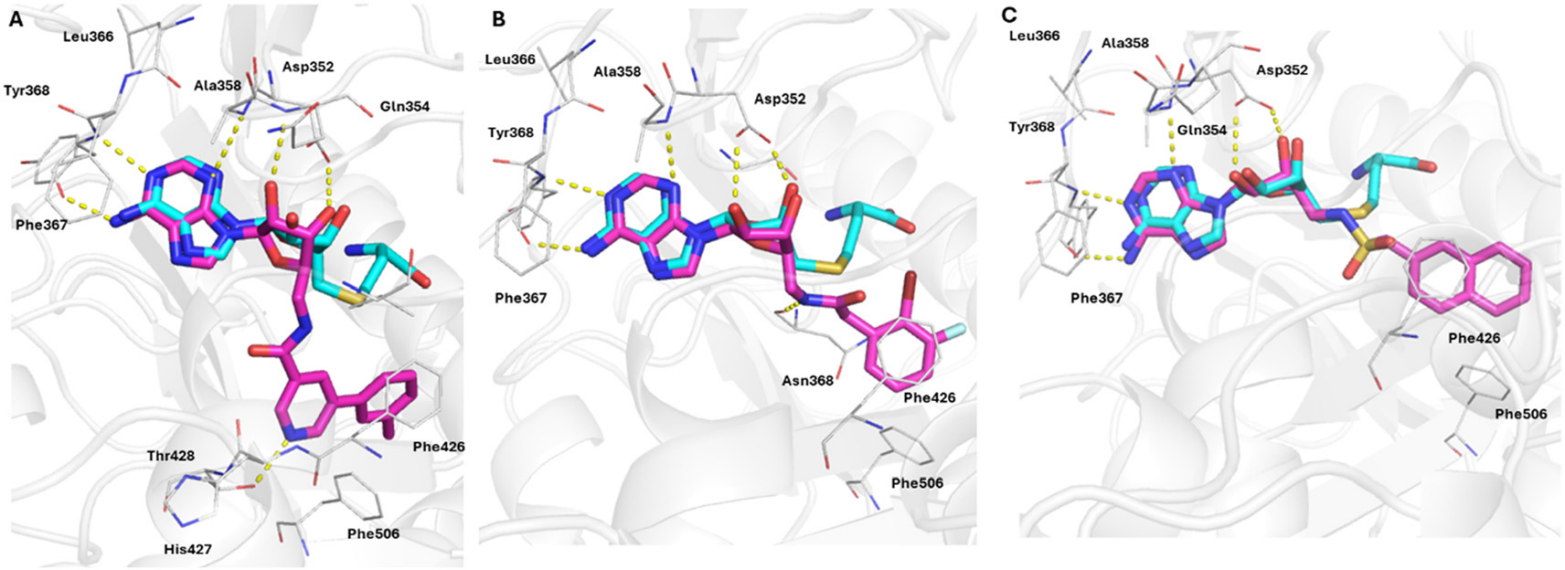
Predicted binding modes of 63 (A), 64 (B) and 65 (C) in complex with SARS-CoV-2 nsp14/10 (PDB: 7R2V). Ligands are depicted with magenta-colored carbon atoms, MTase with grey carbon atoms. Polar interactions are depicted as yellow dashed lines. Crystallographic reference ligand SAH is shown with cyan-colored carbon atoms for orientation.

**Scheme 3:**
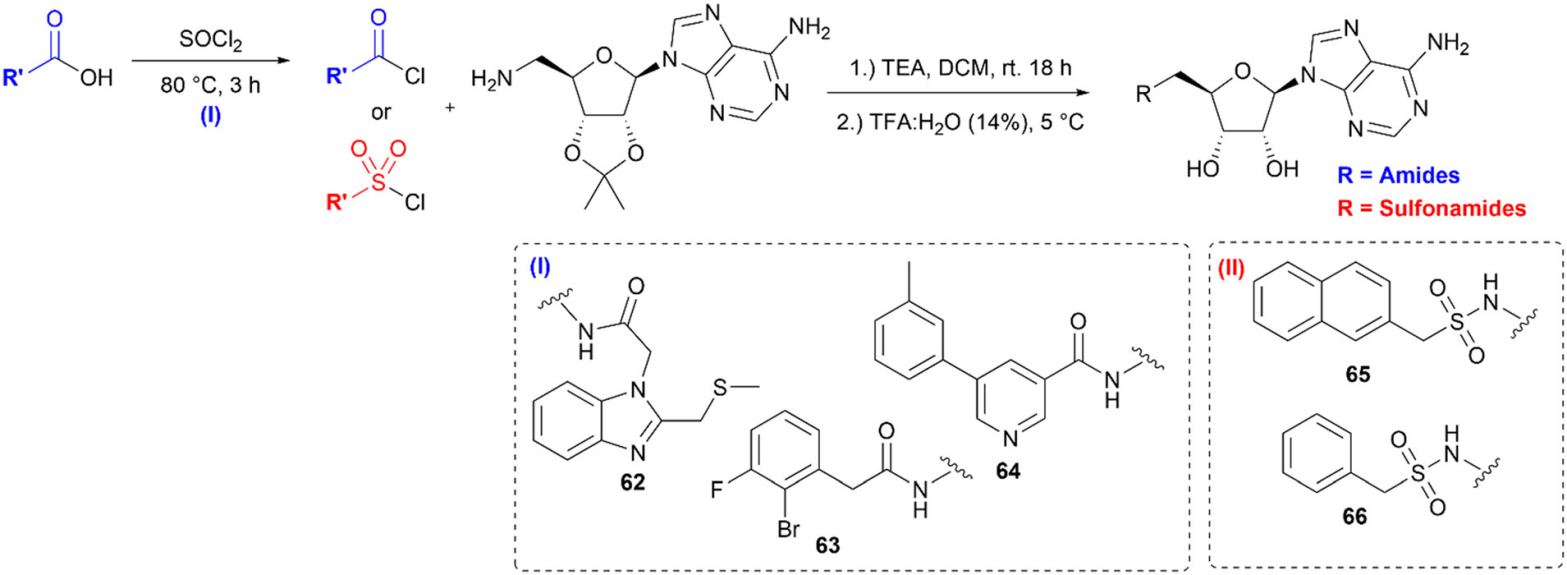
Synthesis scheme for the generation of adenosine-based virtual screening hits. 
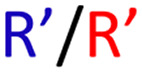
 = (un)substituted aromatic and heteroaromatic residues. I) Amide synthesis (blue) from acyl chloride and acid BBs, II) sulfonamide synthesis (red) from sulfonylchloride BBs.

To elucidate the potential of finding new sub-structures and expansion of the SAR analysis, the whole adenosine-based chemical space of 25 241 molecules was virtually screened against nsp14/10. Therefore, a template-based molecular docking was conducted using a nsp14/10-SAH complex structure (PDB-ID: 7R2V).^82^ The top 6% scoring poses (2110 molecules, ChemGauss score (−12.2)–(−16.6) kcal mol^−1^) were visually inspected and compared with the binding mode of SAH (re-docking RMSD: 1.75 Å, −10.4 kcal mol^−1^) (Fig. S21). Based on the predicted binding modes, the most prevalent structural motifs contain aromatic moieties oriented towards Phe426, which forms hydrophobic π–π stacking interactions and lies in the cap- binding pocket. This pocket is occupied by the aromatic amide and sulfonamide substituents, exhibiting also polar interactions with Thr428 (64) and Asn368 (63). The adenosine scaffolds occupy the SAM-binding pocket like SAH and exhibit polar interactions with the residues Thr368, Ala358, Gln354 and Asp352. Compared to our initially synthesized library (Fig. 2), most of the top-scored molecules exhibit an additional carbon atom between the amide and the aromatic residue (Fig. 6B and C) and favour amides over sulfonamides. However, the chemical space is biased by containing ten times more amides (24 470) than sulfonamides (2294). Among the top 6% scored molecules, five compounds which exhibit the most prevalent structural motifs were selected for synthesis and *in vitro* testing. The BBs in the virtual synthesis also include acids, which require conversion into the respective acyl chlorides with thionyl chloride (46–48, Scheme 3). The chlorination of 63 and 64 was successful. However, the conversion to the acyl chloride of 62 did not lead to the respective acyl chloride. The acyl and sulfonyl chlorides of 63–66 were subsequently transformed into the target molecules according to the above-mentioned method (Scheme 2). All synthesized compounds showed nsp14/10-binding with low micromolar potency (Table 1). However, a limitation of the focused chemical space of adenosine-amide/sulfonamide derivatives was identified. During the process of compound selection for synthesis, several BBs were either not available (even though labelled “in-stock” in ZINC) or had very high costs. Therefore, an alternative chemical space was created using the sub-set of “economical” (ZINC keyword “BB, economical”) BBs (Table S6). Additionally, this virtual screening library also tolerates up to two RO5 violations as HBA/HBD count is easily exceeded when building upon the adenosine scaffold. Consequently, that chemical space is more suited for early SAR exploration but may result in molecules with limited cellular permeability due to enhanced polarity. This economic adenosine-based library consists of 14 228 compounds and is provided as SMILES (SI, version2eco) to users for virtual synthesis campaigns with enzymes/receptors where the adenosine scaffold might be suitable for ligands. If cost is no limitation, a similarly less constrained (up to two RO5 violations tolerated) chemical space of 110 049 compounds is also provided (SI, version2).

**Table 1:** Experimentally derived yield, purity and binding affinity (*K*_D_) to nsp14/10 of adenosine-derivatives 62–66 and their docking scores (top score: −16.6 kcal mol^−1^)

cpd ID	Yield (%)	Purity (%)	*K*_D_ a (μM)	ChemGauss score (kcal mol^−1^)
62	0	n.d.	n.d.	−16.0
63	99	96	17.2 ± 5.5	−14.6
64	50	99	2.90 ± 0.38	−14.5
65	43	95	3.80 ± 0.78	−12.2
66	99	99	42.3 ± 11.1	−12.2
SAH	—	—	0.42 ± 0.07^b^	−10.4

a *K*_D_ determined by FP-assay.

b *K* ^app^_D_. n.d. signifies not determined.

## Conclusions

This study introduced a parallel synthesis method for the generation of a small, adenosine-based library of 51 (un)substituted aromatic, heteroaromatic and aliphatic adenosine derivatives containing both electron-rich and -poor substituents and basic residues with a synthesis success rate of 96%. This parallel synthesis method provides a suitable basis for rapidly generating adenosine derivatives as tool compounds in good yields and high purity, without the requirement for extensive purification. Therefore, it represents a more economical and ecological method compared to previously described procedures. To demonstrate the biological relevance of adenosine derivatives for SAR elucidation, an FP-assay was used to identify binders and asses their affinity for three model MTases DNMT2, METTL3/14 and nsp14/10. Sulfonamides 34, 40, 42 bound to METTL3/14 with low micromolar affinity. Compounds 34, 35 and 46 displayed binding affinities in the nanomolar range for nsp14/10 (Fig. 6 and S15–S17). Such 5′-amino-5′-deoxyadenosine sulfonamide scaffolds represent promising inhibitors which have been described previously for other targets, like salicyl-AMP ligase (MbtA),^51^ and anthranilyl-CoA synthetase PqsA^52^ but most recently for the inhibition of the SARS-CoV nsp14-methyltransferase.^54–57^ Additionally, a broader chemical space of 5′-amino-5′-deoxyadenosine amides and sulfonamides was generated by virtual synthesis of the respective amine 3 with commercially available BBs. A structure-based virtual screening of this library led to the identification of four further nsp14/10 ligands 63–66 with low micromolar affinity (Table 1). Due to limited accessibility and high cost of some BBs from this around 28k molecules large space, a smaller, more cost-efficient library of around 14k molecules was generated as well as a bigger less restrained variant of 100k molecules. All three spaces are provided for virtual screenings and subsequent DIY synthesis with the described procedure as SMILES (SI; versions 1, 2 and 2eco).

## Experimental section

### Synthetic procedures and characterization data

#### General information

All reagents and solvents were purchased in analytical grade quality and used without further purification. Reaction progress was monitored by thin-layer chromatography using Alugram Xtra F254 silica plates from MACHEREY-NAGEL. NMR spectra were recorded on a Bruker Fourier 300 MHz, on a Bruker Avance Neo 400 MHz or on a Magritek Spinsolve 60 MHz spectrometer. Chemical shifts are indicated in parts per million (ppm), with the residual proton peaks of the solvent (DMSO-*d*_6_ or CD_3_OD from Deutero GmbH) as internal standard. The identities and purities of the final compounds were determined by a combined HPLC/ESI-MS analysis using either an Agilent 1100 series HPLC system with an analytical Agilent Zorbax SB-Aq (4.6 × 150 mm) or a Waters Alliance e2695 HPLC system with an analytical MZ-Aqua Perfect C18 (4.6 × 250 mm, 10 μm) column coupled to a Waters Acquity QDa single quadrupole detector. Both instruments were used with the mobile phase: MeCN/H_2_O + 0.1% HCOOH = 10 : 90 to 100 : 0; (flow rate: 0.7 mL min^−1^; *t* = 10 min) and a column at 40 °C oven temperature. Samples were applied using 5−10 μL injections with AUC evaluation at 210 nm and 254 nm. Electro spray ionization (ESI-MS) mass spectra were recorded on an Agilent 1100 series LC/MSD ion trap spectrometer in the positive ion mode. All tested compounds have a purity ≥95%. Fourier-transformed ATR-corrected IR spectra were measured on an Avatar 330 single-crystal spectrometer from ThermoNicolet. Melting points (uncorrected) were determined using a Schorpp Gerätetechnik MPM-H3 melting point device using semi-open capillaries. Specific rotations *α*^20^_D_ were determined by a Krüss P3000 polarimeter and are given in deg cm^3^ g^−1^ dm^−1^. The parallel synthesis was pursued in a Synthesis 1 parallel synthesizer from Heidolph Instruments GmbH & CO. KG.

#### General procedure parallel synthesis

To a solution of 3 (50 mg, 0.16 mmol, 1.11 equiv.) in DCM (5 mL), a sulfonyl chloride or acyl chloride (0.14 mmol, 1.0 equiv.) and NEt_3_ (0.024 mL, 0.17 mmol, 1.2 equiv.) were added, and the mixture was agitated in the parallel synthesizer at rt overnight. Saturated aqueous NaHCO_3_-solution (5 × 5 mL) was added to the parallel synthesizer tubes and the organic phase was washed through agitation of the parallel synthesizer. Then, the DCM was removed under reduced pressure at 40 °C and the residues were suspended in 14 vol% TFA : H_2_O (0.3 mL : 1.7 mL) and kept at 5 °C until full conversion to the deprotected derivatives was detected by LC-MS analysis. The solution was dried by lyophilization to give the respective trifluoroacetate salts.

#### General procedure acyl chlorides

The carboxylic acid (0.14 mmol, 1.0 equiv.) was dissolved in thionyl chloride (4 mL) and heated under reflux for 3 h. The thionyl chloride was removed under reduced pressure at 40 °C through co-distillation with cyclohexane and the obtained residue was used in the next reaction without further purification.

#### 9-((3a*R*,4*R*,6*R*,6a*R*)-6-(Aminomethyl)-2,2-dimethyltetrahydro-furo[3,4-*d*][1,3]dioxol-4-yl)-9*H*-purin-6-amine (3)

To a solution of 2′,3′-*O*-isopropylidenadenosine 1 (14.2 g, 46.1 mmol, 1.0 equiv.) in THF (300 mL) were added triphenylphosphine (12.1 g, 46.1 mmol, 1.0 equiv.) and phthalimide (6.78 g, 46.1 mmol). After the suspension was cooled to 0 °C, DIAD (9.0 mL, 46.1 mmol, 1.0 equiv.) was added dropwise and the reaction was stirred at rt for 18 h. The precipitations were filtered off and the obtained solid (12.0 g, 27.5 mmol, 1.0 equiv.) was dissolved in EtOH (450 mL). Under vigorous stirring, hydrazine hydrate (9.4 mL, 303 mmol, 11.0 equiv.) was added slowly and the mixture was heated to 80 °C for 4 h. After cooling down, the colorless precipitates were removed, and the filtrate was concentrated under reduced pressure at 40 °C. The obtained solid was purified by column chromatography (DCM : MeOH = 10 : 1 + 0.5% TEA) to afford 3 (6.11 g, 20.0 mmol, 73%) as a colorless solid. ^1^H NMR (300 MHz, DMSO-d_6_): *δ*/ppm = 8.36 (s, 1H), 8.16 (s, 1H), 7.33 (s, 2H), 6.08 (d, *J* = 3.2 Hz, 1H), 5.45 (dd, *J* = 6.3, 3.2 Hz, 1H), 4.98 (dd, *J* = 6.3, 2.8 Hz, 1H), 4.10 (td, *J* = 5.8, 2.9 Hz, 1H), 2.72 (dd, *J* = 5.8, 2.9 Hz, 2H), 1.53 (s, 3H), 1.32 (s, 3H). ^13^C NMR (75.5 MHz, DMSO-d_6_): *δ*/ppm = 156.1, 152.7, 149.0, 140.0, 119.2, 113.2, 89.1, 86.9, 82.7, 81.6, 43.6, 27.1, 25.2. FT-IR: *ν*/cm^−1^ = 3130, 1676, 1602, 1569, 1472, 1372, 1302, 1242, 1208, 1079, 1006, 873, 838, 798, 781, 689. *α*^20^_D_ = −31° (10 mg mL^−1^; MeOH). mp: 199–201 °C.

#### *N*-(((2*R*,3*S*,4*R*,5*R*)-5-(6-Amino-9*H*-purin-9-yl)-3,4-dihydroxy-tetrahydrofuran-2-yl)methyl)-3,5-difluorobenzamide (4)

The compound was prepared from 3 (50 mg, 0.16 mmol) according to the general procedure to afford the final product as a colorless trifluoroacetate salt (23 mg, 0.05 mmol, 24%, 0.3 equiv. TFA). ^1^H NMR (300 MHz, CD_3_OD): *δ*/ppm = 8.42 (s, 1H), 8.19 (s, 1H), 7.37–7.27 (m, 2H), 7.08 (tt, *J* = 8.9, 2.4 Hz, 1H), 5.97 (d, *J* = 4.8 Hz, 1H), 4.72 (t, *J* = 5.1 Hz, 1H), 4.31 (t, *J* = 5.1 Hz, 1H), 4.22–4.14 (m, 1H), 3.81–3.62 (m, 2H). ^13^C NMR (75.5 MHz, CD_3_OD): *δ*/ppm = 167.6, 166.0 (d, *J* = 12.1 Hz), 162.7 (d, *J* = 12.3 Hz), 152.4, 149.9, 145.9, 144.4, 139.0 (t, *J* = 8.4 Hz), 120.8, 111.5 (d, *J* = 26.6 Hz), 107.8 (t, *J* = 25.8 Hz), 91.0, 84.9, 75.2, 72.8, 42.7. FT-IR: *ν*/cm^−1^ = 3094, 2360, 1693, 1593, 1484, 1444, 1318, 1200, 1123, 991, 837, 799, 763, 723, 668. *α*^20^_D_ = −34° (10 mg mL^−1^; DMSO). mp: 136–138 °C. ESI-MS: *m*/*z* calculated for C_17_H_16_F_2_N_6_O_4_ [M + H]^+^ = 407.13 (100%); found: 407.01 (100%). Purity: 96% (HPLC, 254 nm, MeCN/H_2_O = 10 : 90 to 100 : 0 + 0.1% HCOOH, *t*_R_ = 6.71 min).

#### *N*-(((2*R*,3*S*,4*R*,5*R*)-5-(6-Amino-9*H*-purin-9-yl)-3,4-dihydroxytetra-hydrofuran-2-yl)methyl)-4-(trifluoromethyl)benzamide (5)

The compound was prepared from 3 (50 mg, 0.16 mmol) according to the general procedure to afford the final product as a colorless trifluoroacetate salt (39 mg, 0.06 mmol, 43%, 1.5 equiv. TFA). ^1^H NMR (300 MHz, CD_3_OD): *δ*/ppm = 8.42 (s, 1H), 8.18 (s, 1H), 7.86 (d, *J* = 8.1 Hz, 2H), 7.66 (d, *J* = 8.1 Hz, 2H), 5.97 (d, *J* = 5.0 Hz, 1H), 4.71 (t, *J* = 5.1 Hz, 1H), 4.30 (t, *J* = 4.8 Hz, 1H), 4.23–4.16 (m, 1H), 3.80–3.63 (m, 2H). ^13^C NMR (75.5 MHz, CD_3_OD): *δ*/ppm = 169.1, 152.5, 149.9, 146.1, 144.3, 139.2, 134.14 (q, *J* = 32.5 Hz), 129.1, 127.0, 126.53 (d, *J* = 3.9 Hz), 123.4, 90.9, 85.0, 75.2, 72.9, 42.9. FT-IR: *ν*/cm^−1^ = 3311, 2362, 1671, 1578, 1455, 1326, 1200, 1128, 1068, 1016, 836, 800, 773, 723. *α*^20^_D_ = −32° (10 mg mL^−1^; MeOH). mp: 47–49 °C. ESI-MS: *m*/*z* calculated for C_18_H_17_F_3_N_6_O_4_ [M + H]^+^ = 439.13 (100%); found: 439.22 (100%). Purity: 97% (HPLC, 254 nm, MeCN/H_2_O = 10 : 90 to 100 : 0 + 0.1% HCOOH, *t*_R_ = 7.76 min).

#### *N*-(((2*R*,3*S*,4*R*,5*R*)-5-(6-Amino-9*H*-purin-9-yl)-3,4-dihydroxytetra-hydrofuran-2-yl)methyl)thiophene-2-carboxamide (6)

The compound was prepared from 3 (50 mg, 0.16 mmol) according to the general procedure to afford the final product as a colorless trifluoroacetate salt (93 mg, 0.13 mmol, 93%, 3.2 equiv. TFA). ^1^H NMR (300 MHz, CD_3_OD): *δ*/ppm = 8.34–8.26 (m, 1H), 8.09–7.97 (m, 1H), 7.49–7.39 (m, 2H), 6.98–6.86 (m, 1H), 5.97–5.82 (m, 1H), 4.71–4.60 (m, 1H), 4.36–4.29 (m, 1H), 4.20–4.06 (m, 1H), 3.72–3.53 (m, 2H). ^13^C NMR (75.5 MHz, CD_3_OD): *δ*/ppm = 164.7, 163.8, 163.4, 162.9, 152.5, 149.7, 146.1, 144.3, 139.8, 131.9, 129.7, 128.8, 91.1, 85.1, 75.0, 72.5, 42.0. FT-IR: *ν*/cm^−1^ = 3105, 2476, 1669, 1527, 1507, 1455, 1354, 1185, 1130, 840, 801, 723. *α*^20^_D_ = −15° (10 mg mL^−1^; MeOH). mp: 63–65 °C. ESI-MS: *m*/*z* calculated for C_15_H_17_N_6_O_4_S [M + H]^+^ = 377.10 (100.0%); found: 377.00 (100%). Purity: 98% (HPLC, 254 nm, MeCN/H_2_O = 10 : 90 to 100 : 0 + 0.1% HCOOH, *t*_R_ = 7.06 min).

#### *N*-(((2*R*,3*S*,4*R*,5*R*)-5-(6-Amino-9*H*-purin-9-yl)-3,4-dihydroxy-tetrahydrofuran-2-yl)methyl)-3-phenylpropanamide (7)

The compound was prepared from 3 (50 mg, 0.16 mmol) according to the general procedure to afford the final product as a colorless trifluoroacetate salt. The crude residue was purified by RP-flash column chromatography. (19 mg, 0.03 mmol, 21%, 2.2 equiv. TFA). ^1^H NMR (300 MHz, CD_3_OD): *δ*/ppm = 8.38 (s, 1H), 8.30 (s, 1H), 7.26–7.02 (m, 5H), 5.94 (d, *J* = 5.5 Hz, 1H), 4.60 (t, *J* = 5.2 Hz, 1H), 4.11–4.02 (m, 2H), 3.58–3.40 (m, 2H), 2.84 (t, *J* = 8.3, 6.9 Hz, 2H), 2.45 (t, *J* = 8.4, 6.9 Hz, 2H). ^13^C NMR (75.5 MHz, CD_3_OD): *δ*/ppm = 175.5, 152.5, 146.1, 144.2, 142.1, 129.5, 129.3, 127.2, 90.7, 85.2, 75.3, 72.8, 42.2, 38.9, 32.8. FT-IR: *ν*/cm^−1^ = 3297, 2359, 1694, 1555, 1507, 1424, 1200, 1135, 1077, 833, 799, 723, 701. *α*^20^_D_ = −30° (10 mg mL^−1^; MeOH). mp: 53–55 °C. ESI-MS: *m*/*z* calculated for C_19_H_23_N_6_O_4_ [M + H]^+^ = 399.18 (100%); found: 399.42 (100%). Purity: 99% (HPLC, 254 nm, MeCN/H_2_O = 10 : 90 to 100 : 0 + 0.1% HCOOH, *t*_R_ = 6.15 min).

#### *N*-(((2*R*,3*S*,4*R*,5*R*)-5-(6-Amino-9*H*-purin-9-yl)-3,4-dihydroxy-tetrahydrofuran-2-yl)methyl)benzo[*c*][1,2,5]oxadiazole-5-carboxamide (8)

The compound was prepared from 3 (50 mg, 0.16 mmol) according to the general procedure to afford the final product as a colorless trifluoroacetate salt (55 mg, 0.11 mmol, 79%, 1.0 equiv. TFA). ^1^H NMR (300 MHz, CD_3_OD): *δ*/ppm = 9.23–8.90 (m, 3H), 8.70 (s, 1H), 8.50 (s, 1H), 8.42 (s, 1H), 8.12 (dd, *J* = 9.4, 1.0 Hz, 1H), 7.93 (dd, *J* = 9.4, 1.4 Hz, 1H), 5.96 (d, *J* = 5.6 Hz, 1H), 4.72 (t, *J* = 5.3 Hz, 1H), 4.29–4.20 (m, 1H), 4.2–4.09 (m, 1H), 3.79–3.55 (m, 2H). ^13^C NMR (75.5 MHz, CD_3_OD): *δ*/ppm = 165.0, 159.2 (q, *J* = 35.1 Hz), 151.7, 149.1, 148.9, 148.6, 146.8, 142.4, 137.9, 131.4, 119.1, 116.5, 115.8, 88.0, 83.3, 73.3, 71.3, 41.9. FT-IR: *ν*/cm^−1^ = 2922, 2360, 1649, 1423, 1197, 1131, 798, 768, 722. *α*^20^_D_ = −30° (10 mg mL^−1^; DMSO). mp: 62–64 °C. ESI-MS: *m*/*z* calculated for C_17_H_17_N_8_O_5_ [M + H]^+^ = 413.13 (100%); found: [M + H]^+^ = 413.00 (100%). Purity: 96% (HPLC, 254 nm, MeCN/H_2_O = 10 : 90 to 100 : 0 + 0.1% HCOOH, t_R_ = 3.57 min).

#### *N*-(((2*R*,3*S*,4*R*,5*R*)-5-(6-Amino-9*H*-purin-9-yl)-3,4-dihydroxy-tetrahydrofuran-2-yl)methyl)benzo[d][1,3]dioxole-4-carbox-amide (9)

The compound was prepared from 3 (50 mg, 0.16 mmol) according to the general procedure to afford the final product as a beige trifluoroacetate salt (35 mg, 0.07 mmol, 50%, 0.6 equiv. TFA). ^1^H NMR (300 MHz, DMSO-d_6_): *δ*/ppm = 8.74 (s, 1H), 8.62 (s, 1H), 8.31 (s, 1H), 7.89 (t, *J* = 5.8 Hz, 1H), 7.24 (dd, *J* = 8.1, 1.3 Hz, 1H), 7.07 (dd, *J* = 7.8, 1.3 Hz, 1H), 6.92 (t, *J* = 7.8 Hz, 1H), 6.05 (dd, *J* = 5.9, 1.1 Hz, 2H), 5.94 (d, *J* = 5.6 Hz, 1H), 4.66 (t, *J* = 5.3 Hz, 1H), 4.21 (dd, *J* = 5.0, 3.9 Hz, 1H), 4.14–4.08 (m, 1H), 3.69–3.63 (m, 2H). ^13^C NMR (75.5 MHz, DMSO-d_6_): *δ*/ppm = 163.4, 152.4, 148.7, 147.7, 145.2, 142.0, 121.7, 121.2, 119.1, 116.3, 111.1, 101.6, 88.0, 83.2, 73.3, 71.1, 41.1. FT-IR: *ν*/cm^−1^ = 3324, 1642, 1589, 1540, 1453, 1241, 1204, 1135, 1124, 1056, 1035, 918, 859, 753, 723, 669. *α*^20^_D_ = −20° (10 mg mL^−1^; DMSO). mp: 91–93 °C. ESI-MS: *m*/*z* calculated for C_18_H_19_N_6_O_6_ [M + H]^+^ = 415.14 (100%); found: 415.03 (100%). Purity: 97% (HPLC, 254 nm, MeCN/H_2_O = 10 : 90 to 100 : 0 + 0.1% HCOOH, *t*_R_ = 6.40 min).

#### *N*-(((2*R*,3*S*,4*R*,5*R*)-5-(6-Amino-9*H*-purin-9-yl)-3,4-dihydroxy-tetrahydrofuran-2-yl)methyl)-4-nitrobenzamide (10)

The compound was prepared from 3 (50 mg, 0.16 mmol) according to the general procedure to afford the final product as a colorless trifluoroacetate salt (41 mg, 0.07 mmol, 50%, 1.3 equiv. TFA). ^1^H NMR (300 MHz, CD_3_OD): *δ*/ppm = 8.41 (s, 1H), 8.22–8.13 (m, 3H), 7.92–7.87 (m, 2H), 5.97 (d, *J* = 5.0 Hz, 1H), 4.72 (t, *J* = 5.0 Hz, 1H), 4.32 (t, *J* = 4.9 Hz, 1H), 4.23–4.16 (m, 1H), 3.81–3.65 (m, 2H). ^13^C NMR (75.5 MHz, CD_3_OD): *δ*/ppm = 168.4, 152.5, 151.0, 149.8, 146.1, 144.3, 141.1, 129.7, 124.6, 120.8, 91.0, 84.9, 75.2, 72.9, 42.9. FT-IR: *ν*/cm^−1^ = 3312, 2478, 1667, 1599, 1523, 1429, 1346, 1198, 1135, 869, 833, 799, 720. *α*^20^_D_ = −34° (10 mg mL^−1^; DMSO). mp: 98–100 °C. ESI-MS: *m*/*z* calculated for C_17_H_18_N_7_O_6_ [M + H]^+^ = 416.13 (100%); found: 416.02 (100%). Purity: 96% (HPLC, 254 nm, MeCN/H_2_O = 10 : 90 to 100 : 0 + 0.1% HCOOH, *t*_R_ = 5.94 min).

#### *N*-(((2*R*,3*S*,4*R*,5*R*)-5-(6-Amino-9*H*-purin-9-yl)-3,4-dihydroxy-tetrahydrofuran-2-yl)methyl)-4-fluorobenzamide (11)

The compound was prepared from 3 (50 mg, 0.16 mmol) according to the general procedure to afford the final product as a colorless trifluoroacetate salt (41 mg, 0.08 mmol, 57%, 1.4 equiv. TFA). ^1^H NMR (300 MHz, CD_3_OD): *δ*/ppm = 8.38 (s, 1H), 8.11 (s, 1H), 7.79–7.66 (m, 2H), 7.11–6.91 (m, 2H), 5.93 (d, *J* = 5.1 Hz, 1H), 4.69 (t, *J* = 5.1 Hz, 1H), 4.29 (t, *J* = 4.9 Hz, 1H), 4.20–4.12 (m, 1H), 3.79–3.56 (m, 2H). ^13^C NMR (75.5 MHz, CD_3_OD): *δ*/ppm = 169.3, 167.8, 164.5, 152.5, 149.8, 146.1, 144.4, 131.8, 130.89 (d, *J* = 9.0 Hz), 116.5, 116.2, 90.9, 85.1, 75.2, 72.8, 42.6. FT-IR: *ν*/cm^−1^ = 3094, 2360, 1673, 1633, 1603, 1505, 1445, 1198, 1133, 851, 799, 764, 722. *α*^20^_D_ = −26° (10 mg mL^−1^; MeOH). mp: 113–115 °C. ESI-MS: *m*/*z* calculated for C_17_H_18_FN_6_O_4_ [M + H]^+^ = 389.14 (100%); found: 389.06 (100%). Purity: 95% (HPLC, 254 nm, MeCN/H_2_O = 10 : 90 to 100 : 0 + 0.1% HCOOH, *t*_R_ = 5.89 min).

#### *N*-(((2*R*,3*S*,4*R*,5*R*)-5-(6-Amino-9*H*-purin-9-yl)-3,4-dihydroxy-tetrahydrofuran-2-yl)methyl)-4-methoxybenzamide (12)

The compound was prepared from 3 (50 mg, 0.16 mmol) according to the general procedure to afford the final product as a colorless trifluoroacetate salt (52 mg, 0.04 mmol, 29%, 8.4 equiv. TFA). ^1^H NMR (300 MHz, CD_3_OD): *δ*/ppm = 8.36 (s, 1H), 8.05 (s, 1H), 7.60 (d, *J* = 8.9 Hz, 2H), 6.81 (d, *J* = 8.9 Hz, 2H), 5.92 (d, *J* = 4.9 Hz, 1H), 4.67 (t, *J* = 5.1 Hz, 1H), 4.31 (t, *J* = 4.9 Hz, 1H), 4.17–4.11 (m, 1H), 3.71 (s, 3H), 3.69–3.56 (m, 2H). ^13^C NMR (75.5 MHz, CD_3_OD): *δ*/ppm =170.0, 163.9, 152.3, 149.7, 145.9, 144.5, 130.1, 127.3, 114.7, 91.0, 85.3, 75.2, 72.7, 55.9, 42.3. FT-IR: *ν*/cm^−1^ = 3327, 2360, 1692, 1606, 1573, 1506, 1438, 1311, 1257, 1199, 1135, 844, 767, 723. *α*^20^_D_ = −44° (10 mg mL^−1^; MeOH). mp: 59−61 °C. ESI-MS: *m*/*z* calculated for C_18_H_21_N_6_O_5_ [M + H]^+^ = 401.16 (100%); found: 401.08 (100%). Purity: 98% (HPLC, 254 nm, MeCN/H_2_O = 10 : 90 to 100 : 0 + 0.1% HCOOH, *t*_R_ = 5.83 min).

#### 3-(((2*R*,3*S*,4*R*,5*R*)-5-(6-Amino-9*H*-purin-9-yl)-3,4-dihydroxy-tetrahydrofuran-2-yl)methyl)-1,1-dimethylurea (13)

The compound was prepared from 3 (50 mg, 0.16 mmol) according to the general procedure to afford the final product as a colorless trifluoroacetate salt (33 mg, 0.09 mmol, 64%, 0.3 equiv. TFA). ^1^H NMR (300 MHz, CD_3_OD): *δ*/ppm = 8.45 (s, 1H), 8.35 (s, 1H), 6.01 (d, *J* = 5.4 Hz, 1H), 4.74 (t, *J* = 5.3 Hz, 1H), 4.27 (t, *J* = 4.6 Hz, 1H), 4.13 (q, *J* = 5.3 Hz, 1H), 3.54 (d, *J* = 5.7 Hz, 2H), 2.89 (d, *J* = 1.0 Hz, 6H). ^13^C NMR (75.5 MHz, CD_3_OD): *δ*/ppm = 159.6, 152.2, 148.7, 146.4, 142.3, 119.4, 89.1, 84.4, 73.9, 71.4, 42.2, 35.1. FT-IR: *ν*/cm^−1^ = 3323, 1677, 1632, 1539, 1421, 1199, 1132, 1051, 1026, 1004, 831, 799, 765, 722. *α*^20^_D_ = −15° (10 mg mL^−1^; MeOH). mp: 57–59 °C. ESI-MS: *m*/*z* calculated for C_17_H_17_N_8_O_5_ [M + H]^+^ = 338.16 (100%), found: 338.13 (100%). Purity: 100% (HPLC, 254 nm, MeCN/H_2_O = 10 : 90 to 100 : 0 + 0.1% HCOOH, *t*_R_ = 1.90 min).

#### *N*-(((2*R*,3*S*,4*R*,5*R*)-5-(6-Amino-9*H*-purin-9-yl)-3,4-dihydroxy-tetrahydrofuran-2-yl)methyl)-4-butoxybenzamide (14)

The compound was prepared from 3 (50 mg, 0.16 mmol) according to the general procedure to afford the final product as a colorless trifluoroacetate salt (31 mg, 0.07 mmol, 48%,0.6 equiv. TFA). ^1^H NMR (300 MHz, CD_3_OD): *δ*/ppm = 8.44 (s, 1H), 8.18 (s, 1H), 7.81–7.73 (m, 2H), 7.02–6.92 (m, 2H), 6.05 (d, *J* = 5.1 Hz, 1H), 4.43 (dd, *J* = 5.3, 4.3 Hz, 1H), 4.30 (q, *J* = 4.8 Hz, 1H), 4.06 (t, *J* = 6.4 Hz, 2H), 3.81 (ddd, *J* = 43.4, 14.2, 4.9 Hz, 2H), 1.87–1.72 (m, 2H), 1.64–1.42 (m, 2H), 1.02 (t, *J* = 7.4 Hz, 3H). ^13^C NMR (75.5 MHz, CD_3_OD): *δ*/ppm = 170.3, 163.5, 154.1, 148.5, 143.7, 130.2, 127.4, 115.2, 91.0, 85.3, 75.1, 72.8, 69.0, 42.4, 32.4, 20.2, 14.1. FT-IR: *ν*/cm^−1^ = 3323, 2959, 1678, 1607, 1549, 1505, 1475, 1425, 1300, 1256, 1203, 1136, 1069, 844, 801, 768, 723, 669. *α*^20^_D_ = +48° (10 mg mL^−1^; MeOH). mp: 114–116 °C. ESI-MS: m/z calculated for C_21_H_27_N_6_O_5_ [M + H]^+^ = 443.2 (100%); found: 443.00 (100%). Purity: 98% (HPLC, 254 nm, MeCN/H_2_O = 10 : 90 to 100 : 0 + 0.1% HCOOH, *t*_R_ = 4.88 min).

#### *N*-(((2*R*,3*S*,4*R*,5*R*)-5-(6-Amino-9*H*-purin-9-yl)-3,4-dihydroxy-tetrahydrofuran-2-yl)methyl)-3-methylbenzamide (15)

The compound was prepared from 3 (50 mg, 0.16 mmol) according to the general procedure to afford the final product as a colorless trifluoroacetate salt (83 mg, 0.14 mmol, 99%, 0.2 equiv. TFA). ^1^H NMR (300 MHz, CD_3_OD): *δ*/ppm = 8.42 (s, 1H), 8.06 (s, 1H), 7.58–7.48 (m, 2H), 7.37–7.24 (m, 2H), 5.99 (d, *J* = 5.0 Hz, 1H), 4.76 (t, *J* = 5.1 Hz, 1H), 4.38 (t, *J* = 4.9 Hz, 1H), 4.24 (q, *J* = 4.9 Hz, 1H), 3.87–3.62 (m, 2H), 2.33 (s, 3H). ^13^C NMR (75.5 MHz, CD_3_OD): *δ*/ppm = 169.4, 151.7, 148.5, 145.6, 142.8, 138.2, 134.1, 132.1, 128.1, 127.4, 124.0, 119.5, 89.7, 83.9, 73.8, 71.4, 41.1, 20.0. FT-IR: *ν*/cm^−1^ = 3312, 1674, 1633, 1605, 1583, 1423, 1331, 1199, 1133, 1066, 977, 892, 833, 799, 784, 743, 722, 685. *α*^20^_D_ = −29° (10 mg mL^−1^; MeOH). mp: 105–107 °C. ESI-MS: *m*/*z* calculated for C_17_H_17_N_8_O_5_ [M + H]^+^ = 385.16 (100%), found: 385.13 (100%). Purity: 99% (HPLC, 254 nm, MeCN/H_2_O = 10 : 90 to 100 : 0 + 0.1% HCOOH, *t*_R_ = 4.12 min).

#### *N*-(((2*R*,3*S*,4*R*,5*R*)-5-(6-Amino-9*H*-purin-9-yl)-3,4-dihydroxy-tetrahydrofuran-2-yl)methyl)-3-methylbutanamide (16)

The compound was prepared from 3 (50 mg, 0.16 mmol) according to the general procedure to afford the final product as a colorless trifluoroacetate salt (58 mg, 0.13 mmol, 85%, 0.5 equiv. TFA). ^1^H NMR (300 MHz, CD_3_OD): *δ*/ppm = 8.40 (s, 1H), 8.32 (s, 1H), 5.95 (d, *J* = 5.4 Hz, 1H), 4.67 (t, *J* = 5.3 Hz, 1H), 4.15 (t, *J* = 5.2, 4.0 Hz, 1H), 4.13–4.02 (m, 1H), 3.50 (t, *J* = 5.2 Hz, 2H), 2.08–1.90 (m, 3H), 0.92–0.78 (m, 7H). ^13^C NMR (75.5 MHz, CD_3_OD): *δ*/ppm = 175.9, 153.3, 150.0, 147.2, 143.9, 90.7, 85.2, 75.4, 72.9, 46.3, 42.3, 27.3, 22.7. FT-IR: *ν*/cm^−1^ = 3271, 2959, 1674, 1644, 1553, 1508, 1467, 1426, 1371, 1333, 1200, 1134, 1071, 837, 800, 723. *α*^20^_D_ = −34° (10 mg mL^−1^; MeOH). mp: decomp. >149 °C. ESI-MS: *m*/*z* calculated for C_17_H_17_N_8_O_5_ [M + H]^+^ = 351.2 (100%), found: 351.8 (100%). Purity: 96% (HPLC, 254 nm, MeCN/H_2_O = 10 : 90 to 100 : 0 + 0.1% HCOOH, *t*_R_ = 4.44 min).

#### *N*-(((2*R*,3*S*,4*R*,5*R*)-5-(6-Amino-9*H*-purin-9-yl)-3,4-dihydroxy-tetrahydrofuran-2-yl)methyl)cyclohexanecarboxamide (17)

The compound was prepared from 3 (50 mg, 0.16 mmol) according to the general procedure to afford the final product as a colorless trifluoroacetate salt (73 mg, 0.14 mmol, 99%, 0.1 equiv. TFA). ^1^H NMR (300 MHz, CD_3_OD): *δ*/ppm = 8.44 (s, 1H), 8.36 (s, 1H), 5.99 (d, *J* = 5.5 Hz, 1H), 4.72 (t, *J* = 5.3 Hz, 1H), 4.18 (t, *J* = 4.6 Hz, 1H), 4.12 (q, *J* = 5.0 Hz, 1H), 3.64–3.40 (m, 2H), 2.18 (tt, *J* = 11.7, 3.1 Hz, 1H), 1.81–1.17 (m, 10H). ^13^C NMR (75.5 MHz, CD_3_OD): *δ*/ppm = 178.2, 151.9, 148.6, 145.8, 142.6, 119.5, 89.3, 83.9, 73.8, 71.4, 45.1, 40.7, 29.4– 29.3, 25.5, 25.4. FT-IR: *ν*/cm^−1^ = 3195, 2930, 2855, 1634, 1506, 1451, 1423, 1331, 1199, 1133, 1051, 1025, 1004, 895, 832, 799, 722. *α*^20^_D_ = −31° (10 mg mL^−1^; MeOH). mp: 93–95 °C. ESI-MS: *m*/*z* calculated for C_17_H_17_N_8_O_5_ [M + H]^+^ = 377.19 (100%), found: 377.17 (100%). Purity: 100% (HPLC, 254 nm, MeCN/H_2_O = 10 : 90 to 100 : 0 + 0.1% HCOOH, *t*_R_ = 3.80 min).

#### *N*-(((2*R*,3*S*,4*R*,5*R*)-5-(6-Amino-9*H*-purin-9-yl)-3,4-dihydroxy-tetrahydrofuran-2-yl)methyl)nicotinamide (18)

The compound was prepared from 3 (50 mg, 0.16 mmol) according to the general procedure to afford the final product as a colorless trifluoroacetate salt (42 mg, 0.11 mmol, 77%, 0.2 equiv. TFA). ^1^H NMR (300 MHz, CD_3_OD): *δ*/ppm = 9.01 (s, 1H), 8.75 (s, 1H), 8.45 (s, 1H), 8.42–8.32 (m, 1H), 8.23 (s, 1H), 7.69 (s, 1H), 6.00 (d, *J* = 5.0 Hz, 1H), 4.74 (t, *J* = 5.1 Hz, 1H), 4.32 (t, *J* = 4.9 Hz, 1H), 4.22 (q, *J* = 5.4 Hz, 1H), 3.84–3.68 (m, 2H). ^13^C NMR (75.5 MHz, CD_3_OD): *δ*/ppm = 166.0, 151.3, 149.7, 148.6, 146.4, 145.0, 142.9, 137.5, 124.7, 119.5, 89.6, 83.5, 73.8, 71.5, 41.5. FT-IR: *ν*/cm^−1^ = 3101, 1667, 1455, 1423, 1199, 1132, 833, 799, 722. *α*^20^_D_ = −4° (10 mg mL^−1^; MeOH). mp: 62–64 °C. ESI-MS: *m*/*z* calculated for C_17_H_17_N_8_O_5_ [M + H]^+^ = 372.14 (100%) found: 372.11 (100%). Purity: 99% (HPLC, 254 nm, MeCN/H_2_O = 10 : 90 to 100 : 0 + 0.1% HCOOH, *t*_R_ = 2.67 min).

#### *N*-(((2*R*,3*S*,4*R*,5*R*)-5-(6-Amino-9*H*-purin-9-yl)-3,4-dihydroxy-tetrahydrofuran-2-yl)methyl)-2-chloro-6-fluorobenzamide (19)

The compound was prepared from 3 (50 mg, 0.16 mmol) according to the general procedure to afford the final product as a colorless trifluoroacetate salt (91 mg, 0.15 mmol, 99%, 0.2 equiv. TFA). ^1^H NMR (300 MHz, CD_3_OD): *δ*/ppm = 8.41 (s, 1H), 7.83 (s, 1H), 7.51–7.38 (m, 1H), 7.30 (d, *J* = 8.2, 0.9 Hz, 1H), 7.16 (td, *J* = 8.6, 1.0 Hz, 1H), 5.99 (d, *J* = 6.1 Hz, 1H), 4.81 (dd, *J* = 6.2, 5.0 Hz, 1H), 4.35–4.25 (m, 2H, H-9), 3.99–3.89 (m, 1H), 3.74–3.63 (m, 1H). ^13^C NMR (75.5 MHz, CD_3_OD): *δ*/ppm = 164.0, 160.9, 157.6, 152.2, 148.4, 145.8, 142.7, 131.78–131.4, 125.6, 125.35–125.3, 119.7, 114.4, 114.1, 89.7, 84.2, 73.5, 71.5, 41.3. FT-IR: *ν*/cm^−1^ = 3324, 1643, 1609, 1575, 1477, 1450, 1433, 1333, 1248, 1199, 1134, 1071, 899, 835, 787, 723. *α*^20^_D_ = −50° (10 mg mL^−1^; MeOH). mp: 88–90 °C. ESI-MS: *m*/*z* calculated for C_17_H_17_N_8_O_5_ [M + H]^+^ = 423.10 (100%); found: 423.07 (100%). Purity: 100% (HPLC, 254 nm, MeCN/H_2_O = 10 : 90 to 100 : 0 + 0.1% HCOOH, *t*_R_ = 5.35 min).

#### *N*-(((2*R*,3*S*,4*R*,5*R*)-5-(6-Amino-9*H*-purin-9-yl)-3,4-dihydroxy-tetrahydrofuran-2-yl)methyl)-3,5-dichlorobenzamide (20)

The compound was prepared from 3 (50 mg, 0.16 mmol) according to the general procedure to afford the final product as a colorless trifluoroacetate salt (100 mg, 0.15 mmol, 99%, 0.1 equiv. TFA). ^1^H NMR (300 MHz, DMSO-d_6_): *δ*/ppm = 8.93 (t, *J* = 5.8 Hz, 1H), 8.36 (s, 1H), 8.07 (s, 1H), 7.87 (d, *J* = 1.9 Hz, 2H), 7.81 (t, *J* = 1.9 Hz, 1H), 7.32 (s, 2H), 5.87 (d, *J* = 6.0 Hz, 1H), 4.75 (t, *J* = 5.6 Hz, 1H), 4.19 (t, *J* = 4.3 Hz, 1H), 4.13–4.02 (m, 1H), 3.71–3.51 (m, 2H).^13^C NMR (75.5 MHz, DMSO-d_6_): *δ*/ppm = 164.0, 156.2, 152.6, 149.3, 140.4, 137.6, 134.3, 130.7, 126.2, 119.5, 87.8, 82.9, 72.5, 71.3, 41.9. FT-IR: *ν*/cm^−1^ = 3078, 1644, 1566, 1506, 1463, 1416, 1199, 1136, 1100, 1025, 1004, 869, 833, 802, 758, 722, 665. *α*^20^_D_ = −2° (10 mg mL*α*^20^_D_; MeOH). mp: 108–110 °C. ESI-MS: *m*/*z* calculated for C_17_H_16_Cl_2_N_6_O_4_ [M + H]^+^ = 439.07 (100.0%); found: 439.05 (100%). Purity: 100% (HPLC, 254 nm, MeCN/H_2_O = 10 : 90 to 100 : 0 + 0.1% HCOOH, *t*_R_ = 4.50 min).

#### *N*-(((2*R*,3*S*,4*R*,5*R*)-5-(6-Amino-9*H*-purin-9-yl)-3,4-dihydroxy-tetrahydrofuran-2-yl)methyl)-10*H*-phenothiazine-10-carboxamide (21)

The compound was prepared from 3 (50 mg, 0.16 mmol) according to the general procedure to afford the final product as a grey trifluoroacetate salt (108 mg, 0.14 mmol, 99%, 2.6 equiv. TFA). ^1^H NMR (300 MHz, DMSO-d_6_): *δ*/ppm = 8.68–8.48 (m, 3H), 8.31 (s, 1H), 7.54 (dd, *J* = 7.9, 1.4 Hz, 2H), 7.44 (dd, *J* = 7.6, 1.6 Hz, 2H), 7.29 (td, *J* = 7.6, 1.7 Hz, 2H), 7.20 (td, *J* = 7.5, 1.5 Hz, 2H), 6.69 (t, *J* = 5.8 Hz, 1H), 5.92 (d, *J* = 6.1 Hz, 1H), 4.73–4.65 (m, 1H), 4.20–4.14 (m, 1H), 4.12–4.00 (m, 1H), 3.53–3.30 (m, 2H). ^13^C NMR (75.5 MHz, DMSO-d_6_): *δ*/ppm = 159.7–158.4 (q, *J* = 32.1 Hz), 159.3, 158.8, 158.4, 154.4, 153.4, 148.6, 141.9, 138.9, 132.2, 128.0, 127.5, 127.1, 126.5, 88.0, 83.5, 73.2, 71.4, 42.7. FT-IR: *ν*/cm^−1^ = 3210, 1674, 1201, 1126, 1026, 834, 800, 762, 721. *α*^20^_D_ = −6° (10 mg mL^−1^; DMSO). mp: 59–61 °C. ESI-MS: *m*/*z* calculated for C_23_H_21_N_7_O_4_S [M + H]^+^ = 492.15 (100%); found: 492.01 (100%). Purity: 98% (HPLC, 254 nm, MeCN/H_2_O = 10 : 90 to 100 : 0 + 0.1% HCOOH, *t*_R_ = 6.91 min).

#### Ethyl 4-((((2*R*,3*S*,4*R*,5*R*)-5-(6-amino-9*H*-purin-9-yl)-3,4-di-hydroxytetrahydrofuran-2-yl)methyl)carbamoyl)bicyclo[2.2.2]octane-1-carboxylate (26)

The compound was prepared from 3 (50 mg, 0.16 mmol) according to the general procedure to afford the final product as a colorless solid. The product was purified by RP-flash column chromatography (15 mg, 0.03 mmol, 21%). ^1^H NMR (300 MHz, CD_3_OD): *δ*/ppm = 8.25 (d, *J* = 13.6 Hz, 2H), 5.91 (d, *J* = 5.4 Hz, 1H), 4.76 (t, *J* = 5.3 Hz, 1H), 4.18 (dd, *J* = 5.2, 4.1 Hz, 1H), 4.06 (q, *J* = 7.2 Hz, 3H), 3.63 (dd, *J* = 14.1, 5.5 Hz, 1H), 3.48 (dd, *J* = 14.1, 4.7 Hz, 1H), 1.85–1.67 (m, 12H), 1.20 (t, *J* = 7.1 Hz, 3H). ^13^C NMR (75.5 MHz, CD_3_OD): *δ*/ppm = 180.5, 179.0, 142.2, 122.5, 90.5, 84.7, 74.8, 72.7, 61.6, 42.1, 40.1, 39.8, 29.0, 14.5. FT-IR: *ν*/cm^−1^ = 3346, 2951, 2492, 1703, 1621, 1458, 1259, 1070. *α*^20^_D_ = −28° (10 mg mL^−1^; MeOH). mp: 109–111 °C. ESI-MS: *m*/*z* calculated for C_22_H_30_N_6_O_6_ [M + H]^+^ = 475.23 (100%); found: 475.00 (100%). Purity: 99% (HPLC, 254 nm, MeCN/H_2_O = 10 : 90 to 100 : 0 + 0.1% HCOOH, *t*_R_ = 3.57 min).

#### 1-Acetyl-*N*-(((2*R*,3*S*,4*R*,5*R*)-5-(6-amino-9*H*-purin-9-yl)-3,4-di-hydroxytetrahydrofuran-2-yl)methyl)piperidine-4-carbox-amide (27)

The compound was prepared from 3 (50 mg, 0.16 mmol) according to the general procedure to afford the final product as a grey trifluoroacetate salt (20 mg, 0.04 mmol, 29%, 0.6 equiv. TFA). ^1^H NMR (300 MHz, CD_3_OD): *δ*/ppm = 6.98–6.55 (m, 2H), 4.41 (d, *J* = 5.4 Hz, 1H), 3.14 (t, *J* = 5.3 Hz, 1H), 2.95–2.84 (m, 1H), 2.65–2.48 (m, 2H), 2.34 (d, *J* = 13.7 Hz, 1H), 2.06–1.89 (m, 2H), 1.60–1.45 (m, 1H), 1.14–0.99 (m, 1H), 0.96–0.82 (m, 1H), 0.49 (s, 3H), 0.26–−0.12 (m, 4H). ^13^C NMR (75.5 MHz, CD_3_OD): *δ*/ppm = 177.5, 171.5, 153.3, 90.7, 85.1, 75.2, 72.9, 47.0, 43.8, 42.2, 30.0, 29.6, 3.5, 21.2. FT-IR: *ν*/cm^−1^ = 3329, 1677, 1428, 1323, 1201, 1134, 836, 800, 723. *α*^20^_D_ = −20° (10 mg mL^−1^; MeOH). mp: 81–83 °C. ESI-MS: *m*/*z* calculated for C_18_H_25_N_7_O_5_ [M + H]^+^ = 420.19 (100%); found: 420.00 (100%). Purity: 95% (HPLC, 254 nm, MeCN/H_2_O = 10 : 90 to 100 : 0 + 0.1% HCOOH, *t*_R_ = 2.46 min).

#### *N*-(((2*R*,3*S*,4*R*,5*R*)-5-(6-Amino-9*H*-purin-9-yl)-3,4-dihydroxy-tetrahydrofuran-2-yl)methyl)-5-chloro-4-(1,3-dioxoisoindolin-2-yl)-2-methoxybenzamide (28)

The acyl chloride was prepared according to the general procedure for acyl chlorides. The compound was prepared from 3 (50 mg, 0.16 mmol) according to the general procedure to afford the final product as a colorless trifluoroacetate salt (66 mg, 0.08 mmol, 57%, 2.1 equiv. TFA). ^1^H NMR (300 MHz, CD_3_OD): *δ*/ppm = 8.48 (s, 1H), 8.16 (s, 1H), 8.01–7.80 (m, 5H), 7.29 (s, 1H), 6.02 (d, *J* = 4.9 Hz, 1H), 4.79 (t, *J* = 5.1 Hz, 1H), 4.40 (t, *J* = 4.8 Hz, 1H), 4.25 (q, *J* = 4.9 Hz, 1H), 3.92–3.82 (m, 1H), 3.80 (s, 3H), 3.73 (dd, *J* = 14.1, 4.9 Hz, 1H). ^13^C NMR (75.5 MHz, CD_3_OD): *δ*/ppm = 167.7, 166.5, 157.7, 136.1, 134.6, 133.1, 132.5, 126.1, 126.0, 124.9, 116.2, 91.1, 85.1, 75.2, 72.9, 57.4, 42.3. FT-IR: *ν*/cm^−1^ = 3330, 2361, 1725, 1694, 1446, 1402, 1203, 1137, 722. *α*^20^_D_ = −27° (10 mg mL^−1^; MeOH). mp:135–137 °C. ESI-MS: *m*/*z* calculated for C_26_H_22_ClN_7_O_7_ [M + H]^+^ = 580.13 (100%); found: 580.00 (100%). Purity: 98% (HPLC, 254 nm, MeCN/H_2_O = 10 : 90 to 100 : 0 + 0.1% HCOOH, *t*_R_ = 4.31 min).

#### *N*-(((2*R*,3*S*,4*R*,5*R*)-5-(6-Amino-9*H*-purin-9-yl)-3,4-dihydroxy-tetrahydrofuran-2-yl)methyl)-1-((4-nitrophenyl)sulfonyl) piperidine-4-carboxamide (29)

The acyl chloride was prepared according to the general procedure for acyl chlorides. The compound was prepared from 3 (50 mg, 0.16 mmol) according to the general procedure to afford the final product as a colorless trifluoroacetate salt (56 mg, 0.06 mmol, 43%, 3.3 equiv. TFA). ^1^H NMR (300 MHz, *d*_6_-DMSO): *δ*/ppm = 8.44 (d, *J* = 8.4 Hz, 2H), 8.31 (s, 1H), 8.14 (s, 2H), 8.01 (d, *J* = 8.4 Hz, 2H), 7.34 (s, 1H), 5.82 (d, *J* = 6.1 Hz, 1H), 5.46 (d, *J* = 6.1 Hz, 1H), 5.24 (d, *J* = 4.8 Hz, 1H), 4.66 (s, 1H), 3.96 (d, *J* = 27.7 Hz, 2H), 3.65 (d, *J* = 11.5 Hz, 2H), 3.36 (s, 4H), 2.44 (s, 2H), 2.18 (s, 1H), 1.76 (d, *J* = 12.9 Hz, 1H), 1.57 (d, *J* = 12.3 Hz, 4H). ^13^C NMR (75.5 MHz, *d*_6_-DMSO): *δ*/ppm = 173.7, 156.1, 152.5, 150.0, 141.6, 140.4, 129.0, 124.8, 87.8, 83.5, 72.6, 71.1, 45.2, 40.8, 40.1, 27.8. FT-IR: *ν*/cm^−1^ = 1641, 1569, 1526, 1477, 1346, 1311, 1254, 1165, 1092, 1056, 936, 857, 751, 682. *α*^20^_D_ = −36° (10 mg mL^−1^; DMSO). mp: 140–142 °C. ESI-MS: *m*/*z* calculated for C_22_H_26_N_8_O_8_S [M + H]^+^ = 563.16 (100%); found: 563.00 (100%). Purity: 99% (HPLC, 254 nm, MeCN/H_2_O = 10 : 90 to 100 : 0 + 0.1% HCOOH, *t*_R_ = 4.06 min).

#### *N*-(((2*R*,3*S*,4*R*,5*R*)-5-(6-Amino-9*H*-purin-9-yl)-3,4-dihydroxy-tetrahydrofuran-2-yl)methyl)-3-nitrobenzenesulfonamide (31)

The compound was prepared from 3 (50 mg, 0.16 mmol) according to the general procedure to afford the final product as a colorless trifluoroacetate salt (47 mg, 0.09 mmol, 64%, 0.9 equiv. TFA). ^1^H NMR (300 MHz, CD_3_OD): *δ*/ppm = 8.53 (t, *J* = 2.0 Hz, 1H), 8.37–8.29 (m, 3H), 8.18–8.11 (m, 1H), 7.72 (t, *J* = 8.0 Hz, 1H), 5.88 (d, *J* = 5.6 Hz, 1H), 4.67 (t, *J* = 5.5 Hz, 1H), 4.26 (dd, *J* = 5.4, 4.0 Hz, 1H), 4.10–4.04 (m, 1H), 3.30–3.25 (m, 2H). ^13^C NMR (75.5 MHz, CD_3_OD): *δ*/ppm = 153.7, 149.5, 148.0, 144.0, 143.8, 133.6, 131.9, 127.9, 122.8, 91.0, 85.2, 74.9, 72.5, 45.8. FT-IR: *ν*/cm^−1^ = 3103, 2359, 1671, 1532, 1507, 1429, 1352, 1164, 1129, 1070, 972, 881, 799, 723, 661. *α*^20^_D_ = +23° (10 mg mL^−1^; MeOH). mp: 100–102 °C. ESI-MS: *m*/*z* calculated for C_16_H_18_N_7_O_7_S [M + H]^+^ = 452.10 (100%); found: 451.95 (100%). Purity: 97% (HPLC, 254 nm, MeCN/H_2_O = 10 : 90 to 100 : 0 + 0.1% HCOOH, *t*_R_ = 6.56 min).

#### *N*-(((2*R*,3*S*,4*R*,5*R*)-5-(6-Amino-9*H*-purin-9-yl)-3,4-dihydroxy-tetrahydrofuran-2-yl)methyl)-4-chlorobenzenesulfonamide (32)

The compound was prepared from 3 (50 mg, 0.16 mmol) according to the general procedure to afford the final product as a colorless trifluoroacetate salt (80 mg, 0.14 mmol, 99%, 0.3 equiv. TFA). ^1^H NMR (300 MHz, CD_3_OD): *δ*/ppm = 8.39 (s, 1H) 8.34 (s, 1H), 7.79 (d, *J* = 7.0 Hz, 2H), 7.50 (d, *J* = 6.8 Hz, 2H), 5.95 (d, *J* = 5.7 Hz, 1H), 4.70 (t, *J* = 5.5 Hz, 1H), 4.27 (dd, *J* = 5.4, 3.7 Hz, 1H), 4.12 (q, *J* = 4.1 Hz, 1H), 3.27–3.21 (m, 2H). ^13^C NMR (75.5 MHz, CD_3_OD): *δ*/ppm = 152.1, 148.4, 146.1, 142.6, 139.2, 138.5, 129.0, 128.3, 119.6, 89.7, 84.0, 73.6, 71.2, 44.3. FT-IR: *ν*/cm^−1^ = 3100, 1676, 1477, 1427, 1397, 1326, 1200, 1159, 1135, 1085, 1014, 971, 829, 799, 754, 723. *α*^20^_D_ = +16° (10 mg mL^−1^; MeOH). mp: 87–89 °C. ESI-MS: *m*/*z* calculated for C_17_H_17_N_8_O_5_ [M + H]^+^ = 441.07 (100%) found: 41.07 (100%). Purity: 99% (HPLC, 254 nm, MeCN/H_2_O = 10 : 90 to 100 : 0 + 0.1% HCOOH, *t*_R_ = 5.21 min).

#### *N*-(((2*R*,3*S*,4*R*,5*R*)-5-(6-Amino-9*H*-purin-9-yl)-3,4-dihydroxy-tetrahydrofuran-2-yl)methyl)-4-nitrobenzenesulfonamide (33)

The compound was prepared from 3 (50 mg, 0.16 mmol) according to the general procedure to afford the final product as a yellow trifluoroacetate salt (79 mg, 0.14 mmol, 95%, 0.1 equiv. TFA). ^1^H NMR (300 MHz, CD_3_OD): *δ*/ppm = 8.13–8.03 (m, 4H), 7.85–7.76 (m, 2H), 5.68 (d, *J* = 5.7 Hz, 1H), 4.48 (t, *J* = 5.6 Hz, 1H), 4.03 (t, *J* = 4.3 Hz, 1H), 3.89 (q, *J* = 4.1 Hz, 1H), 3.18–3.09 (m, 2H). ^13^C NMR (75.5 MHz, CD_3_OD): *δ*/ppm = 153.0, 149.9, 148.4, 147.5, 146.3, 142.2, 128.0, 123.9, 119.5, 89.6, 83.9, 73.5, 71.2, 44.5. FT-IR: *ν*/cm^−1^ = 3105, 1675, 1607, 1528, 1478, 1427, 1349, 1309, 1200, 1161, 1132, 1012, 970, 902, 855, 831, 799, 737, 723, 685. *α*^20^_D_ = +18° (10 mg mL^−1^; MeOH). mp: 75–77 °C. ESI-MS: *m*/*z* calculated for C_17_H_17_N_8_O_5_ [M + H]^+^ = 452.10 (100%), found: 452.07 (100%). Purity: 99% (HPLC, 254 nm, MeCN/H_2_O = 10 : 90 to 100 : 0 + 0.1% HCOOH, *t*_R_ = 4.69 min).

#### *N*-(((2*R*,3*S*,4*R*,5*R*)-5-(6-Amino-9*H*-purin-9-yl)-3,4-dihydroxy-tetrahydrofuran-2-yl)methyl)naphthalene-2-sulfonamide (34)

The compound was prepared from 3 (50 mg, 0.16 mmol) according to the general procedure to afford the final product as a colorless trifluoroacetate salt (99 mg, 0.11 mmol, 79%, 3.9 equiv. TFA). ^1^H NMR (300 MHz, CD_3_OD): *δ*/ppm = 8.27–8.19 (m, 2H), 8.13 (s, 1H), 7.82–7.63 (m, 4H), 7.50–7.37 (m, 2H), 5.83 (d, *J* = 5.8 Hz, 1H), 4.66 (t, *J* = 5.6 Hz, 1H), 4.22 (dd, *J* = 5.5, 3.6 Hz, 1H), 4.09 (q, *J* = 4.0 Hz, 1H), 3.28–3.12 (m, 2H). ^13^C NMR (75.5 MHz, CD_3_OD): *δ*/ppm = 153.5, 149.4, 147.7, 143.8, 138.2, 135.9, 133.3, 130.4, 130.0, 129.8, 129.0, 128.8, 128.6, 123.2, 91.1, 85.4, 74.8, 72.5, 45.7. FT-IR: *ν*/cm^−1^ = 3360, 2487, 1671, 1433, 1323, 1186, 1130, 1073, 973, 839, 800, 723, 656. *α*^20^_D_ = +7° (10 mg mL^−1^; MeOH). mp: 94–96 °C. ESI-MS: *m*/*z* calculated for C_20_H_21_N_6_O_5_S [M + H]^+^ = 457.13 (100%); found: 457.29 (100%). Purity: 98% (HPLC, 254 nm, MeCN/H_2_O = 10 : 90 to 100 : 0 + 0.1% HCOOH, *t*_R_ = 7.91 min).

#### (*E*)-*N*-(((2*R*,3*S*,4*R*,5*R*)-5-(6-Amino-9*H*-purin-9-yl)-3,4-di-hydroxytetrahydrofuran-2-yl)methyl)-2-phenylethene-1-sulfonamide (35)

The compound was prepared from 3 (50 mg, 0.16 mmol) according to the general procedure to afford the final product as a colorless trifluoroacetate salt (89 mg, 0.07 mmol, 50%, 6.7 equiv. TFA). ^1^H NMR (300 MHz, CD_3_OD): *δ*/ppm = 8.27 (s, 1H), 8.16 (s, 1H), 7.35–7.15 (m, 5H), 6.81 (d, *J* = 15.5 Hz, 1H), 5.86 (d, *J* = 5.5 Hz, 1H), 4.58 (t, *J* = 5.5 Hz, 1H), 4.23 (dd, *J* = 5.4, 3.9 Hz, 1H), 4.07 (q, *J* = 4.3 Hz, 1H), 3.29–3.20 (m, 2H). ^13^C NMR (75.5 MHz, CD_3_OD): *δ*/ppm =163.2–162.8 (q, *J* = 35.8 Hz), 152.5, 149.6, 146.2, 144.2, 141.9, 134.0, 131.7, 130.0, 129.2, 126.8, 120.8, 91.0, 85.5, 75.2, 72.5, 45.5. FT-IR: *ν*/cm^−1^ = 3115, 2361, 1669, 1507, 1430, 1321, 1187, 1132, 973, 835, 800, 746, 723, 690. *α*^20^_D_ = +12° (10 mg mL^−1^; MeOH). mp: 108–110 °C. ESI-MS: *m*/*z* calculated for C_18_H_20_N_6_O_5_S [M + H]^+^ = 432.12 (100%); found: 433.00 (100%). Purity: 99% (HPLC, 254 nm, MeCN/H_2_O = 10 : 90 to 100 : 0 + 0.1% HCOOH, *t*_R_ = 3.06 min).

#### *N*-(((2*R*,3*S*,4*R*,5*R*)-5-(6-Amino-9*H*-purin-9-yl)-3,4-dihydroxy-tetrahydrofuran-2-yl)methyl)-3,5-bis(trifluoromethyl) benzenesulfonamide (36)

The compound was prepared from 3 (50 mg, 0.16 mmol) according to the general procedure to afford the final product as a colorless trifluoroacetate salt (36 mg, 0.07 mmol, 50%, 1.4 equiv. TFA). ^1^H NMR (300 MHz, DMSO-d_6_): *δ*/ppm = 8.62 (t, *J* = 5.9 Hz, 1H), 8.46–8.30 (m, 4H), 8.21 (s, 1H), 7.94 (s, 2H), 5.84 (d, *J* = 5.8 Hz, 1H), 4.60 (t, *J* = 5.5 Hz, 1H), 4.10 (dd, *J* = 5.2, 3.6 Hz, 1H), 3.99–3.91 (m, 1H), 3.27–3.20 (m, 2H). ^13^C NMR (75.5 MHz, DMSO-d_6_): *δ*/ppm = 154.5, 150.3, 148.8, 143.3, 140.8, 131.6, 131.1, 127.2, 124.4, 120.8, 87.9, 83.2, 72.8, 71.0, 44.8. FT-IR: *ν*/cm^−1^ = 3108, 2361, 1693, 1360, 1279, 1137, 905, 844, 724, 698, 682. *α*^20^_D_ = −2° (10 mg mL^−1^; DMSO). mp: 97–99 °C. ESI-MS: *m*/*z* calculated for C_18_H_16_F_6_N_6_O_5_S [M + H]^+^ = 543.09 (100%); found: 543.30 (100%). Purity: 95% (HPLC, 254 nm, MeCN/H_2_O = 10 : 90 to 100 : 0 + 0.1% HCOOH, *t*_R_ = 7.39 min).

#### *N*-(((2*R*,3*S*,4*R*,5*R*)-5-(6-Amino-9*H*-purin-9-yl)-3,4-dihydroxy-tetrahydrofuran-2-yl)methyl)-2-chloro-5-nitrobenzenesulfon-amide (37)

The compound was prepared from 3 (50 mg, 0.16 mmol) according to the general procedure to afford the final product as a colorless trifluoroacetate salt (49 mg, 0.08 mmol, 57%, 1.2 equiv. TFA). ^1^H NMR (300 MHz, CD_3_OD): *δ*/ppm = 8.50 (d, *J* = 2.7 Hz, 1H), 8.25 (d, *J* = 13.0 Hz, 2H), 8.11 (dd, *J* = 8.7, 2.8 Hz, 1H), 7.65 (d, *J* = 8.7 Hz, 1H), 5.72 (d, *J* = 5.2 Hz, 1H), 4.55 (t, *J* = 5.4 Hz, 1H), 4.15 (dd, *J* = 5.5, 4.4 Hz, 1H), 3.97 (q, *J* = 4.9 Hz, 1H), 3.33 (d, *J* = 5.1 Hz, 2H). ^13^C NMR (75.5 MHz, CD_3_OD): *δ*/ppm = 152.5, 149.4, 147.1, 146.3, 144.3, 141.2, 139.6, 134.1, 128.6, 126.4, 120.8, 91.0, 85.4, 75.0, 72.5, 46.3. FT-IR: *ν*/cm^−1^ = 3102, 1690, 1601, 1527, 1427, 1348, 1198, 1131, 1041, 888, 837, 799, 740, 723. *α*^20^_D_ = +24° (10 mg mL^−1^; MeOH). mp: 84–86 °C. ESI-MS: *m*/*z* calculated for C_16_H_16_ClN_7_O_7_S [M + H]^+^ = 486.06 (100%); found: 486.00 (100%). Purity: 97% (HPLC, 254 nm, MeCN/H_2_O = 10 : 90 to 100 : 0 + 0.1% HCOOH, *t*_R_ = 6.51 min).

#### *N*-(((2*R*,3*S*,4*R*,5*R*)-5-(6-Amino-9*H*-purin-9-yl)-3,4-dihydroxy-tetrahydrofuran-2-yl)methyl)naphthalene-1-sulfonamide (38)

The compound was prepared from 3 (50 mg, 0.16 mmol) according to the general procedure to afford the final product as a colorless trifluoroacetate salt (91 mg, 0.14 mmol, 99%, 1.9 equiv. TFA). ^1^H NMR (300 MHz, DMSO-d_6_): *δ*/ppm = 8.71–8.49 (m, 5H), 8.35 (s, 1H), 8.24–8.10 (m, 2H), 8.07–8.01 (m, 1H), 7.73–7.54 (m, 3H), 5.87 (d, *J* = 6.2 Hz, 1H), 4.64–4.62 (m, 1H), 4.08 (dd, *J* = 5.1, 3.0 Hz, 1H), 4.04–3.96 (m, 1H), 3.27–3.01 (m, 2H). ^13^C NMR (75.5 MHz, DMSO-d_6_): *δ*/ppm = 153.3, 148.7, 148.5, 141.9, 135.5, 133.9, 133.9, 129.0, 128.5, 127.9, 127.6, 127.0, 124.7, 124.6, 119.3, 88.0, 83.9, 72.9, 71.1, 44.8. FT-IR: *ν* / cm^−1^ = 3111, 2359, 1642, 1425, 1315, 1200, 1159, 1130, 799, 770, 721, 676. *α*^20^_D_ = −4° (10 mg mL^−1^; DMSO). mp: 83–85 °C. ESI-MS: *m*/*z* calculated for C_20_H_20_N_6_O_5_S [M + H]^+^ = 457.13 (100%); found: [M + H]+ = 457.00 (100%). Purity: 98% (HPLC, 254 nm, MeCN/H_2_O = 10 : 90 to 100 : 0 + 0.1% HCOOH, *t*_R_ = 4.1 min).

#### *N*-(((2*R*,3*S*,4*R*,5*R*)-5-(6-Amino-9*H*-purin-9-yl)-3,4-dihydroxy tetrahydrofuran-2-yl)methyl)-2,4,6-triisopropylbenzene sulfonamide (39)

The compound was prepared from 3 (50 mg, 0.16 mmol) according to the general procedure to afford the final product as a colorless trifluoroacetate salt (65 mg, 0.10 mmol,71%, 0.9 equiv. TFA). ^1^H NMR (300 MHz, CD_3_OD): *δ*/ppm = 8.26 (s, 1H), 8.15 (s, 1H), 7.05 (s, 2H), 5.80 (d, *J* = 5.8 Hz, 1H), 4.60 (t, *J* = 5.8 Hz, 1H), 4.24–4.11 (m, 1H), 4.08–3.87 (m, 3H), 3.17–3.01 (m, 2H), 2.83–2.64 (m, 1H), 1.21–0.93 (m, 18H). ^13^C NMR (75.5 MHz, CD_3_OD): *δ*/ppm = 154.3, 153.0, 151.8, 149.6, 146.6, 144.6, 133.5, 124.9, 91.5, 85.5, 74.7, 72.6, 45.1, 35.3, 30.6, 25.2, 25.2, 24.0, 23.9. FT-IR: *ν*/cm^−1^ = 2960, 2362, 1671, 1619, 1425, 1202, 1140, 1060, 974, 883, 836, 800, 723. *α*^20^_D_ = +23° (10 mg mL^−1^; MeOH). mp: 68–70 °C. ESI-MS: *m*/*z* calculated for C_25_H_37_N_6_O_5_S [M + H]^+^ = 533.25 (100%); found: 533.09 (100%). Purity: 98% (HPLC, 254 nm, MeCN/H_2_O = 10 : 90 to 100 : 0 + 0.1% HCOOH, *t*_R_ = 8.21 min).

#### *N*-(((2*R*,3*S*,4*R*,5*R*)-5-(6-Amino-9*H*-purin-9-yl)-3,4-dihydroxy-tetrahydrofuran-2-yl)methyl)-[1,1′-biphenyl]-4-sulfonamide (40)

The compound was prepared from 3 (50 mg, 0.16 mmol) according to the general procedure to afford the final product as a colorless trifluoroacetate salt (88 mg, 0.12 mmol, 86%, 2.2 equiv. TFA). ^1^H NMR (300 MHz, CD_3_OD): *δ*/ppm = 8.34–8.17 (m, 2H), 7.79–7.74 (m, 2H), 7.63–7.57 (m, 2H), 7.50–7.43 (m, 2H), 7.36–7.19 (m, 3H), 5.86 (d, *J* = 5.7 Hz, 1H), 4.61 (t, *J* = 5.6 Hz, 1H), 4.26–4.16 (m, 1H), 4.10–4.01 (m, 1H), 3.23–3.13 (m, 2H). ^13^C NMR (75.5 MHz, CD_3_OD): *δ*/ppm = 153.0, 146.8, 146.5, 144.1, 140.2, 140.1, 130.1, 129.5, 128.5, 128.5, 128.1, 91.0, 85.5, 75.0, 72.5, 45.7. FT-IR: *ν*/cm^−1^ = 3112, 2361, 1672, 1325, 1201, 1134, 839, 800, 763, 723, 695, 670. *α*^20^_D_ = +13° (10 mg mL^−1^; MeOH). mp:78–80 °C. ESI-MS: *m*/*z* calculated for C_22_H_22_N_6_O_5_S [M + H]^+^ = 483.15 (100%); found: 483.29 (100%). Purity: 96% (HPLC, 254 nm, MeCN/H_2_O = 10 : 90 to 100 : 0 + 0.1% HCOOH, *t*_R_ = 7.20 min).

#### *N*-(((2*R*,3*S*,4*R*,5*R*)-5-(6-Amino-9*H*-purin-9-yl)-3,4-dihydroxy tetrahydrofuran-2-yl)methyl)-5-methylthiophene-2-sulfon-amide (41)

The compound was prepared from 3 (50 mg, 0.16 mmol) according to the general procedure to afford the final product as a colorless trifluoroacetate salt (25 mg, 0.05 mmol, 42%, 0.5 equiv. TFA). ^1^H NMR (300 MHz, CD_3_OD): *δ*/ppm = 8.37 (s, 1H), 8.32 (s, 1H), 7.35 (d, *J* = 3.7 Hz, 1H), 6.76 (dd, *J* = 3.7, 1.2 Hz, 1H), 5.93 (d, *J* = 6.0 Hz, 1H), 4.70 (t, *J* = 5.7 Hz, 1H), 4.29–4.23 (m, 1H), 4.14 (q, *J* = 3.9 Hz, 1H), 3.25–3.21 (m, 2H), 2.46 (s, 3H). ^13^C NMR (75.5 MHz, CD_3_OD): *δ*/ppm = 153.6, 149.7, 149.0, 147.6, 144.1, 139.2, 133.5, 127.0, 91.2, 85.6, 74.9, 72.7, 45.9, 15.3. FT-IR: *ν*/cm^−1^ = 3104, 1689, 1507, 1438, 1326, 1199, 1144, 1078, 799, 723. *α*^20^_D_ = +15° (10 mg mL^−1^; MeOH). mp: 95–97 °C. ESI-MS: *m*/*z* calculated for C_15_H_19_N_6_O_5_S_2_ [M + H]^+^ = 427.09 (100%); found: 426.96 (100%). Purity: 97% (HPLC, 254 nm, MeCN/H_2_O = 10 : 90 to 100 : 0 + 0.1% HCOOH, *t*_R_ = 6.36 min).

#### *N*-(((2*R*,3*S*,4*R*,5*R*)-5-(6-Amino-9*H*-purin-9-yl)-3,4-dihydroxy-tetrahydrofuran-2-yl)methyl)-5-(dimethylamino)naphthalen-1-sulfonamide (42)

The compound was prepared from 3 (50 mg, 0.16 mmol) according to the general procedure to afford the final product as a yellow trifluoroacetate salt (118 mg, 0.09 mmol, 64%, 7.2 equiv. TFA). ^1^H NMR (300 MHz, CD_3_OD): *δ*/ppm = 8.40 – 8.30 (m, 2H), 8.19 (d, *J* = 21.4 Hz, 2H), 8.07 (dd, *J* = 7.4, 1.1 Hz, 1H), 7.49–7.38 (m, 2H), 7.32 (dd, *J* = 7.7, 1.1 Hz, 1H), 5.77 (d, *J* = 5.7 Hz, 1H), 4.57 (t, *J* = 5.7 Hz, 1H), 4.03 (dd, *J* = 5.4, 3.6 Hz, 1H), 3.96 (dt, *J* = 5.1, 3.6 Hz, 1H), 3.21–3.00 (m, 2H), 2.90 (s, 6H). ^13^C NMR (75.5 MHz, CD_3_OD): *δ*/ppm = 152.6, 149.0, 146.2, 144.4, 137.0, 130.7, 130.5, 129.8, 129.7, 129.0, 125.4, 122.8, 117.8, 117.7, 91.1, 85.5, 74.9, 72.5, 46.3, 45.6. FT-IR: *ν*/cm^−1^ = 3110, 2361, 1669, 1507, 1435, 1321, 1201, 1132, 1062, 839, 800, 724. *α*^20^_D_ = +13° (10 mg mL^−1^; MeOH). mp: 102–104 °C. ESI-MS: *m*/*z* calculated for C_22_H_26_N_7_O_5_S [M + H]^+^ = 500.17 (100%); found: 500.01 (100%). Purity: 99% (HPLC, 254 nm, MeCN/H_2_O = 10 : 90 to 100 : 0 + 0.1% HCOOH, *t*_R_ = 6.93 min).

#### *N*-(((2*R*,3*S*,4*R*,5*R*)-5-(6-Amino-9*H*-purin-9-yl)-3,4-dihydroxy tetrahydrofuran-2-yl)methyl)-4-bromo-3-nitrobenzenesulfon amide (44)

The compound was prepared from 3 (50 mg, 0.16 mmol) according to the general procedure to afford the final product as a yellow trifluoroacetate salt (115 mg, 0.14 mmol, 99%, 2.2 equiv. TFA). ^1^H NMR (300 MHz, DMSO-d_6_): *δ*/ppm = 8.70 (t, *J* = 6.0 Hz, 1H), 8.51–8.18 (m, 5H), 8.08 (d, *J* = 8.4 Hz, 1H), 7.93 (dd, J = 8.4, 2.1 Hz, 1H), 5.87 (d, J = 6.0 Hz, 1H), 4.63 (t, *J* = 5.6 Hz, 1H), 4.14–4.07 (m, 1H), 4.03–3.95 (m, 1H), 3.30–3.11 (m, 2H). ^13^C NMR (75.5 MHz, DMSO-d_6_): *δ*/ppm = 154.3, 149.8, 149.6, 141.4, 136.2, 131.2, 123.7, 118.0, 88.1, 83.6, 72.9, 71.1, 44.9. FT-IR: *ν*/cm^−1^ = 3094, 2360, 1641, 1590, 1537, 1426, 1331, 1168, 1128, 1032, 887, 828, 798, 768, 723, 662. *α*^20^_D_ = +3° (10 mg mL^−1^; DMSO). mp: 69–70 °C. ESI-MS: *m*/*z* calculated for C_16_H_16_BrN_7_O_7_S [M + H]^+^ = 532.01 (100%); found: 531.84 (100%). Purity: 97% (HPLC, 254 nm, MeCN/H_2_O = 10 : 90 to 100 : 0 + 0.1% HCOOH, *t*_R_ = 6.75 min).

#### *N*-(((2*R*,3*S*,4*R*,5*R*)-5-(6-Amino-9*H*-purin-9-yl)-3,4-dihydroxytetrahydrofuran-2-yl)methyl)-2-oxo-2H-chromene-8-sulfonamide (45)

The compound was prepared from 3 (50 mg, 0.16 mmol) according to the general procedure to afford the final product as a beige trifluoroacetate salt (54 mg, 0.08 mmol, 57%, 1.8 equiv. TFA). ^1^H NMR (300 MHz, DMSO-d_6_): *δ*/ppm = 8.62 (t, *J* = 5.9 Hz, 1H), 8.31 (s, 1H), 8.24–8.11 (m, 3H), 7.95 (dd, *J* = 8.7, 2.3 Hz, 1H), 7.57–7.49 (m, 2H), 6.60 (d, *J* = 9.6 Hz, 1H), 5.83 (d, *J* = 6.4 Hz, 1H), 4.66 (t, *J* = 5.8 Hz, 1H), 4.10–3.96 (m, 2H), 3.19–3.11 (m, 2H). ^13^C NMR (75.5 MHz, DMSO-d_6_): *δ*/ppm = 159.4, 156.0, 155.9, 155.6, 152.0, 148.8, 143.6, 140.7, 136.5, 136.4, 129.6, 127.4, 119.5, 119.0, 117.7, 88.2, 83.6, 72.5, 71.2, 45.0. FT-IR: *ν*/cm^−1^ = 3435, 2361, 1674, 1444, 1196, 1134, 844, 803, 725. *α*^20^_D_ = +8° (10 mg mL^−1^; DMSO). mp: 163–165 °C. ESI-MS: *m*/*z* calculated for C_19_H_19_N_6_O_7_S [M + H]^+^ = 475.10 (100%); found: 474.94 (100%). Purity: 97% (HPLC, 254 nm, MeCN/H_2_O = 10 : 90 to 100 : 0 + 0.1% HCOOH, *t*_R_ = 6.01 min).

#### *N*-(((2*R*,3*S*,4*R*,5*R*)-5-(6-Amino-9*H*-purin-9-yl)-3,4-dihydroxy-tetrahydrofuran-2-yl)methyl)-4-chloro-3-(trifluoromethyl) benzenesulfonamide (46)

The compound was prepared from 3 (50 mg, 0.16 mmol) according to the general procedure to afford the final product as a colorless trifluoroacetate salt (91 mg, 0.08 mmol, 57%, 5.4 equiv. TFA). ^1^H NMR (300 MHz, CD_3_OD): *δ*/ppm = 8.36–8.21 (m, 2H), 8.03 (d, *J* = 2.2 Hz, 1H), 7.92 (dd, *J* = 8.4, 2.2 Hz, 1H), 7.63 (d, *J* = 8.4 Hz, 1H), 5.87 (d, *J* = 5.4 Hz, 1H), 4.60 (t, *J* = 5.4 Hz, 1H), 4.22 (dd, *J* = 5.4, 4.3 Hz, 1H), 4.02 (q, *J* = 4.3 Hz, 1H), 3.25–3.15 (m, 2H). ^13^C NMR (75.5 MHz, CD_3_OD): *δ*/ppm = 153.0, 149.7, 146.7, 144.0, 141.5, 137.8, 133.8, 132.9, 130.5–129.2 (q), 127.4–127.3 (q), 125.4, 121.7, 90.9, 85.1, 75.1, 72.4, 45.6. FT-IR: *ν*/cm^−1^ = 3105, 2361, 1670, 1431, 1311, 1131, 1037, 839, 800, 724. *α*^20^_D_ = +22° (10 mg mL^−1^; MeOH). mp: 109–111 °C. ESI-MS: *m*/*z* calculated for C_17_H_17_ClF_3_N_6_O_5_S [M + H]^+^ = 509.06 (100%); found: 508.97 (100%). Purity: 96% (HPLC, 254 nm, MeCN/H_2_O = 10 : 90 to 100 : 0 + 0.1% HCOOH, *t*_R_ = 6.04 min).

#### *N*-(((2*R*,3*S*,4*R*,5*R*)-5-(6-Amino-9*H*-purin-9-yl)-3,4-dihydroxy-tetrahydrofuran-2-yl)methyl)-4-methyl-3-nitrobenzenesulfon amide (47)

The compound was prepared from 3 (50 mg, 0.16 mmol) according to the general procedure to afford the final product as a colorless trifluoroacetate salt (86 mg, 0.12 mmol, 86%, 2.3 equiv. TFA). ^1^H NMR (300 MHz, DMSO-d_6_): *δ*/ppm = 8.37–8.29 (m, 2H), 8.26–8.22 (m, 1H), 8.16–8.06 (m, 1H), 7.84–7.71 (m, 2H), 7.62–7.50 (m, 2H), 5.87 (d, *J* = 6.6 Hz, 1H), 4.68–4.55 (m, 3H), 4.18–4.08 (m, 2H), 3.48–3.23 (m, 2H), 2.07 (s, 3H). ^13^C NMR (75.5 MHz, DMSO-d_6_): *δ*/ppm =. FT-IR: *ν*/cm^−1^ = 159.0, 158.6, 155.9, 151.6, 147.6, 141.0, 137.8, 132.8, 132.8, 129.9, 125.5, 123.0, 88.8, 84.2, 72.6, 71.1, 55.8, 45.0, 30.8. *α*^20^_D_ = −12° (10 mg mL^−1^; DMSO). mp: 102–104 °C. ESI-MS: *m*/*z* calculated for C_17_H_20_N_7_O_7_S [M + H]^+^ = 466.11 (100%); found: 465.98 (100%). Purity: 95% (HPLC, 254 nm, MeCN/H_2_O = 10 : 90 to 100 : 0 + 0.1% HCOOH, *t*_R_ = 6.33 min).

#### *N*-(((2*R*,3*S*,4*R*,5*R*)-5-(6-Amino-9*H*-purin-9-yl)-3,4-dihydroxy tetrahydrofuran-2-yl)methyl)-2,4-dichlorobenzenesulfon amide (48)

The compound was prepared from 3 (50 mg, 0.16 mmol) according to the general procedure to afford the final product as a color trifluoroacetate salt (68 mg, 0.14 mmol, 99%, 0.2 equiv. TFA). ^1^H NMR (300 MHz, CD_3_OD): *δ*/ppm = 8.34–8.24 (m, 2H), 7.85 (d, *J* = 8.5 Hz, 1H), 7.48 (d, *J* = 2.1 Hz, 1H), 7.27 (dd, *J* = 8.5, 2.1 Hz, 1H), 5.82 (d, *J* = 5.9 Hz, 1H), 4.63 (t, *J* = 5.7 Hz, 1H), 4.16 (dd, *J* = 5.5, 3.7 Hz, 1H), 4.06–4.00 (m, 1H), 3.24–3.17 (m, 3H). ^13^C NMR (75.5 MHz, CD_3_OD): *δ*/ppm = 153.6, 150.3, 147.3, 145.0, 140.8, 138.6, 134.6, 133.9, 133.0, 129.2, 91.7, 86.2, 75.6, 73.3, 46.5. FT-IR: *ν*/cm^−1^ = 3108, 2360, 1682, 1331, 1200, 1140, 1103, 1065, 1040, 825, 724. *α*^20^_D_ = +7° (10 mg mL^−1^; DMSO). mp: 94–96 °C. ESI-MS: *m*/*z* calculated for C_16_H_17_Cl_2_N_6_O_5_S [M + H]^+^ = 475.04 (100%); found: 474.67 (100%). Purity: 97% (HPLC, 254 nm, MeCN/H_2_O = 10 : 90 to 100 : 0 + 0.1% HCOOH, *t*_R_ = 6.7 min).

#### *N*-(((2*R*,3*S*,4*R*,5*R*)-5-(6-Amino-9*H*-purin-9-yl)-3,4-dihydroxy tetrahydrofuran-2-yl)methyl)-6-chloropyridine-3-sulfonamide (49)

The compound was prepared from 3 (50 mg, 0.16 mmol) according to the general procedure to afford the final product as a colorless trifluoroacetate salt (94 mg, 0.13 mmol, 93%, 2.7 equiv. TFA). ^1^H NMR (300 MHz, CD_3_OD): *δ*/ppm = 8.65–8.61 (m, 1H), 8.28 (s, 2H), 8.06 (dd, *J* = 8.4, 2.6 Hz, 1H), 7.44 (dd, *J* = 8.4, 0.7 Hz, 1H), 5.87 (d, *J* = 5.5 Hz, 1H), 4.64 (t, *J* = 5.5 Hz, 1H), 4.24 (dd, *J* = 5.5, 4.1 Hz, 1H), 4.05 (q, *J* = 4.5 Hz, 1H), 3.27 (d, *J* = 4.5 Hz, 2H). ^13^C NMR (75.5 MHz, CD_3_OD): *δ*/ppm = 155.8, 153.3, 149.7, 149.1, 147.3, 143.9, 139.0, 137.9, 126.0, 120.8, 91.0, 85.1, 74.9, 72.4, 45.7. FT-IR: *ν*/cm^−1^ = 3106, 2360, 1671, 1572, 1449, 1333, 1202, 1132, 838, 801, 776, 724. *α*^20^_D_ = +15° (10 mg mL^−1^; MeOH). mp: 83–85 °C. ESI-MS: *m*/*z* calculated for C_15_H_17_ClN_7_O_5_S [M + H]^+^ = 442.07 (100%); found: 441.94 (100%). Purity: 96% (HPLC, 254 nm, MeCN/H_2_O = 10 : 90 to 100 : 0 + 0.1% HCOOH, *t*_R_ = 6.39 min)

#### *N*-(((2*R*,3*S*,4*R*,5*R*)-5-(6-Amino-9*H*-purin-9-yl)-3,4-dihydroxytetra hydrofuran-2-yl)methyl)piperidine-4-sulfonamide (50)

The compound was prepared from 3 (50 mg, 0.16 mmol) according to the general procedure to afford the final product as a grey trifluoroacetate salt (23 mg, 0.06 mmol, 43%, 0.0 equiv. TFA). ^1^H NMR (400 MHz, DMSO-d_6_): *δ*/ppm = 8.89–8.82 (m, 1H), 8.48–8.38 (m, 2H), 8.12 (s, 1H), 7.90 (s, 2H), 5.88 (d, *J* = 6.4 Hz, 1H), 4.71 (t, *J* = 5.9 Hz, 1H), 4.16–4.12 (m, 1H), 4.06–4.03 (m, 1H), 3.41–3.20 (m, 6H), 2.84 (d, *J* = 12.0 Hz, 3H), 2.16–1.99 (m, 3H), 1.85–1.66 (m, 3H). ^13^C NMR (101 MHz, DMSO-d_6_): *δ*/ppm = 155.0, 150.6, 150.6, 141.3, 88.2, 84.1, 72.5, 71.1, 54.3, 52.6, 44.9, 42.5, 42.0, 40.4, 40.2, 24.4, 22.8. FT-IR: *ν*/cm^−1^ = 3097, 1674, 1428, 1320, 1200, 1133, 836, 799, 723. *α*^20^_D_ = −14° (10 mg mL^−1^; MeOH). mp: 81–83 °C. ESI-MS: *m*/*z* calculated for C_15_H_24_N_7_O_5_S [M + H]^+^ = 414.16 (100%); found: [M + 2H]^2+^ = 208.00. Purity: 99% (HPLC, 254 nm, MeCN/H_2_O = 10 : 90 to 100 : 0 + 0.1% HCOOH, *t*_R_ = 1.29 min).

#### *N*-(((2*R*,3*S*,4*R*,5*R*)-5-(6-Amino-9*H*-purin-9-yl)-3,4-dihydroxy-tetrahydrofuran-2-yl)methyl)-4-(methylsulfonyl)benzene-sulfonamide (51)

The compound was prepared from 3 (50 mg, 0.16 mmol) according to the general procedure to afford the final product as a colorless trifluoroacetate salt. (85 mg, 0.11 mmol, 79%, 2.3 equiv. TFA). ^1^H NMR (300 MHz, CD_3_OD): *δ*/ppm = 6.77–6.68 (m, 1H), 6.37 (s, 4H), 4.29 (d, *J* = 5.5 Hz,1H), 3.03 (t, *J* = 5.4 Hz, 1H), 2.63 (dd, *J* = 5.4, 4.0 Hz, 1H), 2.46 (q, *J* = 4.3 Hz, 1H), 1.69–1.58 (m, 2H), 1.49 (s, 3H). ^13^C NMR (75.5 MHz, CD_3_OD): *δ*/ppm = 152.7, 149.7, 146.8, 146.4, 145.4, 144.1, 129.4, 129.0, 90.8, 85.2, 75.1, 72.4, 45.7, 44.0. FT-IR: *ν*/cm^−1^ = 3311, 1673, 1429, 1312, 1199, 1133, 962, 837, 800, 781, 723. *α*^20^_D_ = +15° (10 mg mL^−1^; ACN). mp: 84–86 °C. ESI-MS: *m*/*z* calculated for C_17_H_20_N_6_O_7_S_2_ [M + H]^+^ = 485.08 (100%); found: 485.00 (100%). Purity: 100% (HPLC, 254 nm, MeCN/H_2_O = 10 : 90 to 100 : 0 + 0.1% HCOOH, *t*_R_ = 2.87 min).

#### *N*-(((2*R*,3*S*,4*R*,5*R*)-5-(6-Amino-9*H*-purin-9-yl)-3,4-dihydroxy-tetrahydrofuran-2-yl)methyl)-2-methoxyethane-1-sulfon-amide (52)

The compound was prepared from 3 (50 mg, 0.16 mmol) according to the general procedure to afford the final product as a beige trifluoroacetate salt (50 mg, 0.10 mmol, 71%, 1.1 equiv. TFA). ^1^H NMR (300 MHz, CD_3_OD): *δ*/ppm = 6.84 (s, 1H), 6.78–6.72 (m, 1H), 4.37 (d, *J* = 5.9 Hz, 1H), 3.11 (t, *J* = 5.6 Hz, 1H), 2.71 (dd, *J* = 5.3, 3.5 Hz, 1H), 2.56 (q, *J* = 3.9 Hz, 1H), 2.19–2.01 (m, 2H), 1.87–1.66 (m, 4H), 1.63 (s, 3H). ^13^C NMR (75.5 MHz, CD_3_OD): *δ*/ppm = 152.8, 149.8, 146.5, 144.4, 91.0, 85.9, 75.1, 72.5, 67.7, 59.0, 52.6, 45.6. FT-IR: *ν*/cm^−1^ = 3118, 1678, 1427, 1323, 1199, 1135, 832, 800, 723. *α*^20^_D_ = −11° (10 mg mL−1; ACN). mp: 48–50 °C. ESI-MS: *m*/*z* calculated for C_13_H_20_N_6_O_6_S [M + H]^+^ = 389.12 (100%); found: 389.00 (100%). Purity: 99% (HPLC, 254 nm, MeCN/H_2_O = 10 : 90 to 100 : 0 + 0.1% HCOOH, *t*_R_ = 1.78 min).

#### *N*-(((2*R*,3*S*,4*R*,5*R*)-5-(6-Amino-9*H*-purin-9-yl)-3,4-dihydroxy-tetrahydrofuran-2-yl)methyl)-2-nitro-4-((trifluoromethyl)sulfonyl)benzenesulfonamide (53)

The compound was prepared from 3 (50 mg, 0.16 mmol) according to the general procedure to afford the final product as a yellow trifluoroacetate salt. (21 mg, 0.03 mmol, 21%, 1.4 equiv. TFA). ^1^H NMR (300 MHz, CD_3_OD): *δ*/ppm = 8.59–8.48 (m, 1H), 8.40–8.20 (m, 4H), 5.86 (d, *J* = 5.5 Hz, 1H), 4.62 (t, *J* = 5.5 Hz, 1H), 4.24 (dd, *J* = 5.5, 4.1 Hz, 1H), 4.09 (dt, *J* = 5.6, 3.9 Hz, 1H), 3.58–3.37 (m, 2H). ^13^C NMR (75.5 MHz, CD_3_OD): *δ*/ppm =152.4, 149.7, 146.2, 144.4, 142.6, 136.9, 135.5, 133.9, 128.0, 91.0, 85.3, 75.1, 72.4, 46.2. FT-IR: *ν*/cm^−1^ = 3102, 1696, 1552, 1509, 1378, 1201, 1169, 1138, 1086, 836, 725. *α*^20^_D_ = +32° (10 mg mL^−1^; MeOH). mp: 140–142 °C. ESI-MS: *m*/*z* calculated for C_17_H_16_F_3_N_7_O_9_S_2_ [M + H]^+^ = 584.14 (100%); found: 584.00 (100%). Purity: 95% (HPLC, 254 nm, MeCN/H_2_O = 10 : 90 to 100 : 0 + 0.1% HCOOH, *t*_R_ = 4.82 min).

#### *N*-(((2*R*,3*S*,4*R*,5*R*)-5-(6-Amino-9*H*-purin-9-yl)-3,4-dihydroxy-tetrahydrofuran-2-yl)methyl)-*N*4,*N*4-dimethyl-2-nitrobenzene-1,4-disulfonamide (54)

The compound was prepared from 3 (50 mg, 0.16 mmol) according to the general procedure to afford the final product as a colorless trifluoroacetate salt. (49 mg, 0.09 mmol, 63%, 0.73 equiv. TFA). ^1^H NMR (300 MHz, CD_3_OD): *δ*/ppm = 8.32–8.15 (m, 4H), 8.04 (dd, *J* = 8.2, 1.8 Hz, 1H), 5.85 (d, *J* = 6.4 Hz, 1H), 4.79 (dd, *J* = 6.3, 5.4 Hz, 1H), 4.24 (dd, *J* = 5.4, 2.9 Hz, 1H), 4.19 (q, *J* = 3.5 Hz, 1H), 3.60–3.36 (m, 2H), 2.74 (s, 6H). ^13^C NMR (75.5 MHz, CD_3_OD): *δ*/ppm = 149.5, 142.9, 142.4, 138.3, 133.0, 132.1, 124.8, 91.4, 85.6, 74.4, 72.8, 46.2, 38.1. FT-IR: *ν*/cm^−1^ = 3088, 1694, 1548, 1428, 1350, 1171, 1101, 958, 831, 799, 782, 712. *α*^20^_D_ = +33° (10 mg mL^−1^; MeOH). mp: 86–88 °C. ESI-MS: *m*/*z* calculated for C_18_H_22_N_8_O_9_S_2_ [M + H]^+^ = 559.10 (100%); found: 559.00 (100%). Purity: 96% (HPLC, 254 nm, MeCN/H_2_O = 10 : 90 to 100 : 0 + 0.1% HCOOH, *t*_R_ = 3.97 min).

#### 9-((3a*R*,4*R*,6*R*,6a*R*)-6-((Isopropylamino)methyl)-2,2-dimethyl-tetrahydrofuro[3,4-*d*][1,3]dioxol-4-yl)-9*H*-purin-6-amine (55)

3 (200 mg, 0.65 mmol, 1.0 equiv.) was dissolved in MeOH (4 mL) at 0 °C and acetone (96 μL, 1.31 mmol, 2.0 equiv.) and acetic acid (75 μL, 1.31 mmol, 2.0 equiv.) were added. After stirring for 30 min at 0 °C NaBH3CN (692 mg, 3.27 mmol, 5 equiv.) was added and the reaction mixture was stirred over night at rt. The solvent was removed by reduced pressure and the residue was purified by flash column chromatography (grd, 0 : 100 to 100 : 0 = ACN : H_2_O) to afford the secondary amine 55 as colorless solid (164 mg, 47 mmol, 72%). ^1^H NMR (300 MHz, CDCl_3_): *δ*/ppm = 8.28 (s, 1H), 8.14 (s, 1H), 6.35–6.29 (m, 1H), 5.37–5.30 (m, 1H), 5.07 (dd, *J* = 6.5, 3.0 Hz, 1H), 4.77 (d, *J* = 10.0 Hz, 1H), 4.14 (s, 1H), 3.71–3.59 (m, 1H), 3.36 (d, *J* = 12.1 Hz, 1H), 1.58 (s, 3H), 1.45–1.32 (m, 8H). ^13^C NMR (75.5 MHz, CDCl_3_): *δ*/ppm = 155.3, 152.9, 148.2, 118.5, 115.7, 84.3, 82.4, 81.3, 50.6, 46.2, 27.1, 25.4, 19.6, 19.3. FT-IR: *ν*/cm^−1^ = 2986, 2339, 2168, 1643, 1598, 1477, 1377, 1330, 1211, 1154, 1080, 865, 797. *α*^20^_D_ = −5° (10 mg mL^−1^; CHCl_3_).

#### Morpholino(thiophen-2-yl)methanone (58)

The compound was prepared from morpholine (13.5 mg, 0.16 mmol) according to the general procedure to afford the final product as a colorless oil. (25 mg, 0.13 mmol, 93%). ^1^H NMR (300 MHz, CD_3_OD): *δ*/ppm = 7.44 (dd, *J* = 5.0, 1.2 Hz, 1H), 7.27 (dd, *J* = 3.7, 1.2 Hz, 1H), 7.08–6.98 (m, 1H), 3.81–3.65 (m, 8H). ^13^C NMR (75.5 MHz, CD_3_OD): *δ*/ppm = 174.0, 146.9, 139.3, 139.2, 137.1, 77.2. FT-IR: *ν*/cm^−1^ = 2939, 1603, 1521, 1433, 1292, 1264, 1136, 1048, 999, 834, 735. ESI-MS: *m*/*z* calculated for C_9_H_11_NO_2_S [M + H]+ = 198.05 (100%); found: 198.00 (100%). Purity: 99% (HPLC, 254 nm, MeCN/H_2_O = 10 : 90 to 100 : 0 + 0.1% HCOOH, *t*_R_ = 4.09 min).

#### 4-((4-Chlorophenyl)sulfonyl)morpholine (59)

The compound was prepared from morpholine (13.5 mg, 0.16 mmol) according to the general procedure to afford the final product as a colorless solid (37 mg, 0.14 mmol, 99%). ^1^H NMR (300 MHz, CDCl_3_): *δ*/ppm = 7.74–7.62 (m, 1H), 7.57–7.46 (m, 1H), 3.78–3.68 (m, 2H), 3.03–2.94 (m, 2H). ^13^C NMR (75.5 MHz, CDCl_3_): *δ*/ppm = 139.8, 133.7, 129.6, 129.3, 66.1, 46.0. FT-IR: *ν*/cm^−1^ = 3331, 1639, 1328, 1260, 1158, 1070, 1012, 945, 829, 761, 721. mp: 90–92 °C. ESI-MS: *m*/*z* calculated for C_10_H_12_ClNO_3_S [M + H]+ = 261.02 (100%). Purity: 99% (HPLC, 254 nm, MeCN/H_2_O = 10 : 90 to 100 : 0 + 0.1% HCOOH, *t*_R_ = 6.23 min).

#### (4-Methylpiperazin-1-yl)(thiophen-2-yl)methanone (60)

The compound was prepared from *N*-methylpiperazine (15.5 mg, 0.16 mmol) according to the general procedure to afford the final product as a colorless oil. (25 mg, 0.12 mmol, 86%). ^1^H NMR (300 MHz, CDCl_3_): *δ*/ppm = 7.42 (dd, *J* = 5.1, 1.1 Hz, 1H), 7.26 (dd, *J* = 3.6, 1.2 Hz, 1H), 7.02 (dd, *J* = 5.0, 3.6 Hz, 1H), 3.75 (t, *J* = 5.1 Hz, 4H), 2.44 (t, *J* = 5.0 Hz, 4H), 2.31 (s, 3H). ^13^C NMR (75.5 MHz, CDCl_3_): *δ*/ppm = 163.6, 137.0, 128.9, 128.7, 126.8, 55.0, 46.0. FT-IR: *ν*/cm^−1^ = 3336, 1606, 1522, 1431, 1321, 1253, 1153, 1114, 1001, 823, 736. ESI-MS: *m*/*z* calculated for C_10_H_14_N_2_OS [M + H]+ = 211.09 (100%); found: 211.00 (100%). Purity: 97% (HPLC, 254 nm, MeCN/H_2_O = 10 : 90 to 100 : 0 + 0.1% HCOOH, *t*_R_ = 1.38 min).

#### 1-((4-Chlorophenyl)sulfonyl)-4-methylpiperazine (61)

The compound was prepared from *N*-methylpiperazine (15.5 mg, 0.16 mmol) according to the general procedure to afford the final product as a colorless solid. (30 mg, 0.11 mmol, 79%). ^1^H NMR (300 MHz, CDCl_3_): *δ*/ppm = 7.73–7.61 (m, 1H), 7.56–7.43 (m, 1H), 3.01 (t, *J* = 5.0 Hz, 2H), 2.46 (t, *J* = 5.0 Hz, 2H), 2.25 (s, 1H). ^13^C NMR (75.5 MHz, CDCl_3_): *δ*/ppm = 139.5, 133.9, 129.4, 129.3, 54.0, 46.0, 45.7. FT-IR: *ν*/cm^−1^ = 2799, 1586, 1453, 1351, 1332, 1289, 1169, 1151, 1095, 1013, 937, 831, 786, 761, 723. mp: 106–108 °C. ESI-MS: *m*/*z* calculated for C_11_H_15_ClN_2_O_2_S [M + H]+ = 275.06 (100%); found: 275.00 (100%). Purity: 97% (HPLC, 254 nm, MeCN/H_2_O = 10 : 90 to 100 : 0 + 0.1% HCOOH, *t*_R_ = 3.23 min).

#### *N*-(((2*R*,3*S*,4*R*,5*R*)-5-(6-Amino-9*H*-purin-9-yl)-3,4-dihydroxytetra hydrofuran-2-yl)methyl)-2-(2-bromo-3-fluorophenyl)acetamide (63)

The compound was prepared from 3 (50 mg, 0.16 mmol) according to the general procedure to afford the final product as a beige trifluoroacetate salt (86 mg, 0.14 mmol, 99%, 1.1 equiv. TFA). ^1^H NMR (300 MHz, DMSO-d_6_): *δ*/ppm = 8.94 (s, 2H), 8.64 (s, 1H), 8.55–8.39 (m, 2H), 7.41–7.13 (m, 3H), 5.96 (d, *J* = 6.0 Hz, 1H), 4.71 (t, *J* = 5.6 Hz, 1H), 4.18–4.00 (m, 2H), 3.72 (s, 2H), 3.56–3.40 (m, 2H). ^13^C NMR (75.5 MHz, DMSO-d_6_): *δ*/ppm = 169.0, 156.9, 152.5, 148.8, 147.6, 142.3, 138.7, 128.8, 127.6, 119.3, 114.9, 111.4, 88.0, 83.9, 73.4, 71.3, 42.0, 41.3. FT-IR: *ν*/cm^−1^ = 3086, 2360, 1669, 1445, 1199, 1130, 1038, 798, 773, 720. *α*^20^_D_ = −25° (10 mg mL^−1^; DMSO). mp: 67–69 °C. ESI-MS: *m*/*z* calculated for C_18_H_18_BrFN_6_O_4_ [M + H]^+^ = 481.06 (100%); found: [M + H]^+^ = 481.00 (100%). Purity: 96% (HPLC, 254 nm, MeCN/H_2_O = 10 : 90 to 100 : 0 + 0.1% HCOOH, *t*_R_ = 3.96 min).

#### *N*-(((2*R*,3*S*,4*R*,5*R*)-5-(6-Amino-9*H*-purin-9-yl)-3,4-dihydroxytetra hydrofuran-2-yl)methyl)-5-(*m*-tolyl)nicotinamide (64)

The compound was prepared from 3 (50 mg, 0.16 mmol) according to the general procedure to afford the final product as a beige trifluoroacetate salt (60 mg, 0.07 mmol, 50%, 3.2 equiv. TFA). ^1^H NMR (300 MHz, CD_3_OD): *δ*/ppm = 9.08–8.98 (m, 3H), 8.70 (s, 1H), 8.54–8.45 (m, 1H), 8.40 (s, 1H), 7.65–7.53 (m, 2H), 7.41 (t, *J* = 7.6 Hz, 1H), 7.32–7.22 (m, 1H), 5.96 (d, *J* = 5.6 Hz, 1H), 4.71 (t, *J* = 5.3 Hz, 1H), 4.25 (dd, *J* = 5.0, 3.8 Hz, 1H), 4.20–4.09 (m, 1H), 3.81–3.56 (m, 2H), 2.39 (s, 3H). ^13^C NMR (75.5 MHz, CD_3_OD): *δ*/ppm = 164.9, 159.6, 159.1, 158.7, 158.2, 151.7, 149.3, 148.6, 146.9, 146.8, 142.3, 138.6, 136.1, 133.2, 129.3, 129.2, 127.7, 124.2, 119.1, 87.9, 83.4, 73.3, 71.3, 41.7, 21.1. FT-IR: *ν*/cm^−1^ = 3076, 2359, 2342, 1670, 1550, 1425, 1327, 1199, 1131, 831, 797, 721. *α*^20^_D_ = −18° (10 mg mL^−1^; DMSO). mp: 63–65 °C. ESI-MS: *m*/*z* calculated for C_23_H_24_N_7_O_4_ [M + H]^+^ = 462.19 (100%); found: [M+H]^+^ = 462.00 (81.2%). Purity: 99% (HPLC, 254 nm, MeCN/H_2_O = 10 : 90 to 100 : 0 + 0.1% HCOOH, *t*_R_ = 4.23 min).

#### *N*-(((2*R*,3*S*,4*R*,5*R*)-5-(6-Amino-9*H*-purin-9-yl)-3,4-dihydroxytetra hydrofuran-2-yl)methyl)-1-(naphthalen-2-yl)methanesulfon amide (65)

The compound was prepared from 3 (50 mg, 0.16 mmol) according to the general procedure to afford the final product as a colorless trifluoroacetate salt (36 mg, 0.06 mmol, 43%, 1.3 equiv. TFA). ^1^H NMR (300 MHz, DMSO-d_6_): *δ*/ppm = 8.47 (s, 1H), 8.16–7.75 (m, 8H), 7.56–7.41 (m, 3H), 5.89 (d, *J* = 6.5 Hz, 1H), 4.68–4.46 (m, 3H), 4.14 (dd, *J* = 5.1, 2.7 Hz, 1H), 4.09–4.02 (m, 1H), 3.46–3.19 (m, 2H). ^13^C NMR (75.5 MHz, DMSO-d_6_): *δ*/ppm = 151.5, 139.8, 132.6, 132.4, 129.9, 128.5, 127.9, 127.6, 126.3, 119.3, 112.2, 88.2, 84.2, 72.7, 71.0, 57.1, 44.9. FT-IR: *ν*/cm^−1^ = 3102, 2359, 1676, 1508, 1428, 1318, 1200, 1127, 825, 799, 722. *α*^20^_D_ = n.d. (10 mg mL^−1^; MeOH). mp: 70–72 °C. ESI-MS: *m*/*z* calculated for C_21_H_22_N_6_O_5_S [M + H]^+^ = 471.15 (100%); found: [M+H]^+^ = 471.00 (100%). Purity: 95% (HPLC, 254 nm, MeCN/H_2_O = 10 : 90 to 100 : 0 + 0.1% HCOOH, *t*_R_ = 3.79 min).

#### *N*-(((2*R*,3*S*,4*R*,5*R*)-5-(6-Amino-9*H*-purin-9-yl)-3,4-dihydroxytetra hydrofuran-2-yl)methyl)-1-phenylmethanesulfonamide (66)

The compound was prepared from 3 (50 mg, 0.16 mmol) according to the general procedure to afford the final product as a colorless trifluoroacetate salt (65 mg, 0.14 mmol, 99%, 0.2 equiv. TFA). ^1^H NMR (300 MHz, DMSO-d_6_): *δ*/ppm = 8.48 (s, 1H), 8.22 (s, 2H), 8.01–7.91 (m, 2H), 7.41–7.25 (m, 5H), 5.88 (d, *J* = 6.7 Hz, 1H), 4.64 (dd, *J* = 6.7, 5.0 Hz, 1H), 4.44–4.28 (m, 2H), 4.12 (dd, *J* = 5.1, 2.5 Hz, 1H), 4.09–3.97 (m, 1H), 3.38–3.14 (m, 2H). ^13^C NMR (75.5 MHz, DMSO-d_6_): *δ*/ppm = 153.8, 149.2, 148.4, 141.5, 130.9, 130.4, 128.3, 128.0, 119.4, 88.2, 84.3, 72.8, 71.1, 57.2, 44.9. FT-IR: *ν*/cm^−1^ = 3114, 2360, 1686, 1425, 1318, 1200, 1124, 828, 798 721, 699. *α*^20^_D_ = −27° (10 mg mL^−1^; DMSO). mp: 90–92 °C. ESI-MS: *m*/*z* calculated for C_17_H_20_N_6_O_5_S [M + H]^+^ = 421.13 (100%); found: [M + H]^+^ = 421.00 (100%). Purity: 99% (HPLC, 254 nm, MeCN/H_2_O = 10 : 90 to 100 : 0 + 0.1% HCOOH, *t*_R_ = 3.79 min).

#### 4-((((2*R*,3*S*,4*R*,5*R*)-5-(6-Amino-9*H*-purin-9-yl)-3,4-dihydroxy-tetrahydrofuran-2-yl)methyl)carbamoyl)bicyclo[2.2.2]octane-1-carboxylic acid (67)

(5 mg, 0.01 mmol). ^1^H NMR (300 MHz, CD_3_OD): *δ*/ppm = 8.24 (d, *J* = 12.6 Hz, 2H), 5.90 (d, *J* = 5.4 Hz, 1H), 4.75 (t, *J* = 5.3 Hz, 1H), 4.17 (dd, *J* = 5.2, 4.1 Hz, 1H), 4.10 (q, *J* = 4.8 Hz, 1H), 3.62 (dd, *J* = 14.1, 5.6 Hz, 1H), 3.47 (dd, *J* = 14.1, 4.7 Hz, 1H), 1.85–1.68 (m, 12H). ^13^C NMR (75.5 MHz, CD_3_OD): *δ*/ppm = 180.6, 157.2, 153.5, 142.2, 90.5, 84.7, 74.8, 72.7, 42.1, 40.2, 29.1. FT-IR: *ν*/cm^−1^ = 3340, 2949, 2361, 1640, 1457, 1259, 1066. *α*^20^_D_ = n.d. mp: 116–118 °C. ESI-MS: *m*/*z* calculated for C_20_H_27_N_6_O_6_ [M + H]^+^ = 447.20 (100%); found: 447.00 (100%). Purity: 100% (HPLC, 254 nm, MeCN/H_2_O = 10 : 90 to 100 : 0 + 0.1% HCOOH, *t*_R_ = 2.52 min).

### Protein preparation and purification

#### Recombinant expression of SARS-CoV2 nsp14/10

Expression was performed as described previously in literature.^83^ Briefly, a plasmid coding for the N-terminally His-tagged nsp14/10 protein complex (Addgene plasmid # 159613; https://n2t.net/addgene:159613; RRID: Addgene_159 613) was kindly provided by the group of Hannah T. Baddock (University of Oxford, United Kingdom). The integrity of the plasmid was verified through sequencing by Eurofins Genomics Germany GmbH (Ebersberg, Germany). The plasmid was used to transform competent Rosetta 2(DE3)pLysS *E. coli* cells. Transformed *E. coli* Rosetta 2(DE3)pLysS cells were grown in TB medium supplemented with 50 μg mL^-1^ Kanamycin and 50 μM ZnCl_2_. The culture was incubated under shaking (190 rpm) at 37 °C until an optical density (600 nm) of 1.7 was reached. The cells were precooled to 18 °C for 30 min and overexpression was induced by addition of β-d-1-thiogalactopyranoside (IPTG) at a final concentration of 1 mM. After 18–20 h cells were collected by centrifugation (18 000*g*), cell pellets were flash-frozen in liquid nitrogen and stored at −80 °C until protein purification. For protein isolation, cell pellets were disrupted in nsp14/10-lysis buffer (50 mM Tris-HCl, pH 8.0, 500 mM NaCl, 10 mM imidazole, 5% (v/v) glycerol and 1 mM tris(2-carboxyethyl) phosphine (TCEP), 0.05% polysorbate-20), incubated with lysozyme (Carl Roth) and DNAse I (Roche CustomBiotech) for 40 min at 4 °C with subsequent 12 cycles of 45 s sonication at 50% power with 30 s intervals on ice. The lysate was centrifuged at 20000 rpm for 90 min at 4 °C, then filtered (0.2 μm) and loaded on a HisTrap excel 5 mL (Cytiva) column with the ÄKTA start system (Cytiva). The column was washed with ten column volumes of nsp14/10-lysis buffer and afterwards with four column volumes of a mixture of 93% nsp14/10-lysis buffer: 7% nsp14/10-elution buffer (50 mM Tris-HCl, pH 8.0, 500 mM NaCl, 300 mM imidazole, 5% v/v glycerol and 1 mM TCEP, 0.05% polysorbate 20). The protein was eluted in 100% nsp14/10-elution buffer. Size exclusion chromatography (SEC) was performed on a Superdex 16/600 75 pg column equilibrated in nsp14/10-SEC buffer (25 mM HEPES, pH 7.5, 150 mM NaCl, 5% v/v glycerol, 2 mM TCEP, 0.05% polysorbate-20). The nsp14/10 protein complex was concentrated with Sartorius Vivaspin 20 mL (30 kDa cut-off) centrifugal filters, aliquoted, flash frozen with liquid nitrogen, and kept at −80 °C until further usage.

#### Recombinant expression of human full-length DNMT2

Plasmid coding for the full-length human DNMT2 was kindly provided by Albert Jeltsch (University of Stuttgart, Germany). Transformed *E. coli* Rosetta (DE3) cells were grown in Terrific Broth medium (ForMedium) at 37 °C till optical density at 600 nm wavelength reached 0.8. After precooling to 20 °C for at least 30 min, overexpression was induced by 500 μM isopropyl-β-d-thiogalactopyranosid (IPTG) for 16–20 hours. Cells were collected by centrifugation at 10000 g for 20 min at 4 °C and washed in DNMT2-lysis buffer (50 mM sodium phosphate pH 8.0, 300 mM NaCl, 25 mM imidazole, 0.1% polysorbate-20). Cell pellets were flash frozen in liquid nitrogen and stored at −80 °C. For protein purification, cells were disrupted in DNMT2-lysis buffer (50 mM sodium phosphate pH 8.0, 300 mM NaCl, 25 mM imidazole, 0.1% polysorbate-20), supplemented with lysozyme (Carl Roth) and DNase I (Roche) and after 30–60 min of incubation, sonicated using the Hielscher UP200St-G Ultrasonic Generator (Hielscher Ultrasonics). During sonication, all samples were kept on ice, with constant temperature monitoring to ensure that the solution's temperature did not exceed 15 °C. The lysed cells were centrifuged at 17500 g for 20 min at 4 °C and filtered afterwards. HisTrap HP 5 mL column, pre-equilibrated with DNMT2-lysis buffer, was loaded with the clear filtrate using an ÄKTA start system (Cytiva) and subsequently washed with 10 column volumes of DNMT2-lysis buffer. To remove impurities such as histidine-rich proteins and other nonspecific binders, the column was washed with a mixture of DNMT2-lysis buffer and DNMT2-elution buffer (50 mM sodium phosphate, pH 8.0, 300 mM NaCl, 500 mM imidazole, 0.1% polysorbate-20), adjusted to a final imidazole concentration of 65 mM. DNMT2 protein was eluted with the 100% DNMT2-elution buffer. Size exclusion chromatography (SEC) was performed on a Superdex 16/600 75 pg column equilibrated in DNMT2-SEC buffer (50 mM sodium phosphate pH 8.0, 300 mM NaCl, 1 mM EDTA, 2 mM DTT, 0.1% polysorbate-20). The purified DNMT2 protein was concentrated using Amicon Ultra centrifugal filters with a 10 kDa cut-off (Millipore), aliquoted, flash-frozen with liquid nitrogen, and stored at −80 °C for further use.

#### Recombinant expression of human METTL3/14

The plasmid encoding the MTase domains of human METTL3 (UniProt: Q86U44 aa 252–580) harbouring a Strep tag-II at the C-terminus and METTL14 (UniProt: Q9HCE5 aa 108–444) harbouring a hexahistidine (His6) at the N-terminus was designed and ordered from Genscript and was already used in previous studies.^74^ The sequences were cloned into a pETduet-1 vector (cloning sites: *Eco*RI/*Xho*I) and the resulting plasmid was sequenced and transformed into competent *E. coli* BL21(DE3) cells. The transformed cells were grown at 37 °C in LB medium. At an OD600 of around 0.6, overexpression of METTL3/14 was induced by the addition of IPTG and ZnCl_2_ to a final concentration of 100 μM, respectively. Overexpression was done overnight, and the temperature was reduced to 18 °C. The cells were harvested by centrifugation and suspended in cold METTL3/14-lysis buffer (10 mM potassium phosphate, pH 7.5, 300 mM KCl, 20 mM imidazole, 10% glycerol, 0.1 mM TCEP) supplemented with lysozyme, one tablet of EDTA-free protease inhibitor cocktail (Merck) and DNase I. Afterwards the cells were incubated on ice for 30 min. Lysis was done by sonication (60% power, 10 cycles of 45 s). Cell debris was removed by centrifugation, and the clear supernatant was kept on ice. The pellet was sonicated in METTL3/14-lysis buffer for a second time and insoluble parts were removed by centrifugation. Afterwards, supernatants of both centrifugation steps were pooled and filtrated (0.45 μm). Purification of protein was done on a HisTrap HP 5 mL column, preequilibrated in binding buffer (10 mM potassium phosphate, pH 7.5, 300 mM KCl, 20 mM imidazole). After protein capture onto the column, it was washed with METTL3/14-wash buffer (10 mM potassium phosphate, pH 7.5, 500 mM KCl, 20 mM imidazole). Elution of METTL3/14 was performed with METTL3/14-elution buffer (10 mM potassium phosphate, pH 7.5, 300 mM KCl, 250 mM imidazole). To remove residual impurities a second purification step was performed by SEC on a Superdex 16/600 75 pg column equilibrated with METTL3/14-SEC buffer (25 mM Tris HCl, pH 7.5, 100 mM NaCl). For long-term storage METTL3/14 was flash-frozen in liquid nitrogen and stored at –80 °C.

### Fluorescence polarization assay (FP-assay)

For binding determination and inhibitor screening, a modified MTase fluorescence polarization displacement assay was performed using Greiner Bio-One black-bottom 96-well half area polypropylene plates.^74,84^ All measurements were performed on a Tecan Infinite F200 Pro microplate reader as technical triplicates in a total volume of 30 μL. The polarization filters are coupled to a monochromator setup (*λ*_ex_ = 480 nm, *λ*_em_ = 530 nm) and the polarization values (in mP) were calculated from polarization-specific parallel and orthogonal fluorescence intensities. The fluorescence was measured at 25 °C.

#### *K*_D_-determination of FTAD

*K*_D_ evaluation for FTAD against nsp14/10 was performed by measuring a dilution series of nsp14/10 with a constant FTAD-concentration. Each well contained a total volume of 30 μL including buffer (25 mM HEPES, pH 7.5, 150 mM NaCl, 5% glycerol, 2 mM TCEP, 0.01% triton X-100), 50 nM FTAD and nsp14/10 (2-fold dilution series from 3.00 μM to 46.9 nM; *K*_D_(FTAD, nsp14/10) = 1.3 ± 0.3 μM; Fig. S8). Each measurement was performed as technical triplicates. *K*_D_ evaluation for FTAD against DNMT2 and METTL3/14 were performed and published previously (*K*_D_(FTAD, DNMT2) = 6.4 ± 0.7 μM; *K*_D_(FTAD, METTL3/14) = 1.10 μM).^74^

#### Determination of Z' factor

*Z*′ factors for DNMT2 and METTL3/14 were determined and published previously.^74,84^ For assay quality assessment, a *Z*′ factor was determined for nsp14/10.^73^ SAH and DMSO were used as positive and negative controls, respectively. 1 μL of 100 μM SAH or DMSO were added to 29 μL of FTAD and nsp14/10 in assay buffer (25 mM HEPES, pH 7.5, 150 mM NaCl, 5% glycerol, 2 mM TCEP, 0.01% triton X-100) to final concentrations of 50 nM FTAD, 1 μM nsp14/10 and 3% DMSO. This was performed for 24 replicates of each control (Fig. S7).

#### Screening of SARS-CoV-2 nsp14/10, DNMT2, METTL3/14

The screening of compounds 4–54 was conducted under the same conditions as described above. 1 μL of compound was added to a mixture of 29 μL containing FTAD and enzyme in assay buffer (nsp14/10: 25 mM HEPES, pH 7.5, 150 mM NaCl, 5% glycerol, 2 mM TCEP, 0.01% triton X-100; DNMT2: 50 mM HEPES pH 7.5, 150 mM NaCl, 1 mM DTT, 0.1% PEG-8000, 0.05% PS-20; METTL 3/14: 25 mM Tris-HCl, pH 7.5, 100 mM NaCl) to final concentrations of 100 μM compound, 50 nM FTAD, 1 μM enzyme and 3% DMSO. SAH (DNMT2, nsp14/10) and SFG (METTL3/14) and DMSO were used as positive and negative controls, respectively. The measurements were performed as technical triplicates. For nsp14/10, compounds exhibiting probe displacement at 100 μM concentration as good as SAH, were selected and measured at 10 μM and 1 μM. *K*_D_-determination for compounds 34, 35, and 46 against nsp14/10 and compounds 34, 40, and 42 against METTL3/14 were performed in a total volume of 30 μL including buffer, 50 nM FTAD and 1 μM enzyme and a 2 to 3 fold compound dilution series specifically chosen for each compound. Each measurement was performed as technical triplicates (Fig. S10–S14). FP experiments were analyzed using GraphPad Prism (version 10.4.0., GraphPad Software, Boston, Massachusetts, USA, https://www.graphpad.com).

### Microscale thermophoresis assay (MST)

For *K*_D_ determination of compounds 29, 30, and 41 against nsp14/10, microscale thermophoresis measurements were performed using a Monolith NT.115 Pico instrument (NanoTemper Technologies, Muenchen, Germany) and standard Monolith NT.115 capillaries (NanoTemper Technologies, Muenchen, Germany). The blue channel with an excitation power of 40% and medium MST power was used to measure the emission fluorescence at 25 °C. MST data was analysed using MO. Affinity Analysis software v2.3 (NanoTemper Technologies, Muenchen, Germany) and GraphPad Prism 10.4.0. *K*_D_ evaluation for FTAD against nsp14/10 was performed by measuring a 3-fold dilution series of nsp14/10 with a constant FTAD-concentration (50 nM), *K*_D_(FTAD, nsp14/10) = 1.2 ± 0.1 μM, Fig. S8). For an initial compound screening to validate the assay, FTAD and nsp14/10 were pre-incubated in MST-buffer (25 mM HEPES, pH 7.5, 150 mM NaCl, 5% glycerol, 2 mM TCEP, 0.01% triton X-100) for 5 min before either the compound in buffer or DMSO in buffer were added and the mixtures were incubated for further 10 min before measurement. Final concentrations of the components were: 50 nM FTAD, 100 μM compound/SAH, 0.13 μM nsp14/10 and >1% DMSO. For *K*_D_ determinations, a 3-fold dilution series of the respective compounds in buffer was prepared ranging from 6.25 μM to 2.86 nM and pre-incubated for 5 min with the enzyme–probe mixture upon measurement (Fig. S10–S12). Each measurement was performed as technical triplicates in a total volume of 10 μL. For MST-shift determination and analysis, *F*_norm_ values at 5 s laser “on”-time were used.

### Conversion to *K*_D_-values

These obtained apparent *K*^app^_D_ -values were converted to actual *K*_D_-values using eqn (1). Therefore, the enzyme concentration [E] and the *K*_D_-value of FTAD (*K*^probe^_D_) against the respective enzyme were utilized.1
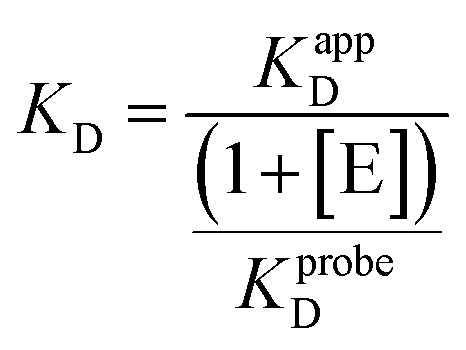


### Isothermal titration calorimetry (ITC)

For determination of binding affinities, isothermal titration calorimetry was performed using a MicroCal PEAQ-ITC Automated (Malvern Panalytical, Malvern, UK) with a 200 μL Hastelloy cell and an injection syringe volume of 40 μL. Nsp14/10 was provided in SEC-buffer (25 mM HEPES, pH 7.5, 150 mM NaCl, 5% glycerol, 0.5% DMSO) and diluted to a final concentration of 10 μM. Compounds were diluted with SEC-buffer to a final concentration of 100 μM to an overall DMSO content of 0.5%. DMSO-content of the enzyme solution was adjusted accordingly to 0.5%. Measurements were performed with 19 ligand injections à 2 μL at 25 °C, a reference power of 10 μcal s^−1^ and stirring speed of 750 rpm as duplicates. ITC data was analysed using MicroCal PEAQ-ITC Analysis Software 1.21 (Malvern Panalytical, Malvern, UK). Due to the high sample consumption, thermodynamic binding profiles are not corrected for buffer ionization effects (Fig. S15–S17, Table S5, Fig. 4).^85,86^

### Virtual synthesis

16 chemical supplier catalogues with the annotation “building block” or “building block economical”, describing compounds sold in preparative quantities, were obtained from the ZINC database^20^ (Table S6). These 1 825 558 entries were filtered (Table S6) for a log *P*^59^ < 3.6 using MOE. This should allow the extraction of unreacted educt with saturated NaHCO_3_-solution, because 5′-amino-5′-deoxyadenosine is soluble under basic conditions, and the respective amides and sulfonamides remain in the organic phase. BBs containing moieties incompatible with the procedure, namely alcohols and free amines, were removed as well as duplicates to result in 76 678 unique acids, 2729 acid chlorides and 4628 sulfonyl chlorides. These were virtually reacted with 5′-amino-5′-deoxyadenosine using the reaction-synthesizer (version 5.3.0) functionality of the CoLibri tool (CoLiBri 8.0.0, BioSolveIT GmbH, Sankt Augustin, Germany, 2023 https://www.biosolveit.de) from command-line to form amides (reaction “rxn101: amide”) and sulfonamides (reaction “rxn210: sulfonamide”) as described in the “cookbook” (https://www.biosolveit.de/infiniSee /#chemical_spaces, accessed 21.03.2023) of the eXplore chemical space described previously.^17^ The reaction products were filtered to remove reactive moieties and PAINS,^78,79^ and to violate the RO5 in at most one parameter. Additionally, the log *P* value was limited to be −1.8 or higher to be compatible with the extraction procedure, thus no compound transfers into the aqueous phase. The final chemical space of 5′-amino-5′-deoxyadenosine amides and sulfonamides consisting of 25 241 molecules (22 969 amides and 2272 sulfonamides) is provided in SMILES file format for virtual screenings (SI). All physico-chemical property prediction and filtering was performed using MOE (Molecular Operating Environment (MOE), 2020.02 or 2024.0601 Chemical Computing Group ULC, 1010 Sherbooke St. West, Suite #910, Montreal, QC, Canada, H3A 2R7, 2023.). Functional group filters were applied using RDKit 4.0.1 (Open-source cheminformatics; https://www.rdkit.org) within KNIME 3.7.1.^87^ An extended version of the library (version2, 110 049 cpds, Table S6) allowing for two RO5 violations and an enhanced BB catalogue is also provided. The economic library was prepared and used in the same manner and resulted in 14 228 molecules (version2eco). Structural diversity of the libraries was calculated using the iSIM method with RDKit fingerprints.^80,81^

### Virtual screening

Molecular docking of SARS-CoV-2 nsp14/10 was performed using the 3D crystal structure with PDB-ID: 7R2V^82^ obtained from the protein data bank.^88^ The receptor was prepared with the OpenEye toolkit Make_receptor 4.2.1.1 (OpenEye Scientific Software, Cadence Molecular Sciences, Inc., Santa Fe, NM. https://www.eyesopen.com)^89–91^ and the binding site was defined from the crystallographic reference ligand SAH with a box volume of 15 627 Å^3^ (dimensions: 28.3 Å × 22.7 Å × 24.3 Å) to cover the SAH binding-site and the sub-site of the substrate RNA nucleobase The chemical space library of 27 764 compounds were protonated with MOE (Molecular Operating Environment (MOE), 2020.09 Chemical Computing Group ULC, 1010 Sherbooke St. West, Suite #910, Montreal, QC, Canada, H3A 2R7, 2023.) and their 3D conformations were generated with OMEGA-pose 4.1.2.0 (OpenEye Scientific Software, Santa Fe, NM, USA; https://www.eyesopen.com, 2019).^92^ For unspecified stereo centres, both stereoisomers were created prior conformer generation (40584) and macrocycles were excluded (35 523 remaining molecules). The docking was performed with HYBRID-4.1.1.0 (Openenye Scientific Software, Santa Fe, NM.; https://www.eyesopen.com).^89,90,92^ The-setup was validated by re-docking of the crystallographic reference ligand SAH using Open Babel (version 3.1.0, https://www.openbabel.org, accessed Aug 2023)^93^ (Hybrid ChemGauss4 score −10.4 kcal mol^-1^; RMSD: 1.75 Å (Fig. S21)). SAM-like nsp14/10 inhibitors with IC_50_-values <1 μM (ref. 54–56) were compiled from literature. Physicochemical property-matched decoys were generated using LiDeB.^92,94^ Protonation and conformer generation was performed as described for SAH. For unspecified stereo centres in decoys, both stereoisomers were created using OMEGA resulting in 10 182 decoy molecules. The docking was performed with HYBRID-4.1.1.0 (Openenye Scientific Software, Santa Fe, NM.; https://www.eyesopen.com).^89,90,92^ Binder-decoys discrimination was evaluated by receiver operating characteristics (ROC) area under the curve (AUC) analysis with an ROC-AUC of 0.85 and an ROC-logAUC_0.1–100%_ of 0.25. Docking of the space1 was performed the same way. Binding modes of the top 6% of the docked molecules by docking score were visually inspected. Molecules with a conserved binding mode of the adenosine sub-structure, substituents expanding towards the RNA-substrate binding site stacking with Phe426, and purchaseability/economy of BBs, were prioritized for synthesis. PyMOL-2.0.5 (Schrödinger, L., & DeLano, W. (2020). PyMOL. Retrieved from https://www.pymol.org/pymol) was used for pose inspection and figure generation.^83,84,86^

## Author contributions

S. N. H.: conceptualization, investigation, methodology, data curation, formal analysis, validation, visualization, writing – original draft. M. S. (Marvin Schwickert) conceptualization, methodology, investigation, writing – review & editing. L. K., Z. N., M. S. (Mark Sabin), A. C. W., J. L. M.: investigation. F. B., T. S. resources, funding acquisition, supervision, writing – review & editing. C. K.: conceptualization, methodology, software, supervision, project administration, writing – review & editing.

## Conflicts of interest

The authors declare no conflicts of interest.

## Supplementary Material

MD-OLF-D5MD00376H-s001

MD-OLF-D5MD00376H-s002

## Data Availability

The following mentioned data were included as part of the ESI: Biochemical assay plots: FP/MST-Assay Fig. S6–14, ITC thermograms, stoichiometry plots and signature plots Fig. S15–S17, Table S5, NMR/LC-MS chromatograms S23–S231, Table S9, experimentally derived yields and purities (Table S1), calculated physicochemical properties (Tables S2, S7, Fig. S18–20, Table S8), molecular docking (Fig. S21) virtual synthesis details Table S6, virtual synthesis libraries (txt). See DOI: https://doi.org/10.1039/D5MD00376H.
