# Supplementary material for: Parallel synthesis of 5′-amino-5′-deoxy-adenosine derivatives for focused chemical space exploration and their application as methyltransferase inhibitors
Source: RSC Med Chem. 2025 Aug 12;16(11):5395–418. doi: 10.1039/d5md00376h (PMC12412055; doi:10.1039/d5md00376h)
Supplement: MD-016-D5MD00376H-s001 [file MD-016-D5MD00376H-s001.pdf]

Supplementary Information (SI) for RSC Medicinal Chemistry.
This journal is © The Royal Society of Chemistry 2025

Supporting Information

Parallel Synthesis of 5'-amino-5'-deoxy-adenosine Derivatives for Focused Chemical Space Exploration and their Application as Methyltransferase Inhibitors†

Sabrina N. Hoba[a,&], Marvin Schwickert[a,&], Luis Kammerer [a], Mark Sabin[a], Annabelle C. Weldert[a], Zarina Nidoieva[a], J. Laurenz Meidner[a], Fabian Barthels[a], Tanja Schirmeister[a] and Christian Kersten[a,b,*]

[a] Institute of Pharmaceutical and Biomedical Sciences, Johannes Gutenberg University Mainz, Staudinger Weg 5, 55128 Mainz, Germany.
[b] Institute for Quantitative and Computational Bioscience, Johannes Gutenberg-University Mainz, BioZentrum I, Hanns-Dieter-Hüsch Weg 15, 55128 Mainz, Germany.

Table of Contents

1. Yields and Purities obtained by parallel synthesis 1
2. LC-MS Chromatogram Adenine Cleavage 1
3. LC-MS Chromatogram of cpd 25 2
4. Side products occurred during parallel synthesis 2
5. Calculated Physicochemical Properties 3
6. Scope Expansion and Limitation 4
7. FP-Assay/MST-Assay Plots 7
8. FP-Assay Raw Data cpd 42 9
9. ITC Data 10
10. Virtual Synthesis 12
11. Predicted Physicochemical Properties of Chemical Spaces 13
12. Molecular docking 15
13. Table of key spectroscopic features 17
14. Spectra and Chromatograms 18
15. References 123

1. Yields and Purities obtained by parallel synthesis

Table S1. Experimentally derived yields and purities obtained by the parallel synthesis.

cpd. ID	Yield (%)	Purity (%)	cpd. ID	Yield (%)	Purity (%)	cpd. ID	Yield (%)	Purity (%)	cpd. ID	Yield (%)	Purity (%)
**4**	24	96	**17**	99	100	**30**	99	86	**43** [a]	66	n.d.
**5**	43	97	**18**	77	99	**31**	64	97	**44**	99	97
**6**	93	98	**19**	99	100	**32**	99	99	**45**	57	97
**7**	21	99	**20**	99	100	**33**	95	99	**46**	57	96
**8**	79	96	**21**	99	98	**34**	79	98	**47**	86	95
**9**	50	97	**22**	0	-	**35**	50	99	**48**	99	97
**10**	50	96	**23**	0	-	**36**	50	95	**49**	93	96
**11**	57	95	**24**	0	-	**37**	57	97	**50**	43	99
**12**	29	98	**25**	-	-	**38**	99	98	**51**	79	100
**13**	64	100	**26**	21	-	**39**	71	98	**52**	71	99
**14**	48	98	**27**	29	95	**40**	86	96	**53**	21	95
**15**	99	99	**28**	57	98	**41**	42	97	**54**	63	96
**16**	85	96	**29**	43	99	**42**	64	99			

[a] Deacetylation occurred during deprotection. No isolation of the final compound. *n.d.* signifies not determined.

2. LC-MS Chromatogram Adenine Cleavage

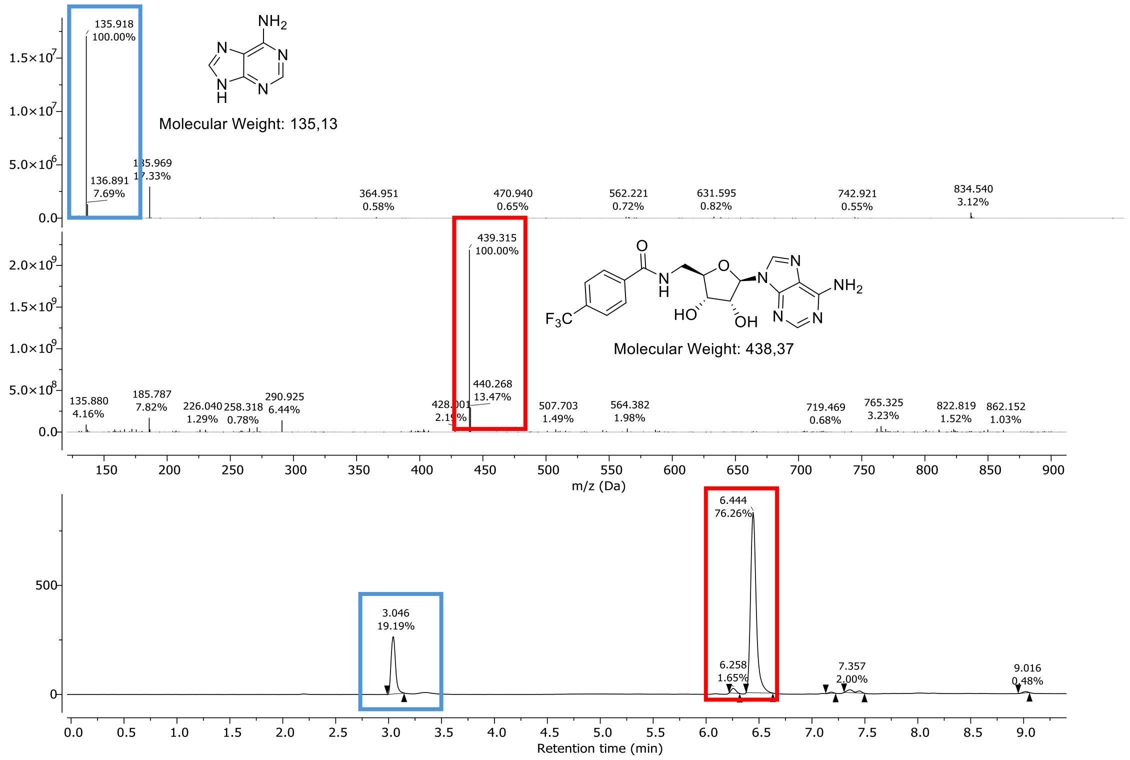

Figure S1. Exemplary LC-MS chromatogram and MS spectra of compound **5** (red) at 254 nm. Adenine cleavage is highlighted in blue.

3. LC-MS Chromatogram of cpd 25

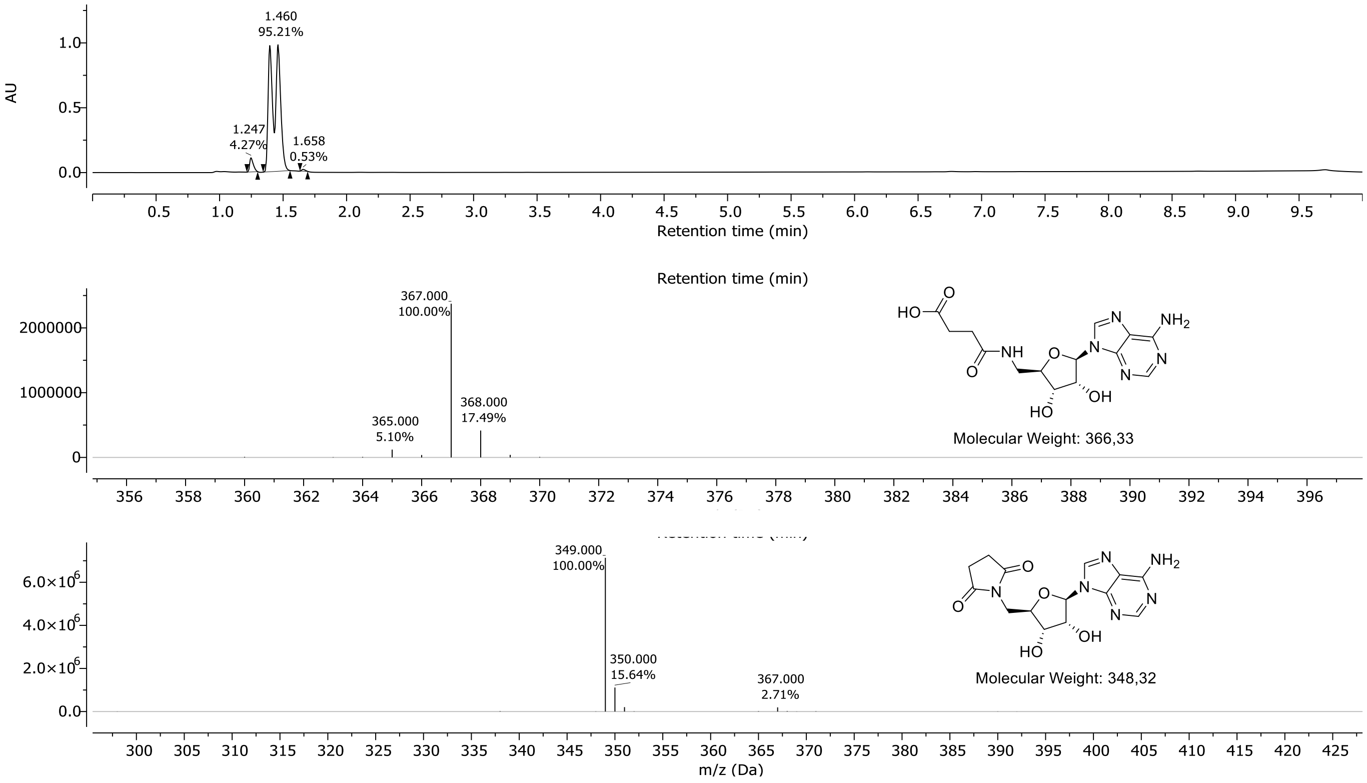

Figure S2. LC-MS chromatogram and MS spectra of compound **25** at 254 nm showing both side products.

4. Side products occurred during parallel synthesis

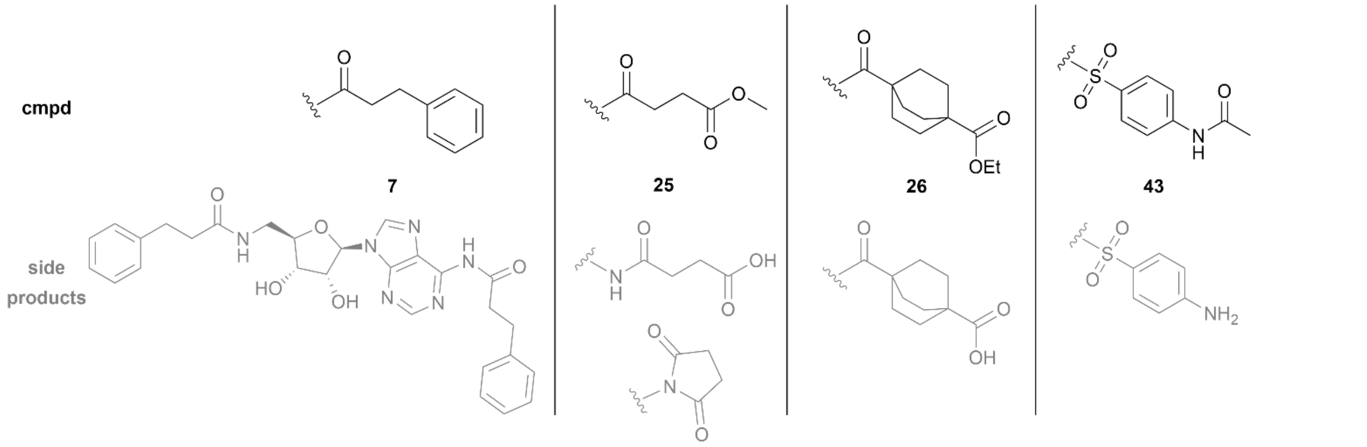

Figure S3. Chemical Structures of side products occurred during the parallel synthesis of compounds **7**, **25**, **26** and **43**.

5. Calculated Physicochemical Properties

Table S2. Calculated physicochemical parameters SlogP, logD, pK$_a$ and TPSA.

cpd.	BB[a]		precursor[b]		final cpd.			
ID	SlogP[1]	TPSA (Å)[2]	SlogP[1]	TPSA (Å)[2]	SlogP[1 c]	logD$_{7.0}$[3]	pKs[3]	TPSA (Å[2])[2]
**4**	2.3	17.1	1.6	126.4	−0.2	−0.2	–	148.4
**5**	3.4	17.1	2.9	126.4	0.9	0.3	-	148.4
**6**	2.1	17.1	1.4	126.4	−0.4	−0.5	–	148.4
**7**	2.4	17.1	1.7	126.4	−0.1	−0.3	–	148.4
**8**	1.6	56.0	0.9	165.3	−0.9	−0.7	–	187.3
**9**	1.8	35.5	1.1	144.9	−0.7	−1.1	–	166.9
**10**	2.0	62.9	1.3	172.2	−0.5	−0.5	–	194.2
**11**	2.2	17.1	1.5	126.4	−0.3	−0.5	–	148.4
**12**	2.1	26.3	1.4	135.6	−0.4	−0.6	–	157.6
**13**	0.9	20.3	0.2	129.7	−1.6	−2.5	–	151.7
**14**	3.1	26.3	2.5	135.6	0.7	0.4	–	157.6
**15**	2.4	17.1	1.7	126.4	−0.1	0.0	–	148.4
**16**	1.8	17.1	1.1	126.4	−0.7	−0.8	–	148.4
**17**	2.3	17.1	1.6	126.4	−0.2	−0.6	–	148.4
**18**	1.5	30.0	0.8	139.3	−1.1	−1.2	–	161.3
**19**	2.9	17.1	2.1	126.4	0.4	0.5	–	148.4
**20**	3.4	17.1	2.7	126.4	0.9	0.9	–	148.4
**21**	4.7	20.3	3.9	129.7	2.1	1.6	–	151.7
**22**	0.6	25.6	−1.5	134.1	−3.3	−3.6	7.8	156.1
**23**	2.1	44.8	1.4	154.1	−0.4	−0.6	–	176.1
**24**	2.4	62.9	1.7	172.2	−0.1	0.8	–	194.2
**25**	0.7	43.4	0	152.7	−1.8	−2.1	-	174.7
**26**	2.7	43.4	1.9	152.7	0.14	−1.3	10.3	174.7
**27**	1.1	37.4	−0.1	146.7	−1.5	−1.9	-	168.7
**28**	3.5	63.7	2.8	173.0	1.0	2.2	-	195.0
**29**	1.8	100.3	3.0	151.8	−0.8	−0.9	-	231.6
**30**	2.3	34.1	1.6	143.5	−0.2	0.8	9.3	166.5
**31**	1.5	80.0	0.8	189.3	−1.0	−1.2	9.9	211.3
**32**	2.3	34.1	1.6	143.5	−0.3	−0.6	10.2	165.5
**33**	1.5	80.0	0.8	189.3	−1.0	−1.2	10.0	211.3
**34**	2.8	34.1	2.1	143.5	0.3	−0.2	10.1	165.5
**35**	2.2	80.0	1.5	143.5	−0.3	−0.8	10.1	165.5
**36**	4.3	34.1	3.6	143.5	1.8	0.5	9.9	165.5
**37**	2.2	34.1	1.5	189.3	−0.3	0.3	9.9	211.3
**38**	2.8	34.1	2.1	143.5	0.3	−0.1	10.1	165.5
**39**	5.0	80.0	4.3	143.5	2.5	2.9	10.1	165.5
**40**	3.3	34.1	2.6	143.5	0.8	0.4	10.1	165.5
**41**	2.0	34.1	1.3	143.5	−0.5	−0.8	10.0	165.5
**42**	2.8	34.1	2.1	146.7	0.3	0.1	10.1	168.7
**43**	1.6	34.1	0.9	172.6	−0.9	−1.5	10.2	194.6
**44**	2.3	37.4	1.6	189.3	−0.2	−0.1	9.9	211.3
**45**	1.6	63.2	0.8	169.8	−1.0	−0.5	9.8	191.8
**46**	3.6	34.1	2.9	143.5	1.1	0.4	10.1	165.5
**47**	1.8	80.0	1.1	189.3	−0.7	−0.9	9.9	211.3

**48**	2.9	34.1	2.2	156.4	−0.9	−1.1	10.1	165.5
**49**	1.7	47.0	1.0	143.5	0.4	0.3	10.0	178.4
**50**	2.0	63.7	1.2	173.0	−3.2	−4.7	8.4	182.1
**51**	1.0	37.4	0.3	177.6	−1.5	−2.0	10.0	199.6
**52**	0.2	43.4	−0.5	152.7	−2.3	−3.1	10.3	174.7
**53**	1.8	114.	1.1	223.4	−0.7	0.4	9.6	245.4
**54**	0.8	117.3	0.1	226.7	−1.7	−1.4	9.6	248.7

[a]SlogP of BB. protonation state at pH = 7.0. [b]SlogP of precursor = compounds with incorporated isoproylidene protecting group, protonation state at pH = 7.0. [c] SlogP of final, deprotected compounds, protonation state at pH = 7.0.

6. Scope Expansion and Limitation

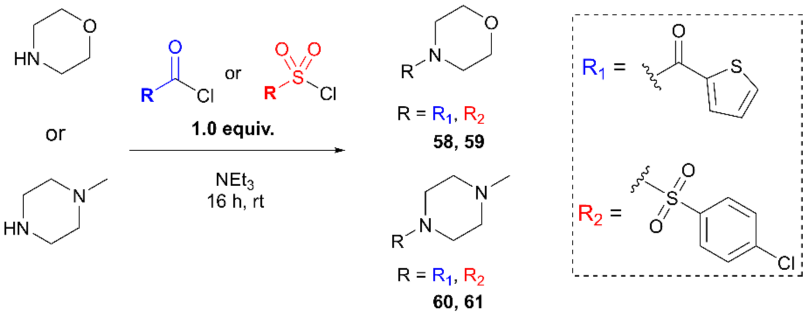

Scheme S1. Synthesis scheme for the generation of amides (**59**, **61**) and sulfonamides (**60**, **62**) based on the parallel synthesis method.

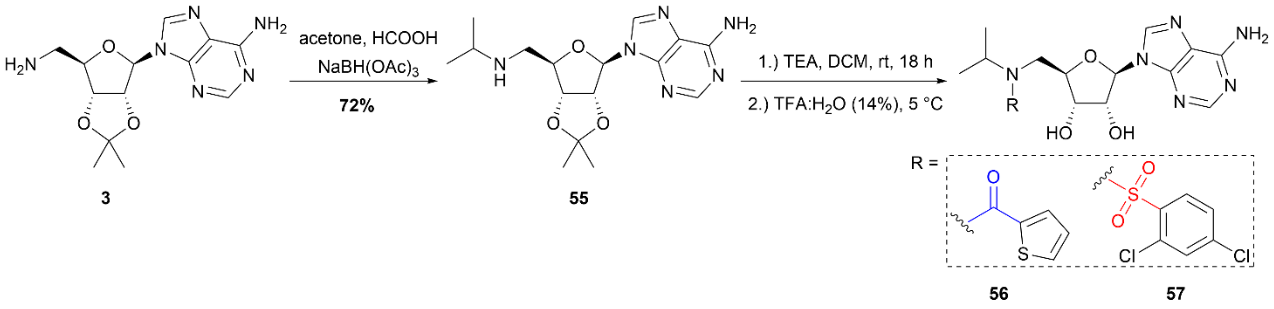

Scheme S2. Synthesis scheme for the generation of the secondary amine 55 and the application of the parallel synthesis to the respective amides and sulfonamides..

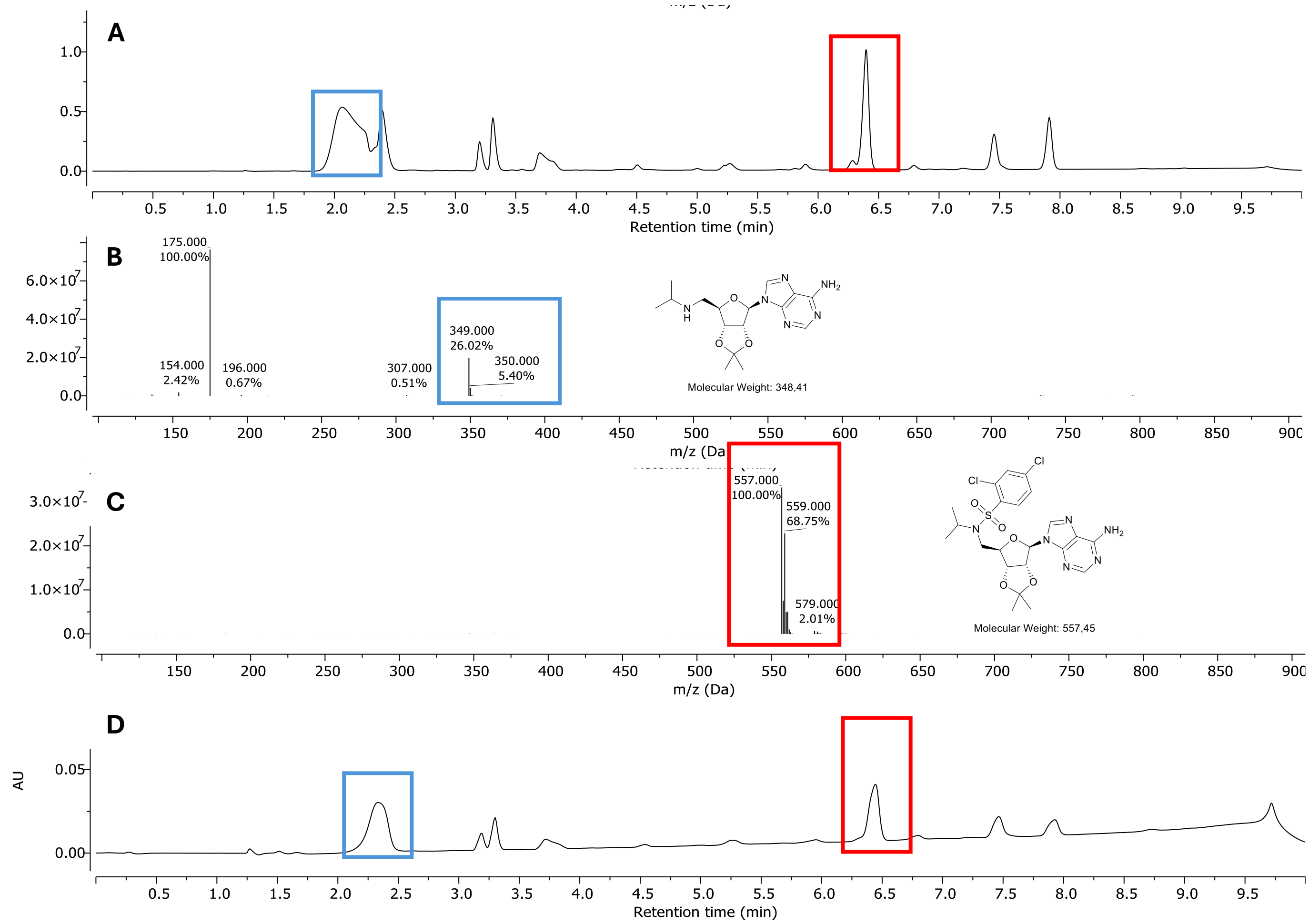

Figure S4. LC-MS chromatogram and MS spectra of parallel synthesis reaction to compound **57** at 254 nm. (**A**) LCMS chromatorgam of reaction showing **55** and protected **57**. (**B**) MS spectra of **55** from the reaction. (**C**) MS spectra of protected **57** from the reaction. (**D**) LCMS chromatogram of organic phase after extraction containing both **55** and protected **57**.

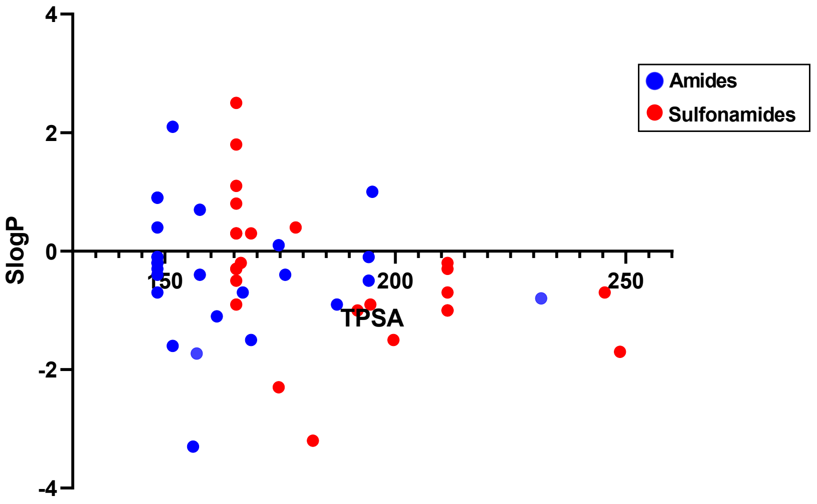

Figure S5. Plot of calculated SlogP[1] against TPSA[2] of the final compounds of the parallel synthesis (**3**–**54**).

Table S3. Comparison of yields and purities obtained by either liquid-liquid extraction or HPLC.

	**liquid-liquid extraction**		**HPLC**	
cmd. ID	yield	purity	yield	purity
4	24	96	64	100
6	93	98	79	98
41	42	97	71	99
48	99	97	64	100

7. FP-Assay/MST-Assay Plots

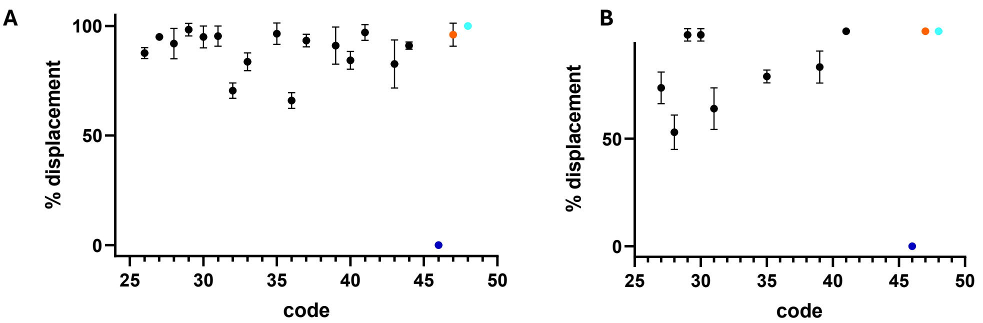

Figure S6. Scatter plots for the displacement of FTAD through the adenosine-based compounds derived from the parallel synthesis. FTAD is displaced out of the SAM-binding site of nsp14/10 at 10 µM (**A**) and 1 µM (**B**) ligand concentration. • free probe (FTAD); • SAH; • probe-enzyme complex. Each datapoint represents the mean ± SD of triplicates. Code represents the individual cpd IDs.

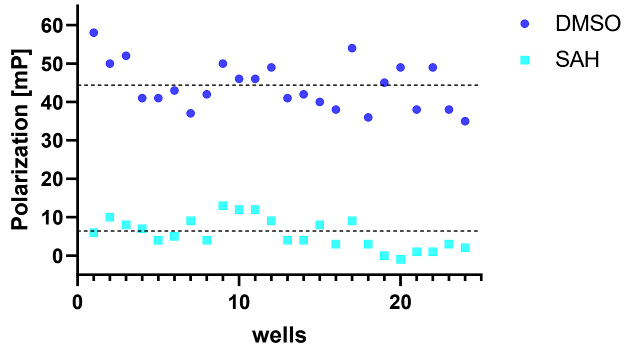

Figure S7. Z'-factor determination for nsp14/10 FP-assay in absence (DMSO) and presence of competing ligand (*100* µM SAH).

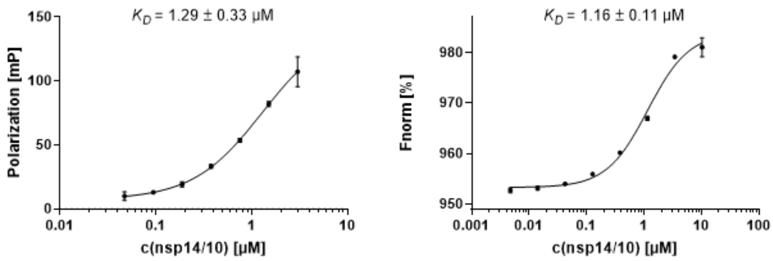

Figure S8. Affinity determination of **FTAD** to nsp14/10 via FP-assay (left, 2-fold dilution series) and MST-assay (right, 3-fold dilution series).

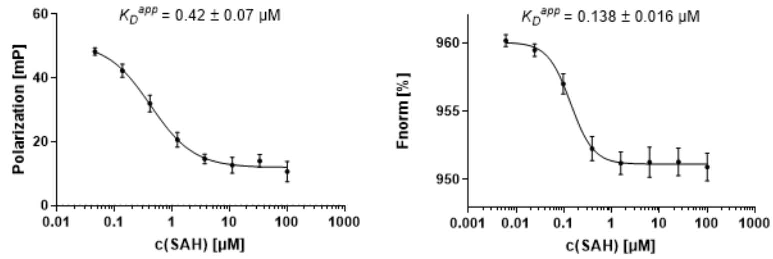

Figure S9. Affinity determination of **SAH** to nsp 14/10 via FP-assay (left, 3-fold dilution series) and MST-assay (right, 4-fold dilution series).

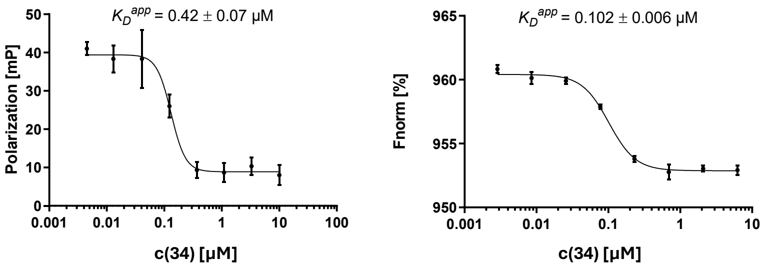

Figure S10. Affinity determination of **34** to nsp14/10 via FP-assay (left, 3-fold dilution series) and MST-assay (right, 4-fold dilution series).

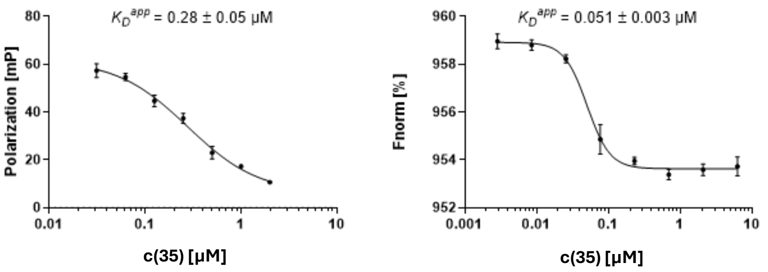

Figure S11. Affinity determination of **35** to nsp10/14 via FP-assay (left, 2-fold dilution series) and MST-assay (right, 4-fold dilution series).

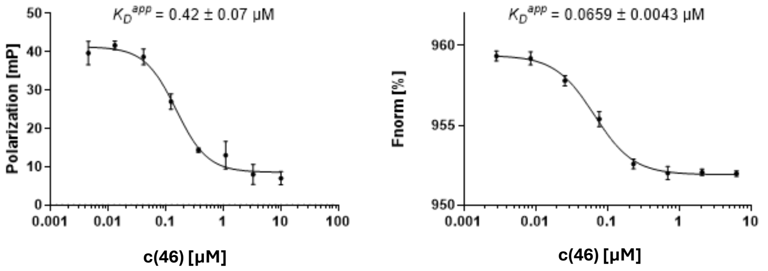

Figure S12. Affinity determination of **46** to nsp10/14 via FP-assay (left, 3-fold dilution series) and MST-assay (right, 4-fold dilution series).

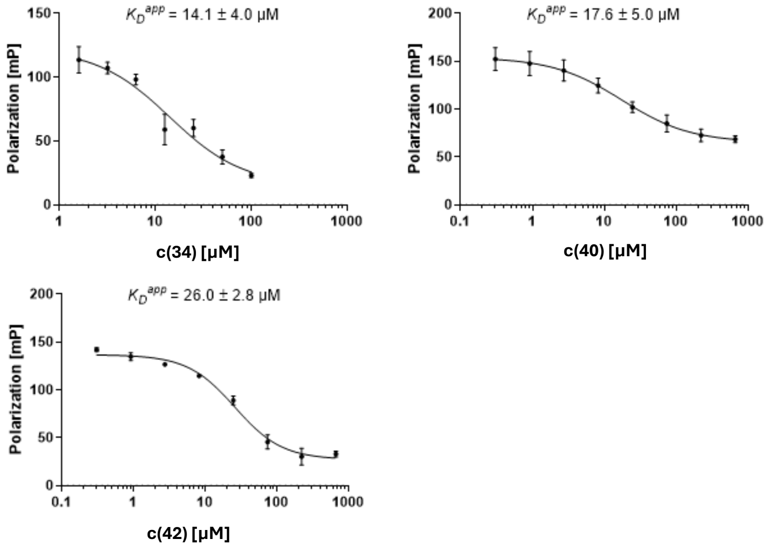

Figure S13. Affinity determination of compounds **34 (A,** 2-fold dilution series**), 40** (**B,** 3-fold dilution series) and **42** (**C,** 3-fold dilution series) to METTL3/14 via FP-assay.

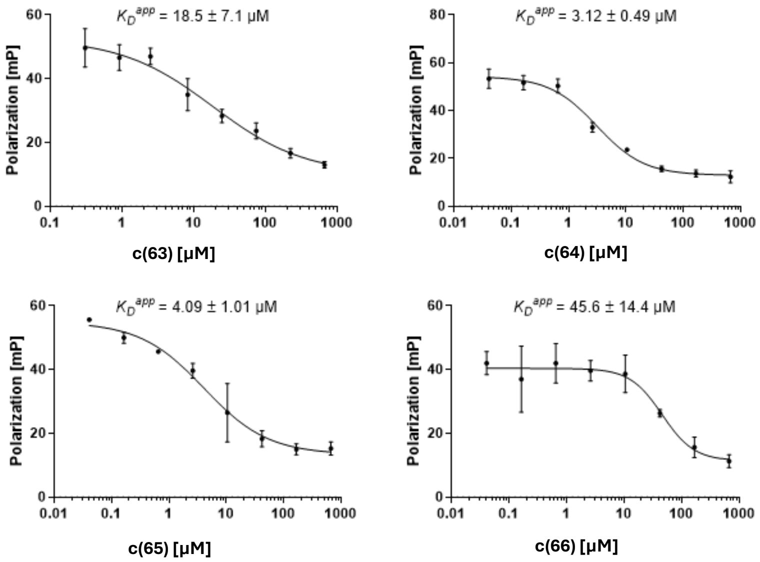

Figure S14. Affinity determination of compounds **63** (**A,** 3-fold dilution series)**, 64** (**B,** 4-fold dilution series**)**, **65** (**C,** 4-fold dilution series) and66 (**D,** 4-fold dilution series) to nsp14/10 via FP-assay.

8. FP-Assay Raw Data cpd 42

Table S4. Raw data (mP and total intensity) of fluorescence polarization displacement assay of cmp. **42**, **SAH** and **probe-enzyme** at 100 µM and dilution series of cpd. **42**.

cmp. ID	µM[a]	mP			total intensity		
SAH	100	13	7	8	15527	14218	14357
DMSO[b]	100	149	148	146	15028	15209	15278
42	100	44	42	39	18205	16177	16524
42	666	30	36	33	25251	24796	26626
	222	23	28	40	22984	20625	20534
	74.0	40	43	54	17281	16313	18091
	24.7	90	84	93	17750	16617	17703
	8.22	116	113	115	16190	14718	16069
	2.74	128	126	126	15091	15438	15739
	0.914	131	134	139	16613	15443	18075
	0.305	144	142	140	16997	14743	15446

[a] concentration of ligand. [b] only probe-enzyme complex without any ligand.

9. ITC Data

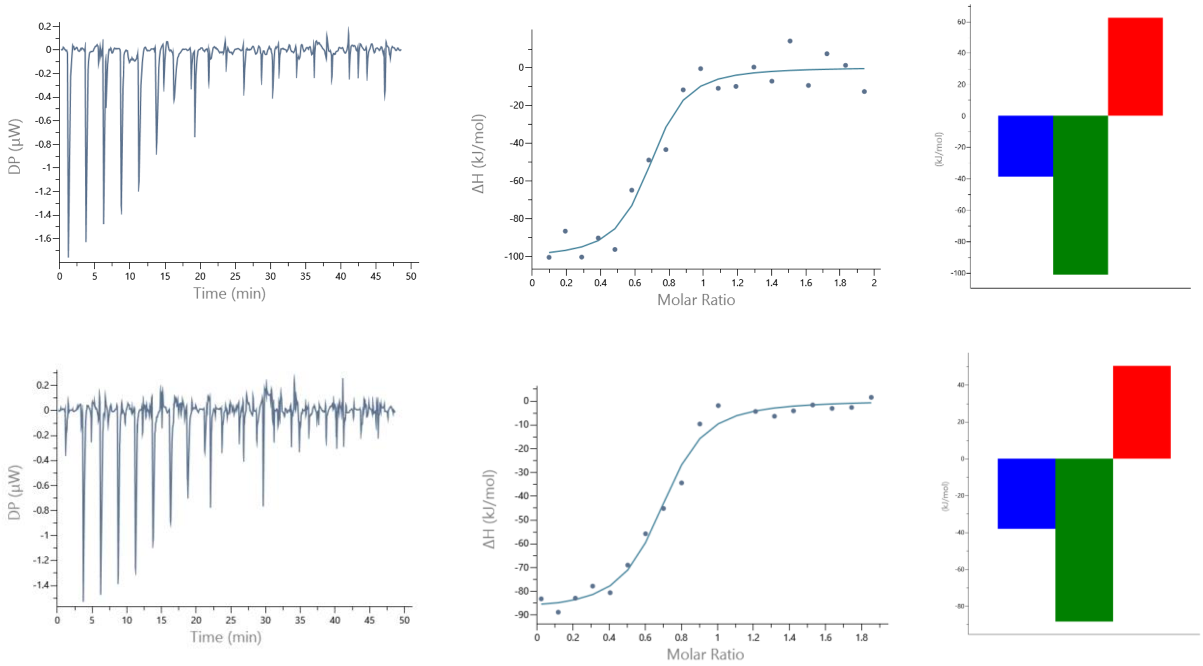

Figure S15. ITC thermograms *(left)*, *binding isotherms (mid)* and *thermodynamic* signature plots *(right),*(n = 1) for compound **34** *binding to nsp14/10*. Duplicates were used for mean ± SD determination.

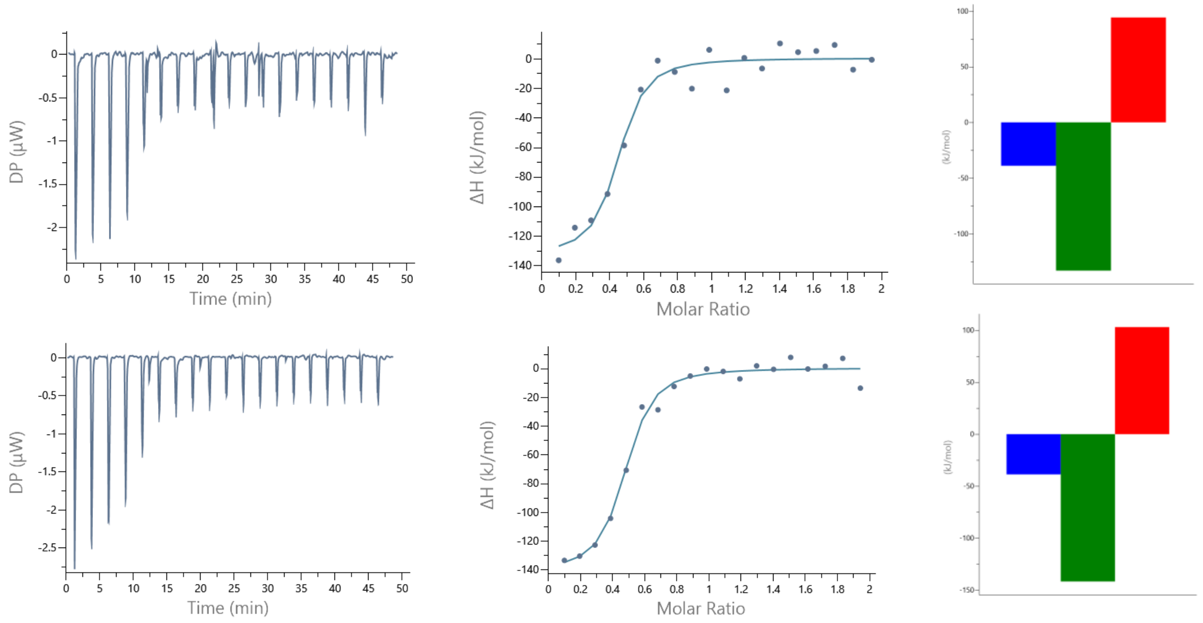

Figure S16. ITC thermograms *(left)*, *binding isotherms (mid)* and *thermodynamic* signature plots *(right),*(n = 1) for compound ***35*** *binding to nsp14/10*. Duplicates were used for mean ± SD determination.

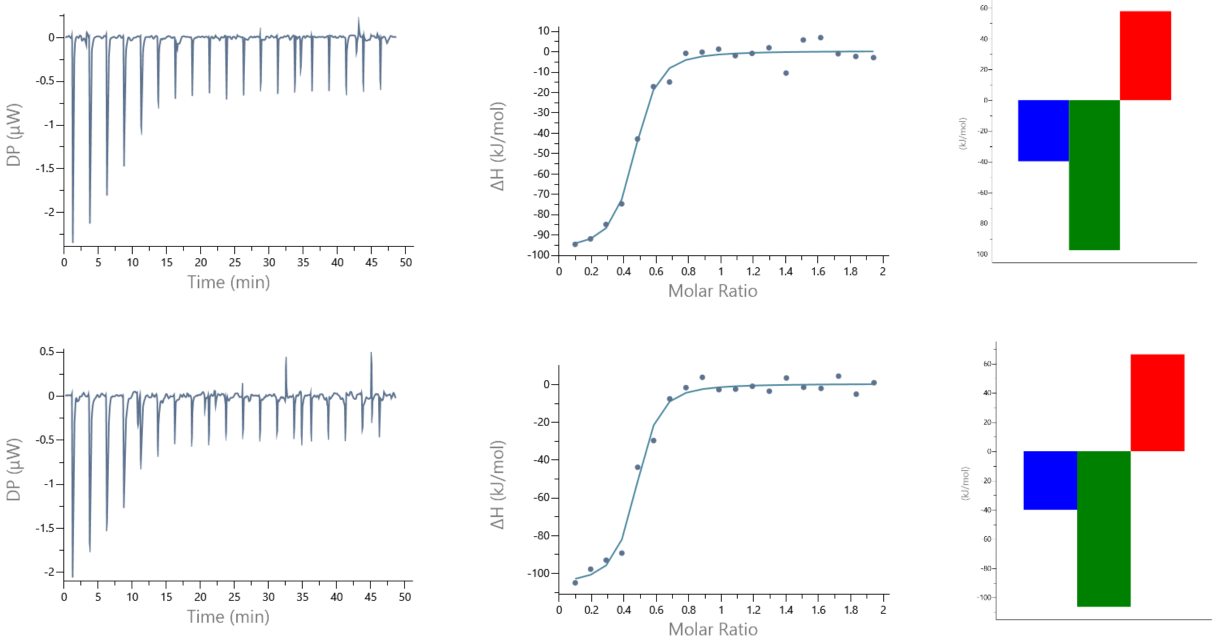

Figure S17. ITC thermograms *(left)*, *binding isotherms (mid)* and *thermodynamic* signature plots *(right),*(n = 1) for compound **46** *binding to nsp14/10*. Duplicates were used for mean ± SD determination.

Table S5. Experimentally derived binding affinity (K_D), stoichiometry (n) and thermodynamic properties (Δ*G*, Δ*H*, −TΔ*S*) of compounds **34**, **35** and **46** binding to nsp14/10 obtained by ITC. Mean values ± SD are presented in Figure S4 of the manuscript.

**code**	**K_D (µM)**	**n**	**Δ*H* (kJ/mol)**	**Δ*G* (kJ/mol)**	**-TΔ*S* (kJ/mol)**
**34**	0.165	0.66	-101	−38.8	62.4
**34**	0.214	0.67	−88.5	−38.1	50.4
**35**	0.151	0.42	−133	−39.0	94.2
**35**	0.169	0.45	−142	−38.7	103
**46**	0.111	0.43	−97.6	−39.7	57.8
**46**	0.100	0.44	−106	−40.0	66.3

10. Virtual Synthesis

Table S6. Vendor catalogues used for chemical space generation and resulting 5’-aza adenosine amides and sulfonamides. ZINC annotations BB: building blocks, BBE: building blocks economical.

Supplier	Catalogue annotation	Size		
Alfa-Aesar[a]	BB	35,369		
Apollo Scientific	BB	24,958		
BLDpharm	BB	86,911		
ChemDiv[a]	BB	34,656		
ChemDiv	BBE[c]	14,684		
ChemSpace	BB	712,559		
Enamine[a]	BB	1,147,314		
Enamine	BBE[c]	83,469		
InterBioScreen	BB	13,248		
LifeChemicals[a]	BB	593		
LifeChemicals	BBE[c]	1017		
KeyOrganics	BB	221,022		
LabNetwork	BB	320,764		
Molport[a]	BB	85,912		
MolPort	BBE[c]	98,135		
OTAVAchemicals	BB	35,328		
SigmaAldrich	BB	117,226		
SPECS	BB	9,013		
TimTec	BB	55,702		
Vitas-M	BB	24,266		
WuXi Apptec	BB	7256		
		**version 1**	**version 2**	**version 2 economical[c]**
**all, filtered, unique**	(no alcohols, no amines SlogP < 3.6)	84,035	204,936	23,333
	• acids	76,678	189,306	21,502
	• acid chlorides	2729	3868	752
	• sulfonyl chlorides	4628	11,762	1079
**products**	(not reactive, no PAINS, RO5 violations ≤ 1 or 2,[b] SlogP > -1.8)	27,764	110,128 [b]	14,228[b]
	• amides	24,471	100,775	13,372
	• sulfonamides	2294	9353	856

[a] Libraries were added to the second iteration of the chemical space. [b] Up to two RO5-violations were tolerated in the second iteration of the chemical space. [c] The “economical” version of the chemical space was generated only from catalogues with the BBE annotation.

11. Predicted Physicochemical Properties of Chemical Spaces

Table S7. Predicted physicochemical properties of chemical space version 1 summarizing covered area of the space.

space	parameter	area	mean
	logD[3]	−3.9–4.1	−0.4
	SlogP[1]	−1.8–3.1	0
	HBD	4–7	4
version 1	HBA	7–12	8
	TPSA[2]	148.4–257.1 A^2	167.6 A^2
	molecular weight	308.2–748.1 g mol^{-1}	452.1 g mol^{-1}
	rot. bonds	4–17	7
	logD[3]	−5.5–5.8	−0.4
	SlogP[1]	−1.8–4.6	0.1
	HBD	4–10	4
version 2	HBA	7–42	8
	TPSA[2]	148.4–489.9	175.2
	molecular weight	308.3–1938.3	482.0
	rot. bonds	4–115	8
	logD[3]	−4.9–3.7	−0.2
	SlogP[1]	−1.8–2.9	0
	HBD	4–7	4
version 2eco	HBA	7–15	8
	TPSA[2]	148.4–267.5 A^2	174.2 A^2
	molecular weight	308.3–779.8 g mol^{-1}	477.3 g mol^{-1}
	rot. bonds	4–27	7

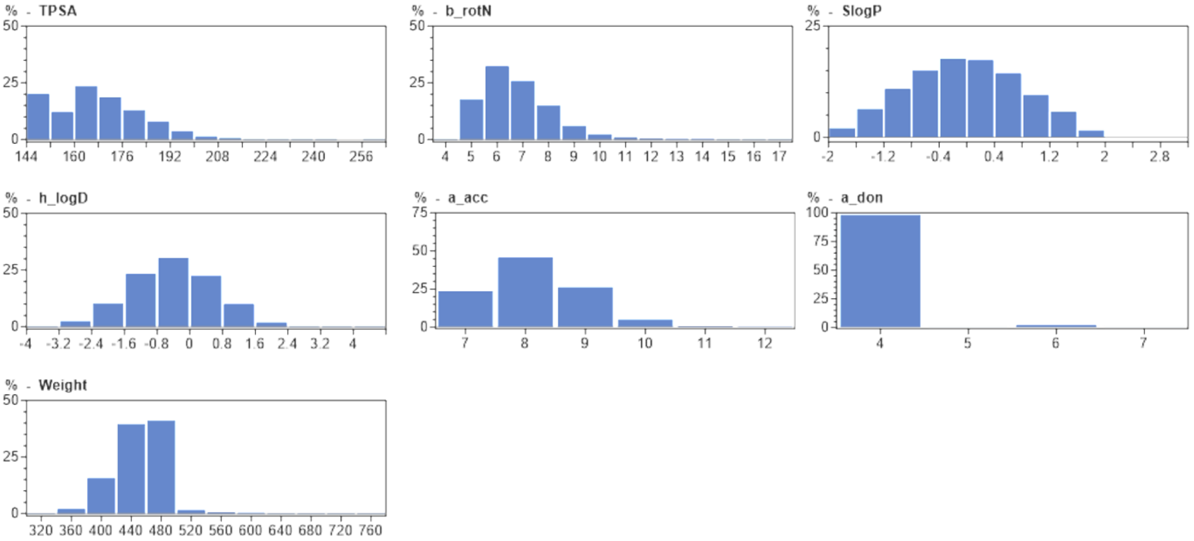

Figure S18. Physicochemical properties (TPSA, rotatable bonds (b_rotN), SlogP, h_logD, HBA (a_acc), HBD (a_don) and molecular weight (Weight) of space 1.

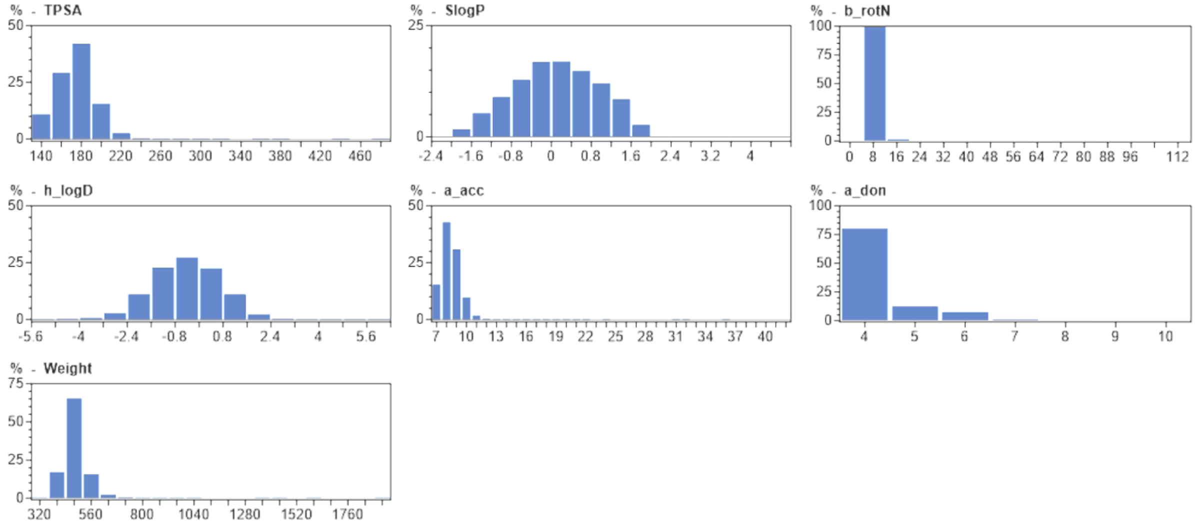

Figure S19. Physicochemical properties (TPSA, rotatable bonds (b_rotN), SlogP, h_logD, HBA (a_acc), HBD (a_don) and molecular weight (Weight) of space 2.

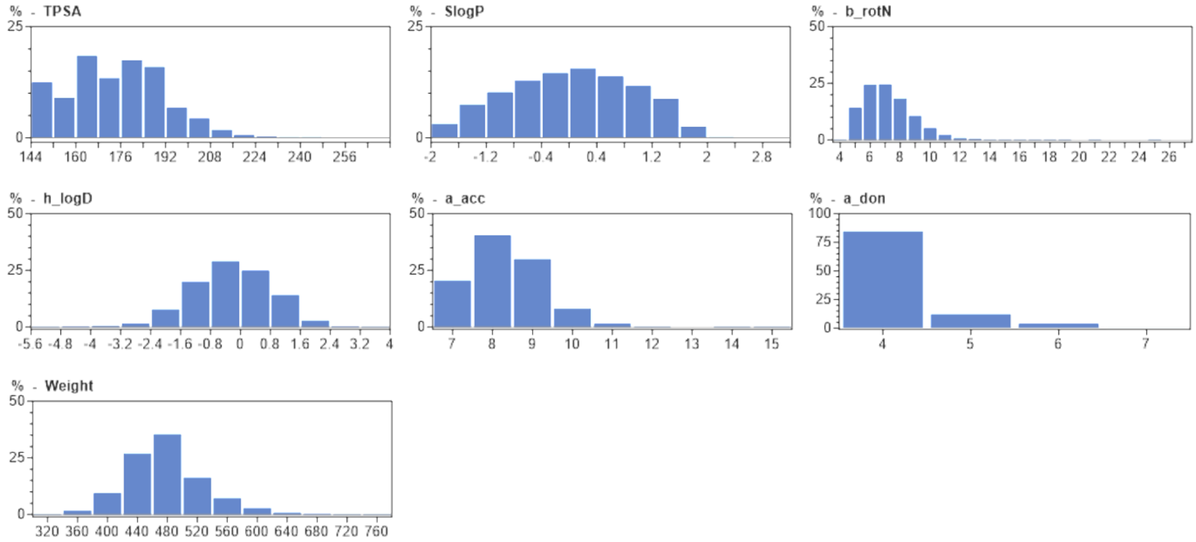

Figure S20. Physicochemical properties (TPSA, rotatable bonds (b_rotN), SlogP, h_logD, HBA (a_acc), HBD (a_don) and molecular weight (Weight) of space 2eco.

12. Molecular docking

A

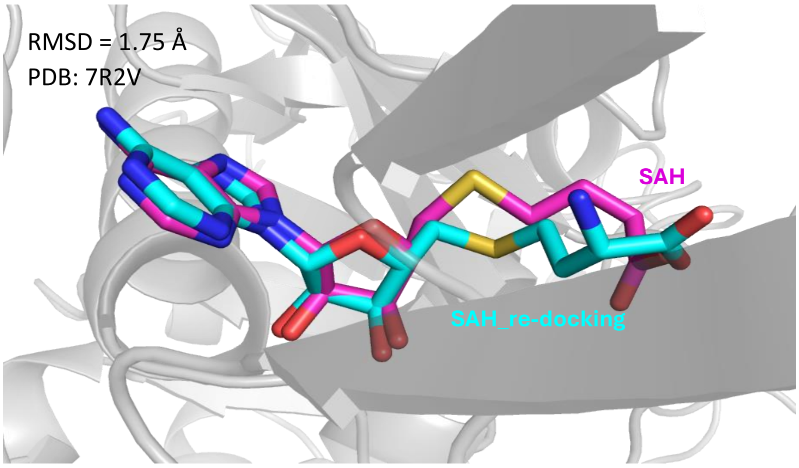

B

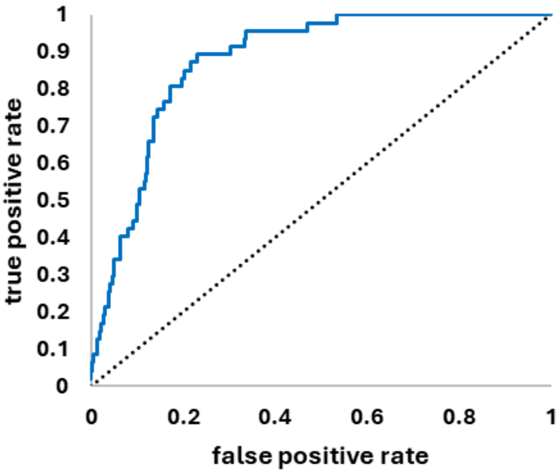

C

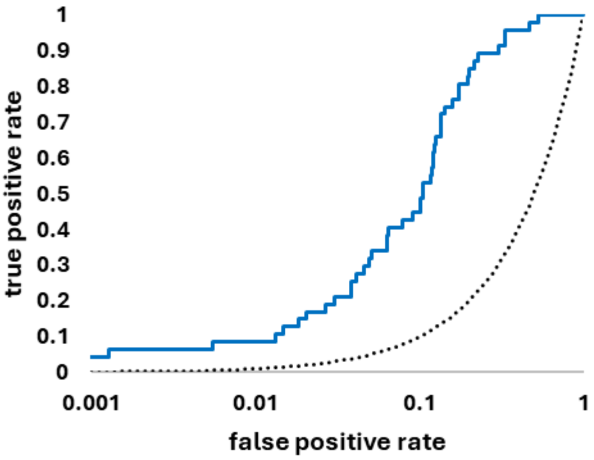

Figure S21 Molecular docking validation. **A**) Re-docking of SAH (RMSD = 1.75 Å, PDB-ID: 7R2V, crystallographic reference ligand SAH is shown with magenta-colored carbon atoms, docking pose with cyan carbon atoms, SARS-CoV-2 nsp14/10 in grey PDB: 7R2V). **B**)Receiver-operating characteristics curve area under the curve (ROC AUC) analysis for the discrimination of 47 binders from 10,182 property-matched decoys (ROC-AUC: 0.88). Perfect discrimination corresponds to a ROC-AUC of 1.00, random distribution to 0.50. **C**) Early enrichment analysis by corrected logarithmic ROC-AUC analysis (ROC-log$_{0.1\text{-}100\%}$ AUC: 0.25). Perfect discrimination corresponds to a ROC-logAUC auf 0.85, random distribution to 0.00.

Table S8. Medoids and outlier structures of spaces version 1, 2 and 2eco derived from similarity analysis using the iSIM method with RDKit fingerprints.

space	Medoids	Outlier
**version 1**	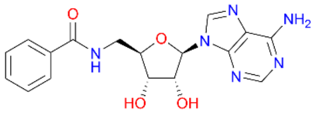	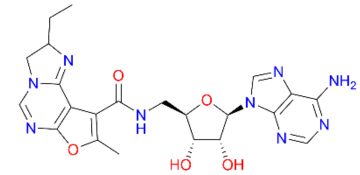
**version 2**	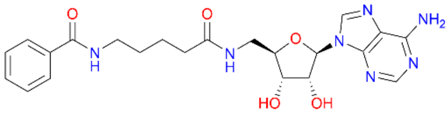	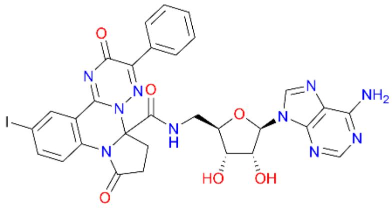
**version 2 eco**	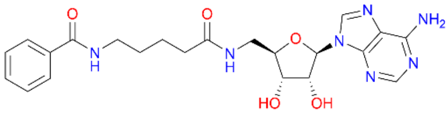	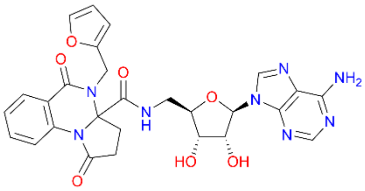

13. Table of key spectroscopic features

Table S9. Chemical shifts of ^{1}H and ^{13}C, splitting and integrals of adenosine-scaffold prevalent in all compounds. Shifts are exemplary of compound **4**.

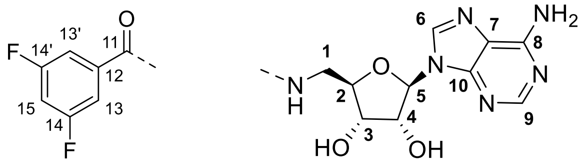

Number	Chemical shifts		Splitting[a]	Integral
	^{1}H (ppm)	^{13}C (ppm)		
1	3.81–3.62	42.7	m	2 H
2	5.97	91.0	d	1 H
3	4.72	75.2	t	1 H
4	4.31	72.8	t	1 H
5	4.22–4.14	84.9	m	1 H
6	8.42	146.1	s	1 H
7	-	120.8	-	-
8	-	152.4	-	-
9	8.19	144.3	s	1 H
10	-	149.9	-	-
NH	-[b]	-	-	1 H
NH_2	-[b]	-	-	2 H
C=O (11)	-	167.6	-	-
12	-	139.0	-	1 H
13/13’		111.5	-	1 H
14/14’	-	165.0/162.7	-	-
15	107.	107.8	-	1 H

[a] m = multiplett, t = triplett, s = singulet of ^{1}H spectra. [b] shifts are visible with DMSO-d_6. *Shifts can differ depending on the solvent used.

14. Spectra and Chromatograms

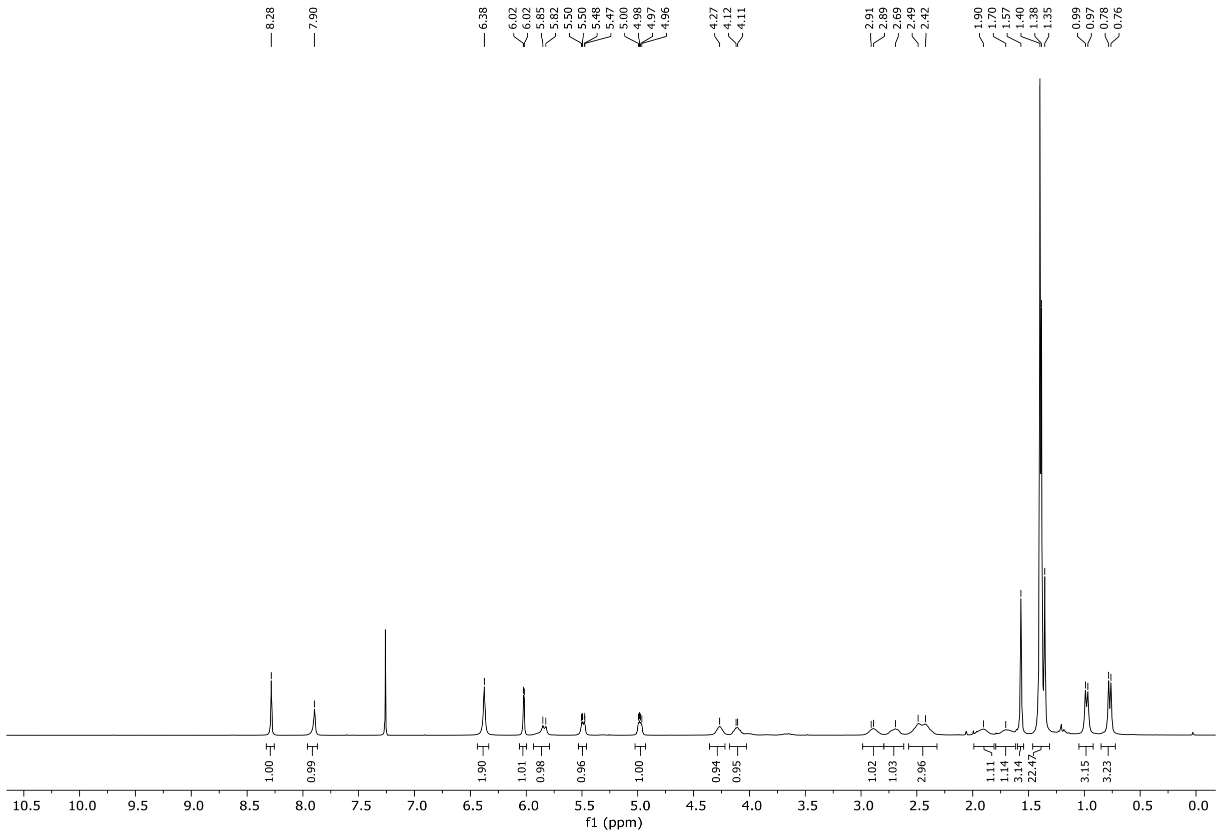

Figure S22: ^{1}H NMR of compound **3**.

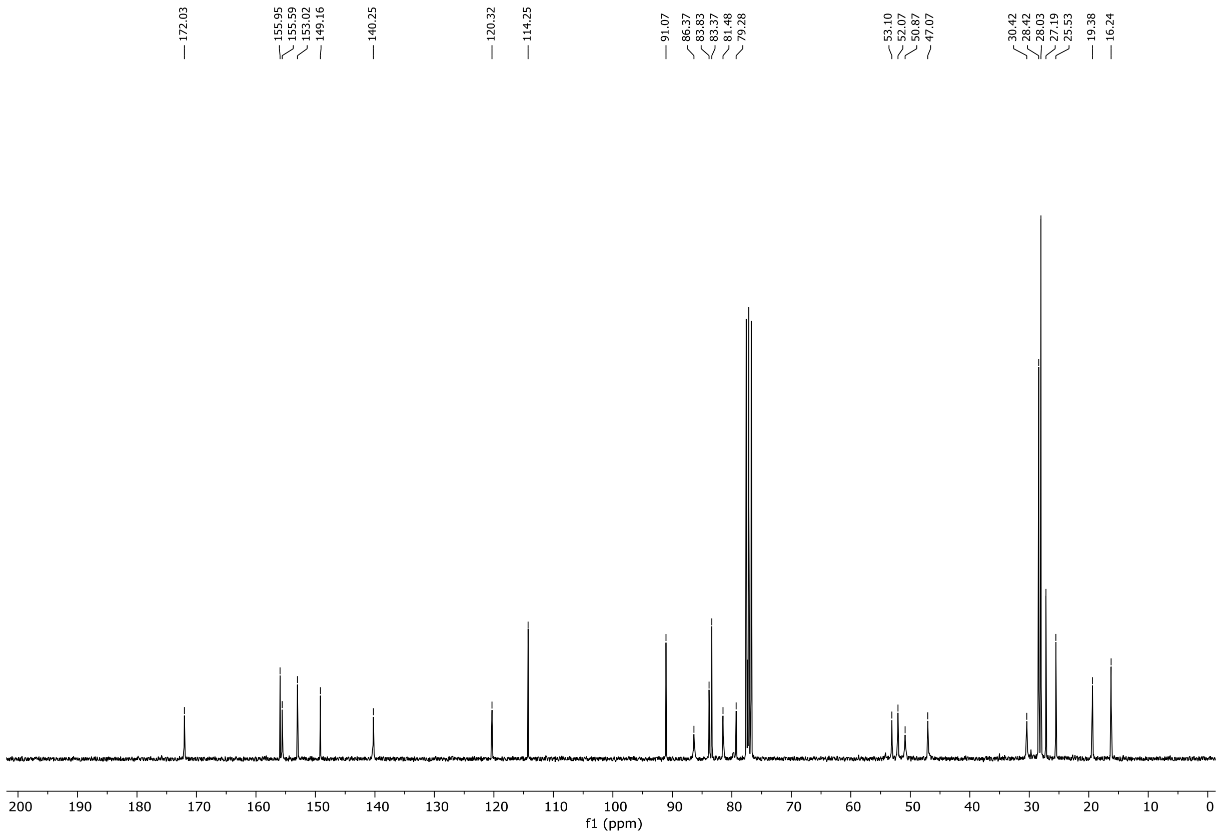

Figure S23. ^{13}C NMR of compound **3.**

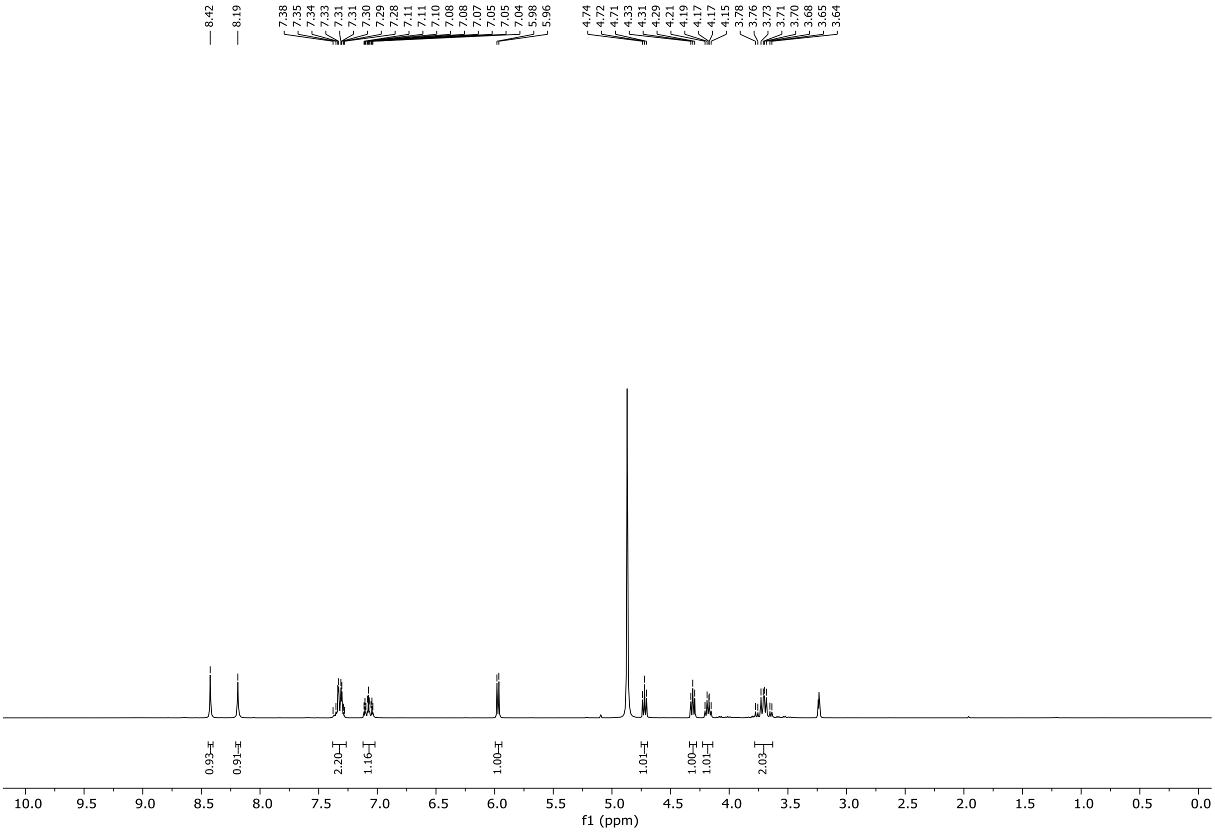

Figure S24. ^{1}H NMR of compound **4.**

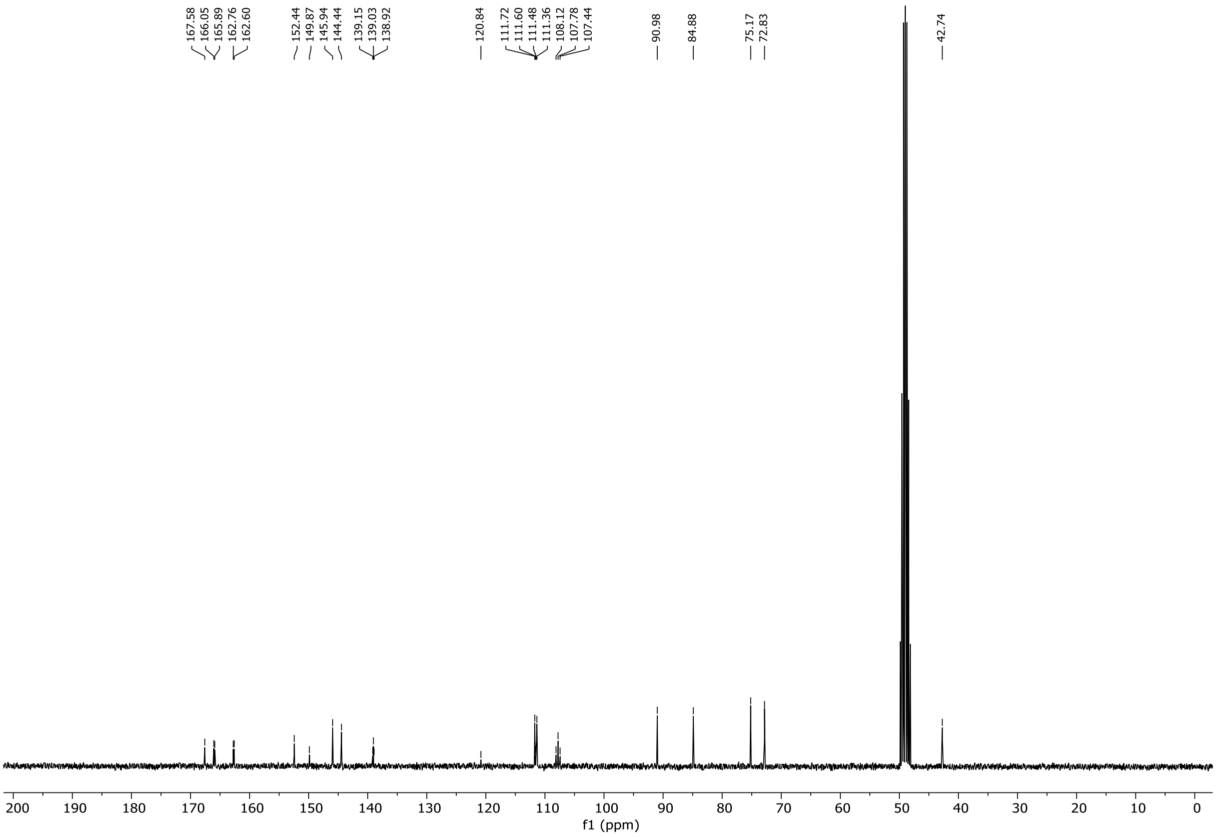

Figure S25. ^{13}C NMR of compound **4.**

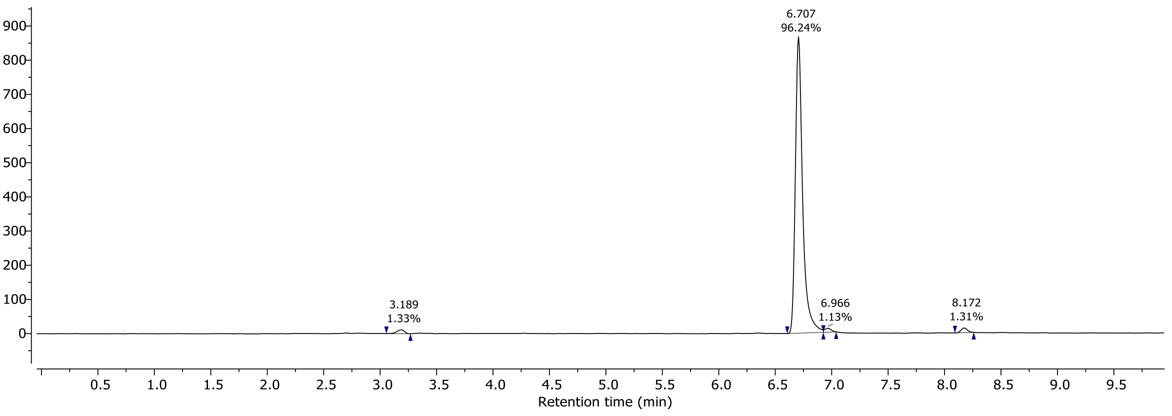

Figure S26. LCMS chromatogram of compound **4.**

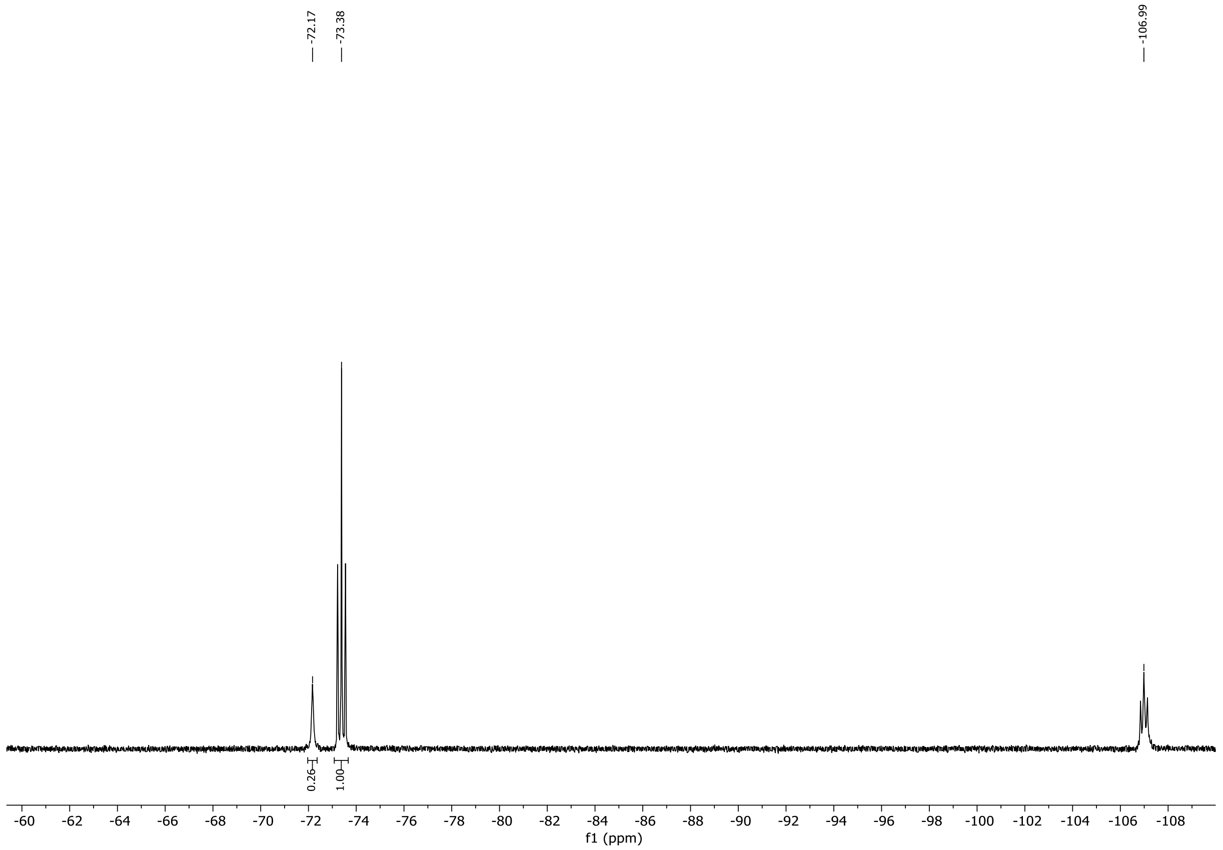

Figure S27. ^{19}F NMR of compound ***4.***

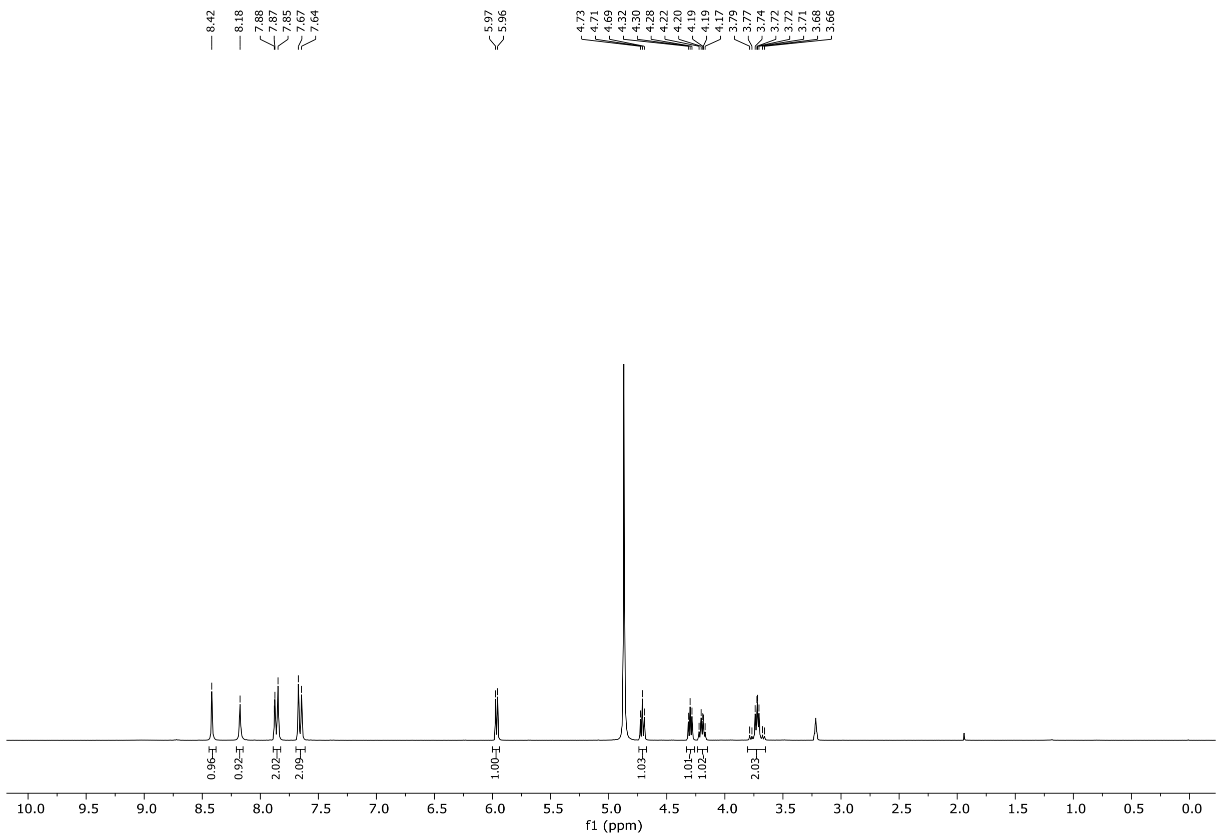

Figure S28. ^{1}H NMR of compound ***5***.

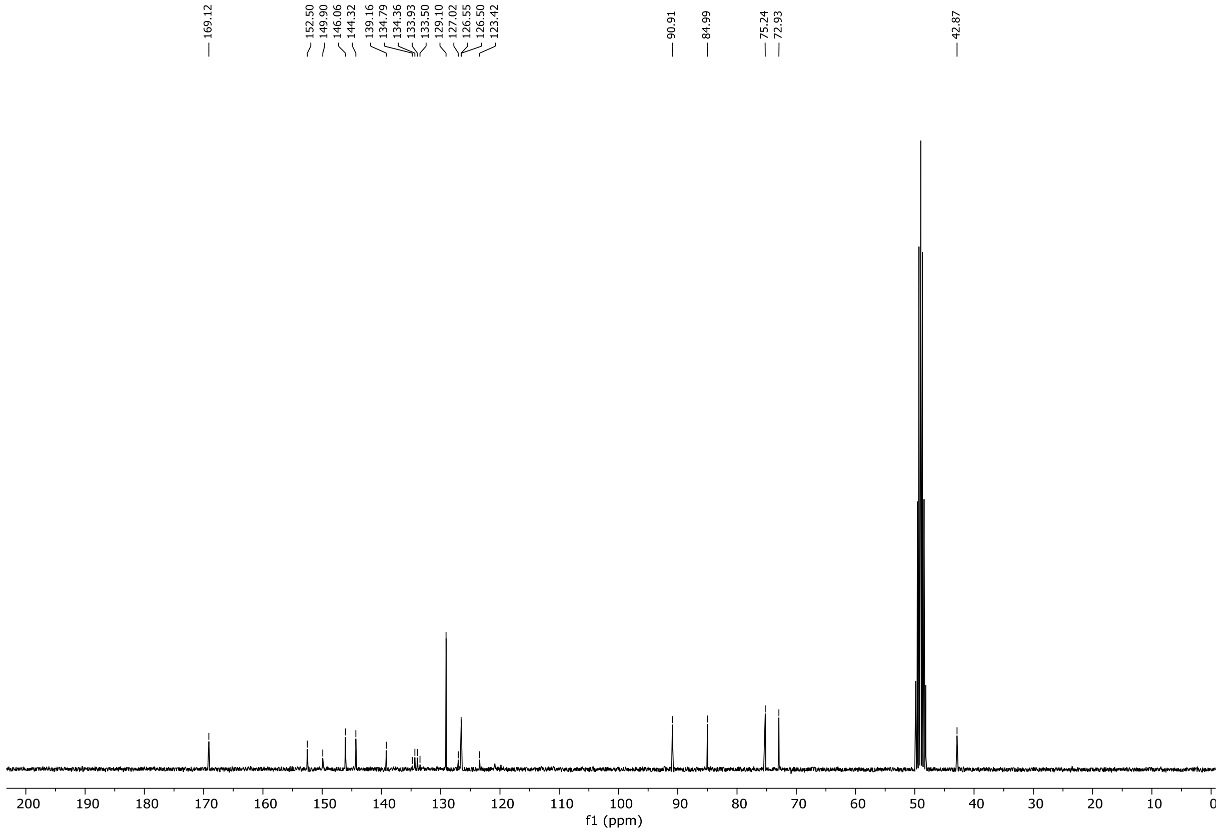

Figure S29. ^{13}C NMR of compound ***5***.

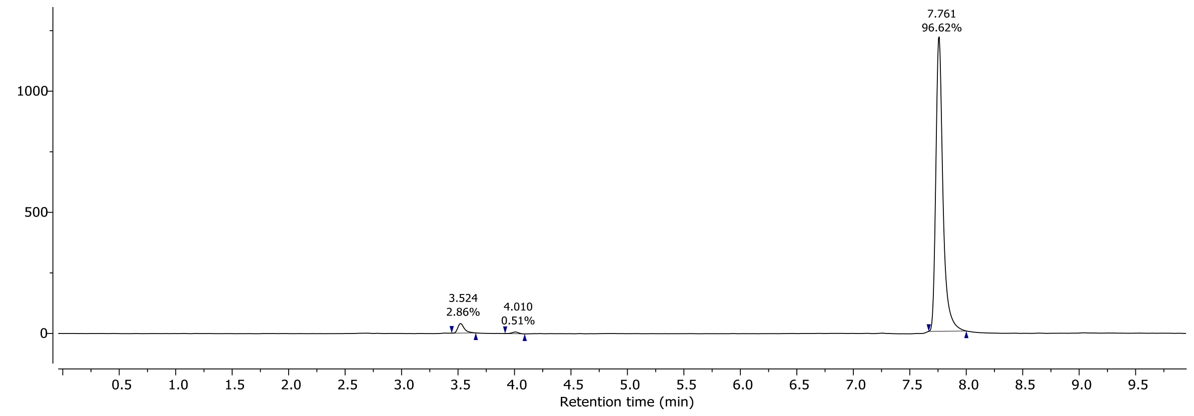

Figure S30. LCMS chromatogram of compound ***5*** at 254 nm.

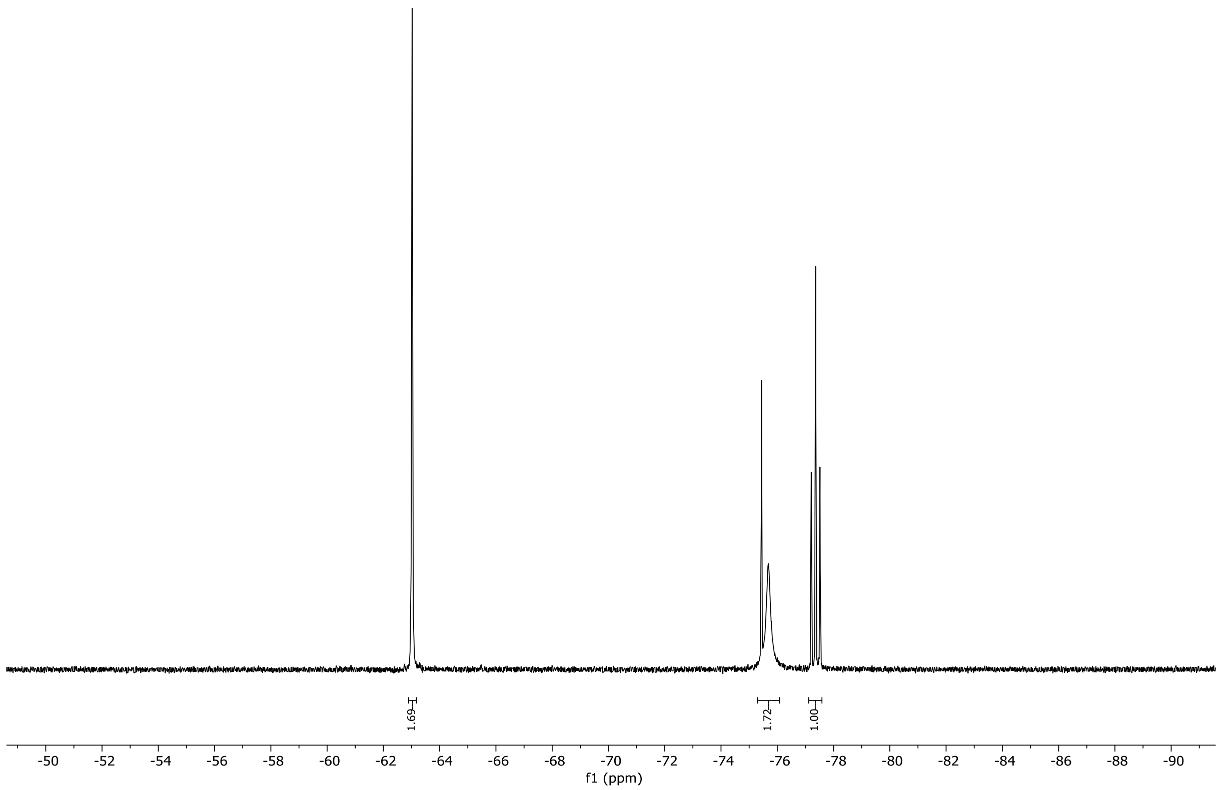

Figure S31. ^{19}F NMR of compound ***5*.**

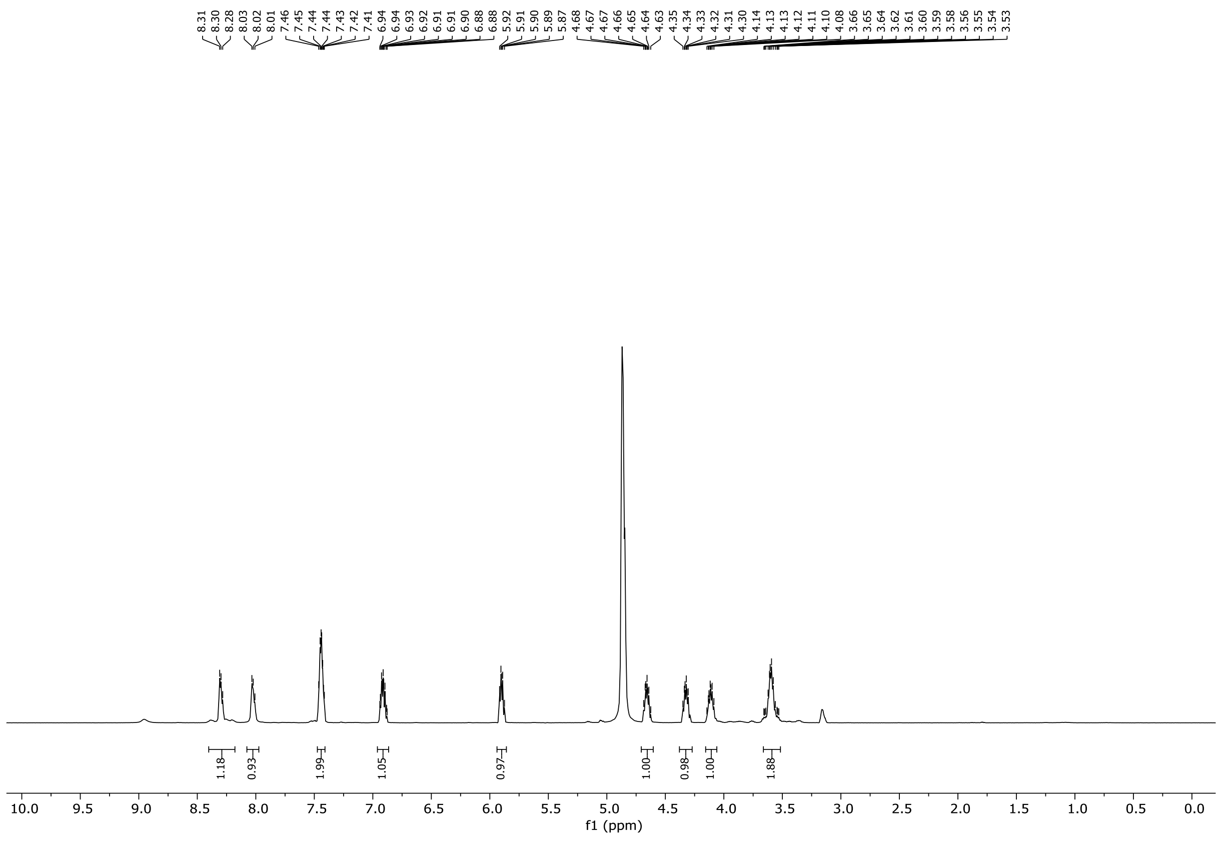

Figure S32. ^{1}H NMR of compound ***6***.

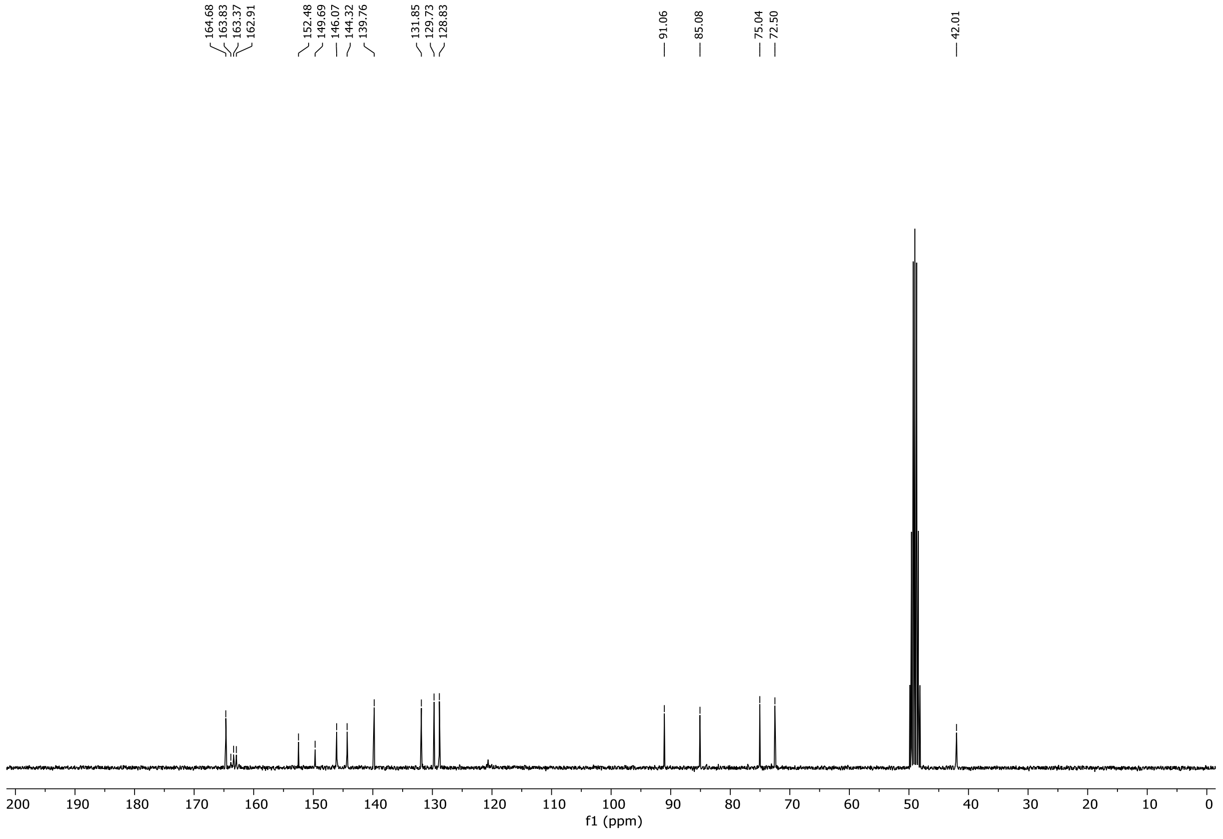

Figure S33. ^{13}C NMR of compound ***6***.

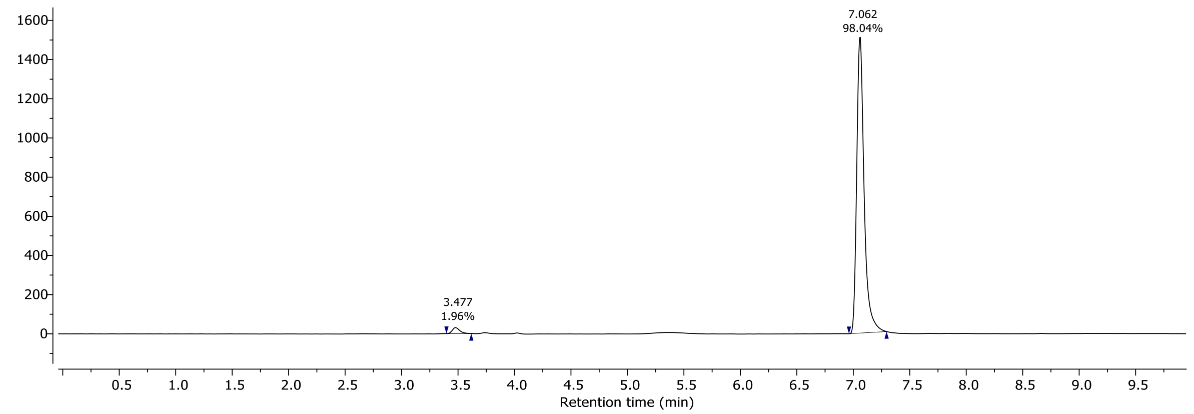

Figure S34. LCMS chromatogram of compound ***6*** at 254 nm.

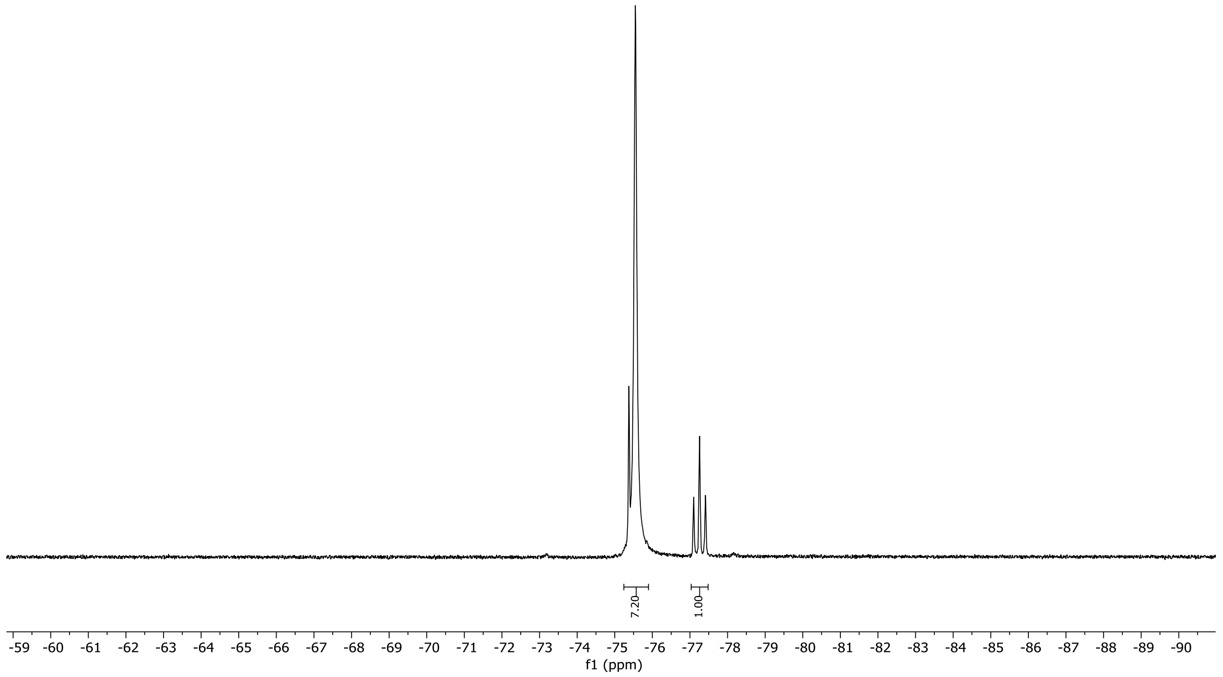

Figure S35. ^{19}F NMR of compound ***6*.**

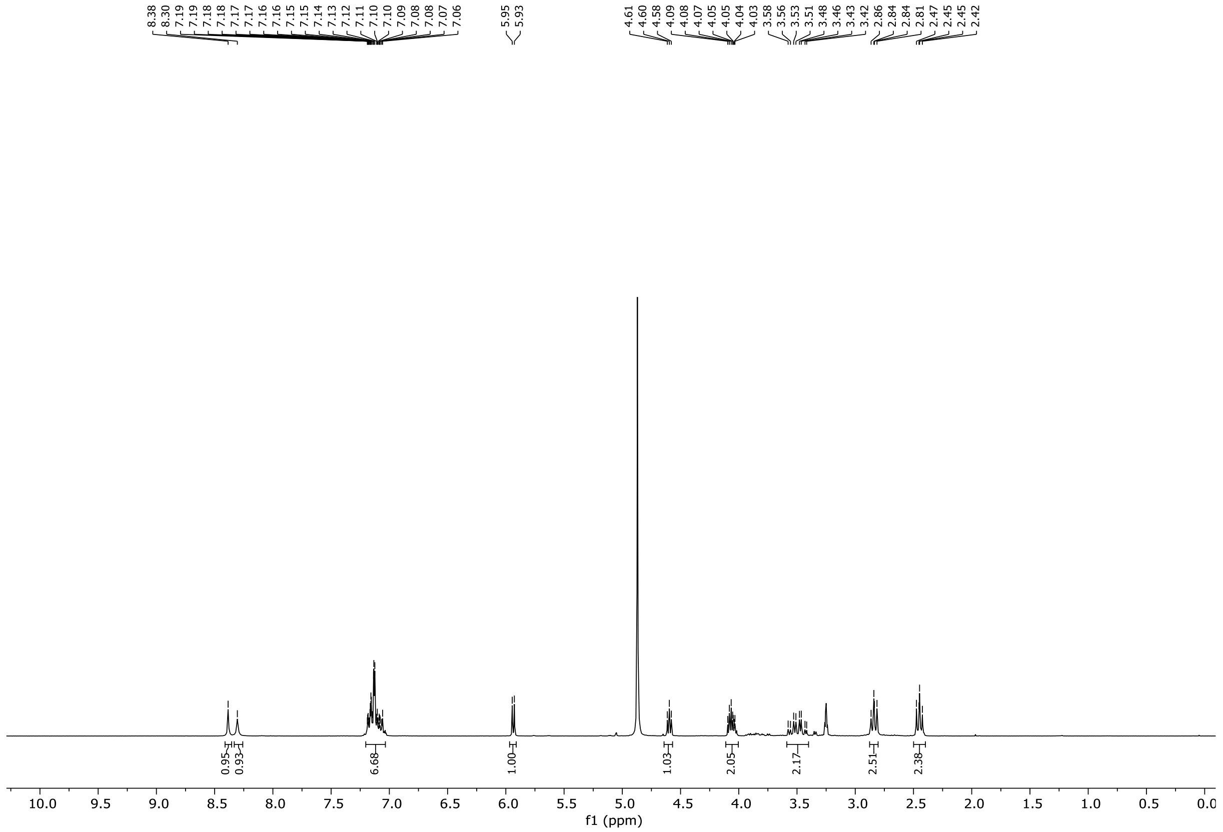

Figure S36. ^{1}H NMR of compound ***7.***

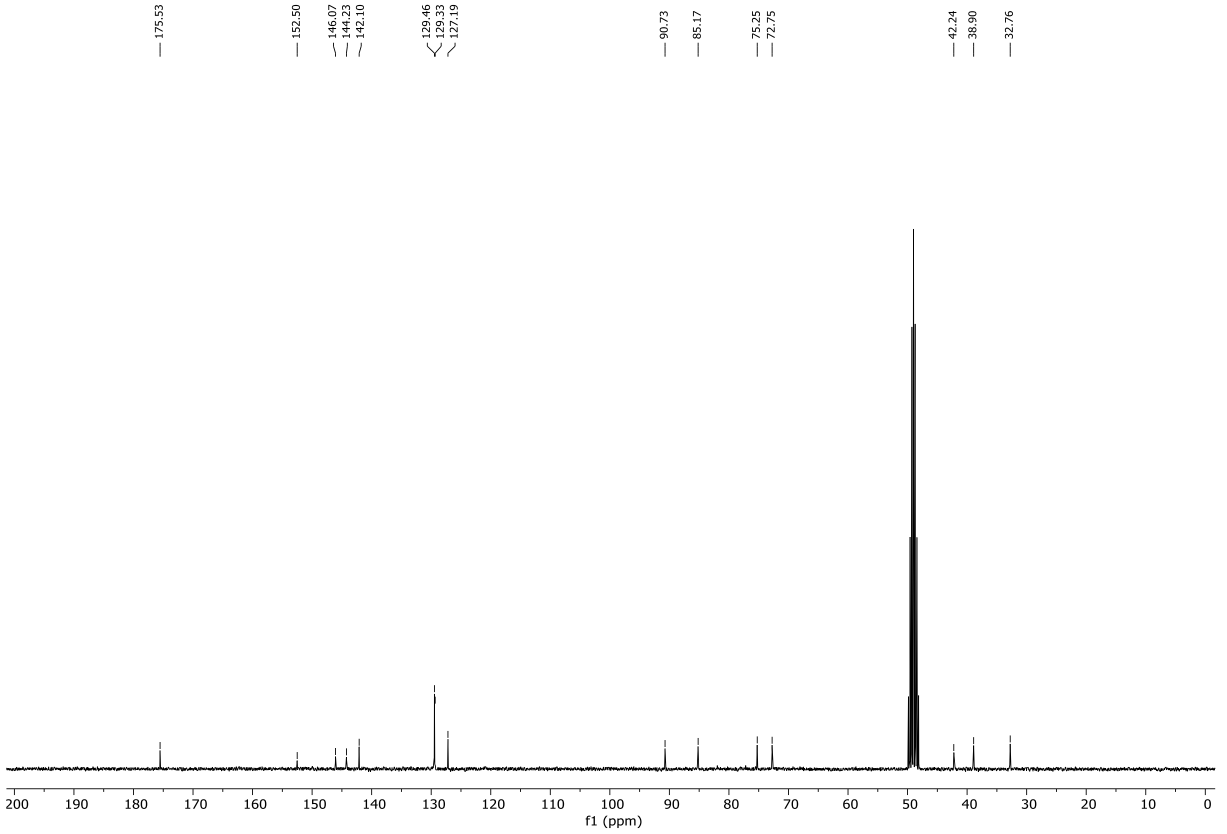

Figure S37. ^{13}C NMR of compound ***7***.

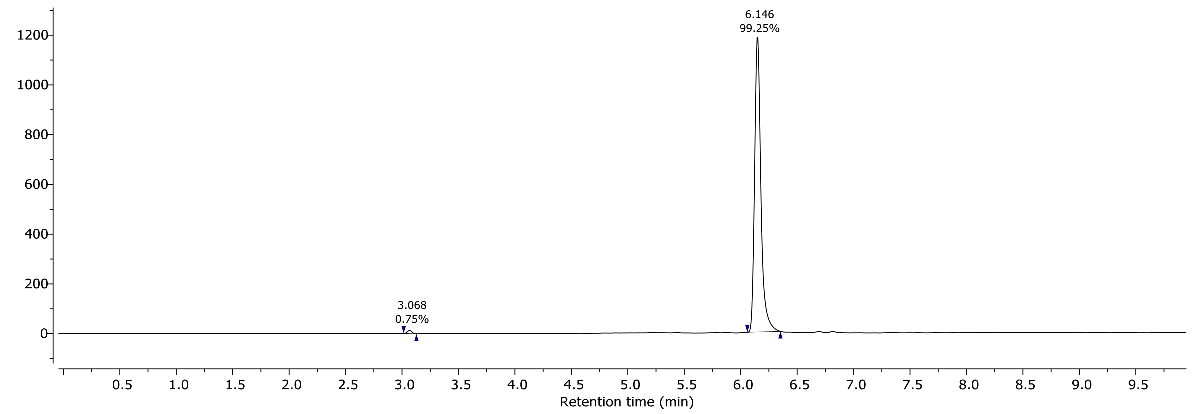

Figure S38. LCMS chromatogram of compound ***7*** at 254 nm.

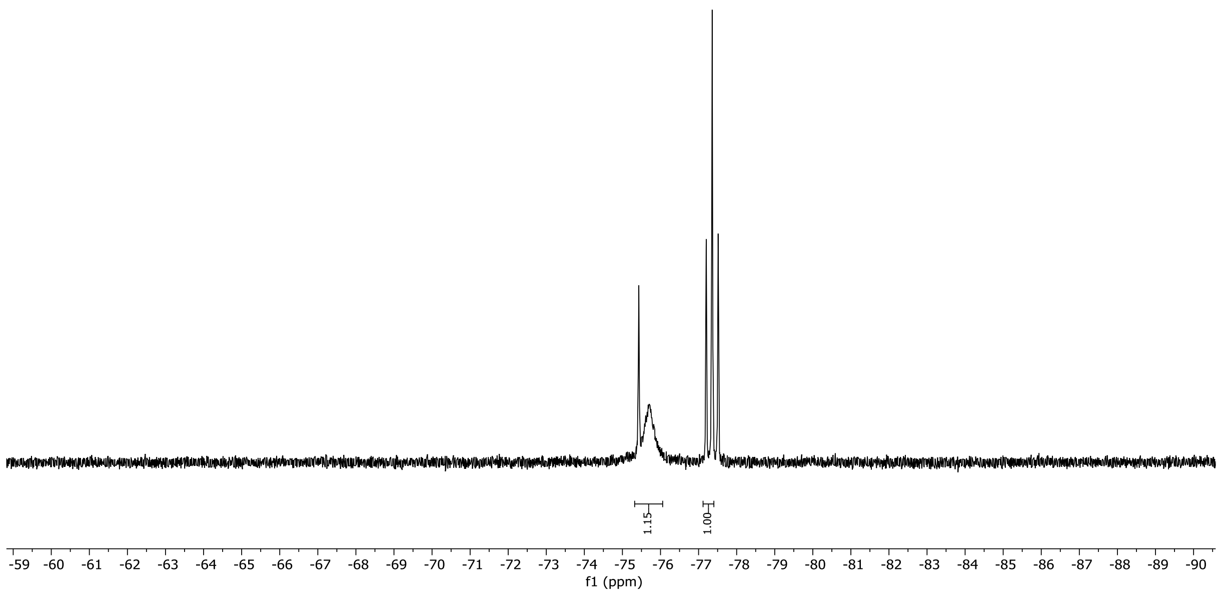

Figure S39. ^{19}F NMR of compound ***7***.

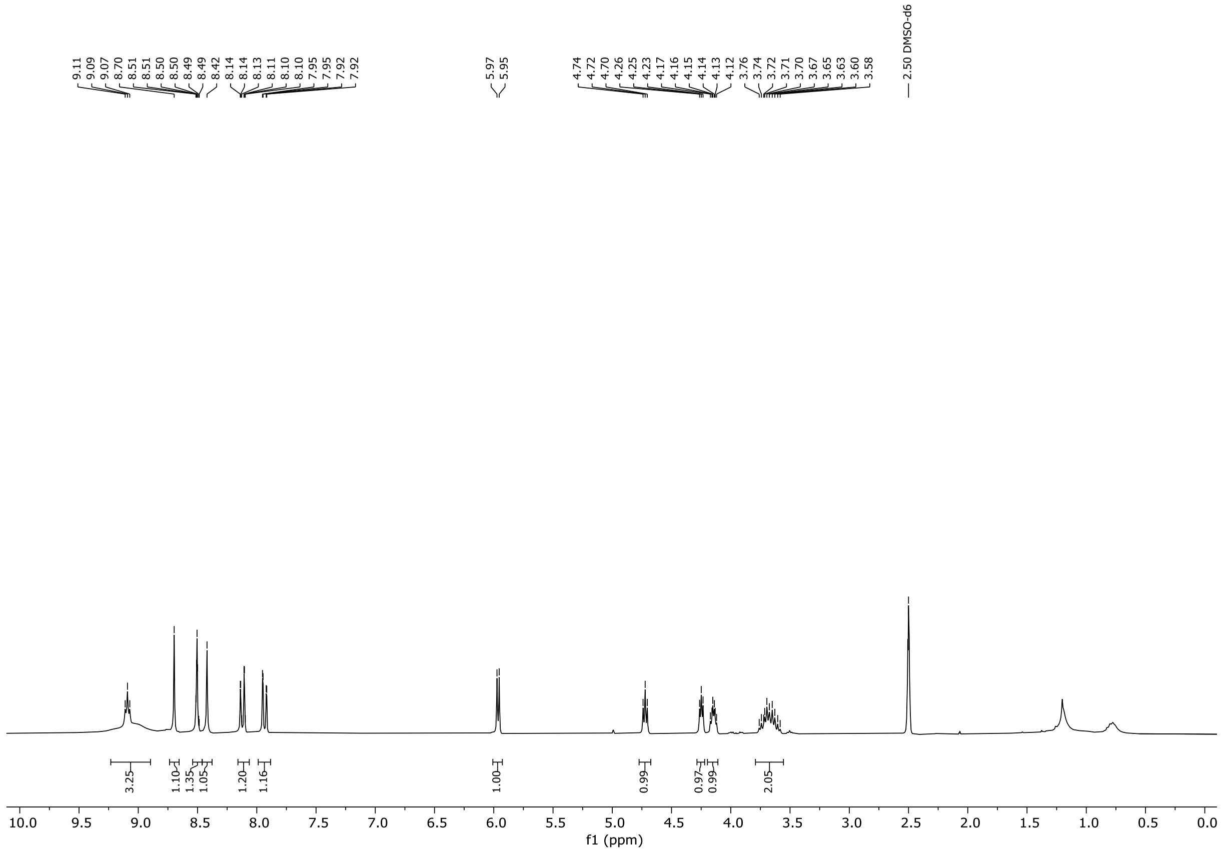

Figure S40. ^{1}H NMR of compound ***8***.

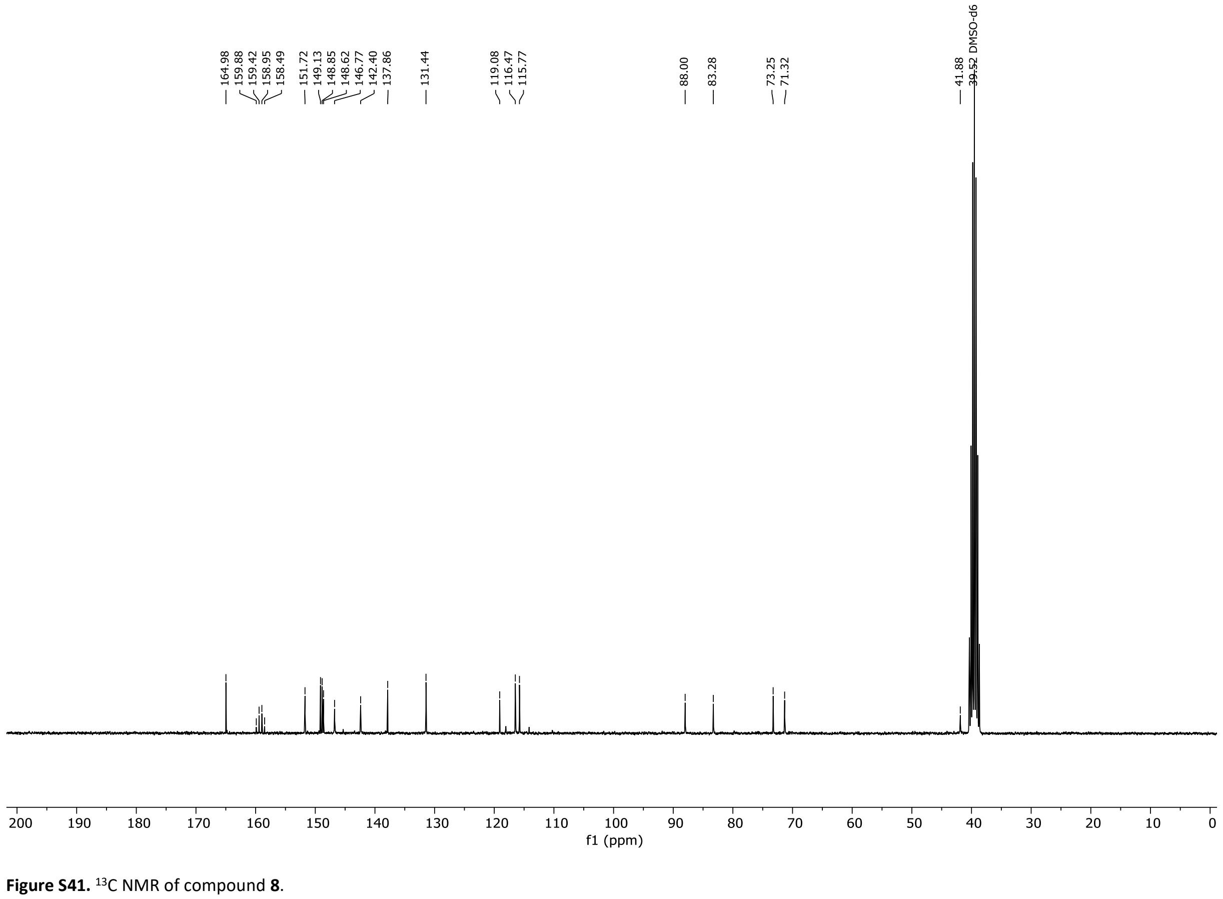

Figure S41. ^{13}C NMR of compound **8**.

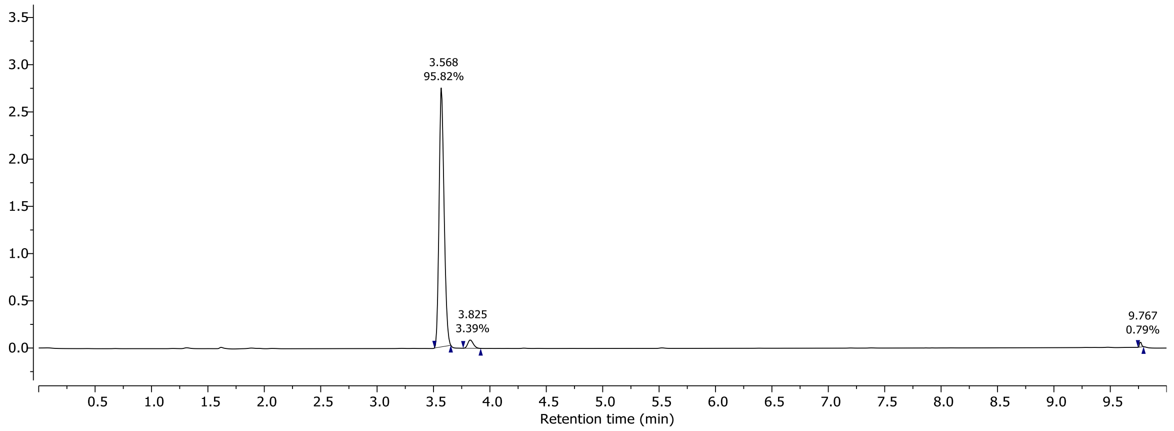

Figure S42. LCMS chromatogram of compound ***8*** at 254 nm.

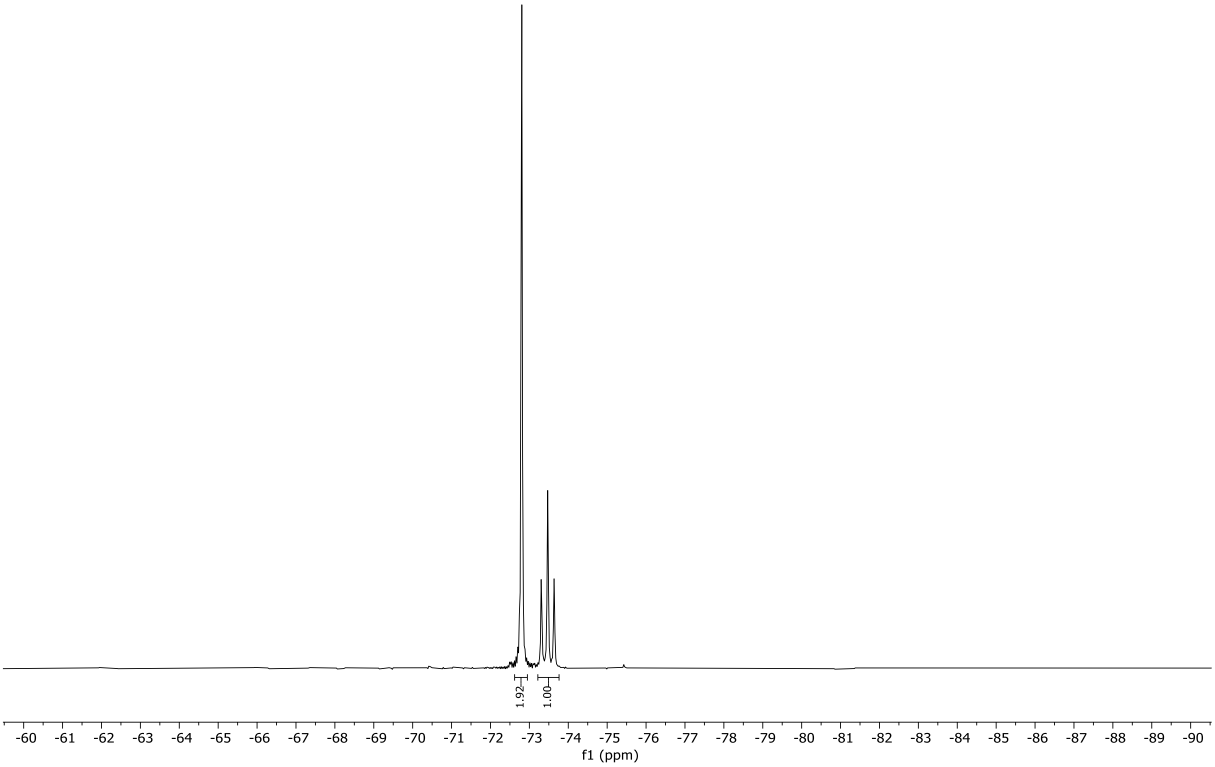

Figure S43. ^{19}F NMR of compound ***8***.

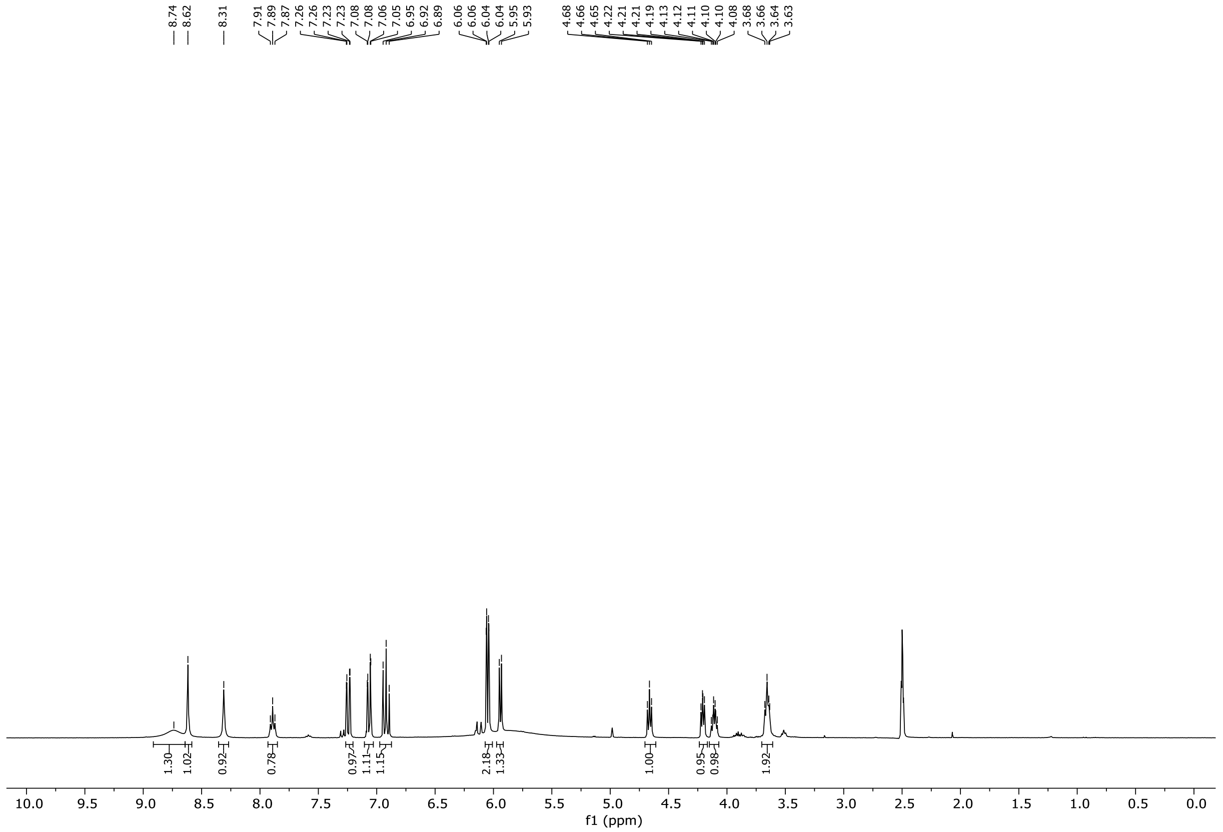

Figure S44. ^{1}H NMR of compound ***9***.

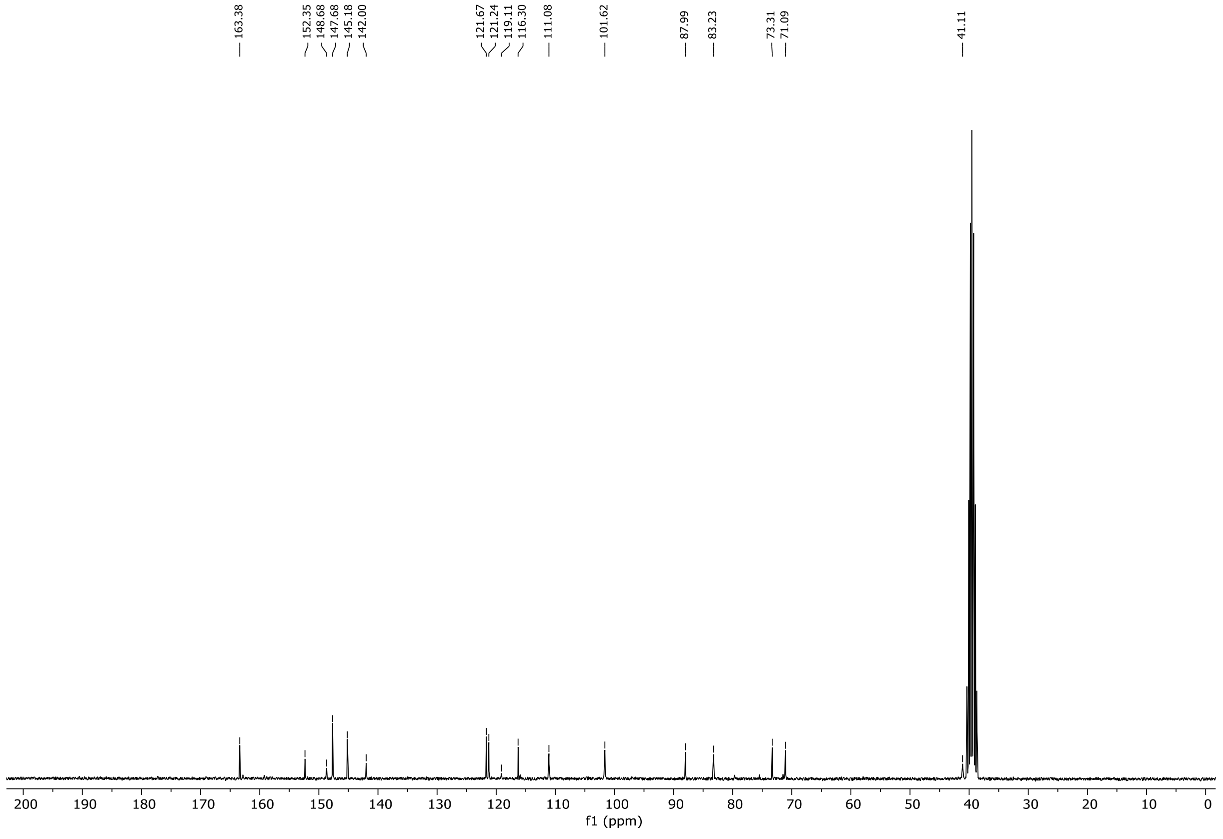

Figure S45. ^{13}C NMR of compound ***9***.

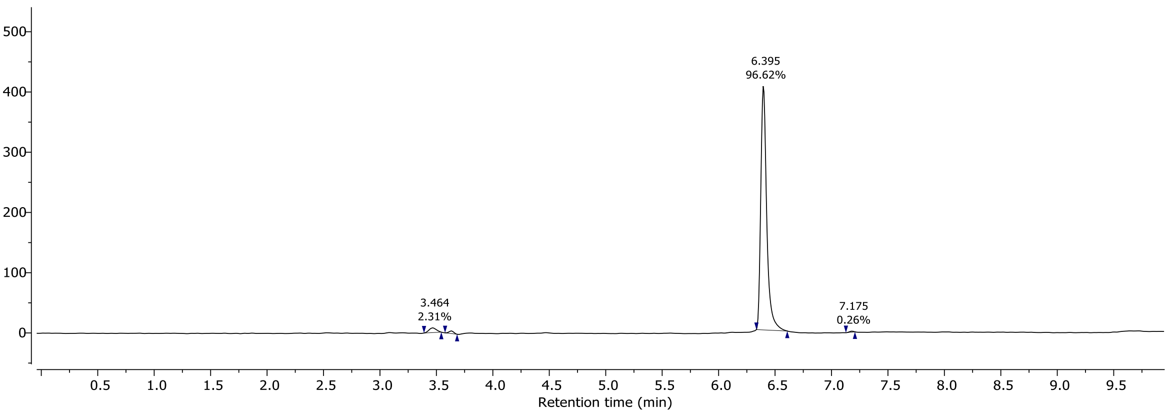

Figure S46. LCMS chromatogram of compound ***9*** at 254 nm.

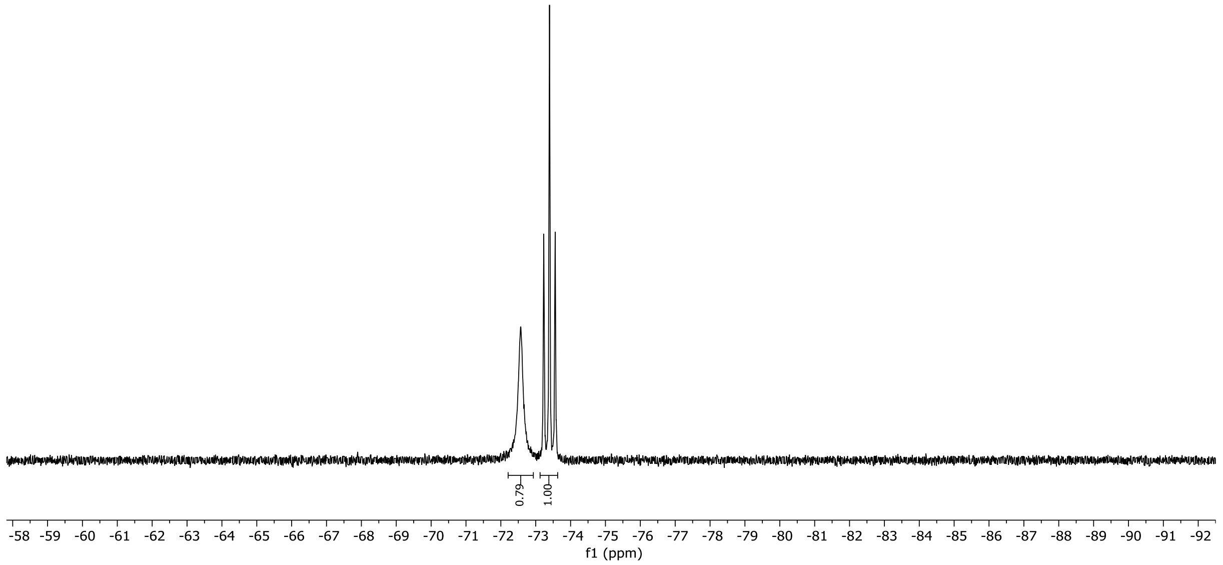

Figure S47. ^{19}F NMR of compound ***9***.

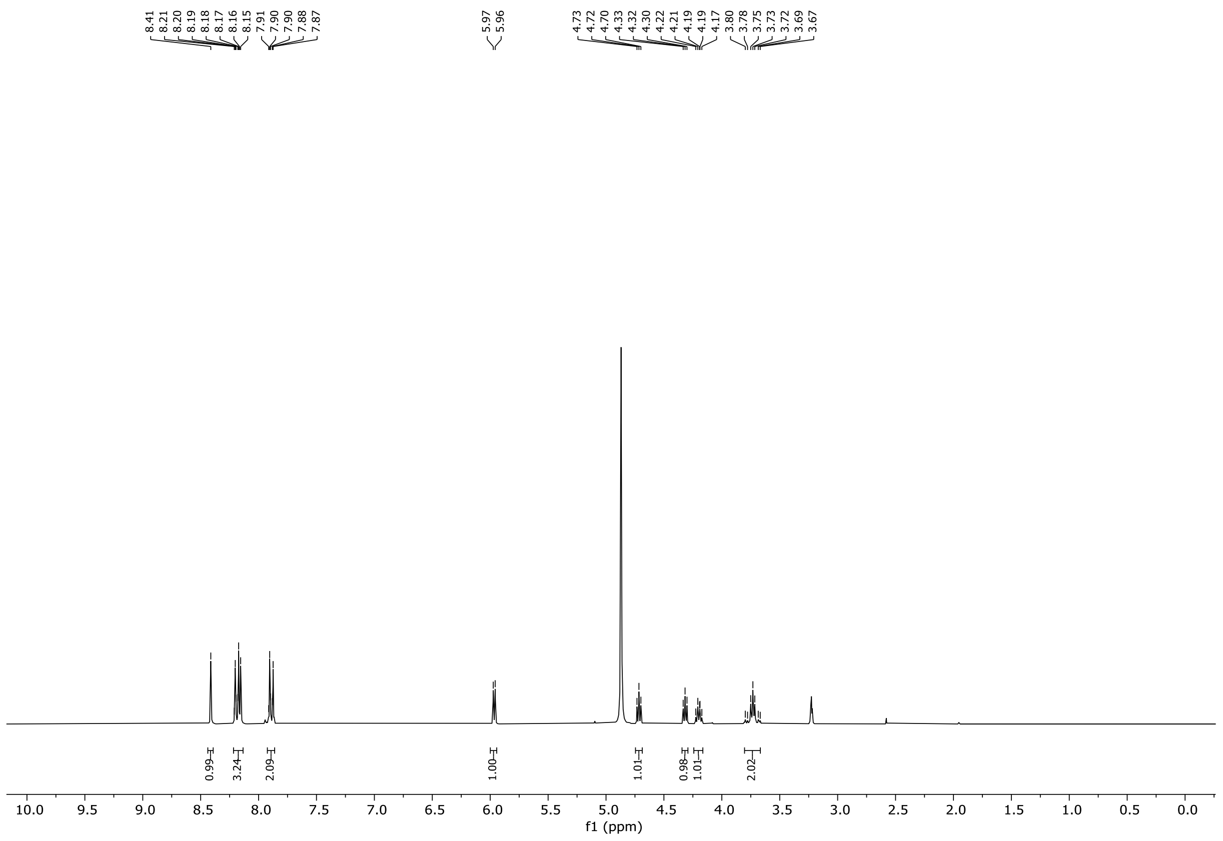

Figure S48. ¹H NMR of compound ***10.***

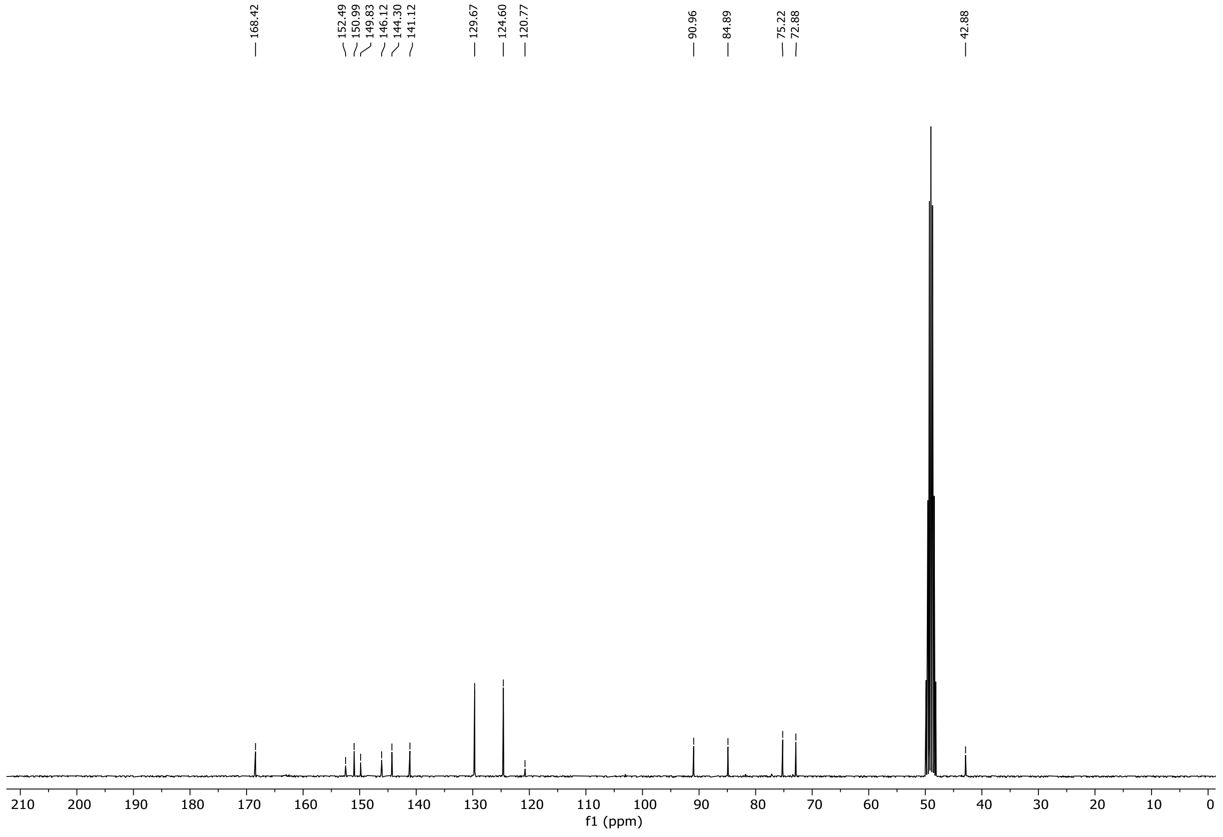

Figure S49. ¹³C NMR of compound ***10***.

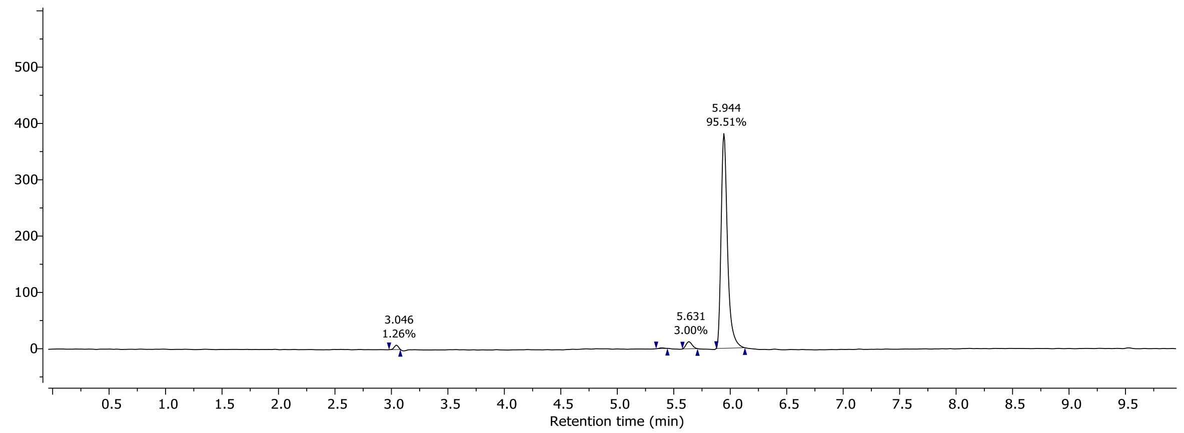

Figure S50. LCMS chromatogram of compound *10* at 254 nm.

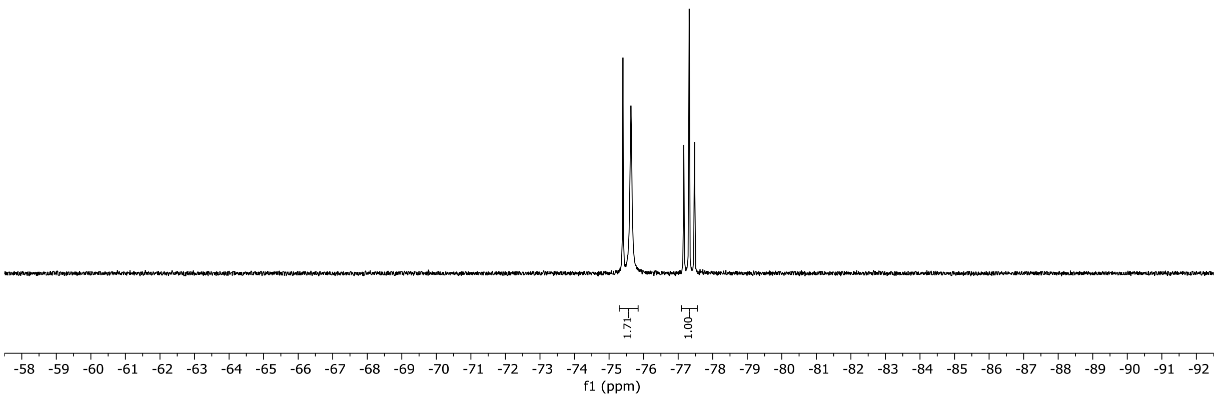

Figure S51. ^{19}F NMR of compound ***10***.

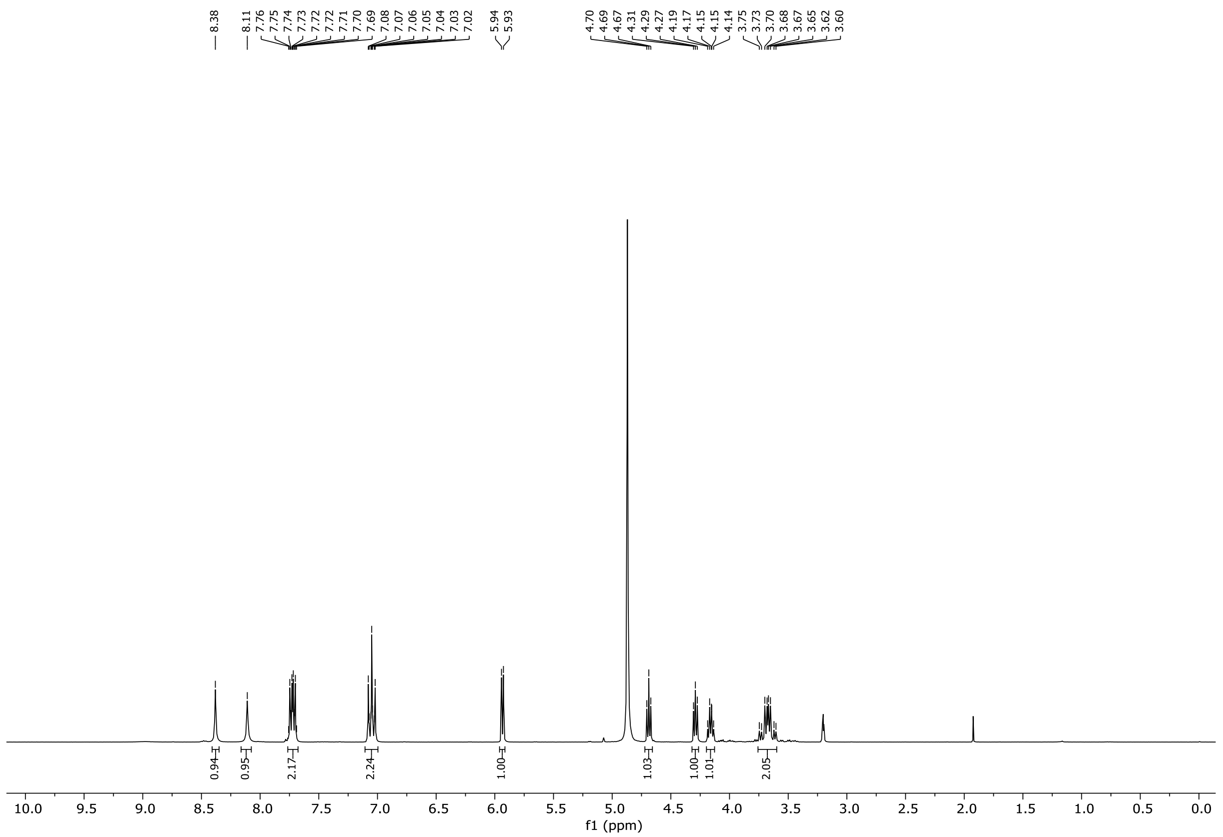

Figure S52. ^{1}H NMR of compound ***11***.

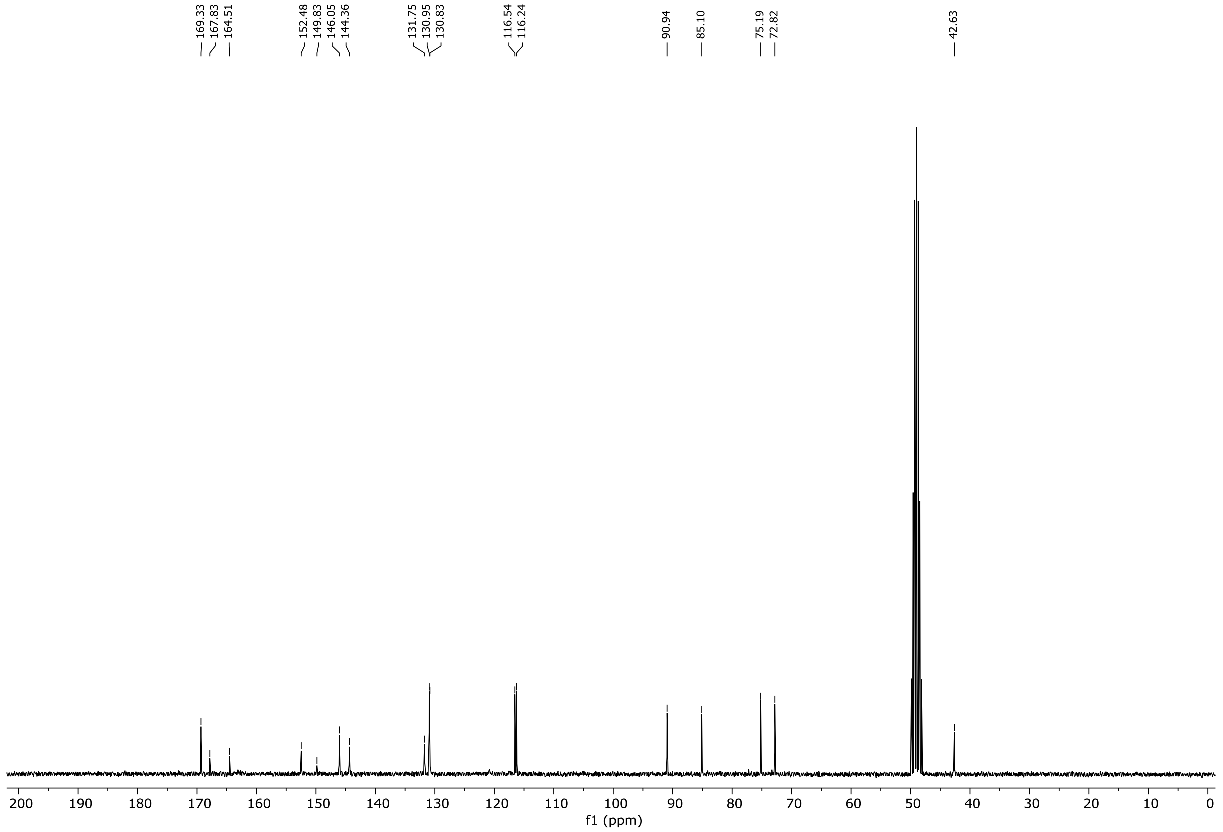

Figure S53. ^{13}C NMR of compound ***11***.

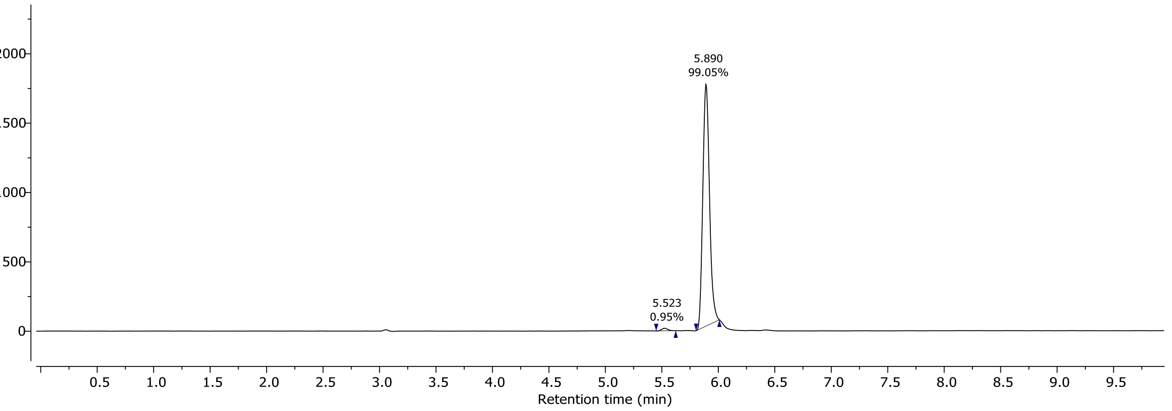

Figure S54. LCMS chromatogram of compound ***11*** at 254 nm.

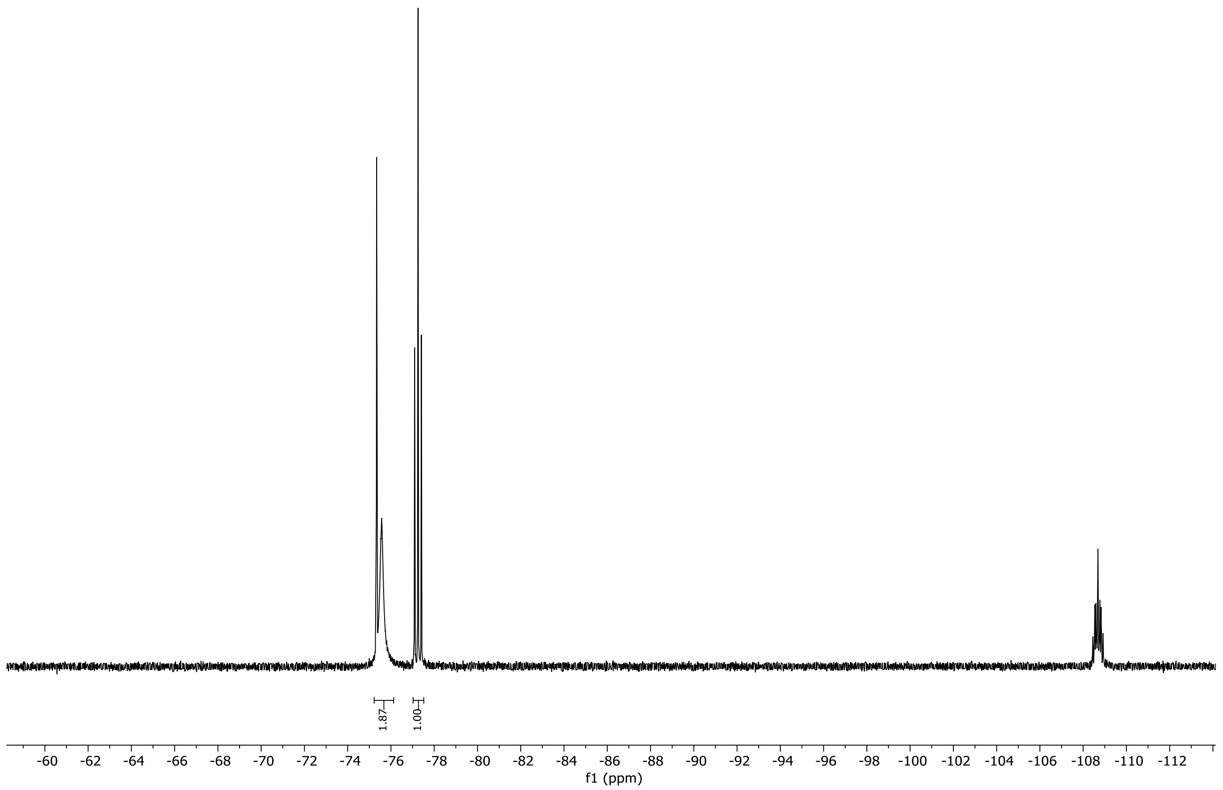

Figure S55. ^{19}F NMR of compound ***11***.

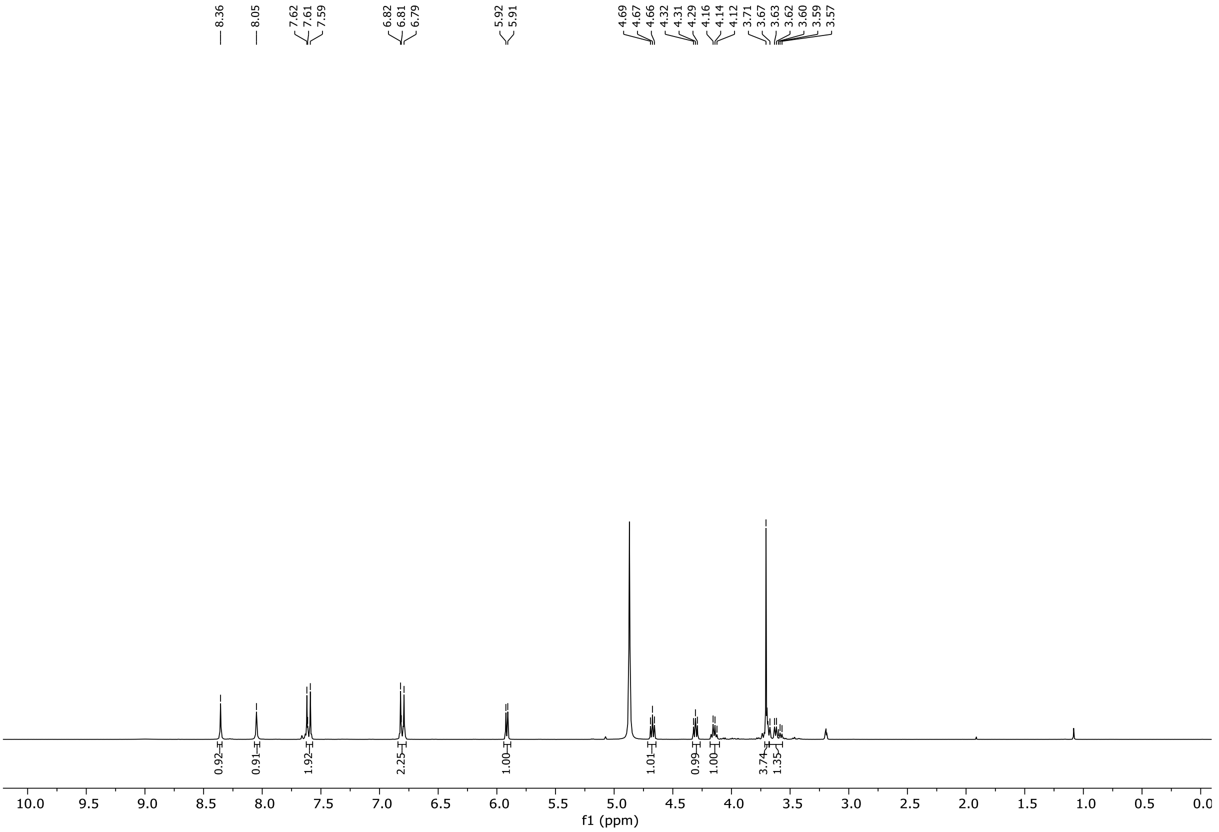

Figure S56. ^{1}H NMR of compound ***12***.

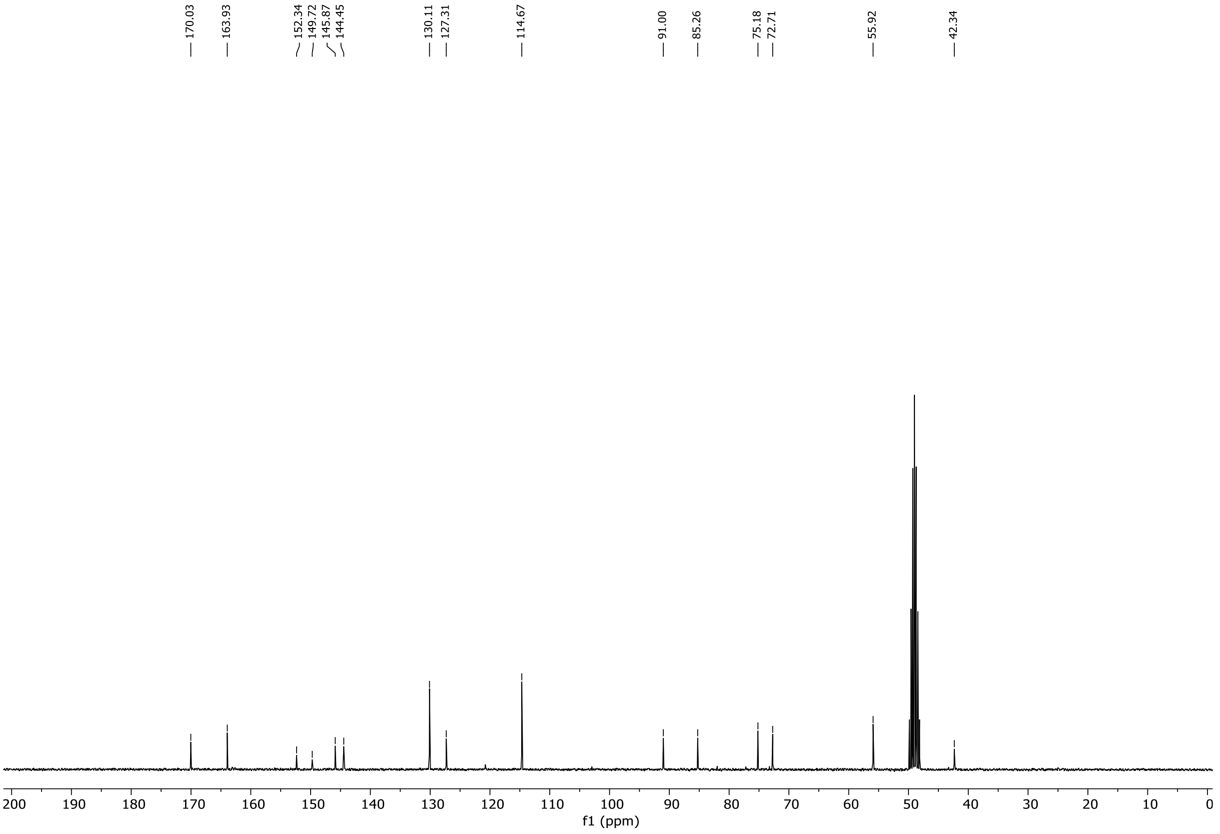

Figure S57. ^{13}C NMR of compound ***12***.

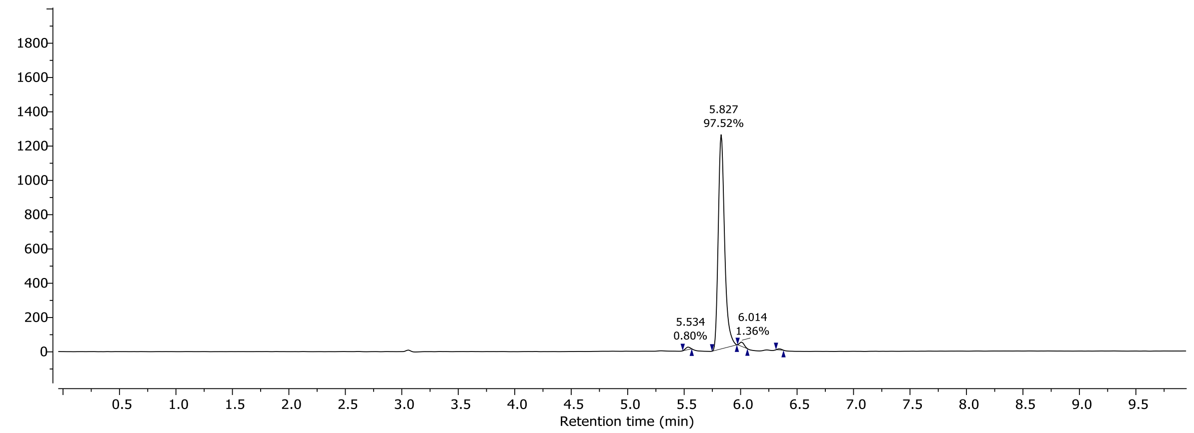

Figure S58. LCMS chromatogram of compound ***12*** at 254 nm.

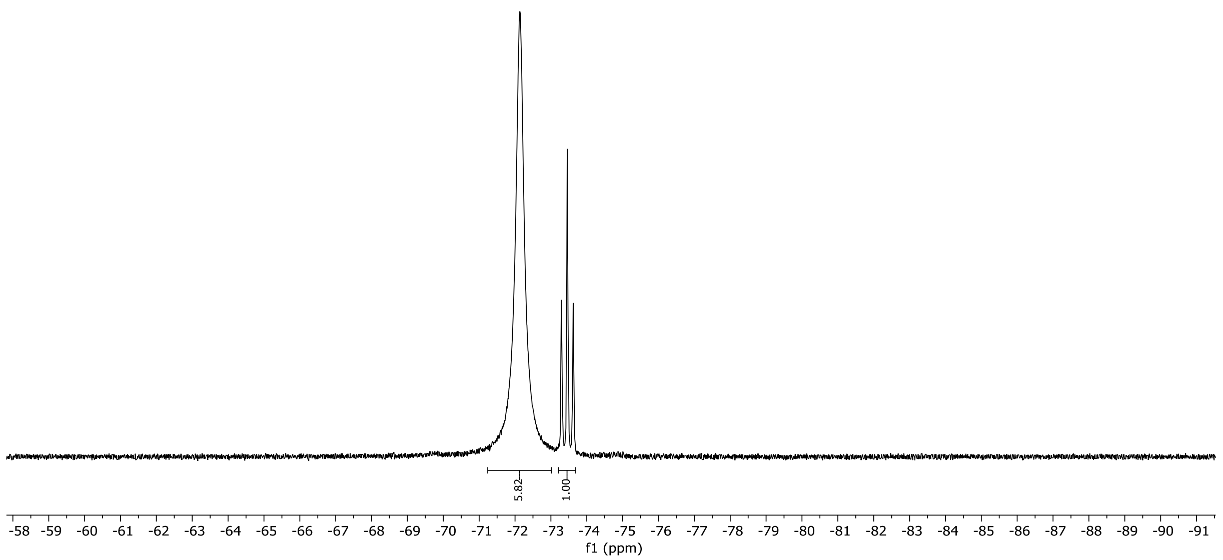

Figure S59. ^{19}F NMR of compound ***12*.**

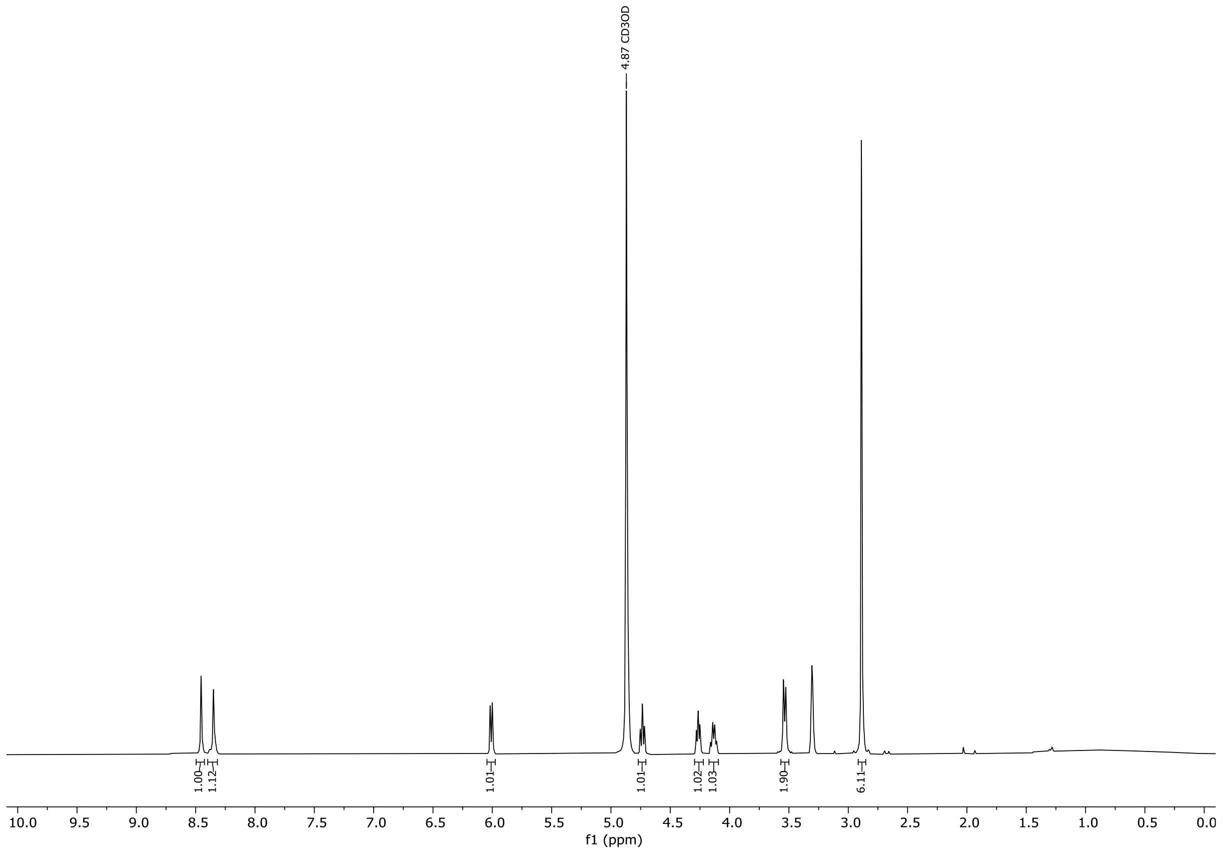

Figure S60. ^{1}H NMR of compound ***13***.

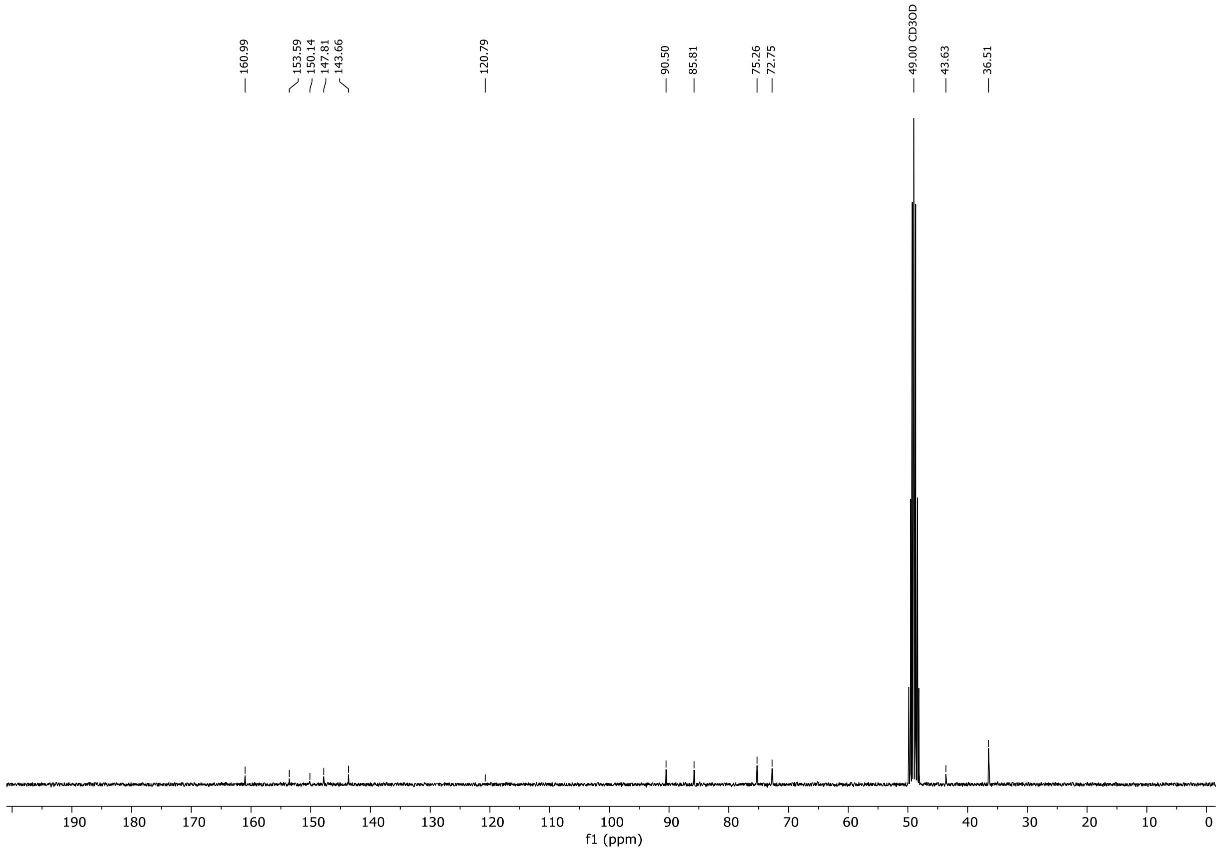

Figure S61. ^{13}C NMR of compound ***13***.

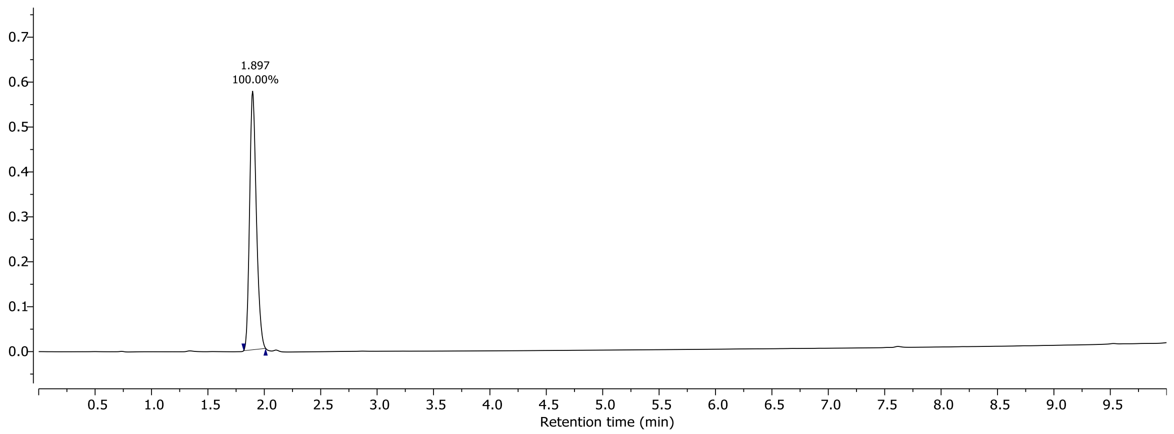

Figure S62. LCMS chromatogram of compound ***13*** at 254 nm.

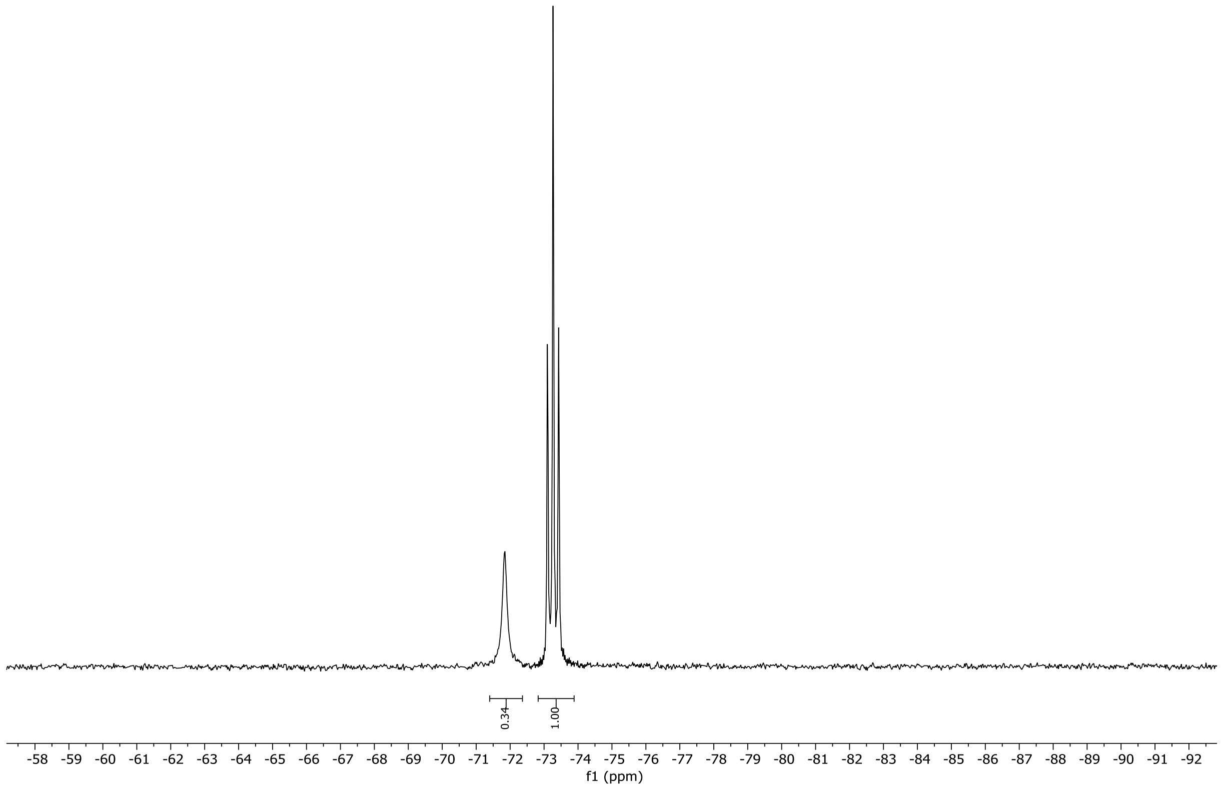

Figure S63. ^{19}F NMR of compound ***13***.

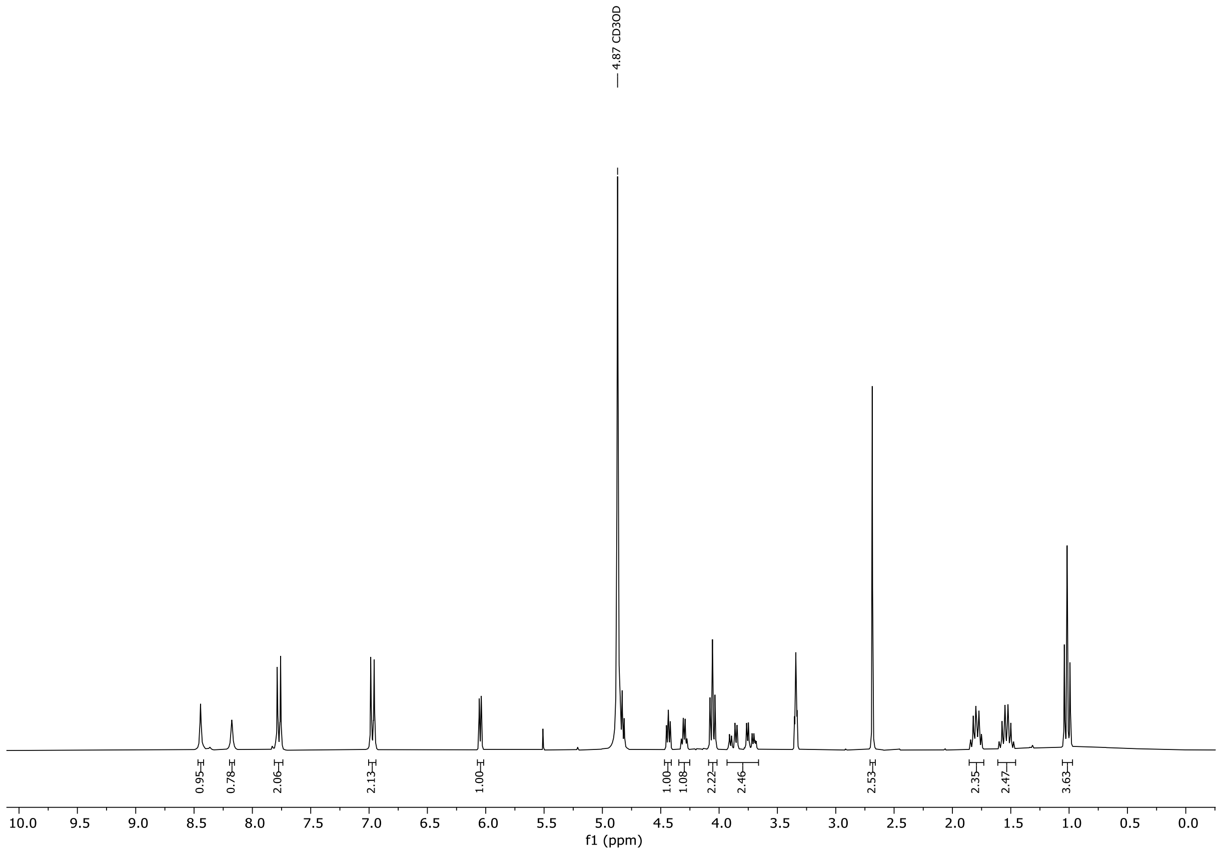

Figure S64. ^{1}H NMR of compound ***14***.

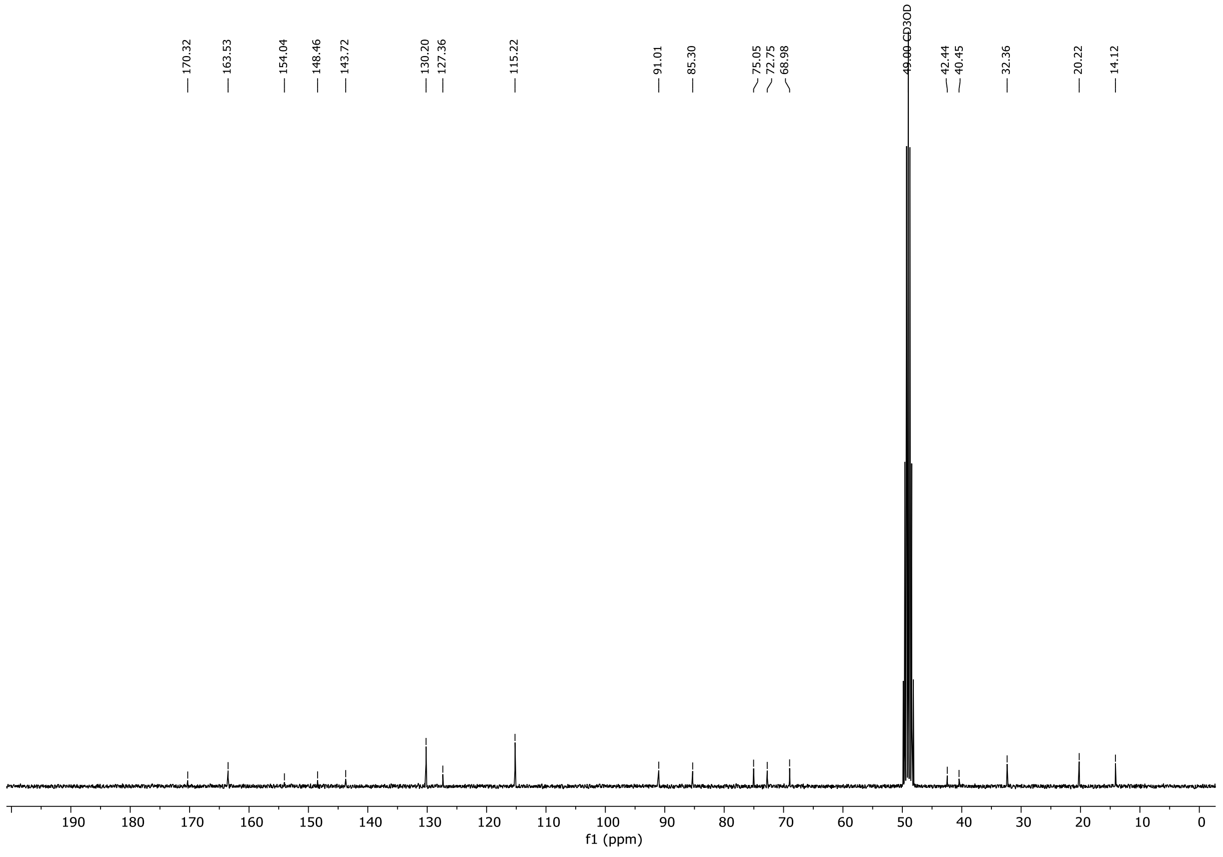

Figure S65. ^{13}C NMR of compound ***14***.

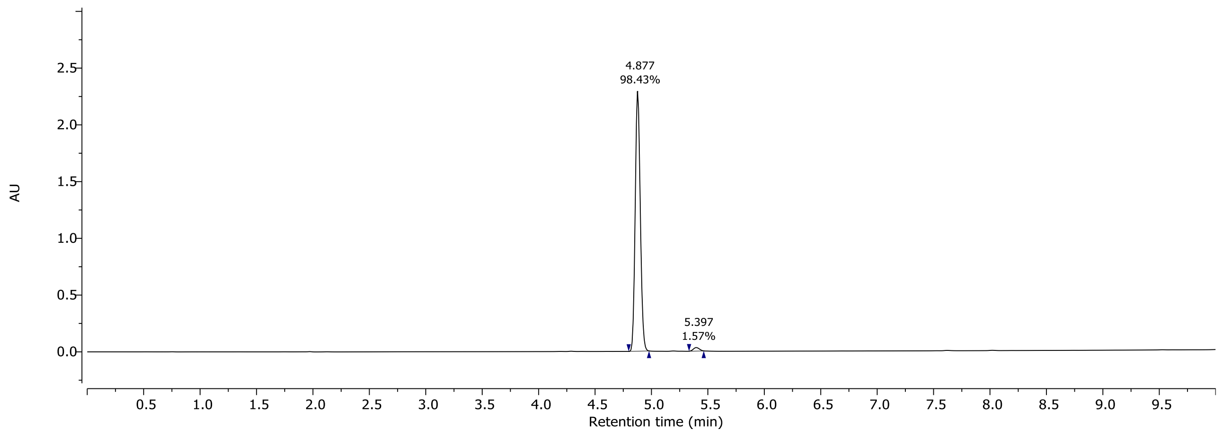

Figure S66. LCMS chromatogram of compound ***14*** at 254 nm.

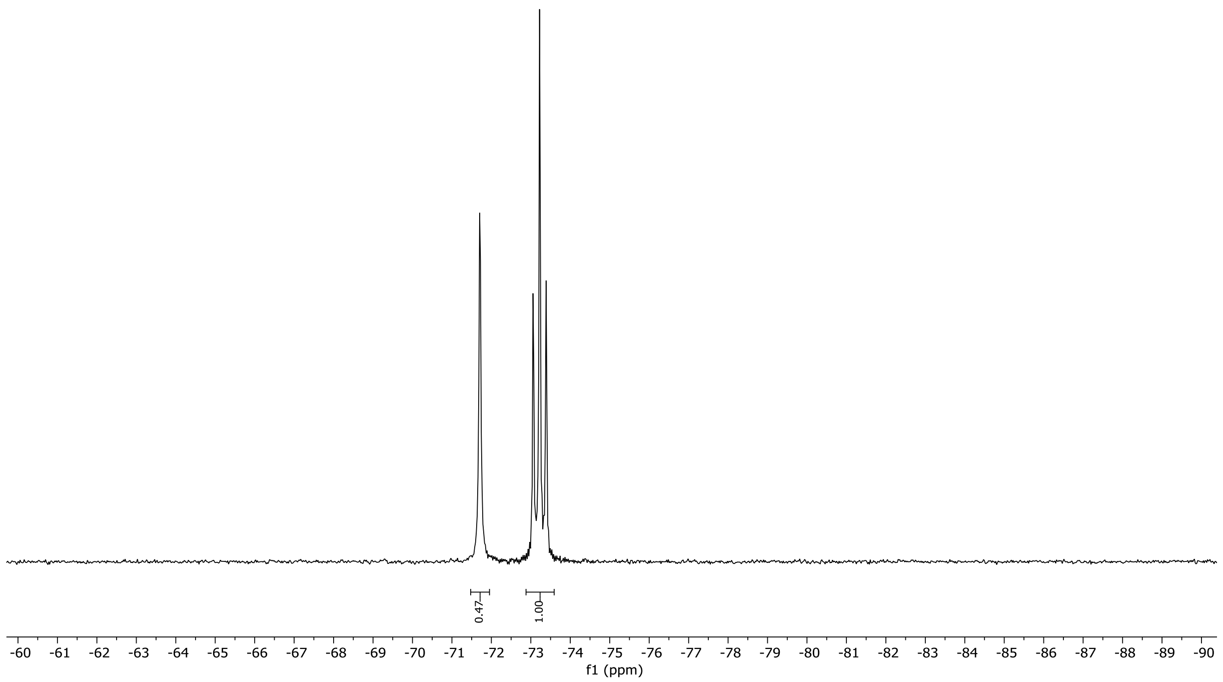

Figure S67. ^{19}F NMR of compound ***14*.**

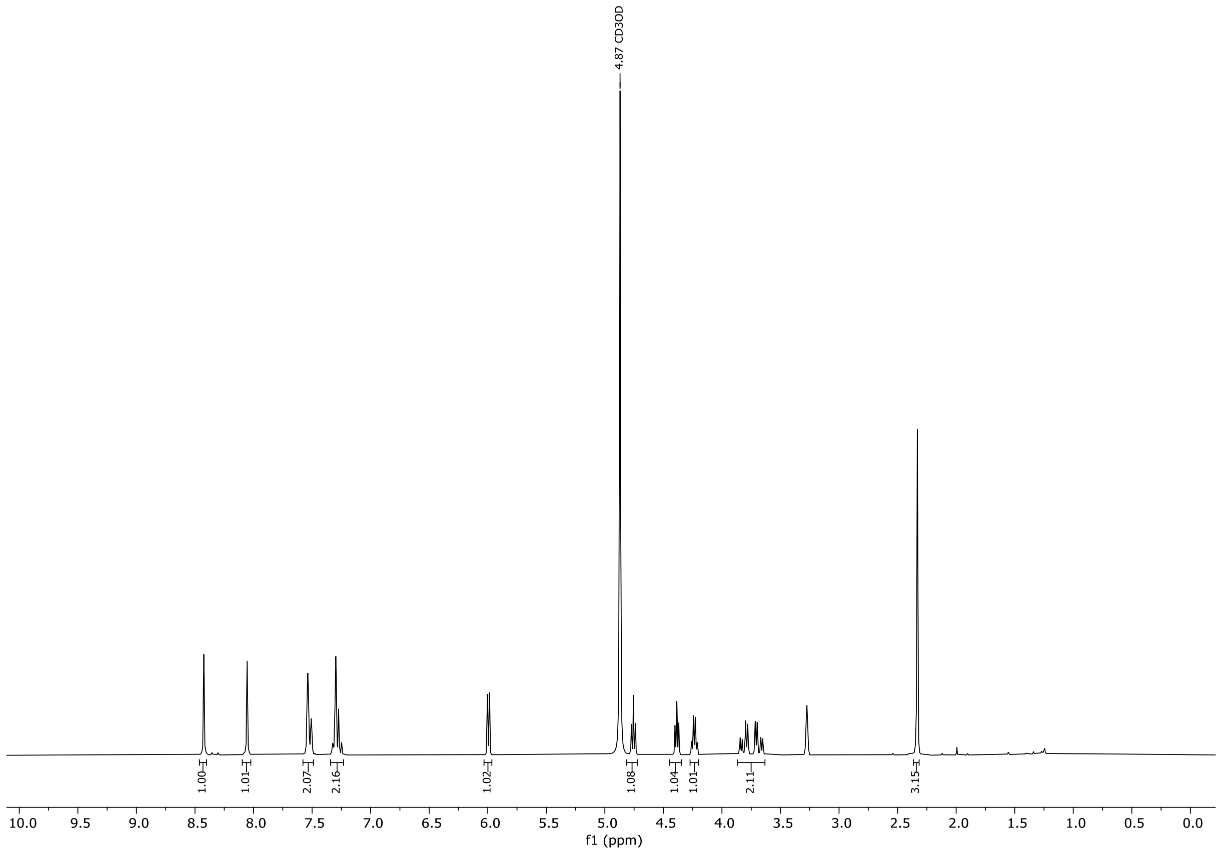

Figure S68. ^{1}H NMR of compound ***15***.

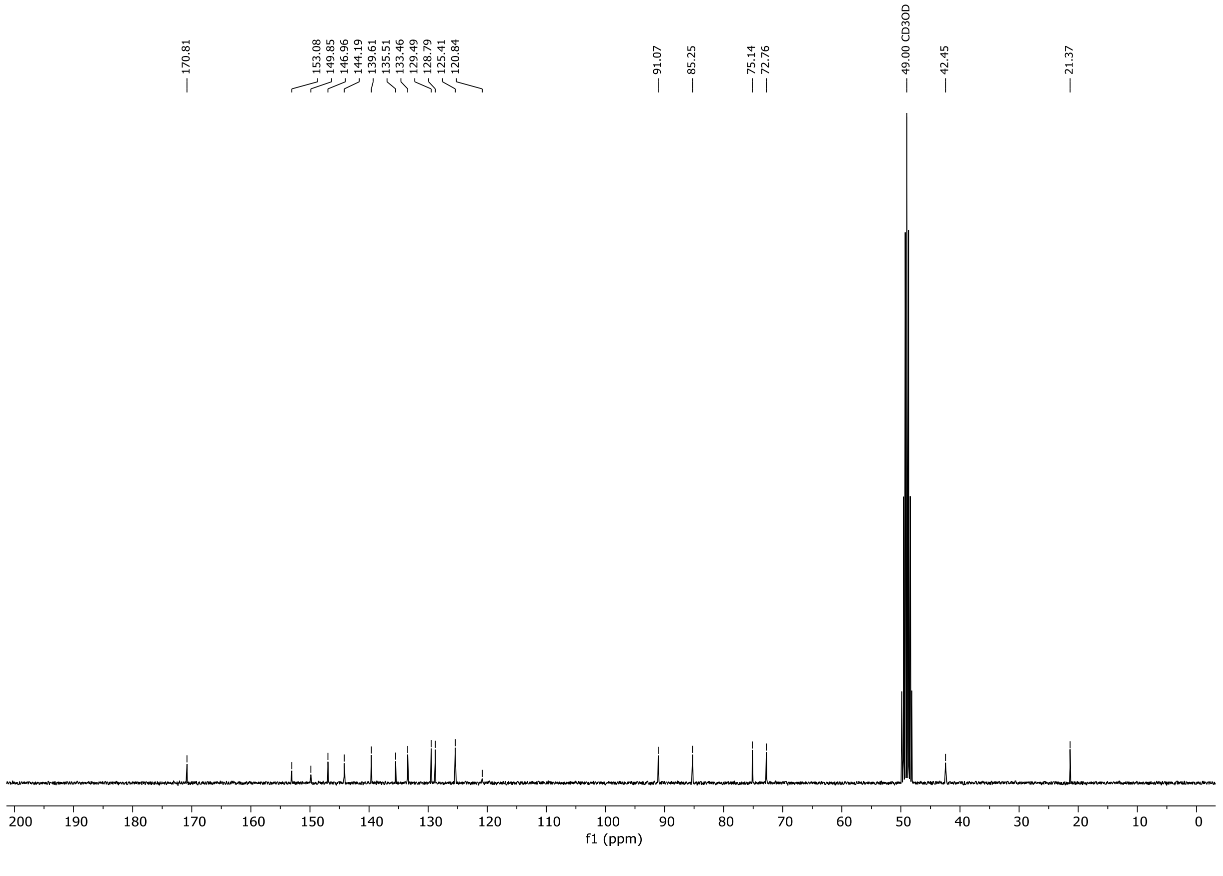

Figure S69. ^{13}C NMR of compound ***15***.

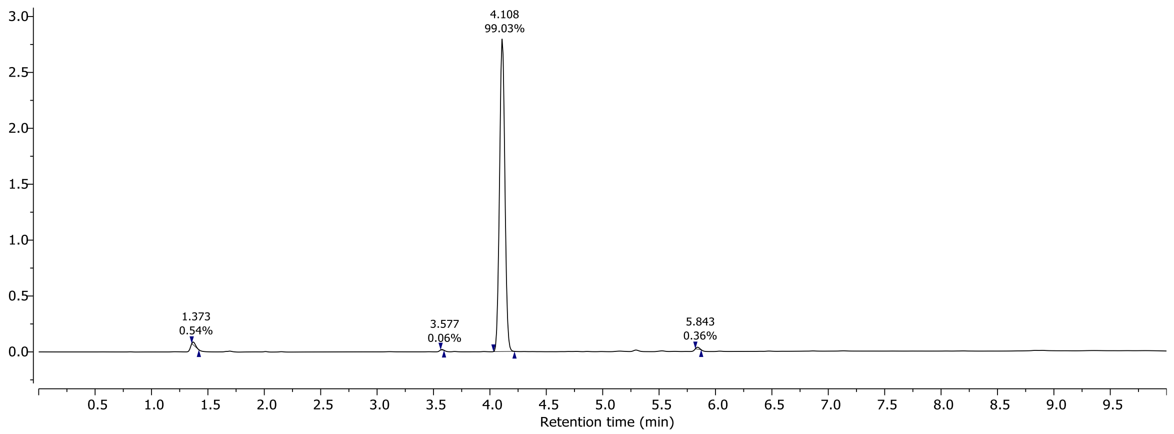

Figure S70. LCMS chromatogram of compound *15* at 254 nm.

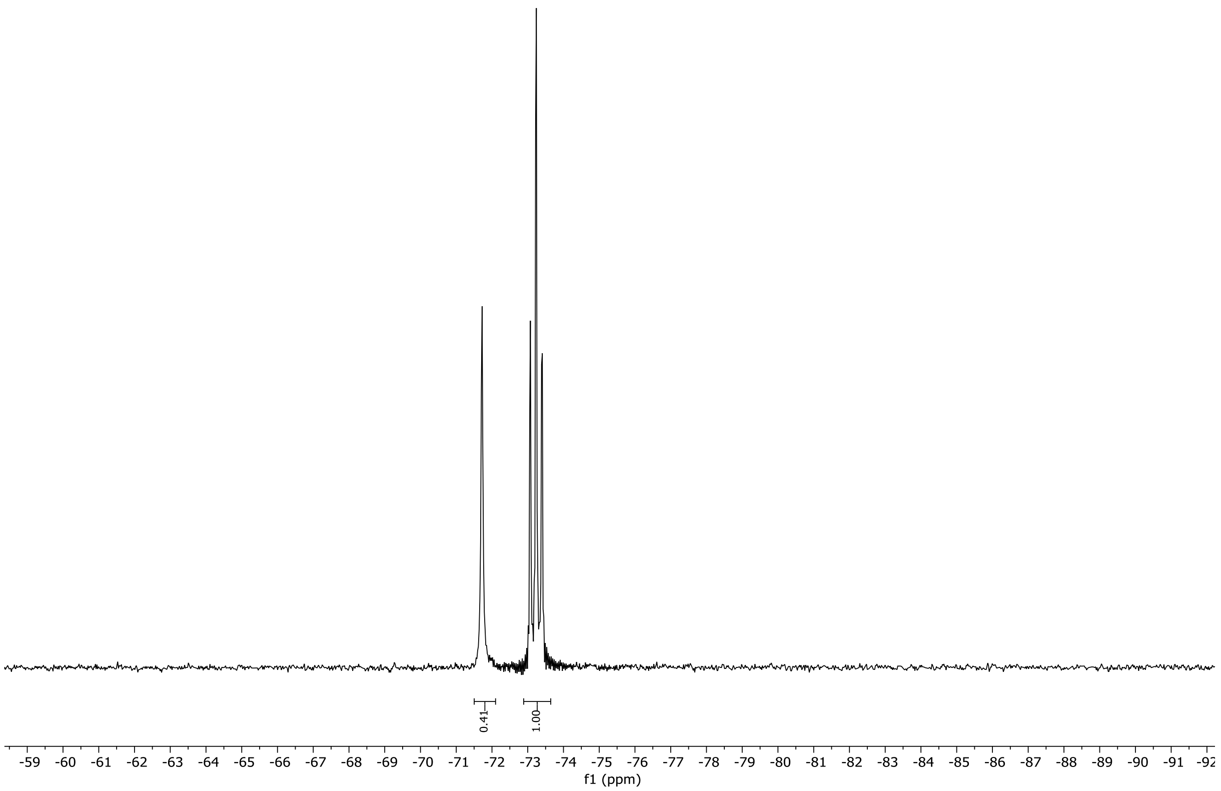

Figure S71. ^{19}F NMR of compound ***15***.

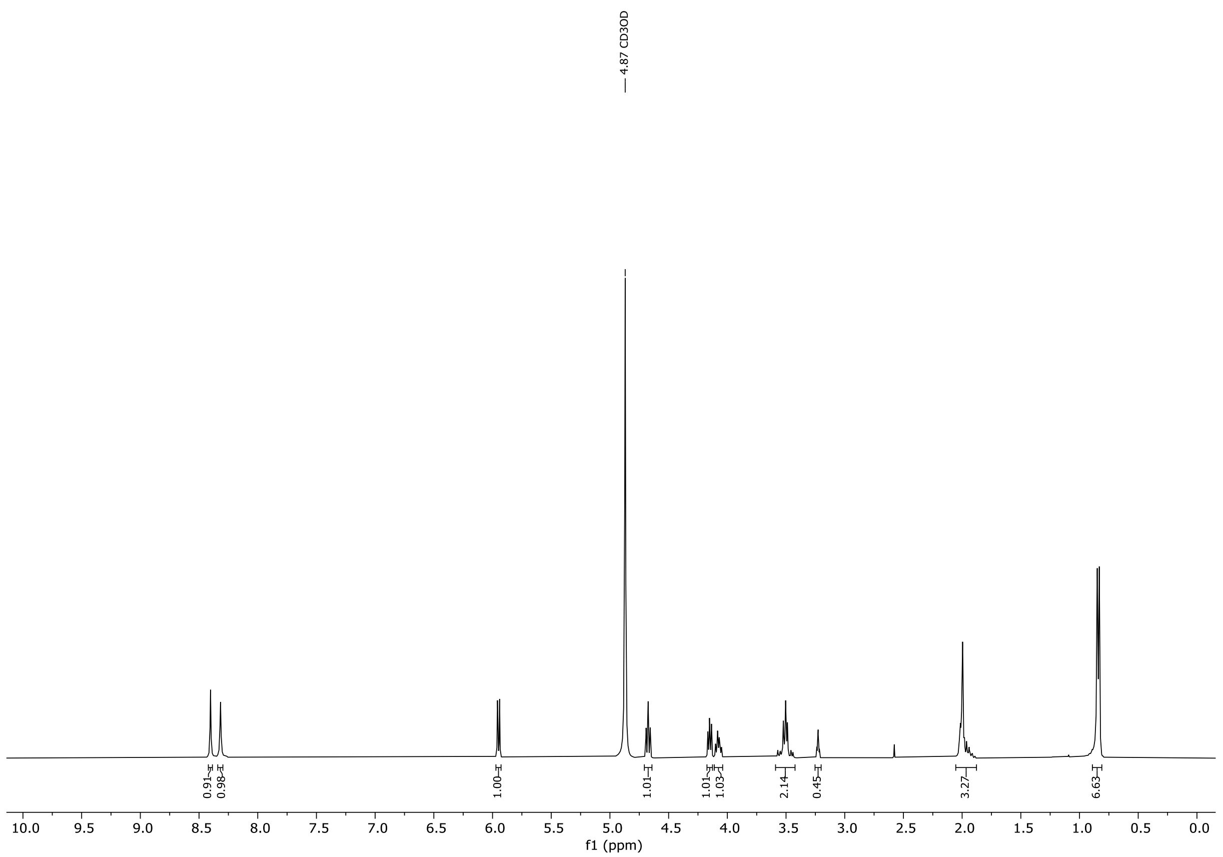

Figure S72. ^{1}H NMR of compound ***16***.

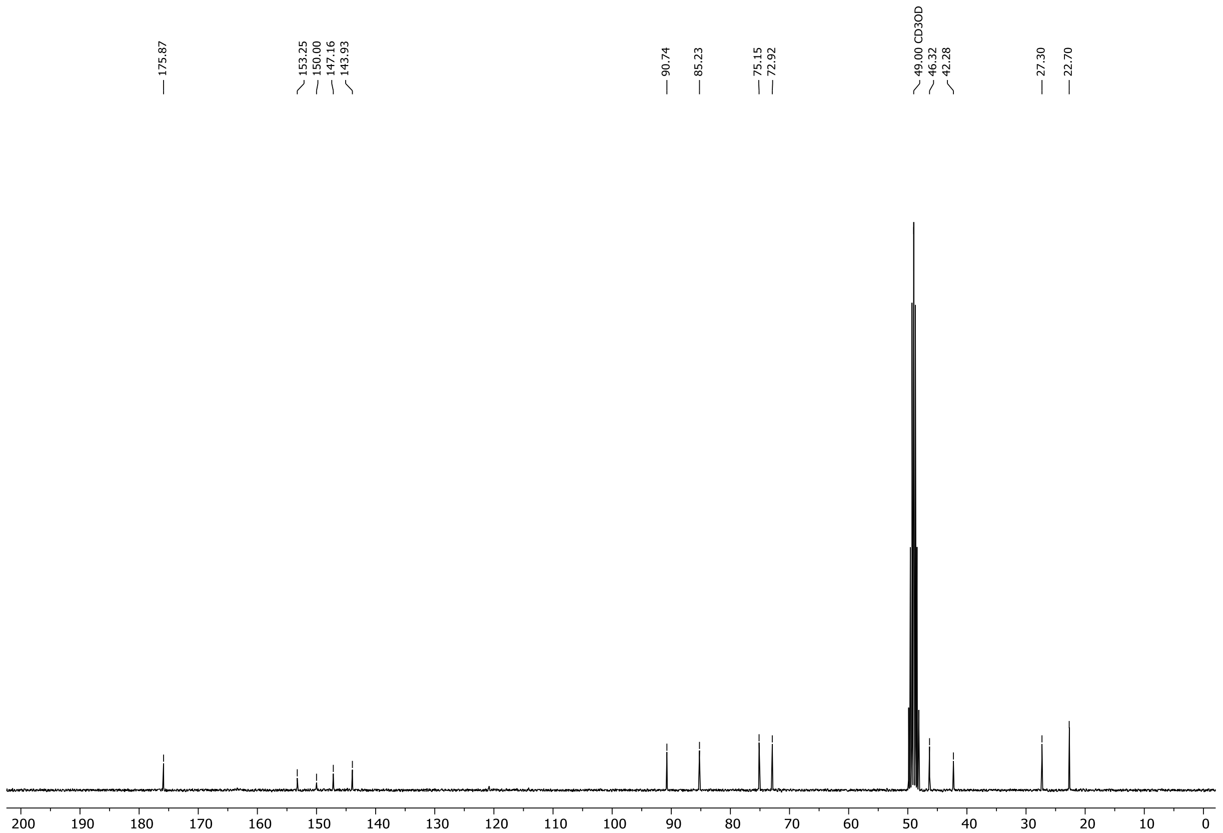

Figure S73. ^{13}C NMR of compound ***16***.

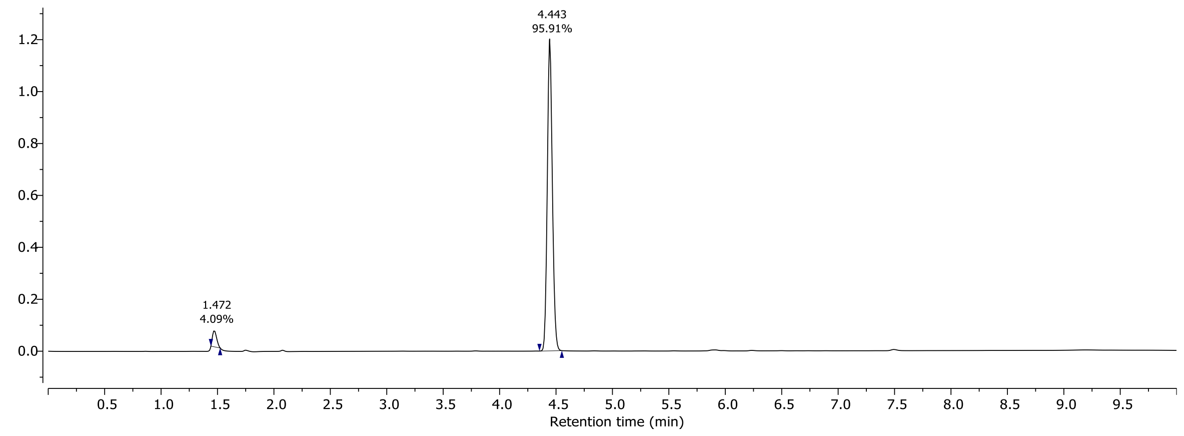

Figure S74. LCMS chromatogram of compound ***16*** at 254 nm.

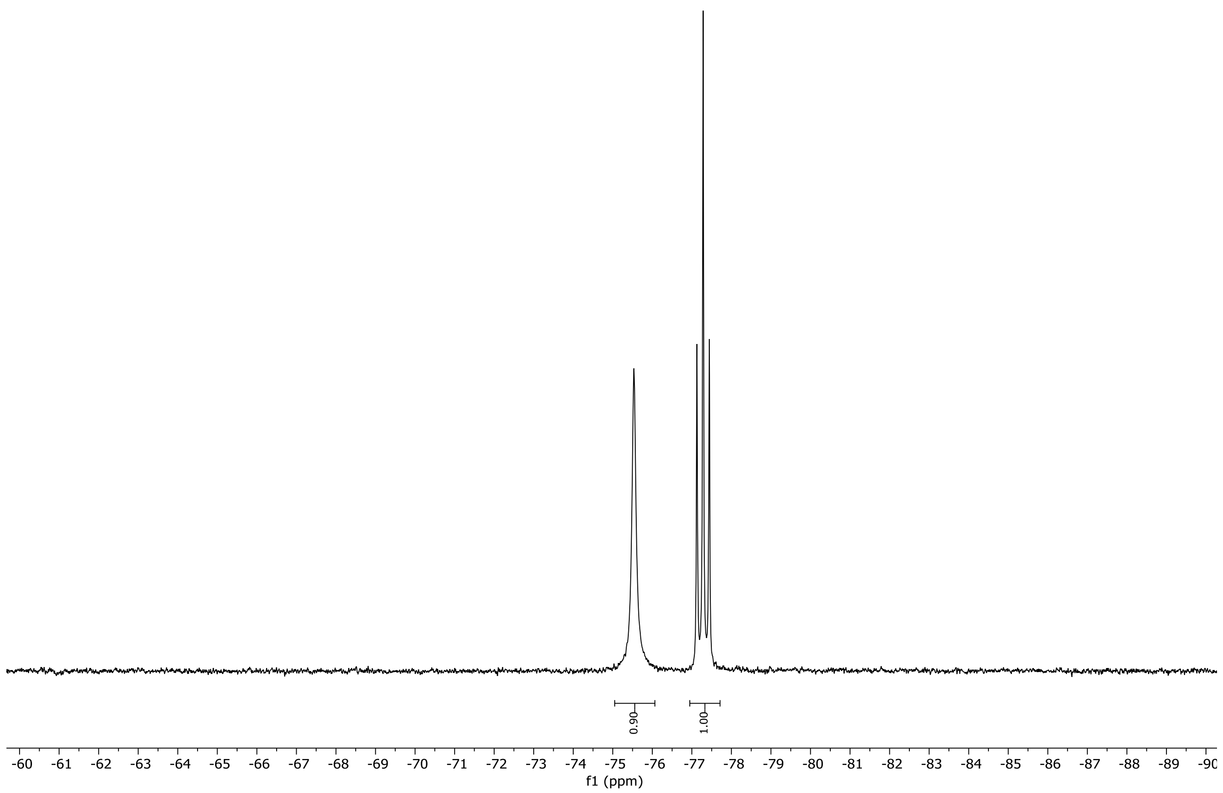

Figure S75.^{19}F NMR of compound ***16***.

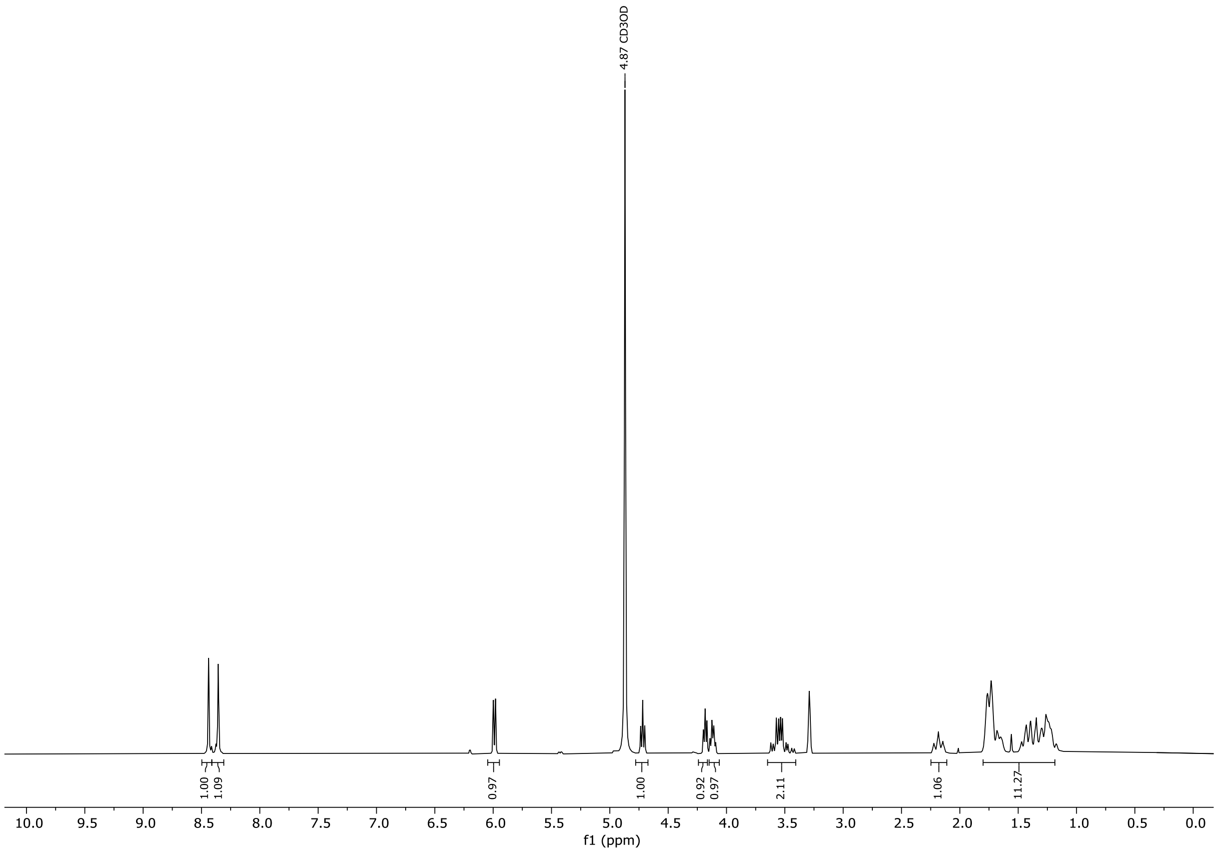

Figure S76. ^{1}H NMR of compound ***17***.

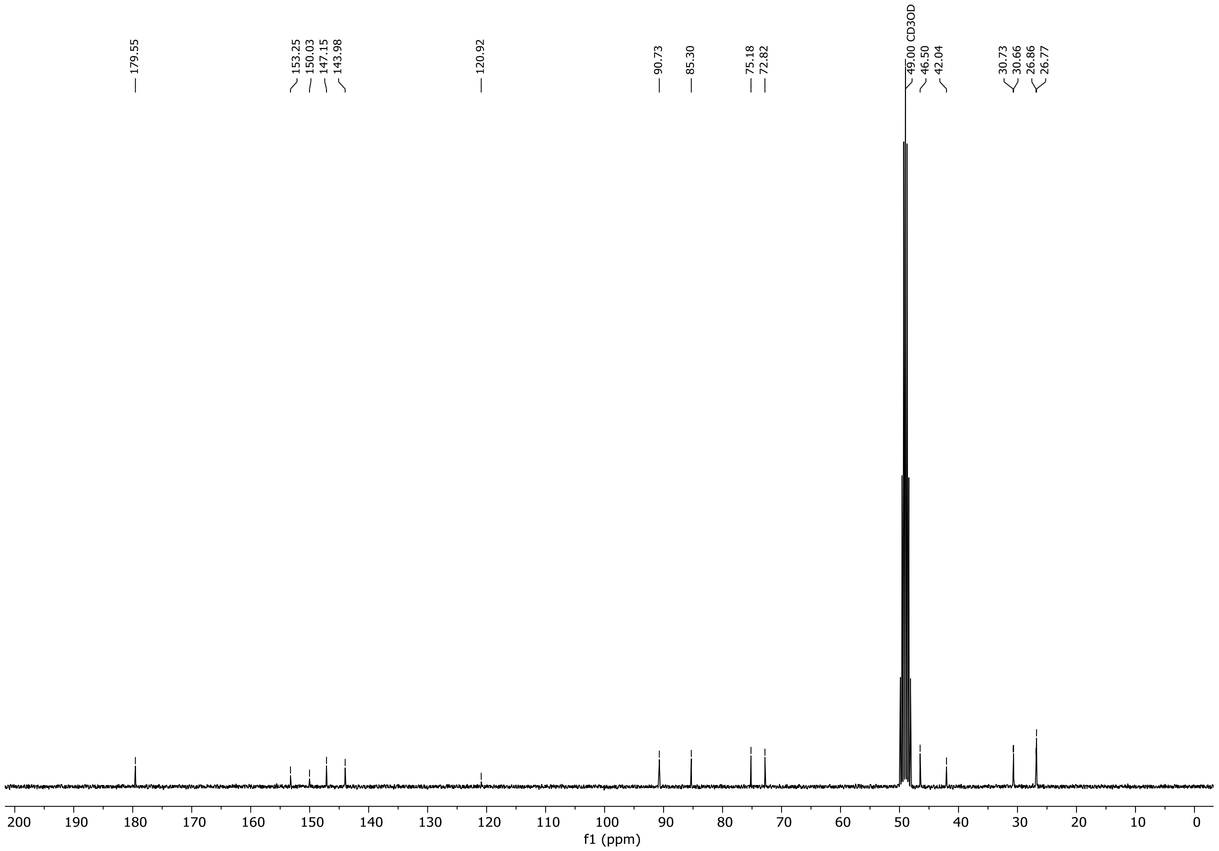

Figure S77. ^{13}C NMR of compound ***17***.

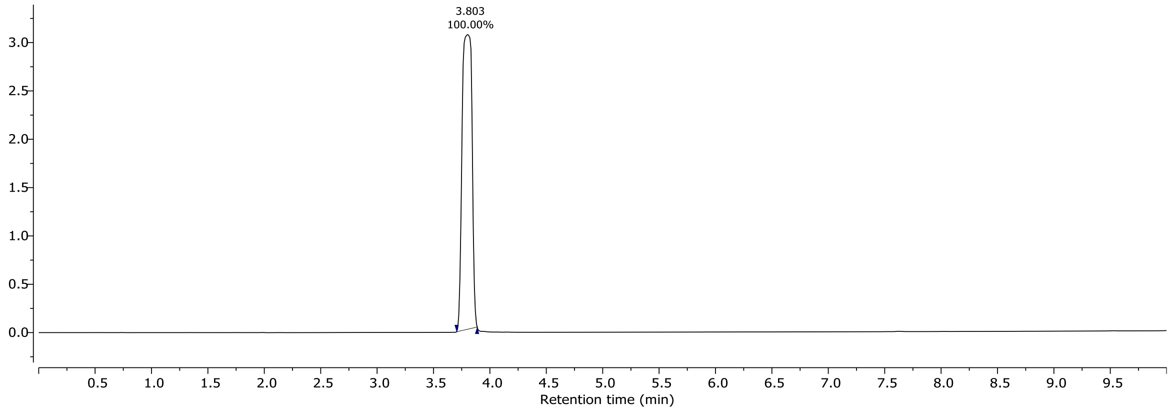

Figure S78. LCMS chromatogram of compound **17** at 254 nm.

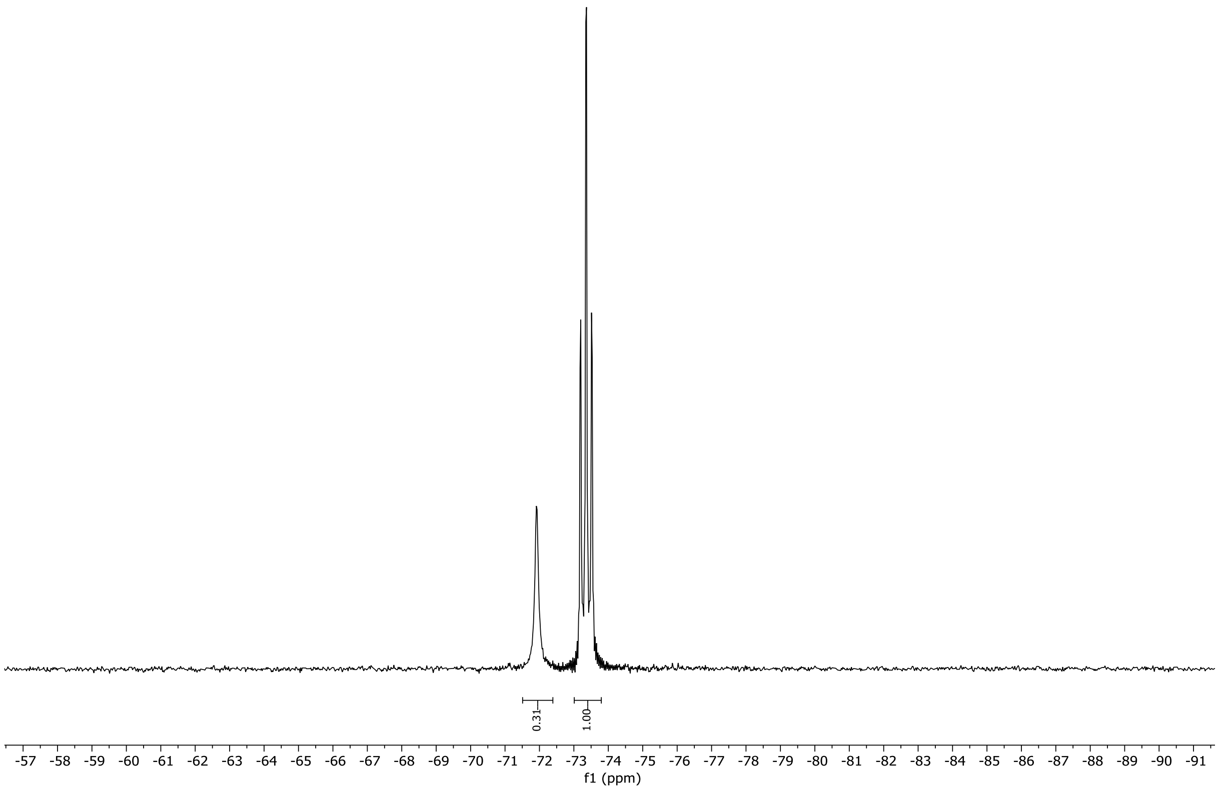

Figure S79. ^{19}**F** NMR of compound **17**.

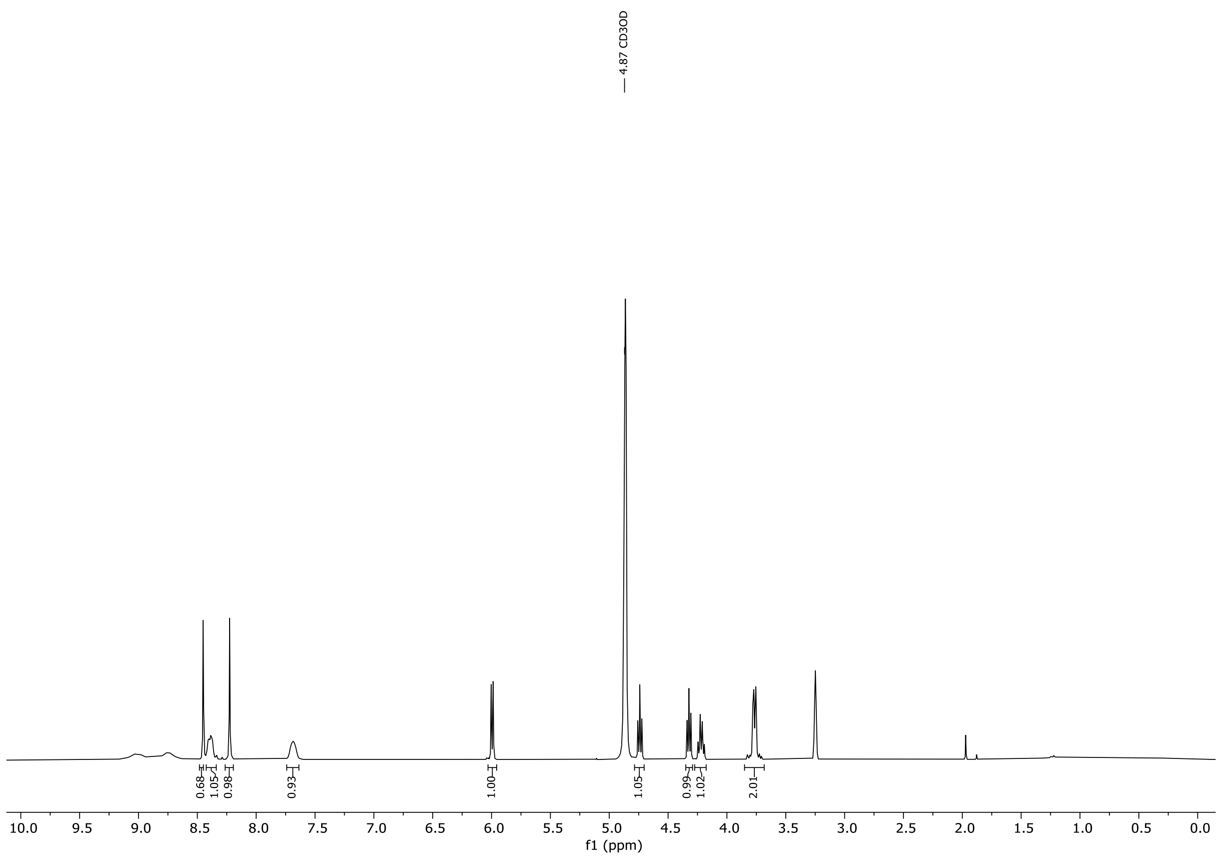

Figure S80. ^{1}H NMR of compound ***18***.

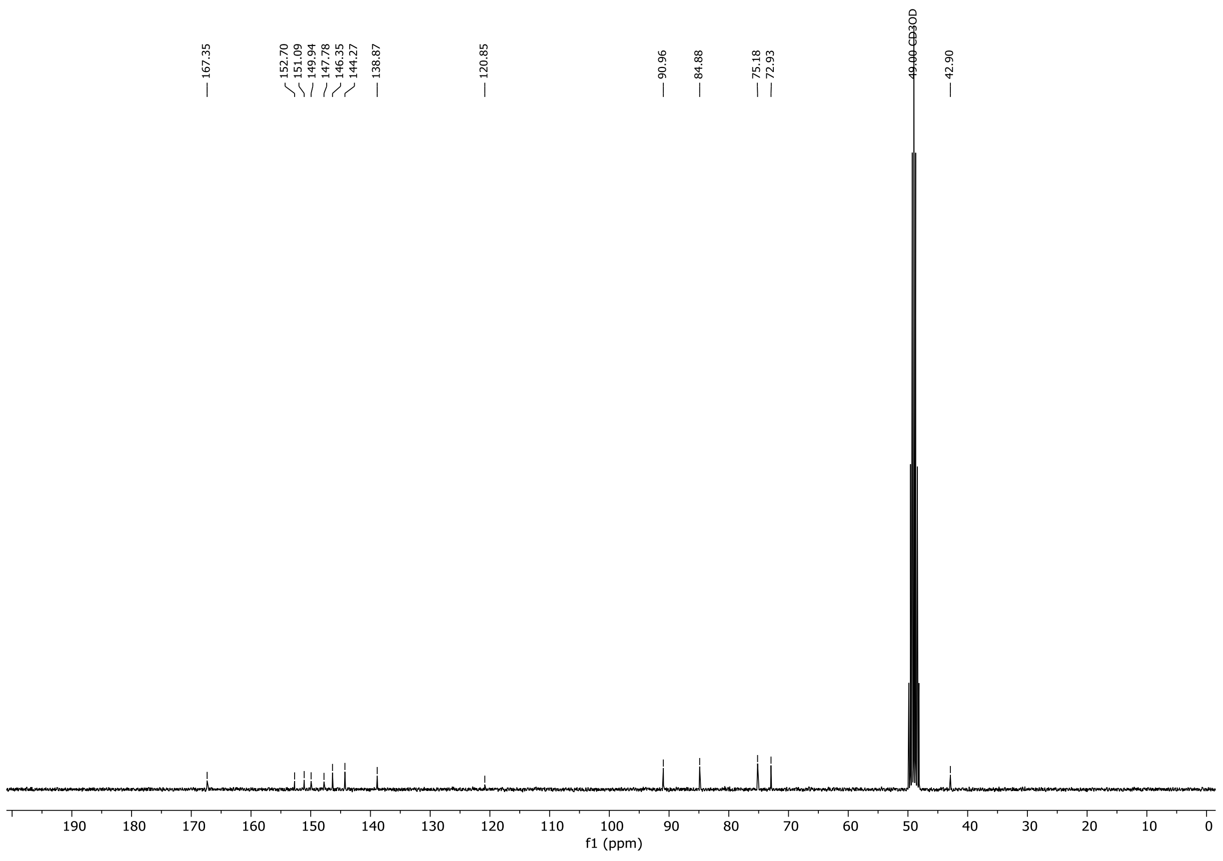

Figure S81. ^{13}C NMR of compound ***18***.

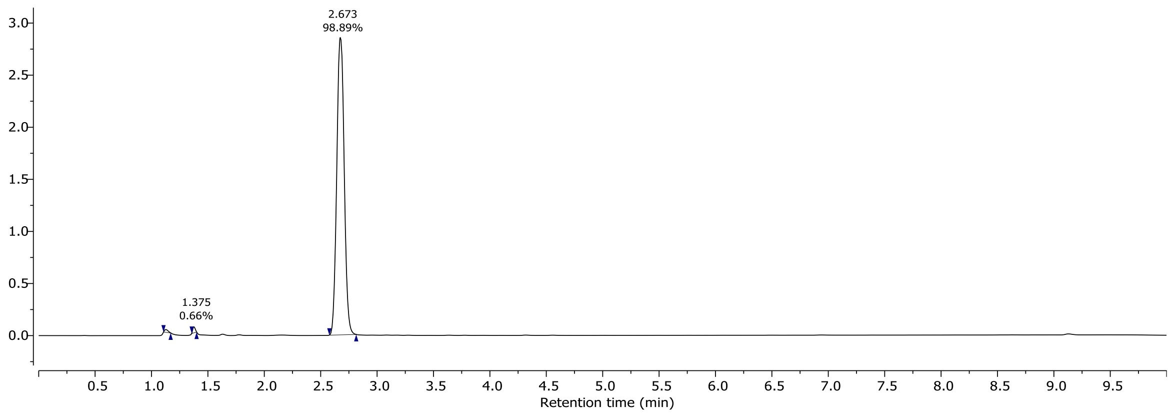

Figure S82. LCMS chromatogram of compound ***18*** at 254 nm.

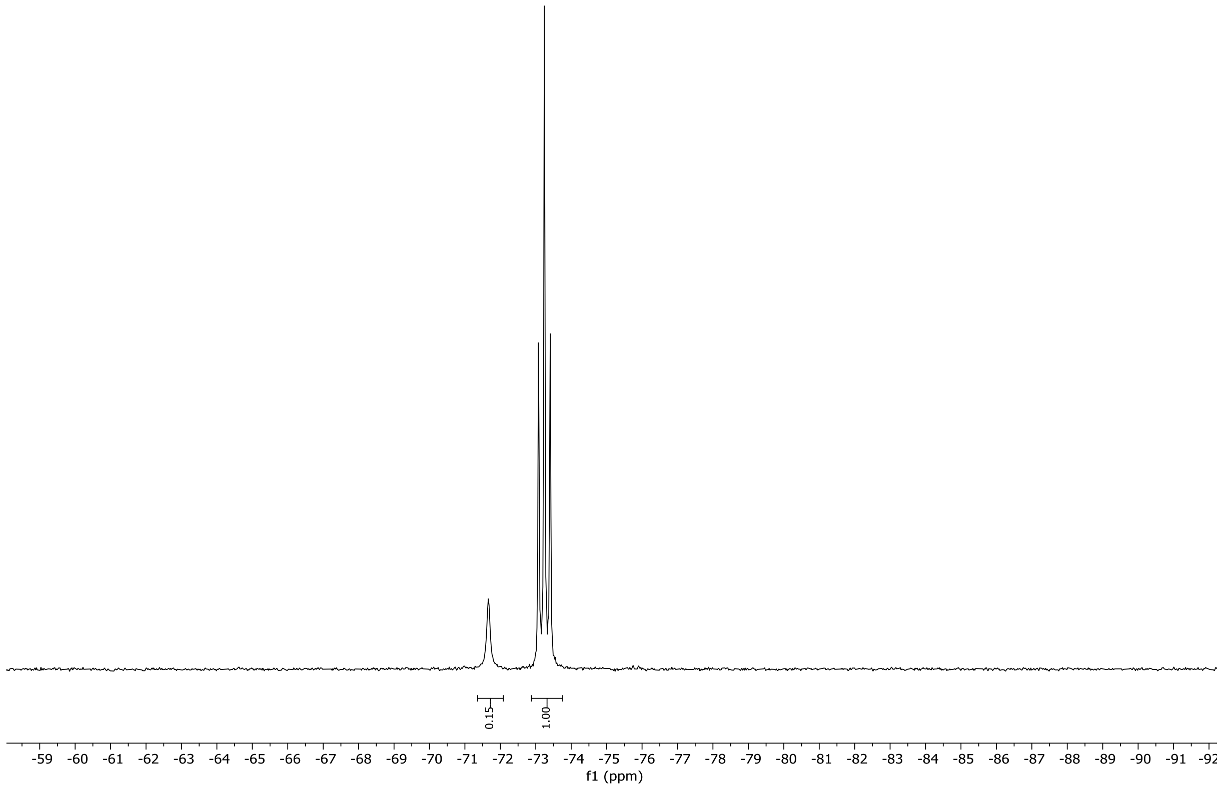

Figure S83. ^{19}F NMR of compound ***18***.

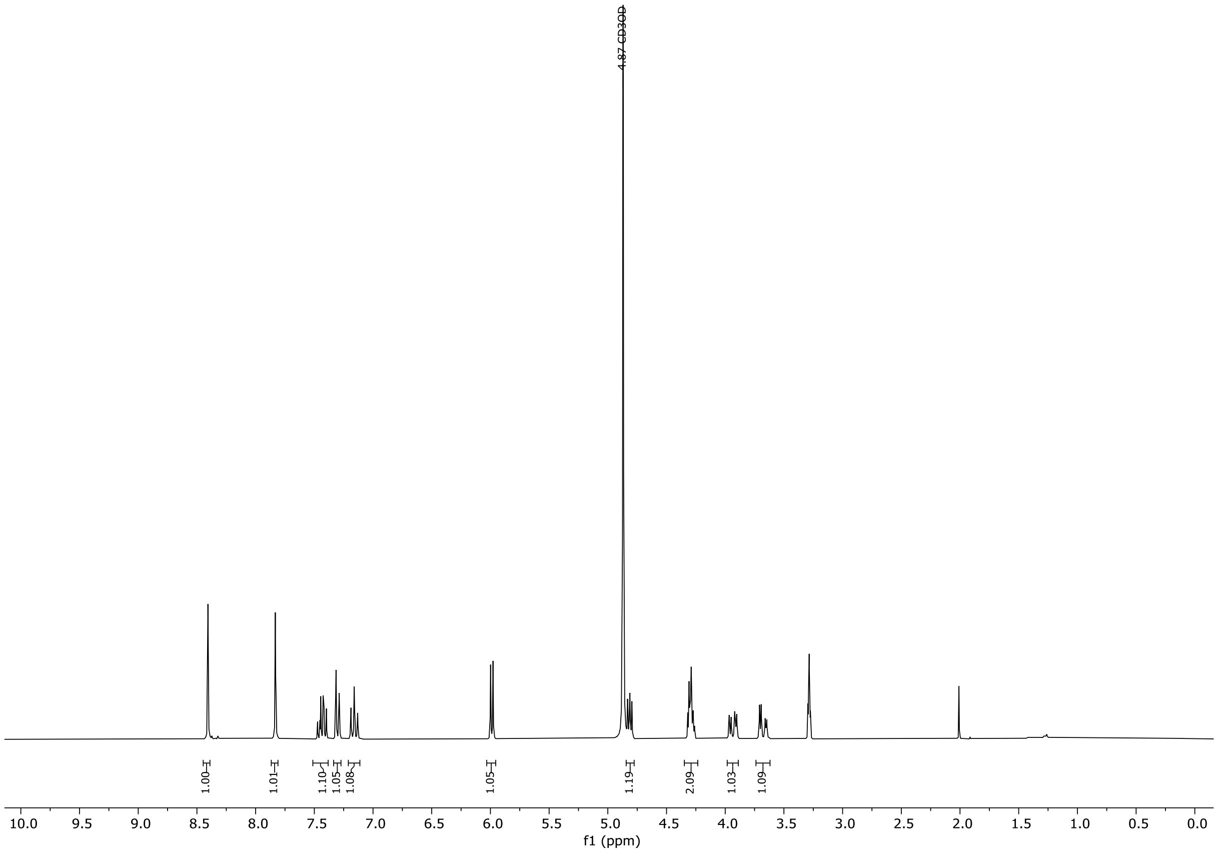

Figure S84. ^{1}H NMR of compound ***19***.

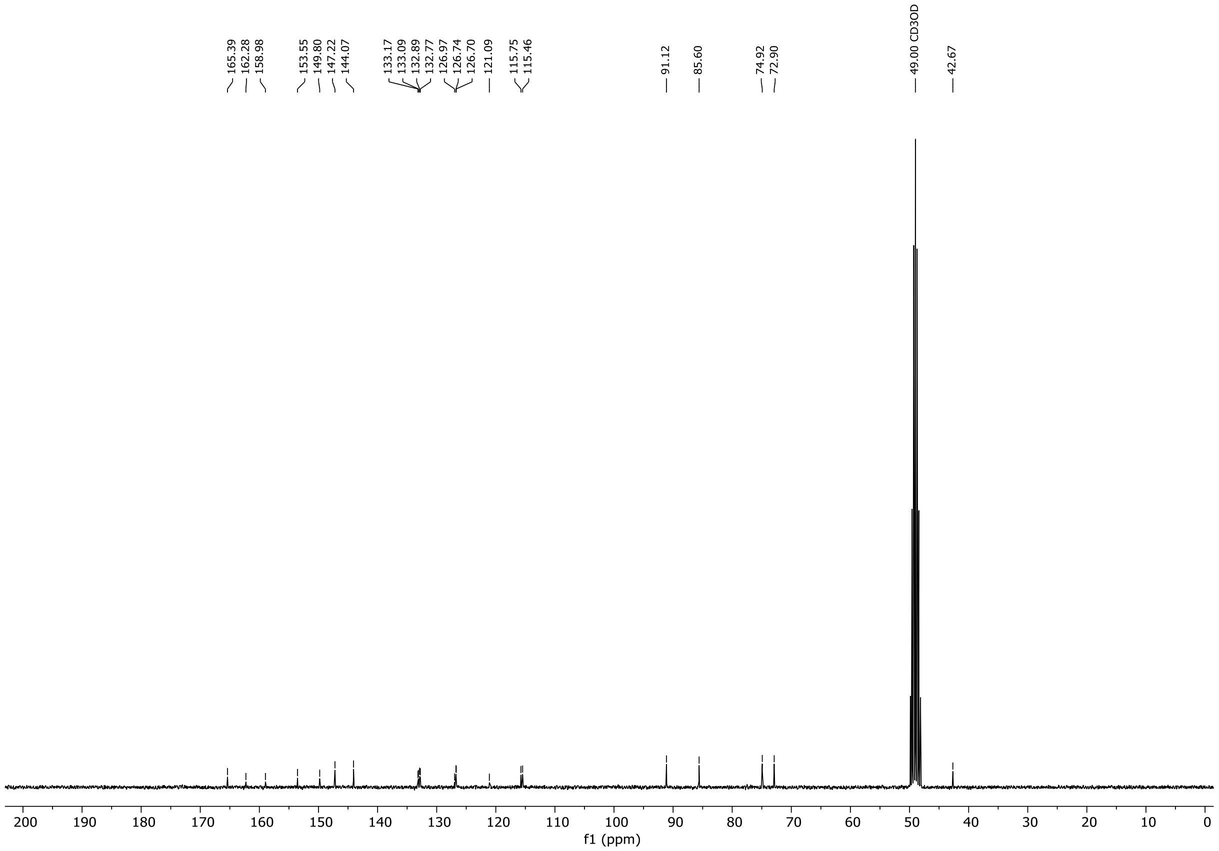

Figure S85. ^{13}C NMR of compound ***19***.

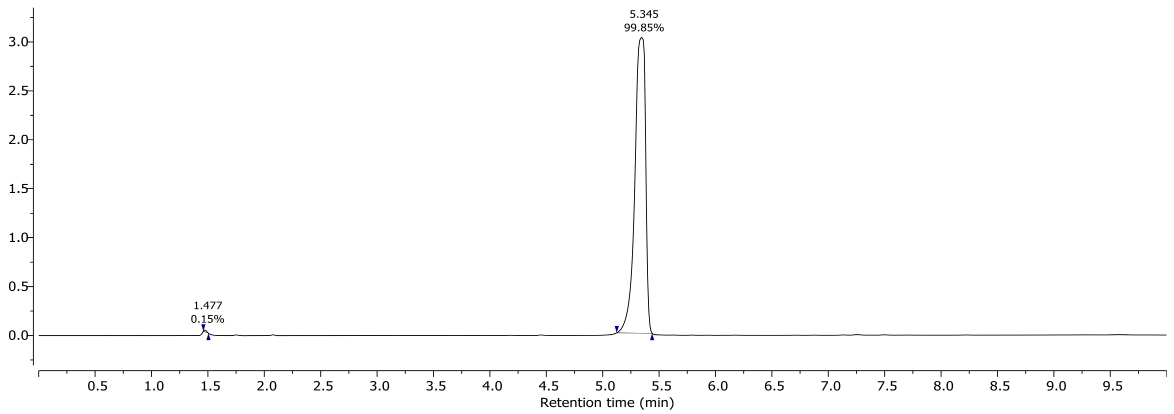

Figure S86. LCMS chromatogram of compound ***19*** at 254 nm.

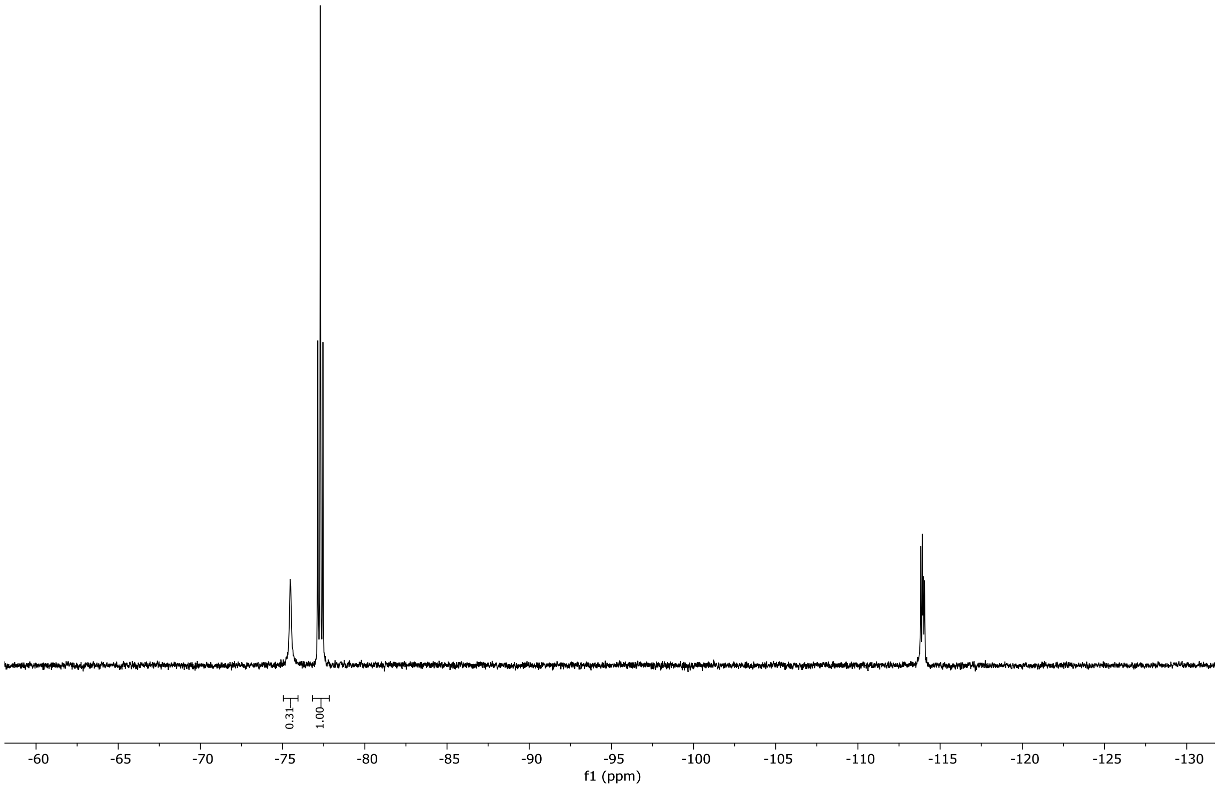

Figure S87. ^{19}F NMR of compound ***19***.

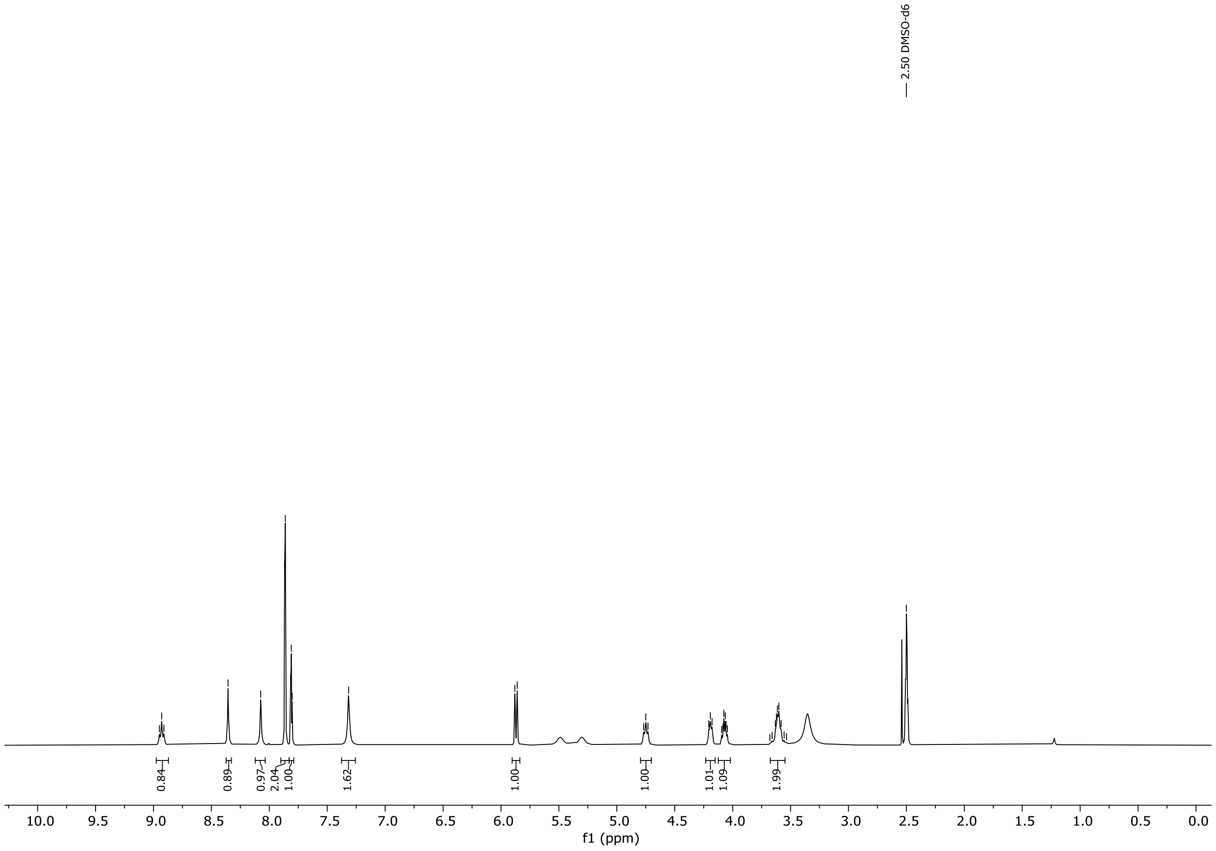

Figure S88. ^{1}H NMR of compound ***20.***

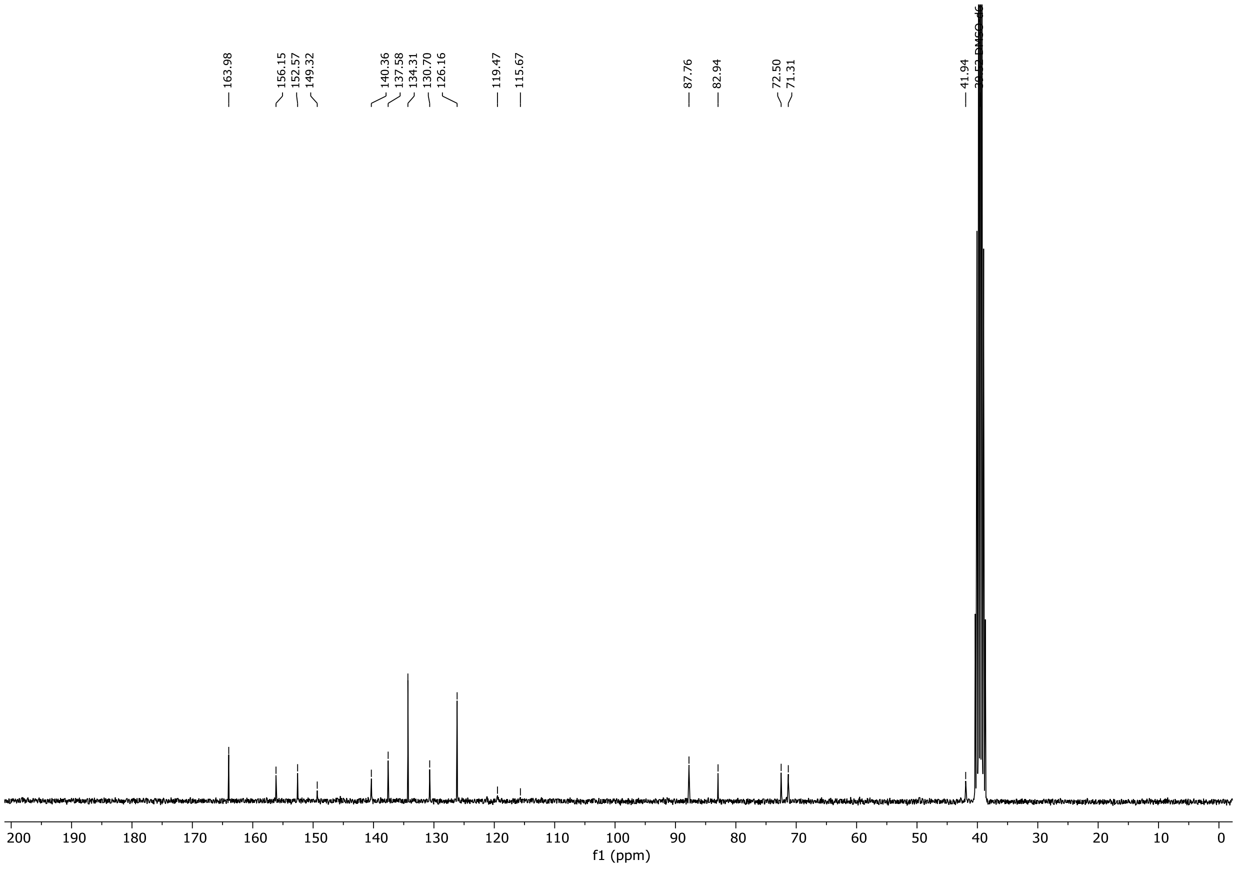

Figure S89. ^{13}C NMR of compound ***20***.

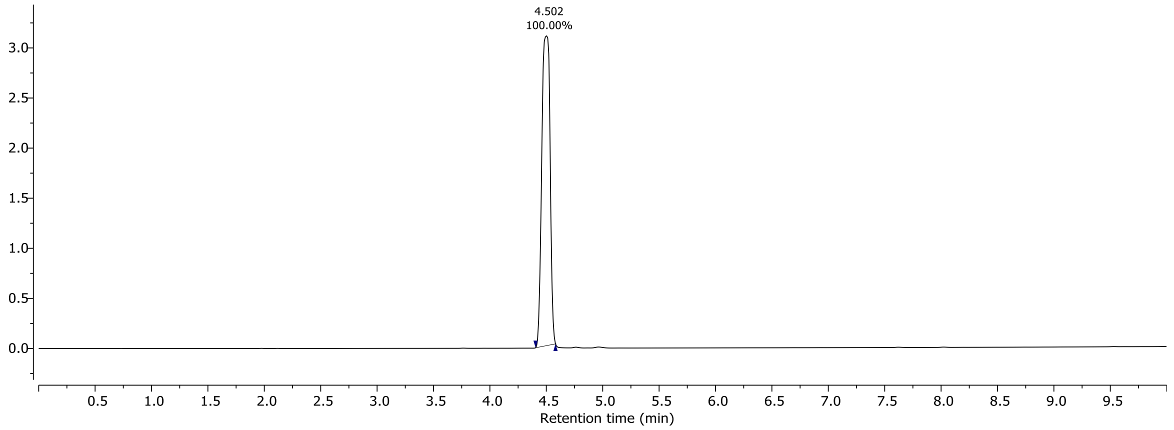

Figure S90. LCMS chromatogram of compound ***20*** at 254 nm.

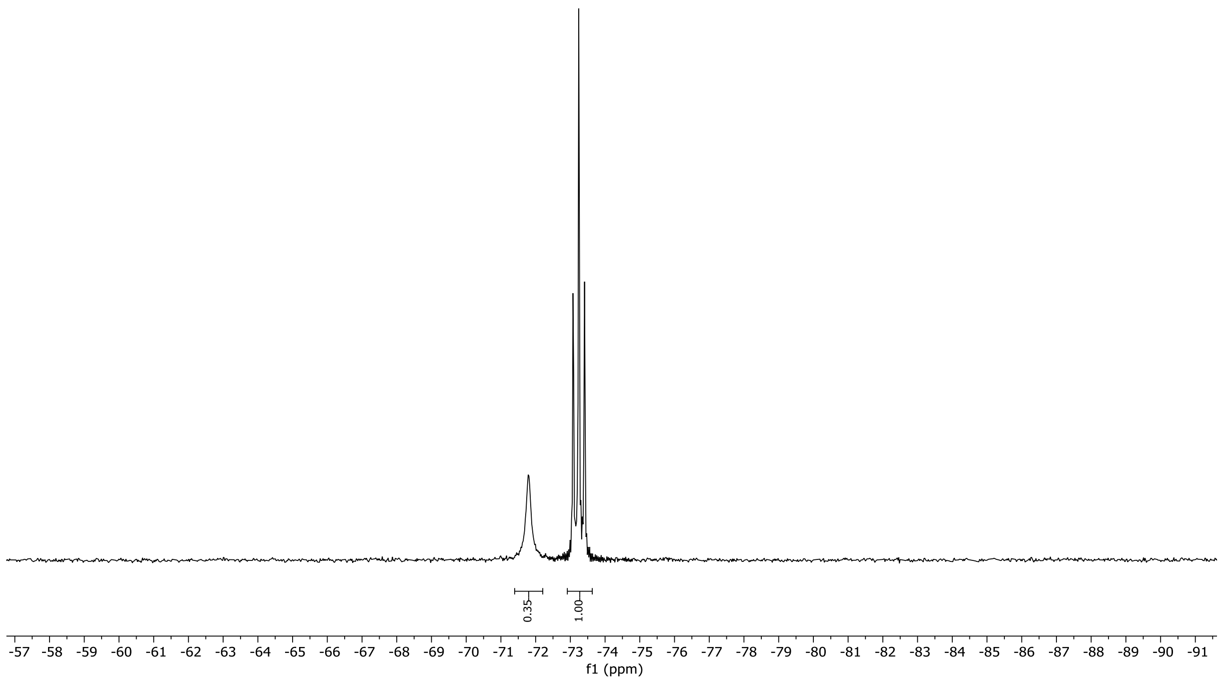

Figure S91. ^{19}F NMR of compound ***20.***

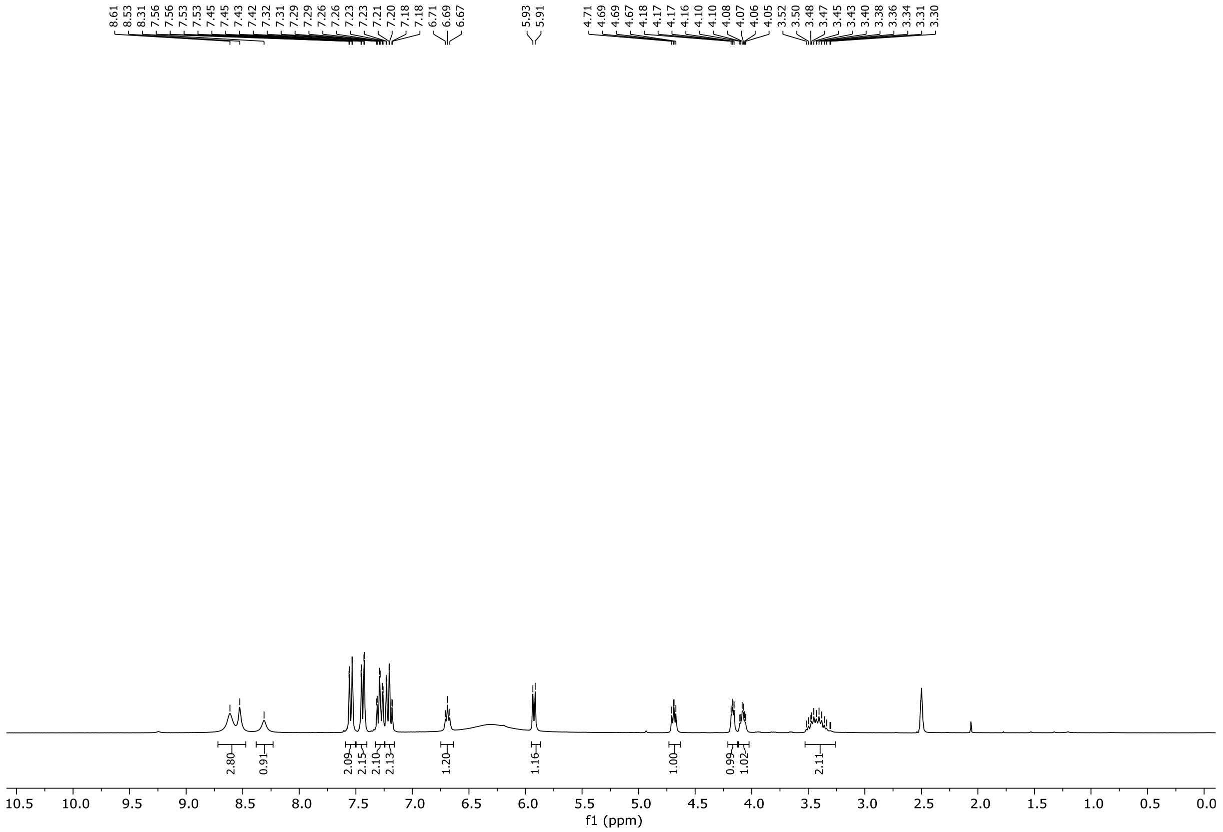

Figure S92. ^{1}H NMR of compound ***21***.

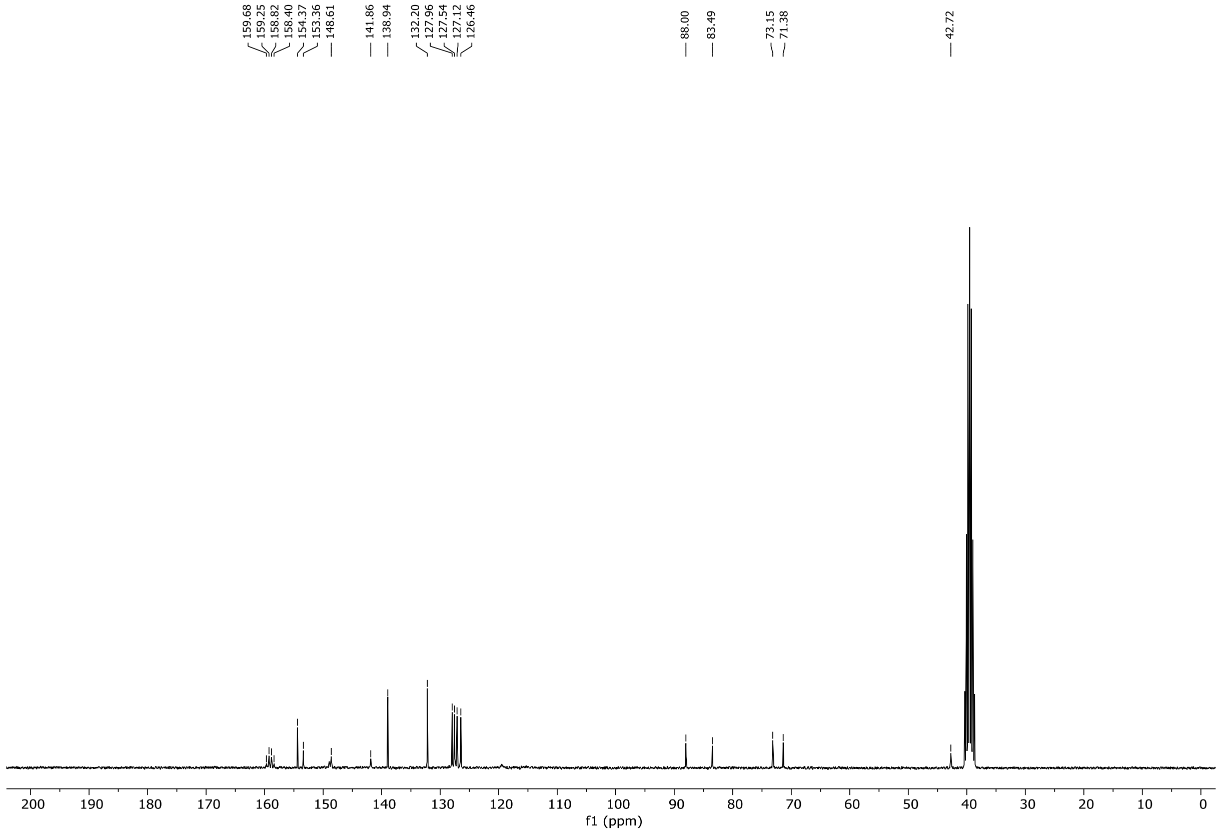

Figure S93. ^{13}C NMR of compound ***21***.

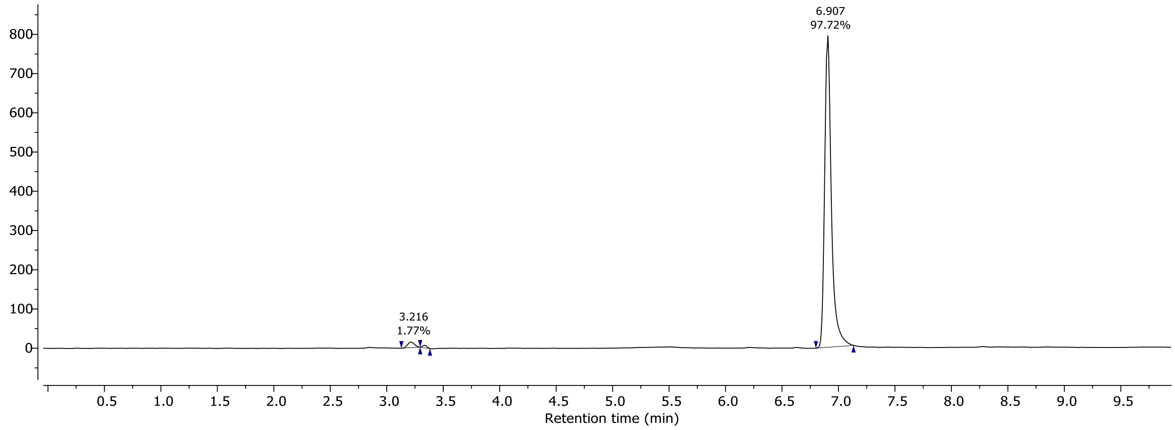

Figure S94. LCMS chromatogram of compound ***21*** at 254 nm.

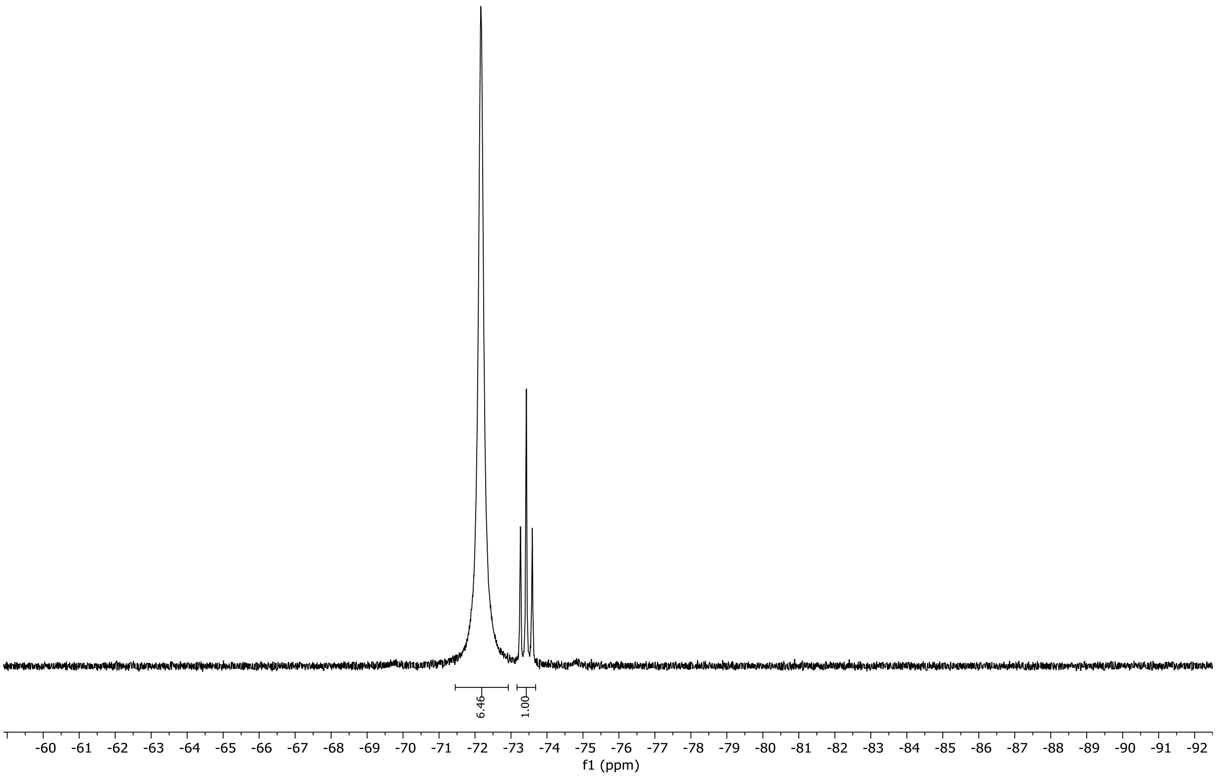

Figure S95. ^{19}F NMR of compound ***21*.**

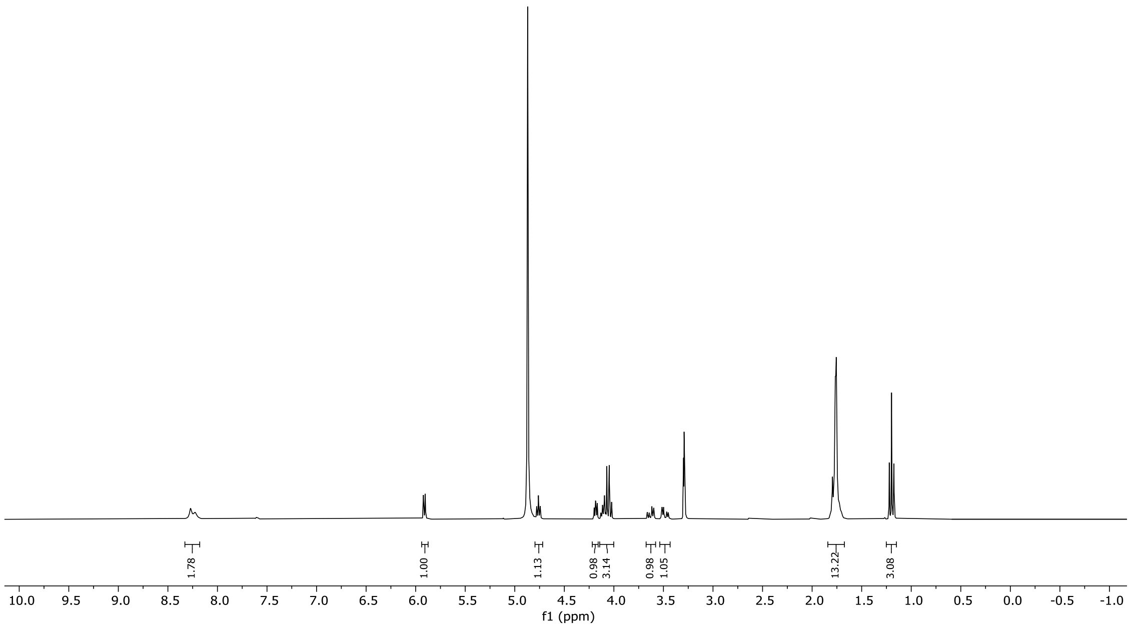

Figure S96. ¹H NMR of compound **26**.

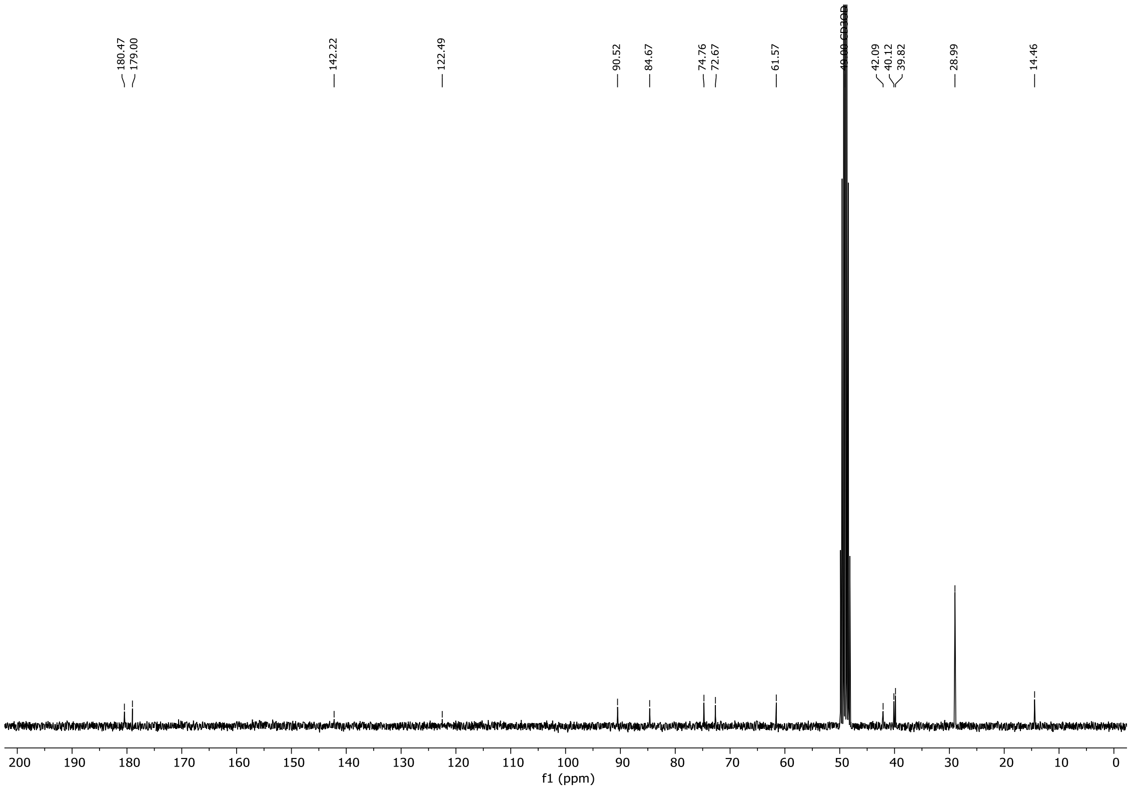

Figure S97. ^{13}C NMR of compound **26.**

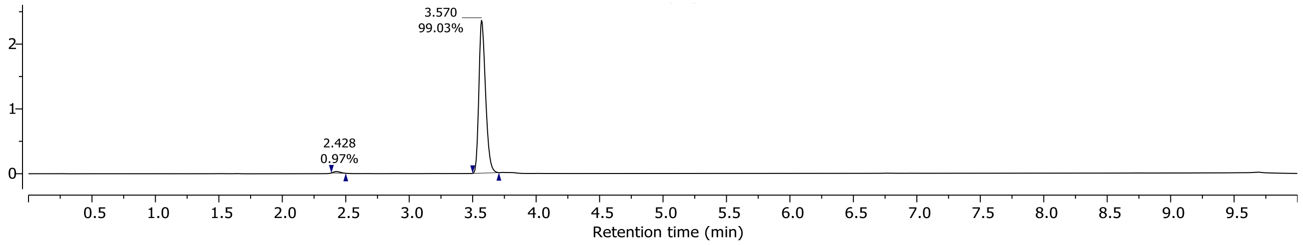

Figure S98. LCMS chromatogram of compound **26** at 254 nm.

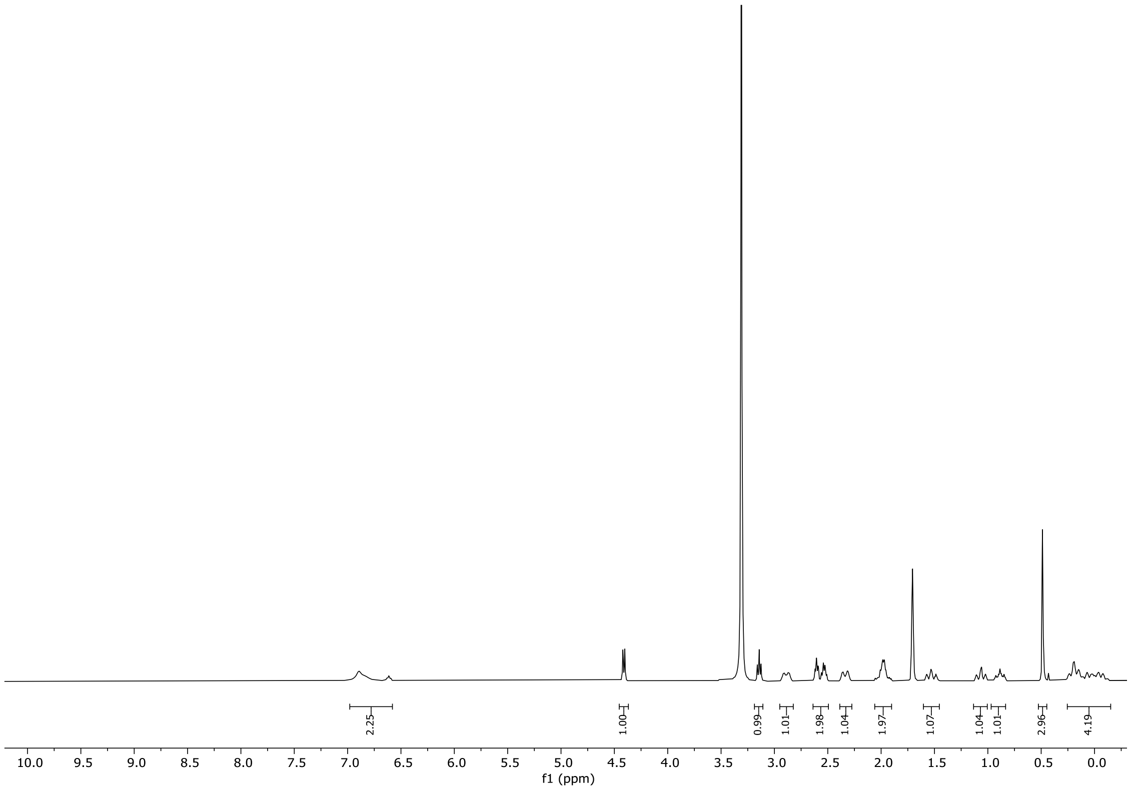

Figure S99. ^{1}H NMR of compound **27**.

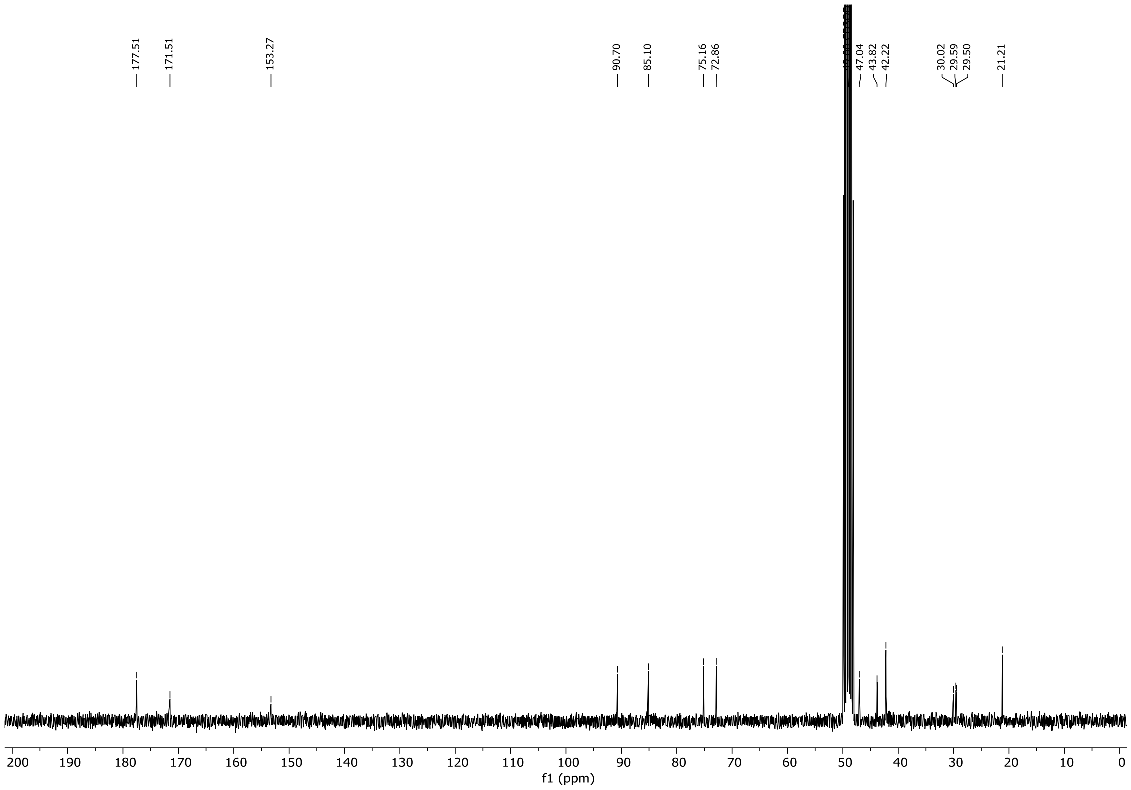

Figure S100. ^{13}C NMR of compound **27.**

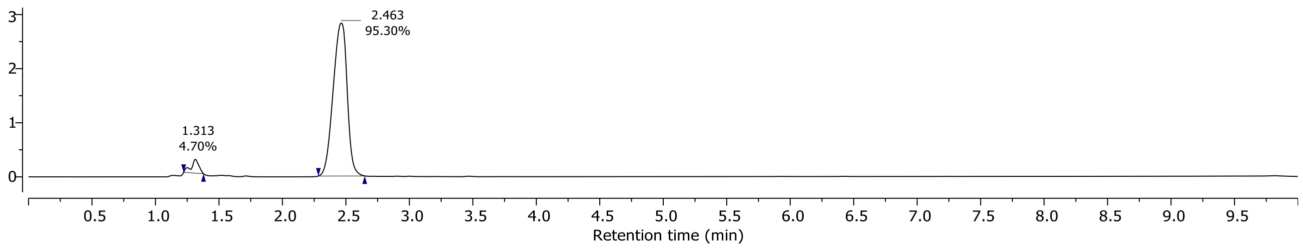

Figure S101. LCMS chromatogram of compound **27** at 254 nm.

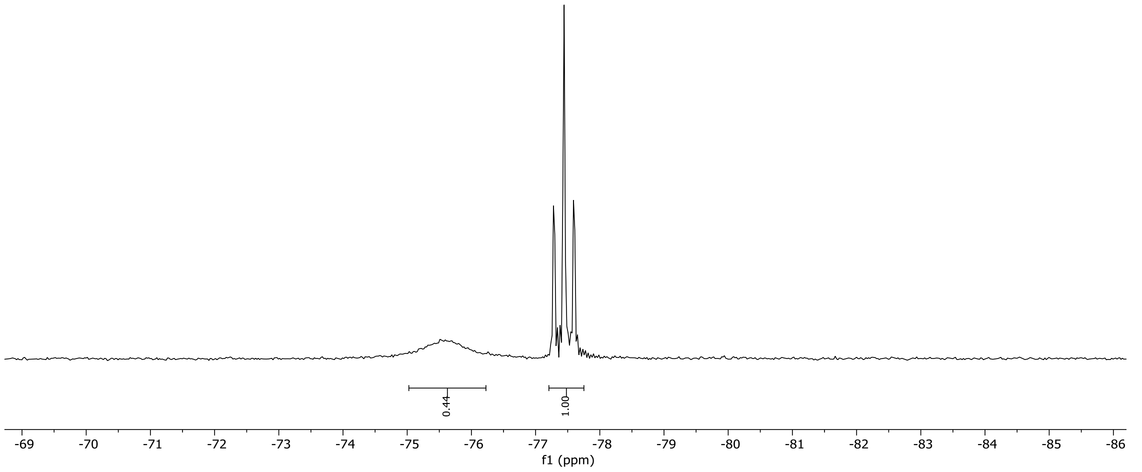

Figure S102. ^{19}F NMR of compound **27**.

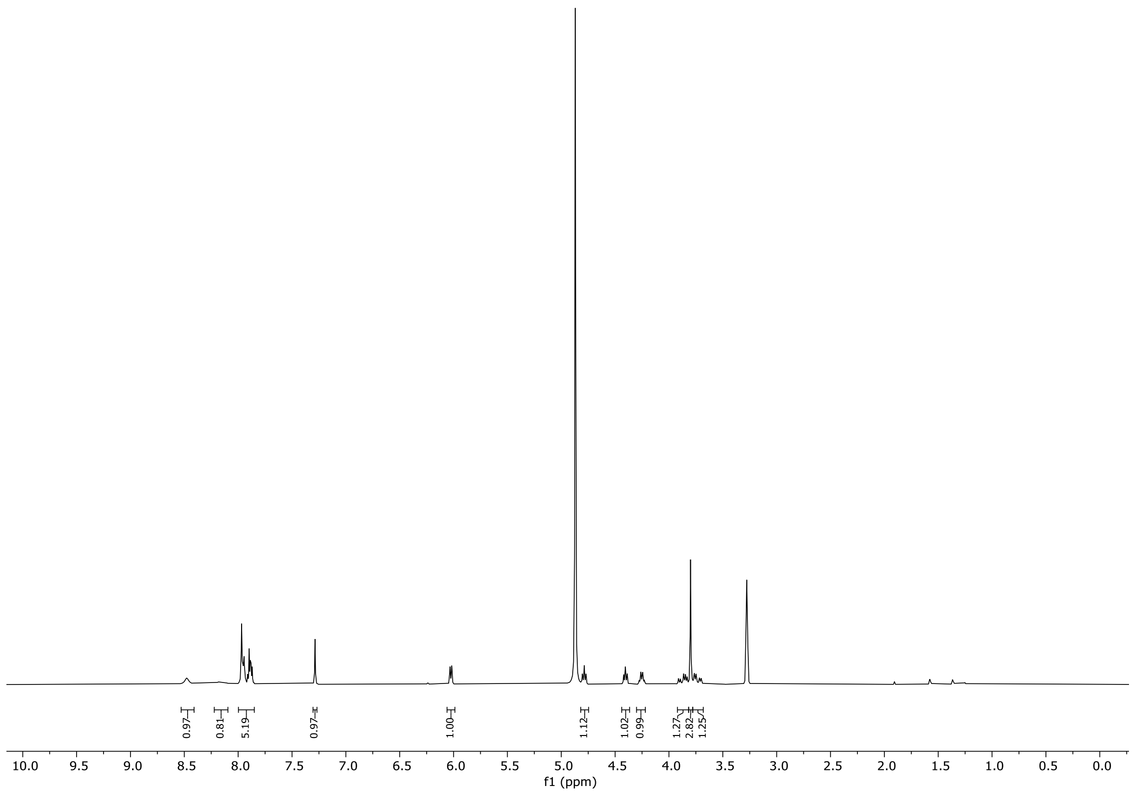

Figure S103. ^{1}H NMR of compound **28**.

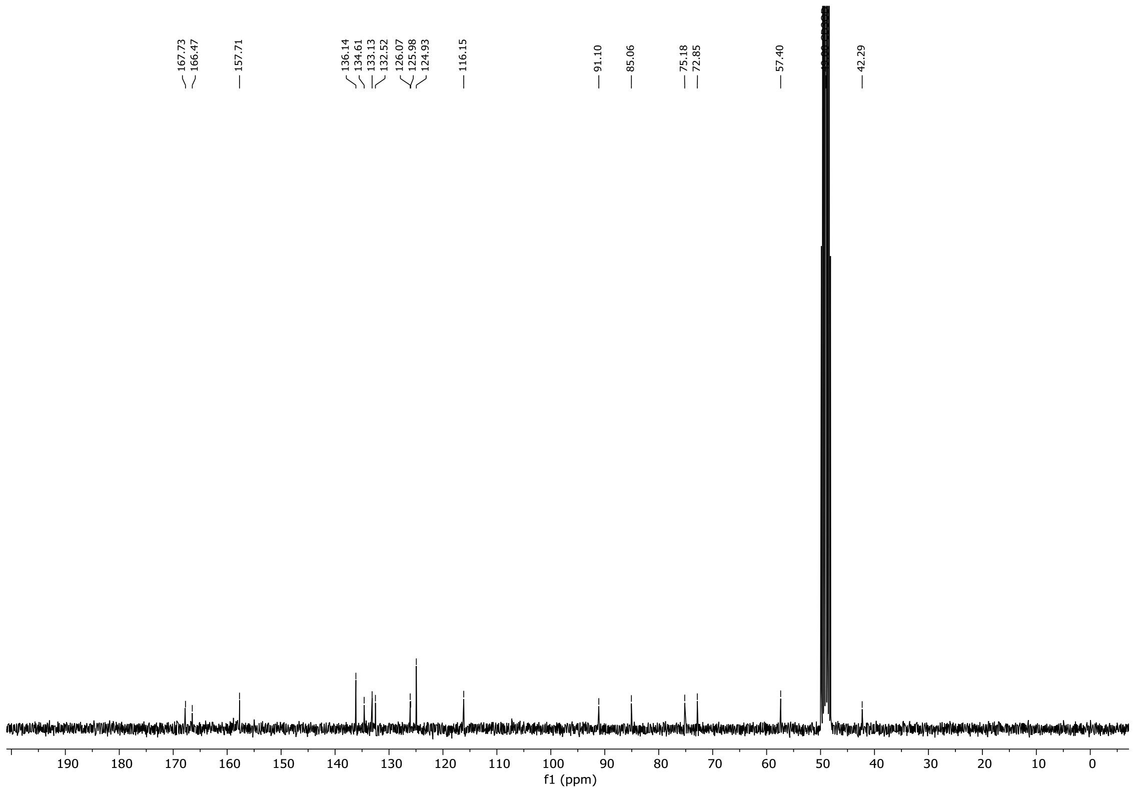

Figure S104. ^{13}C NMR of compound **28.**

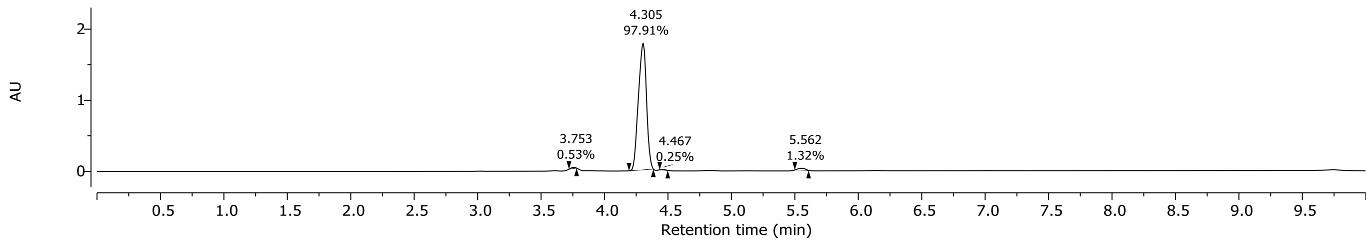

Figure S105. LCMS chromatogram of compound **28** at 254 nm.

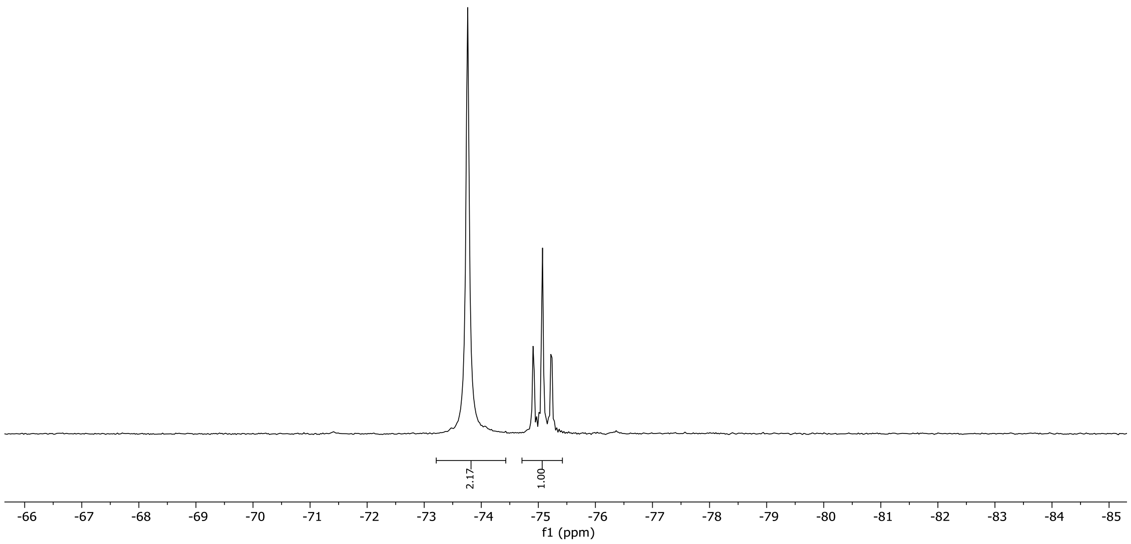

Figure S106. ^{19}F NMR of compound **28**.

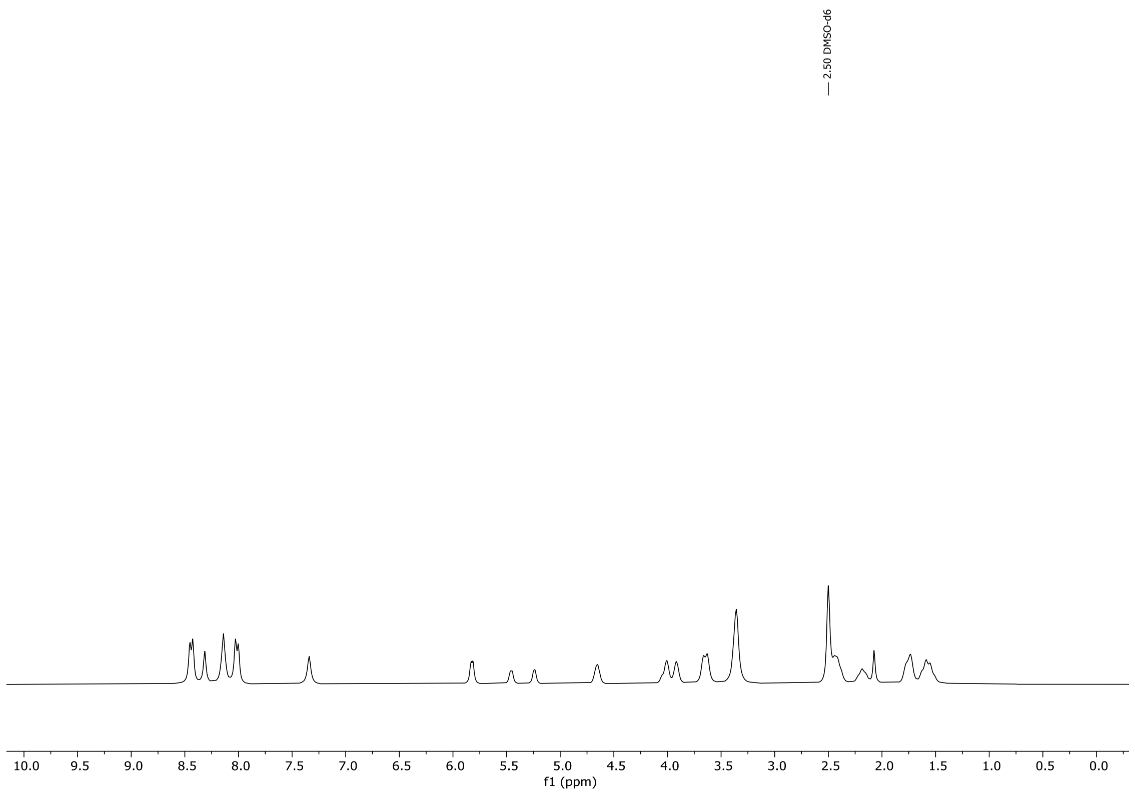

Figure S107. ^{1}H NMR of compound **29**.

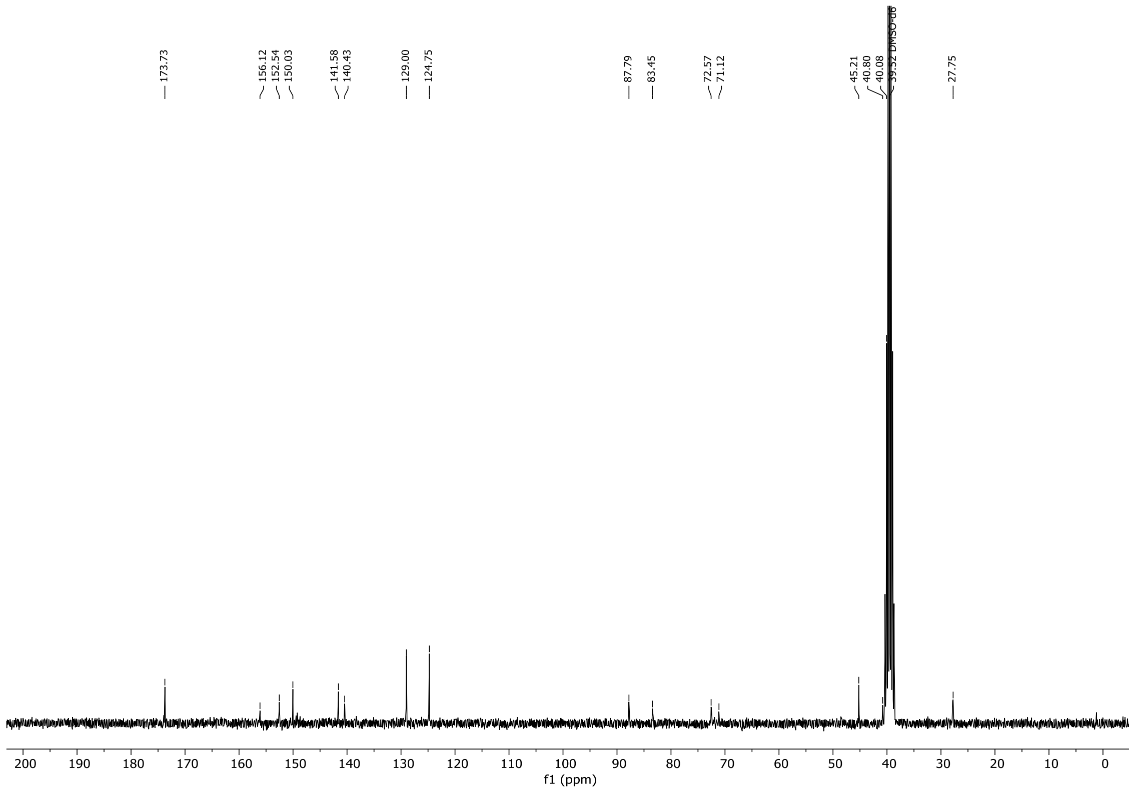

Figure S108. ^{13}C NMR of compound **29.**

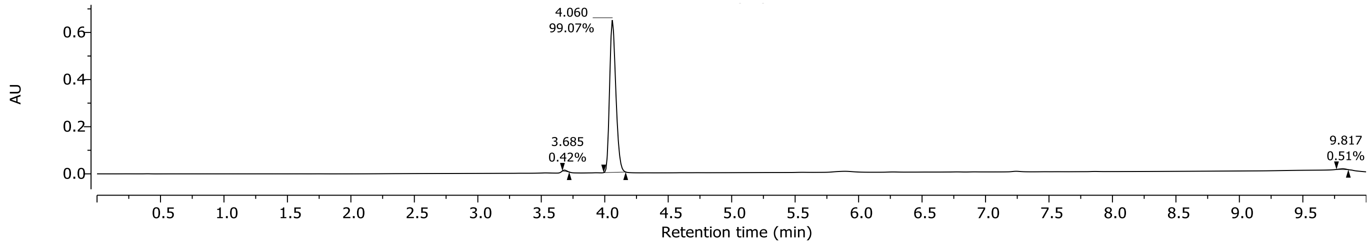

Figure S109. LCMS chromatogram of compound **29** at 254 nm.

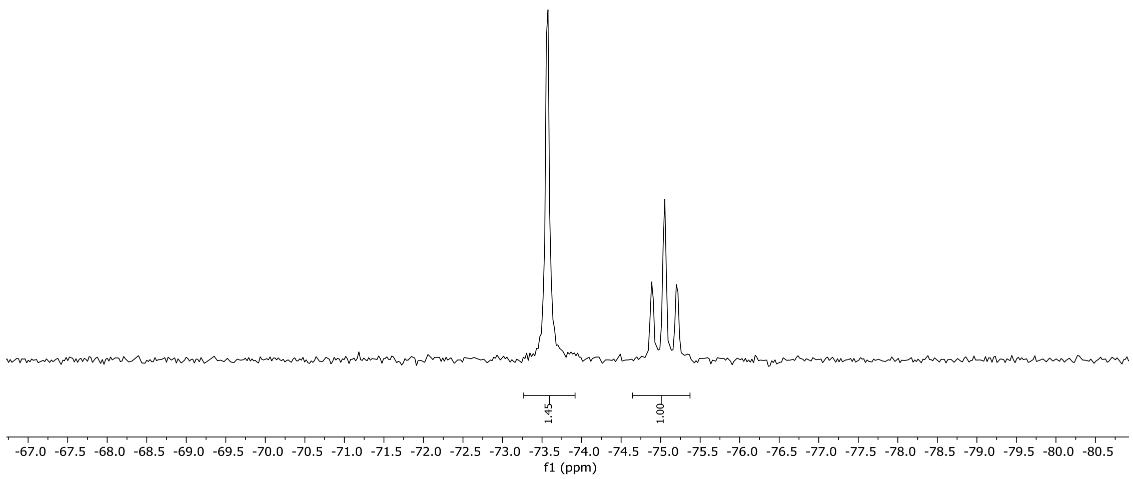

Figure S110. ^{19}F NMR of compound **29**.

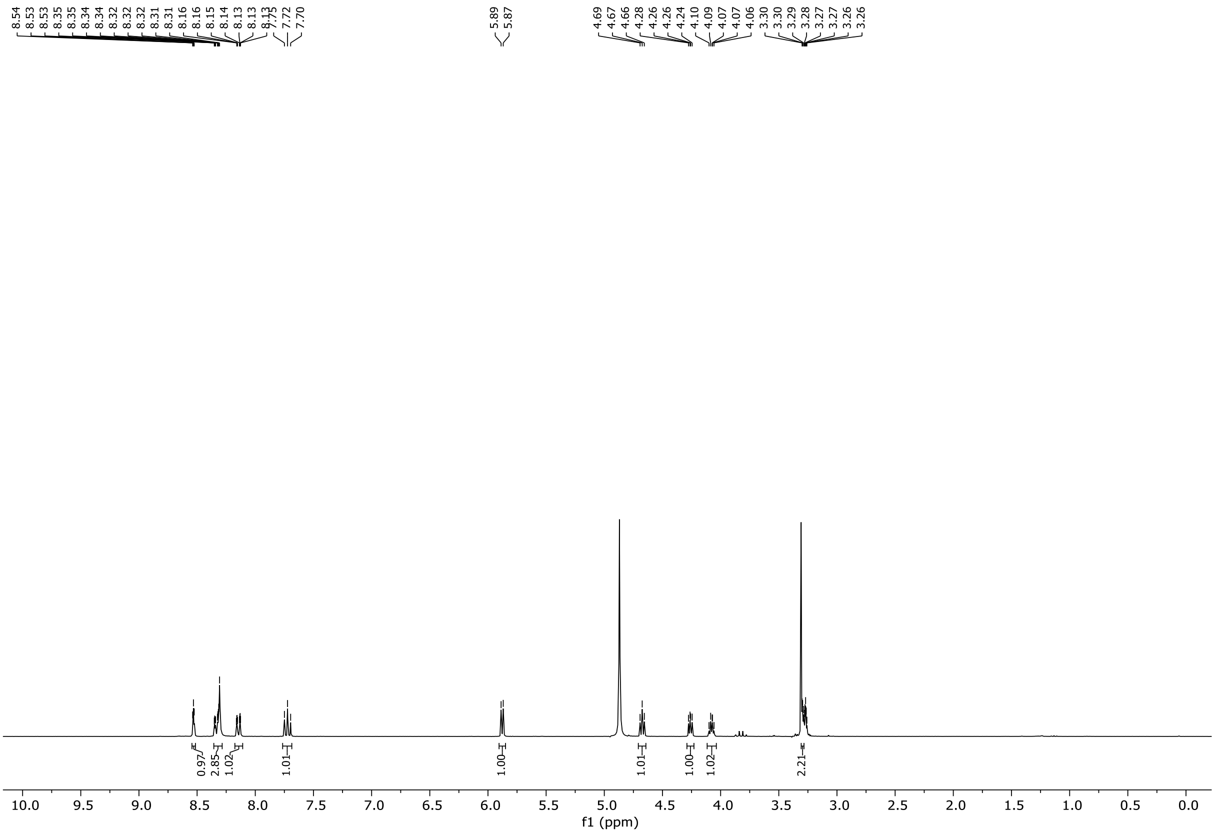

Figure S111. ^{1}H NMR of compound ***31***.

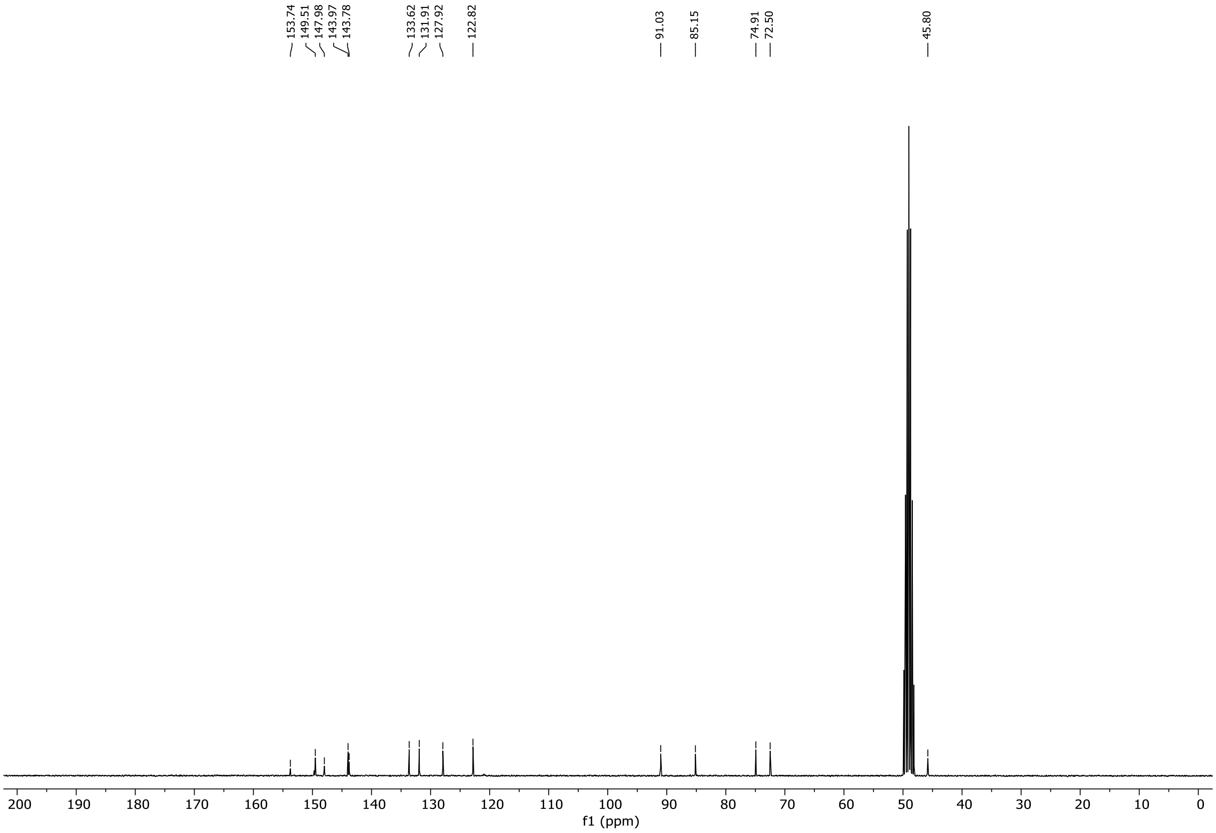

Figure S112. ^{13}C NMR of compound ***31***.

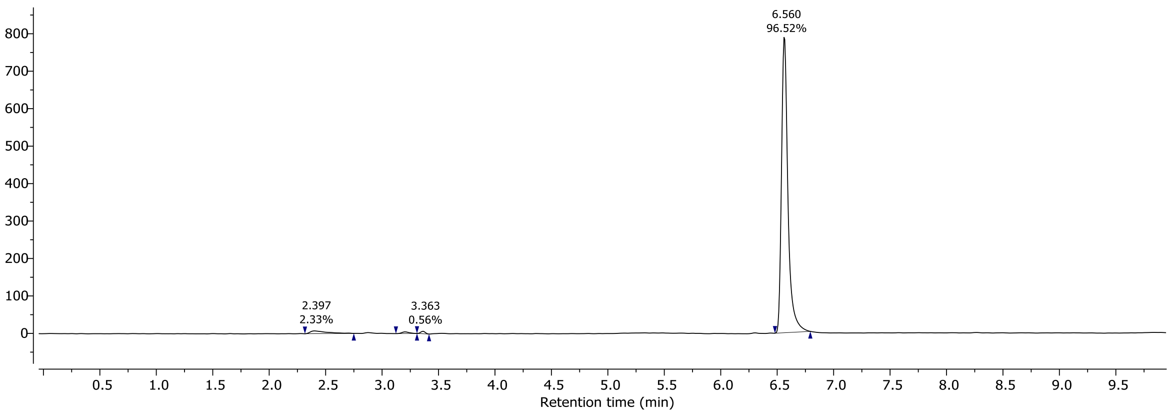

Figure S113. LCMS chromatogram of compound ***31*** at 254 nm.

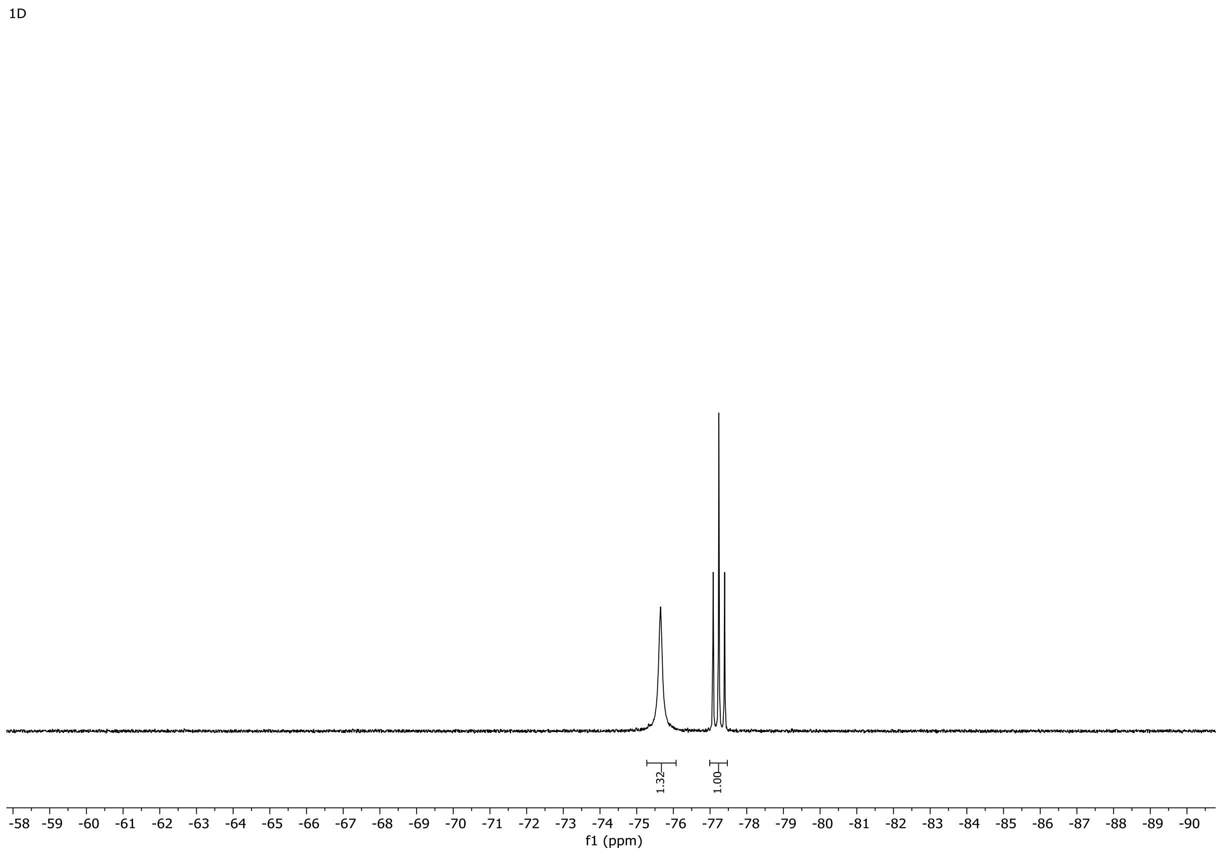

Figure S114. ^{19}F NMR of compound ***31***.

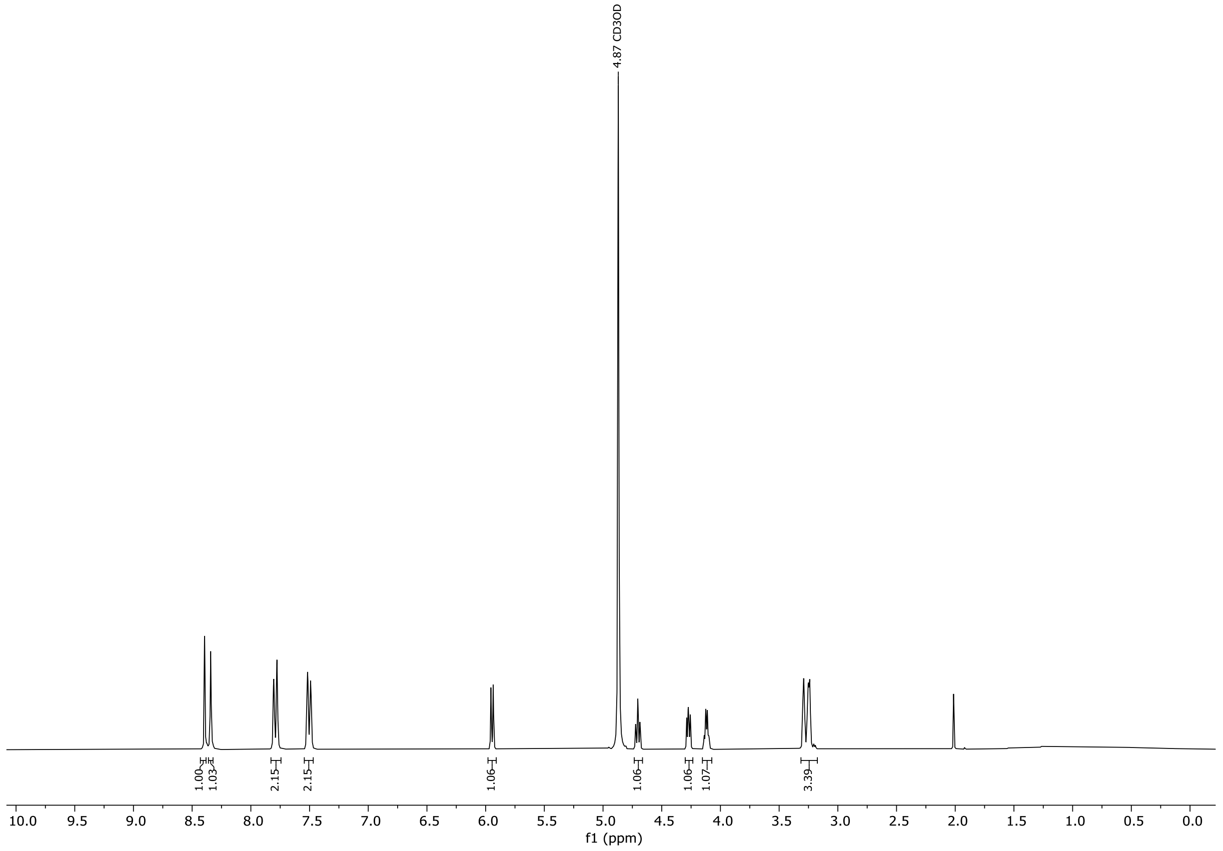

Figure S115. ^{1}H NMR of compound ***32***.

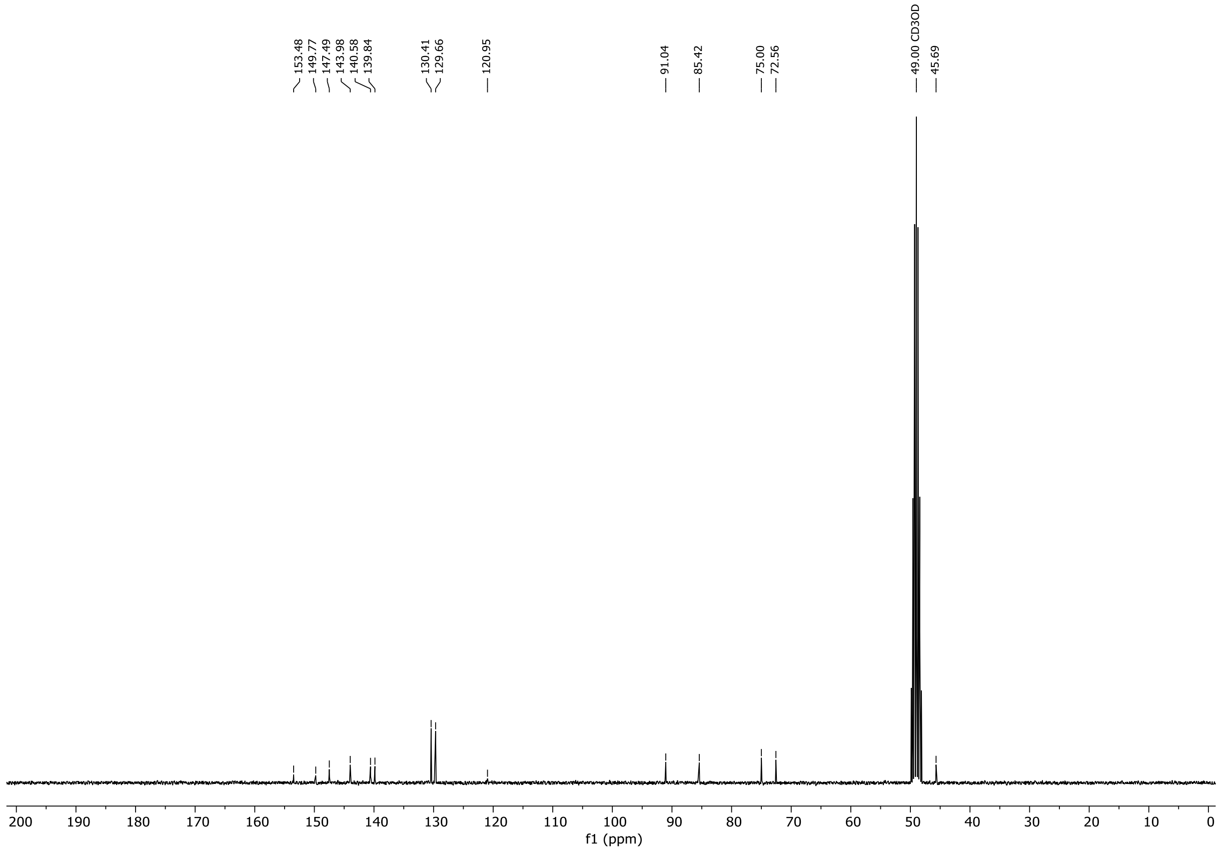

Figure S116. ^{13}C NMR of compound ***32***.

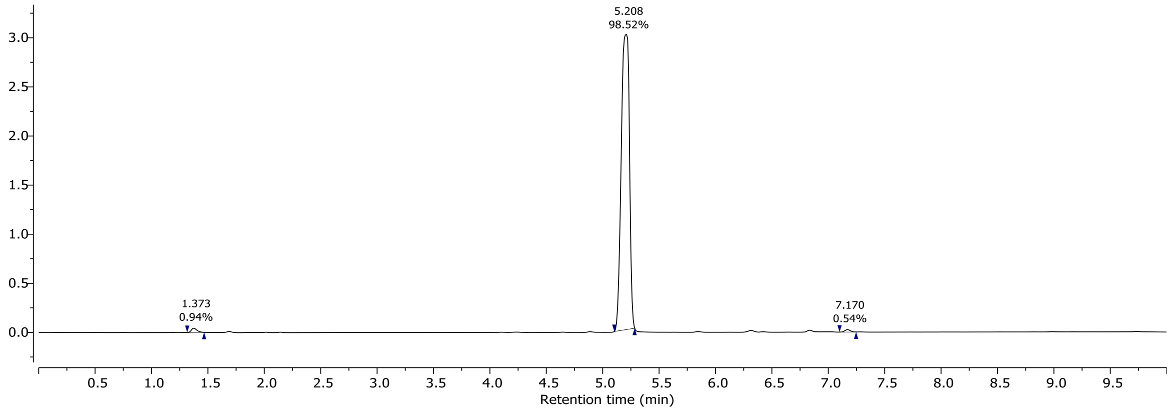

Figure S117. LCMS chromatogram of compound ***32*** at 254 nm.

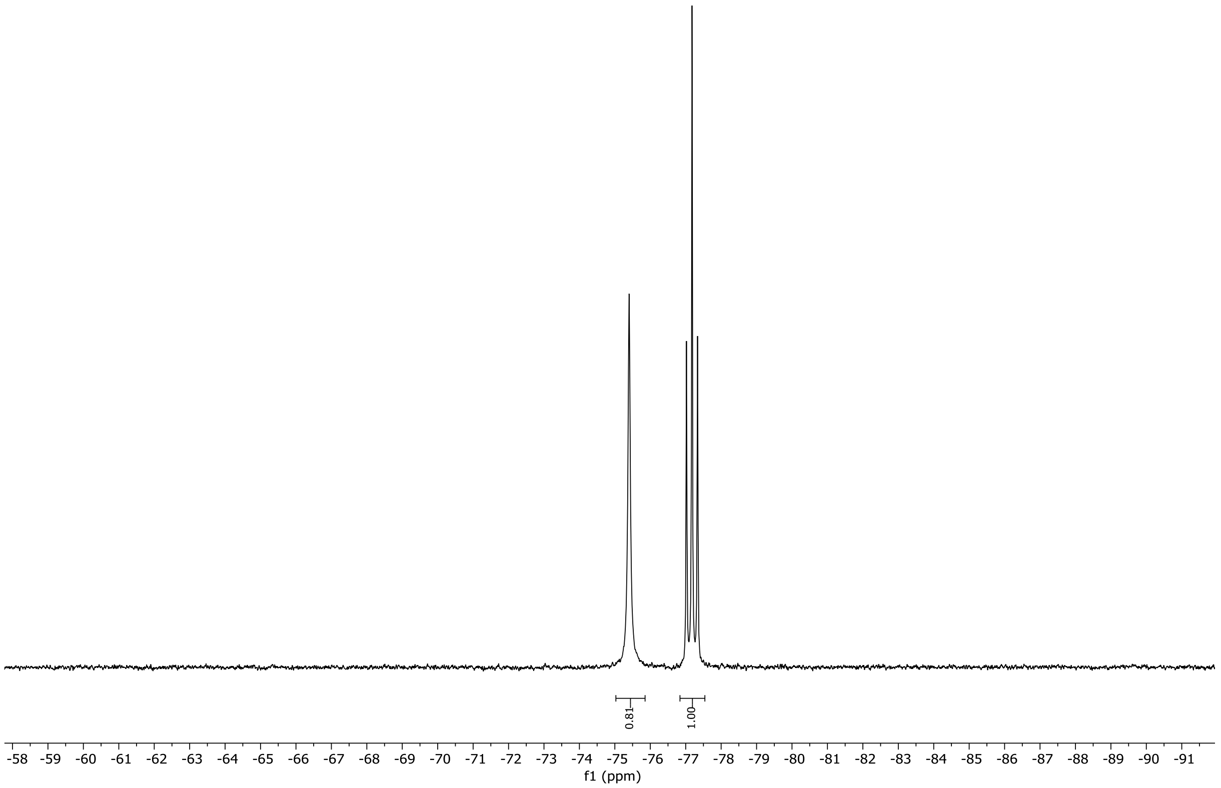

Figure S118. ^{19}F NMR of compound **32**.

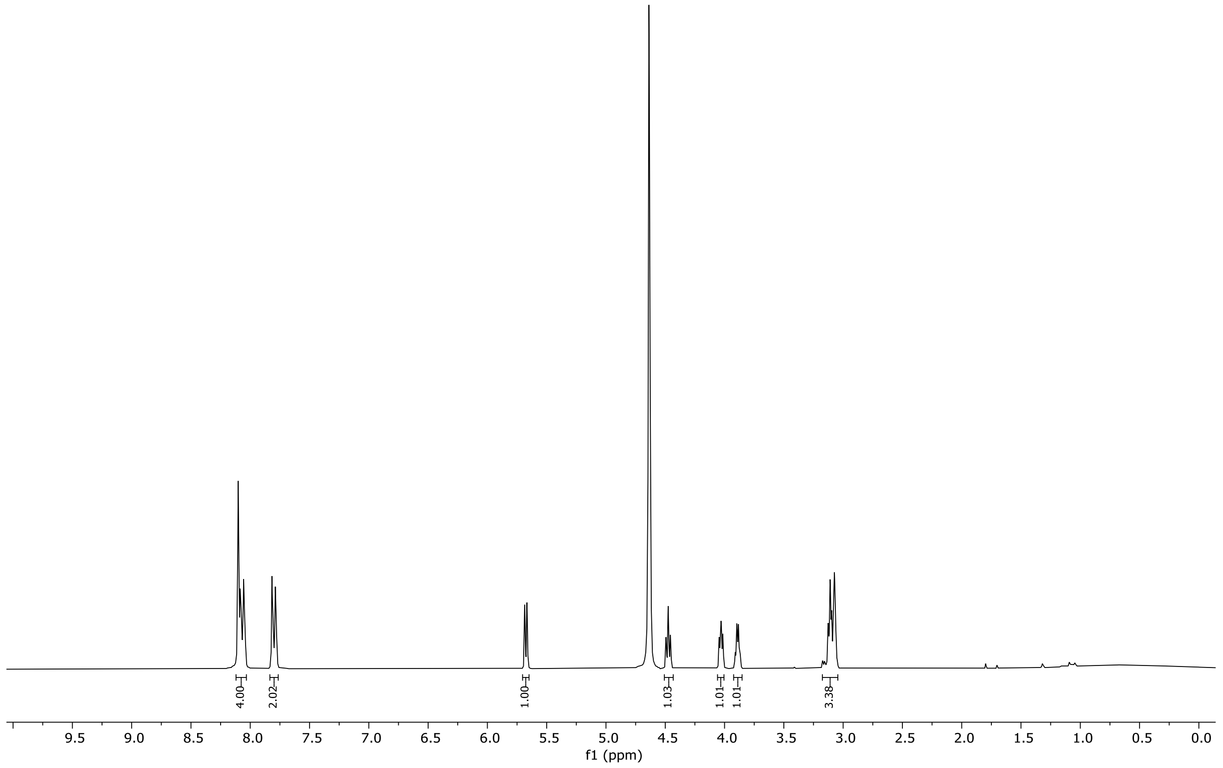

Figure S119. ^{1}H NMR of compound ***33***.

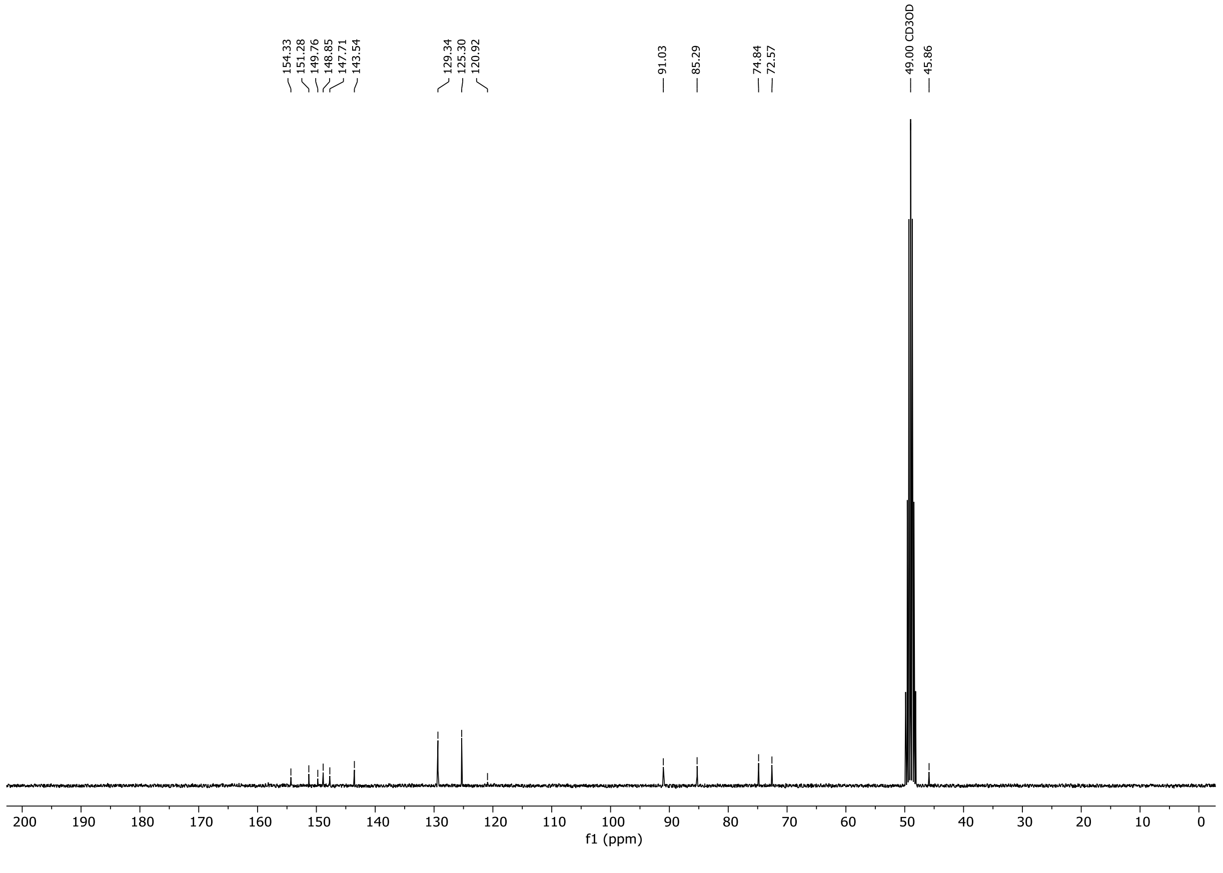

Figure S120. ^{13}C NMR of compound ***33***.

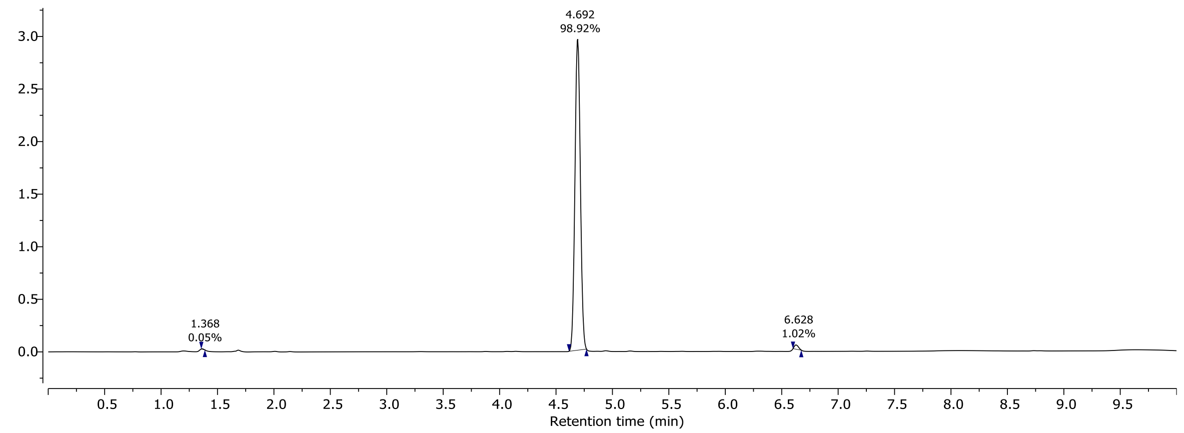

Figure S121. LCMS chromatogram of compound ***33*** at 254 nm.

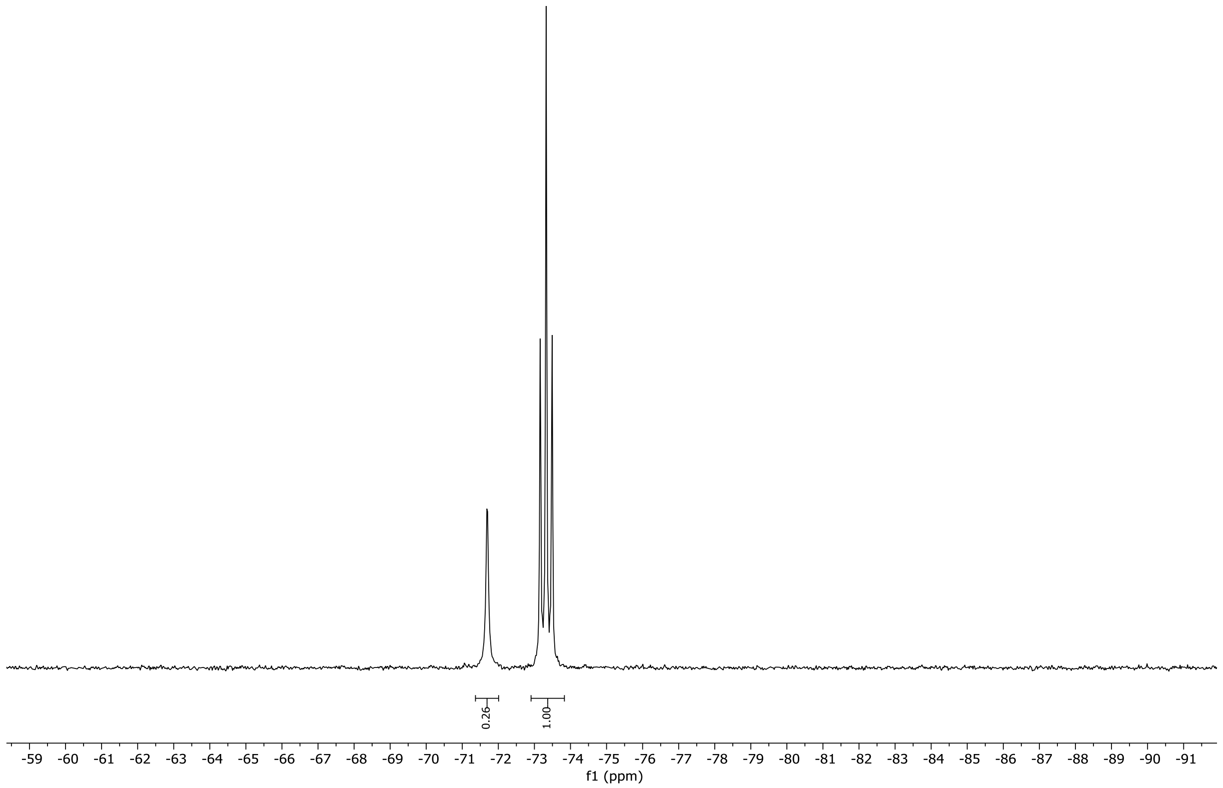

Figure S122. ^{19}F NMR of compound ***33***.

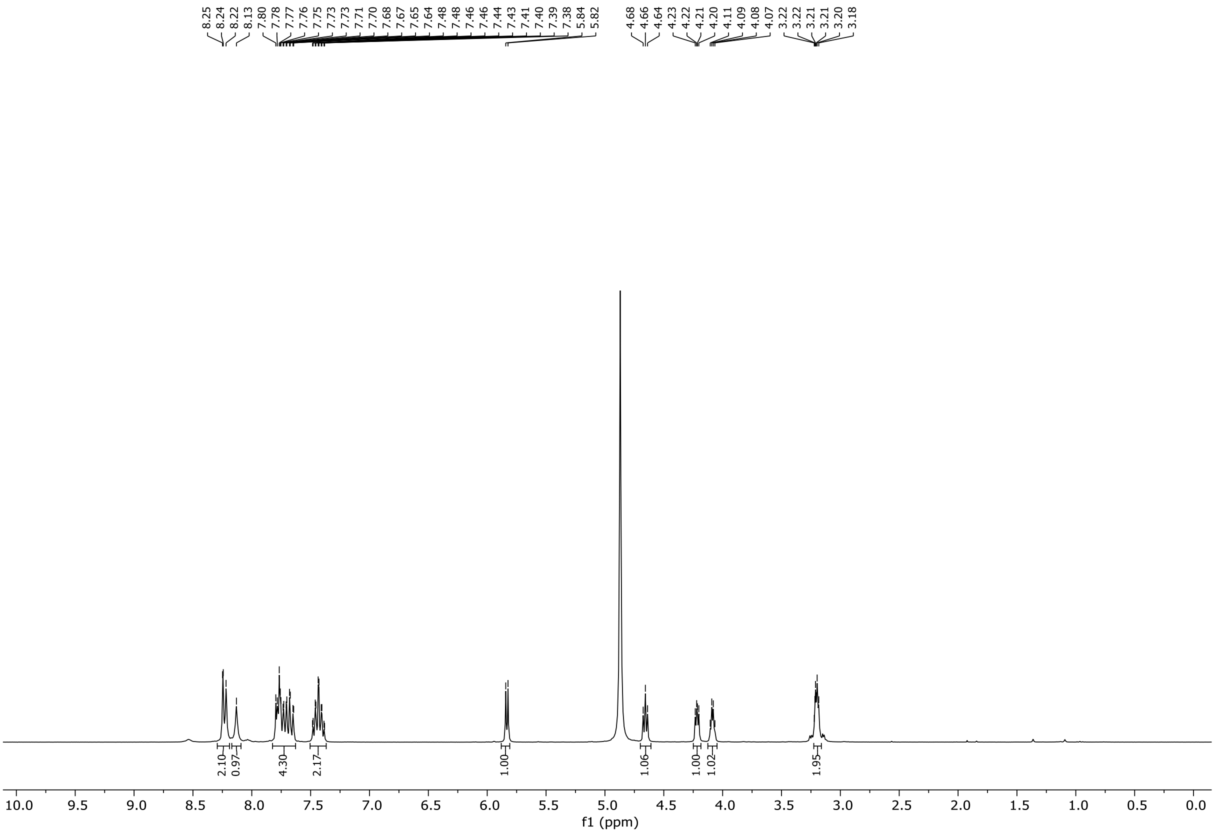

Figure S123. ^{1}H NMR of compound ***34***.

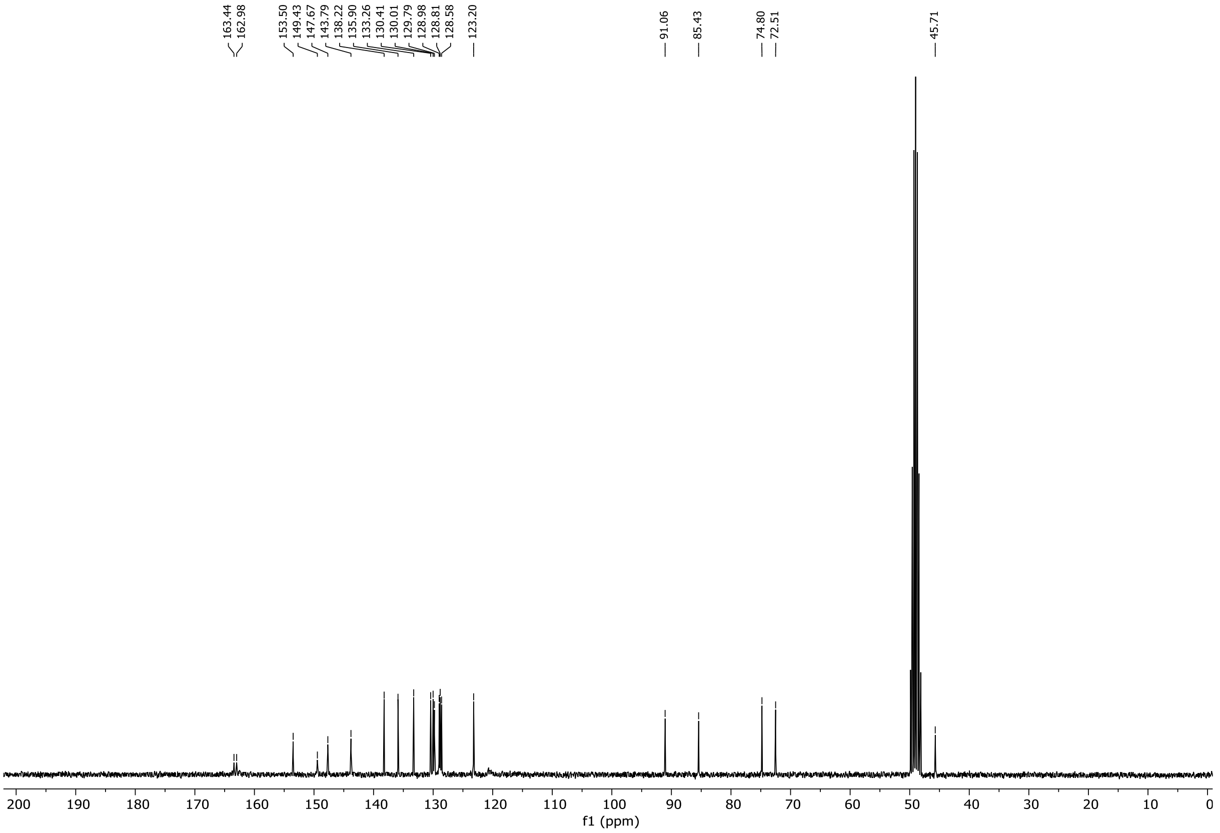

Figure S124. ^{13}C NMR of compound ***34***.

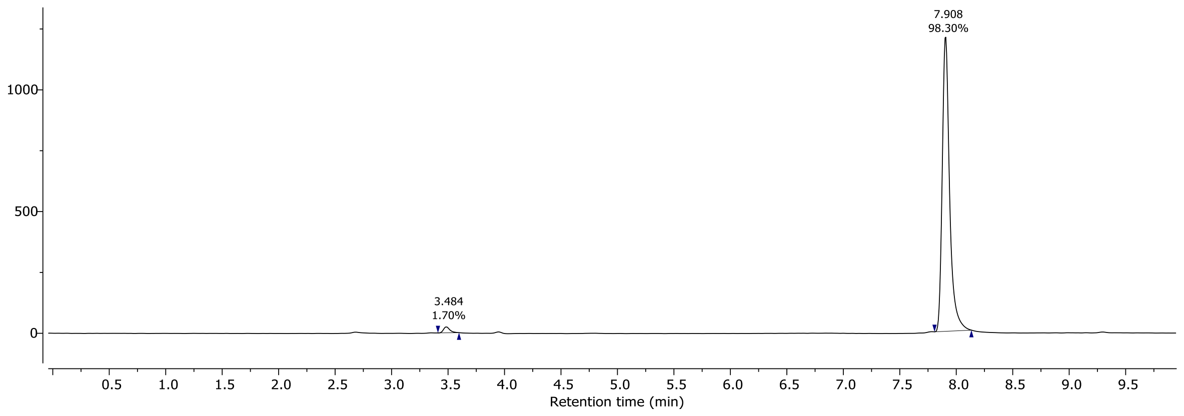

Figure S125. LCMS chromatogram of compound ***34*** at 254 nm.

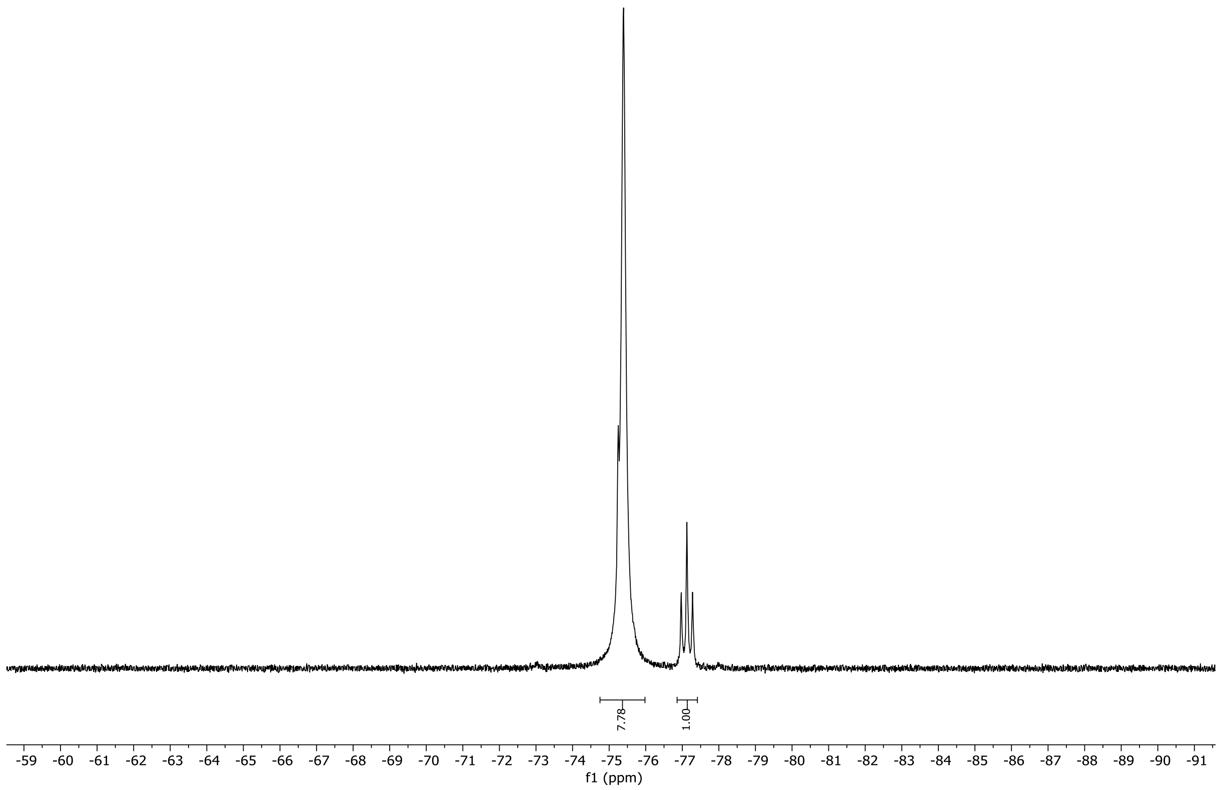

Figure S126. ^{19}F NMR of compound ***34*.**

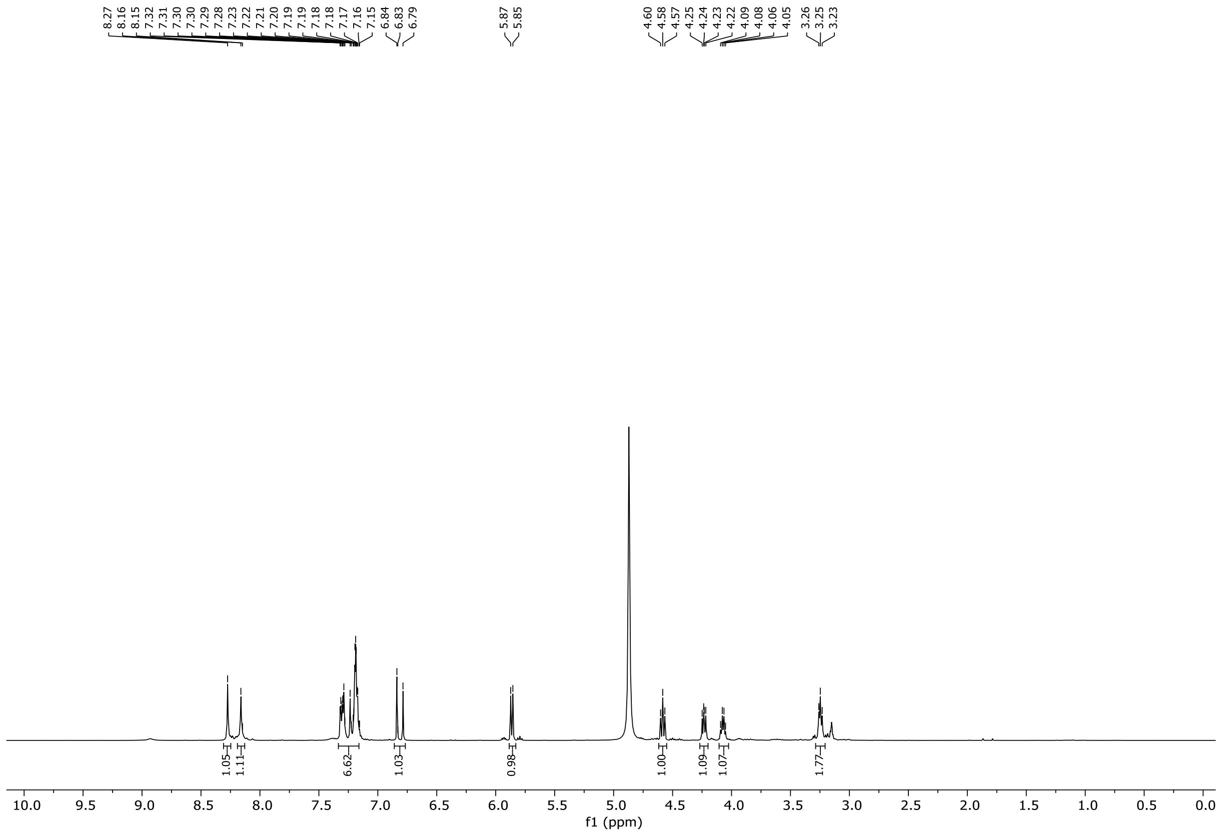

Figure S127. ^{1}H NMR of compound ***35***.

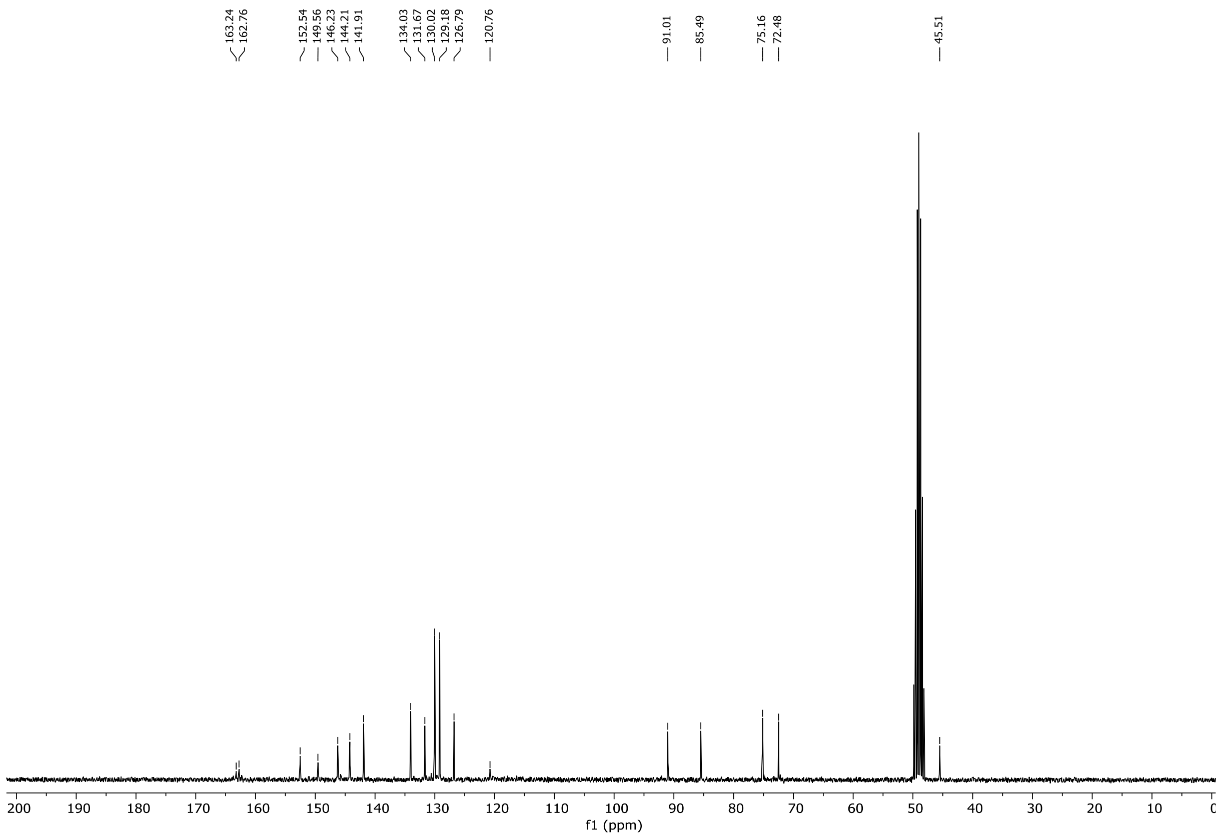

Figure S128. ^{13}C NMR of compound ***35***.

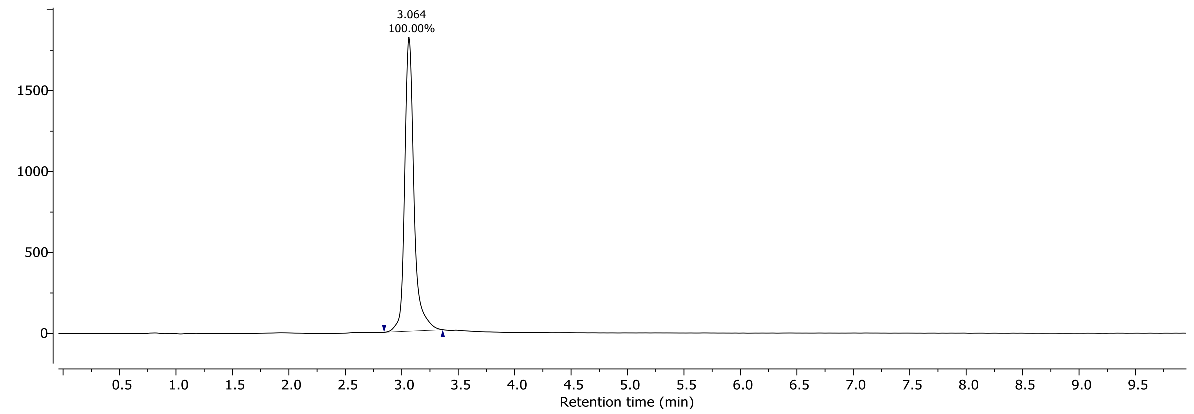

Figure S129. LCMS chromatogram of compound ***35*** at 254 nm.

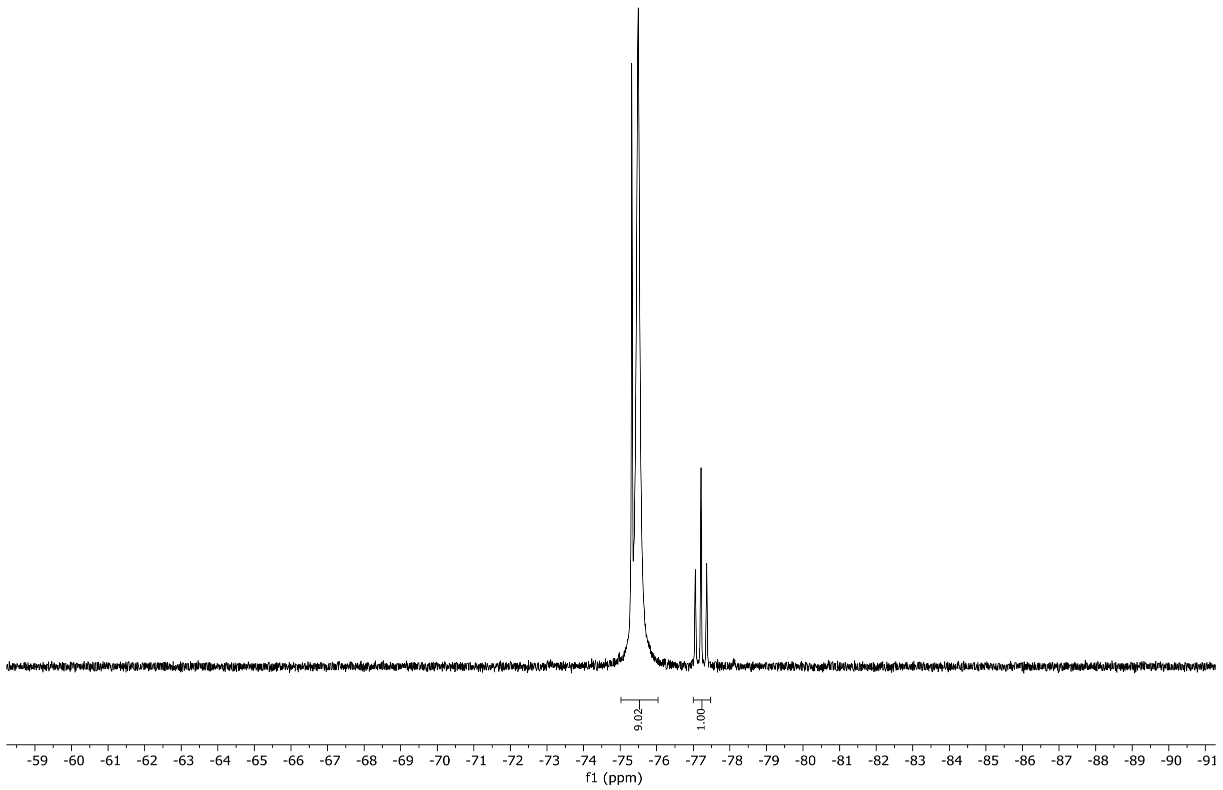

Figure S130. ^{19}F NMR of compound ***35*.**

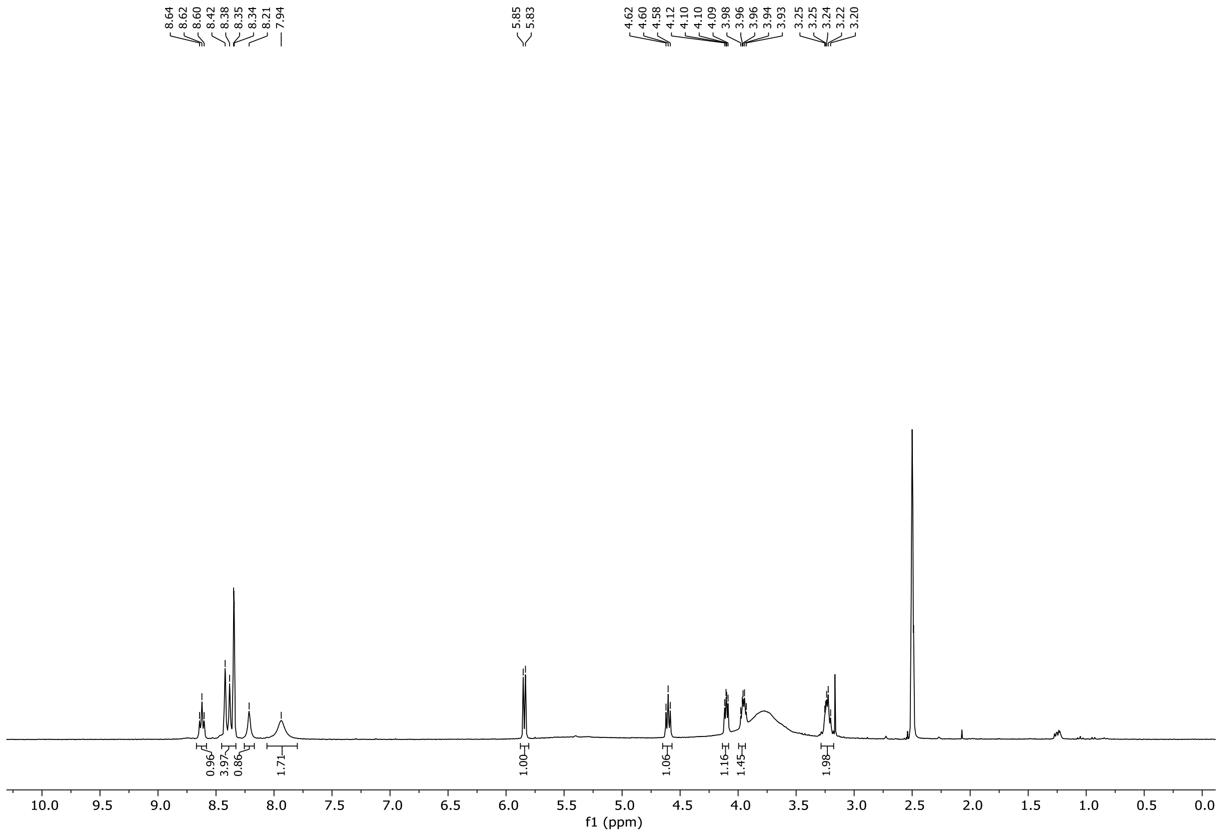

Figure S131. ^{1}H NMR of compound ***36***.

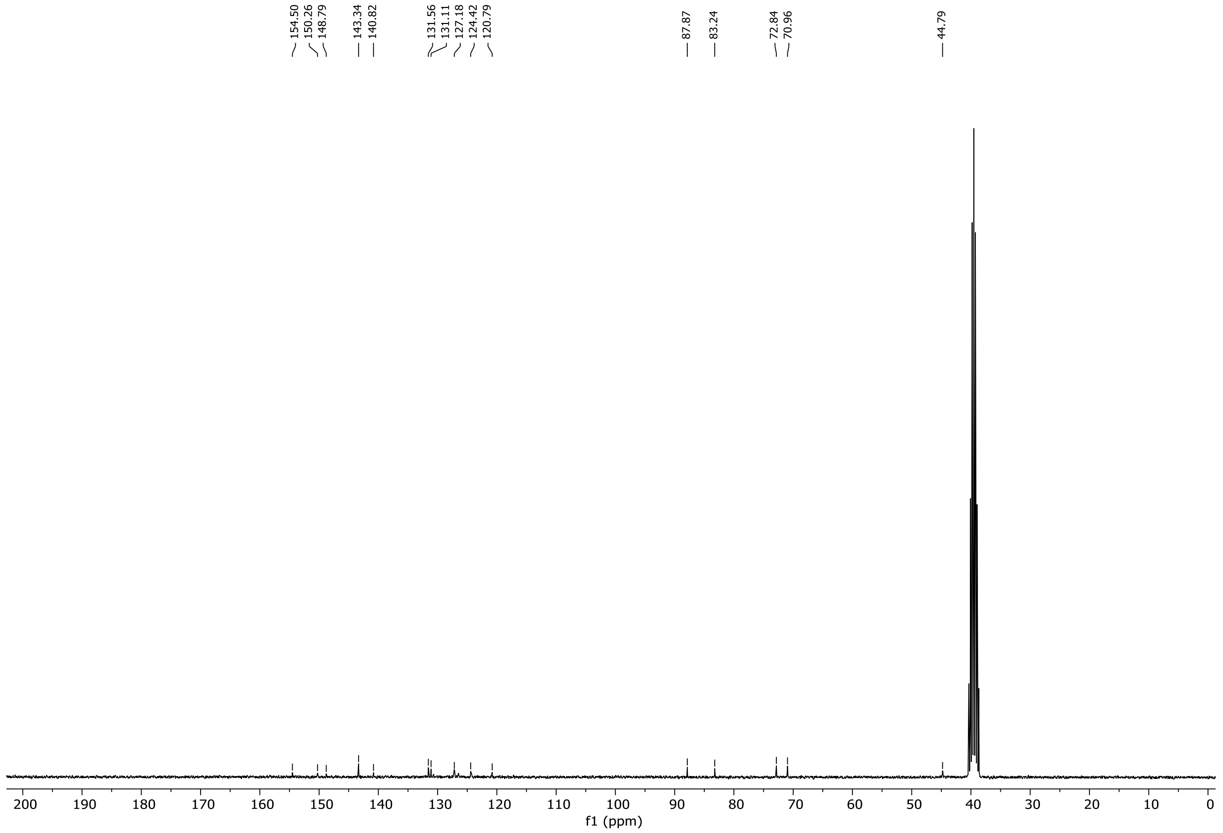

Figure S132. ^{13}C NMR of compound ***36***.

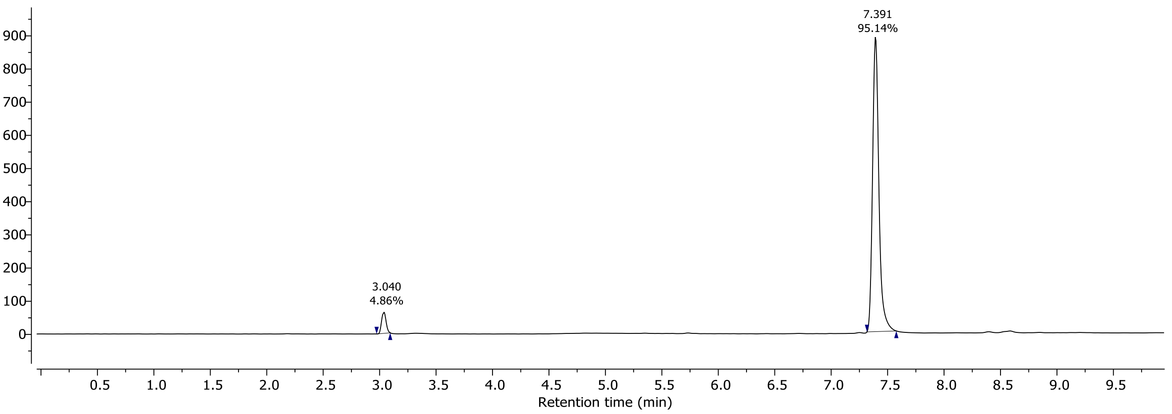

Figure S133. LCMS chromatogram of compound ***36*** at 254 nm.

1D

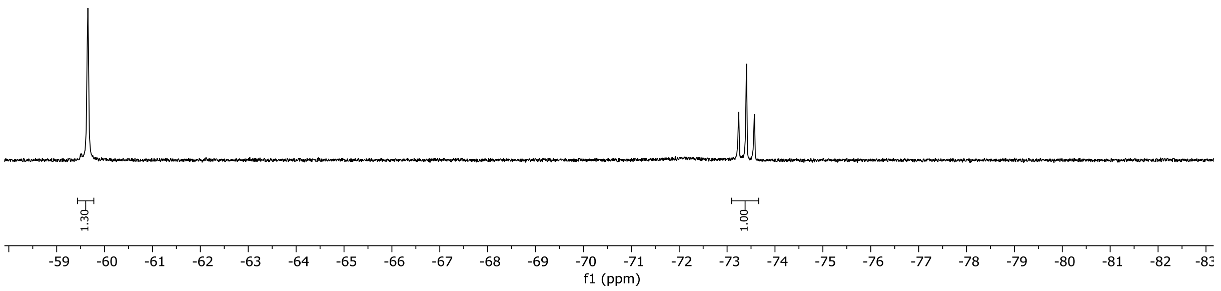

Figure S134. ^{19}F NMR of compound ***36.***

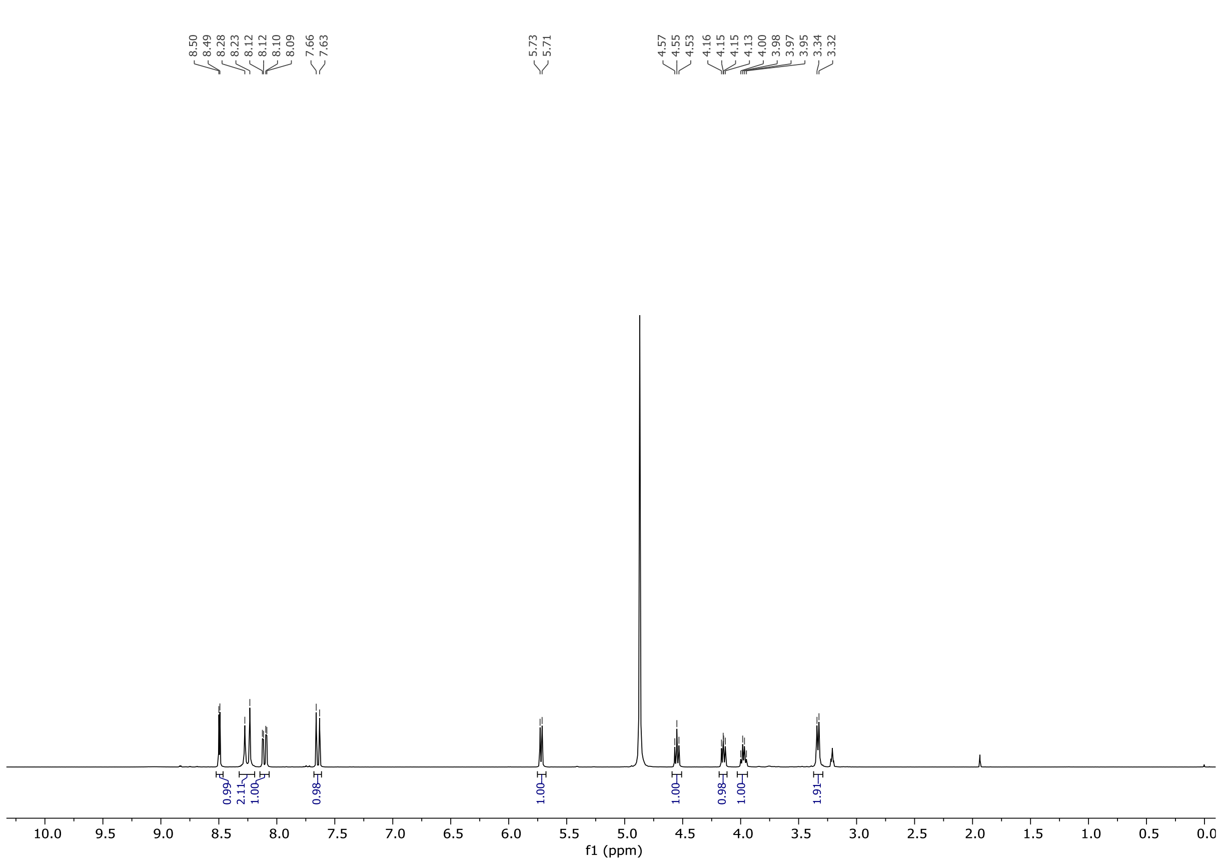

Figure S135. ^{1}H NMR of compound ***37***.

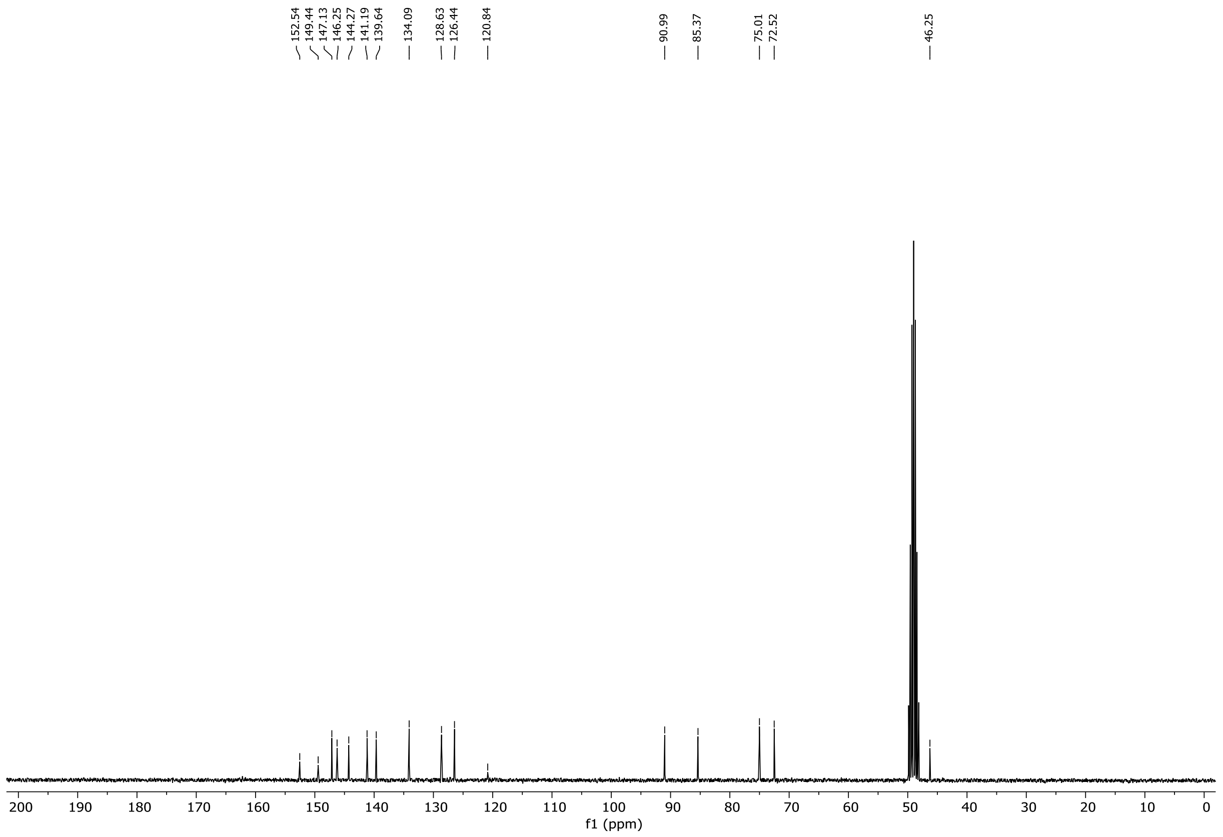

Figure S136. ^{13}C NMR of compound ***37***.

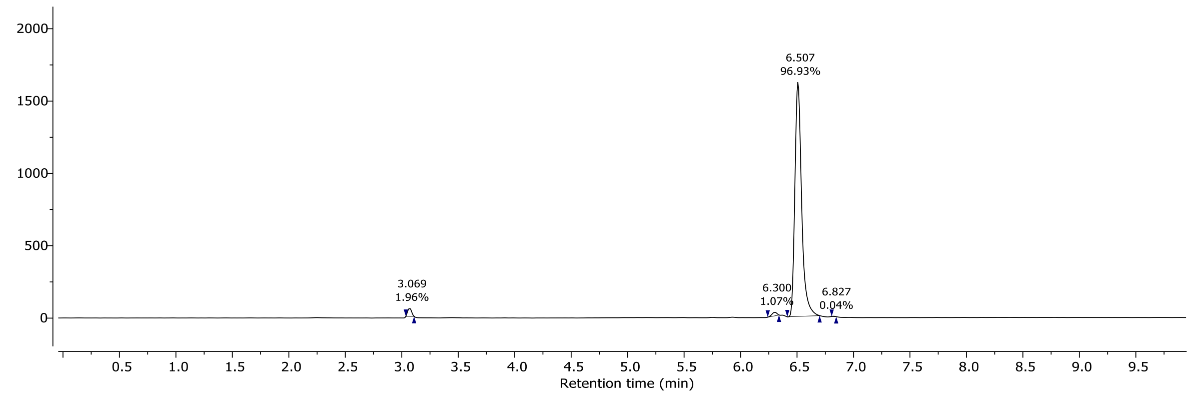

Figure S137. LCMS chromatogram of compound ***37*** at 254 nm.

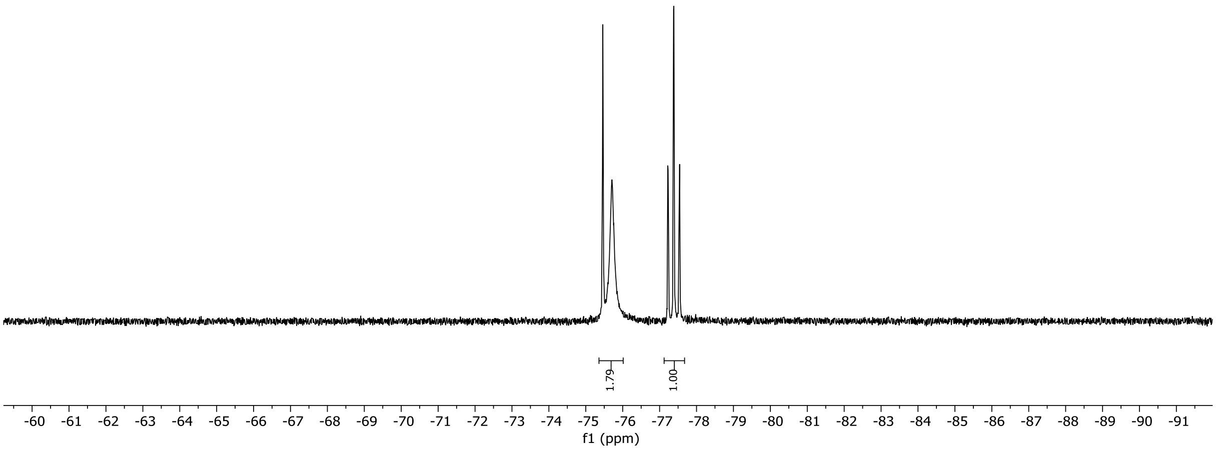

Figure S138. ^{19}F NMR of compound ***37***.

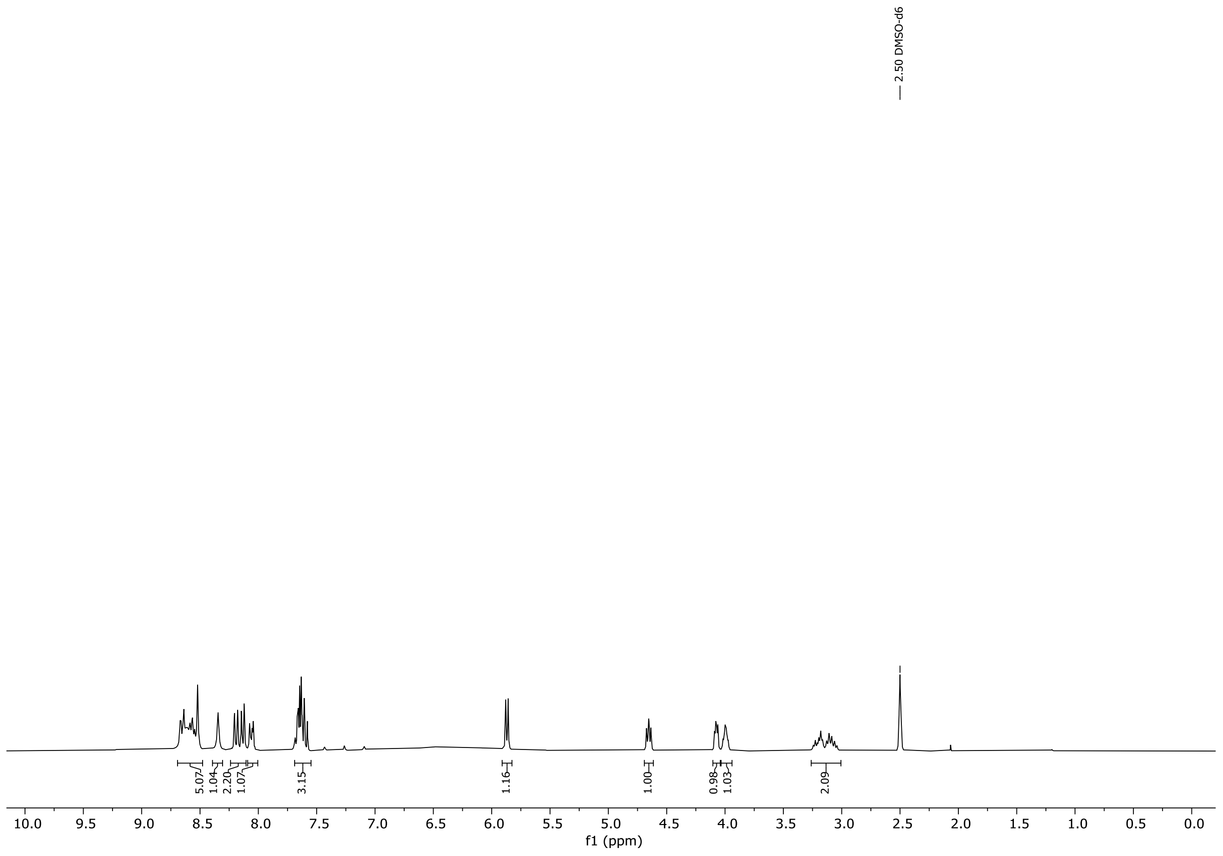

Figure S139. ^{1}H NMR of compound ***38***.

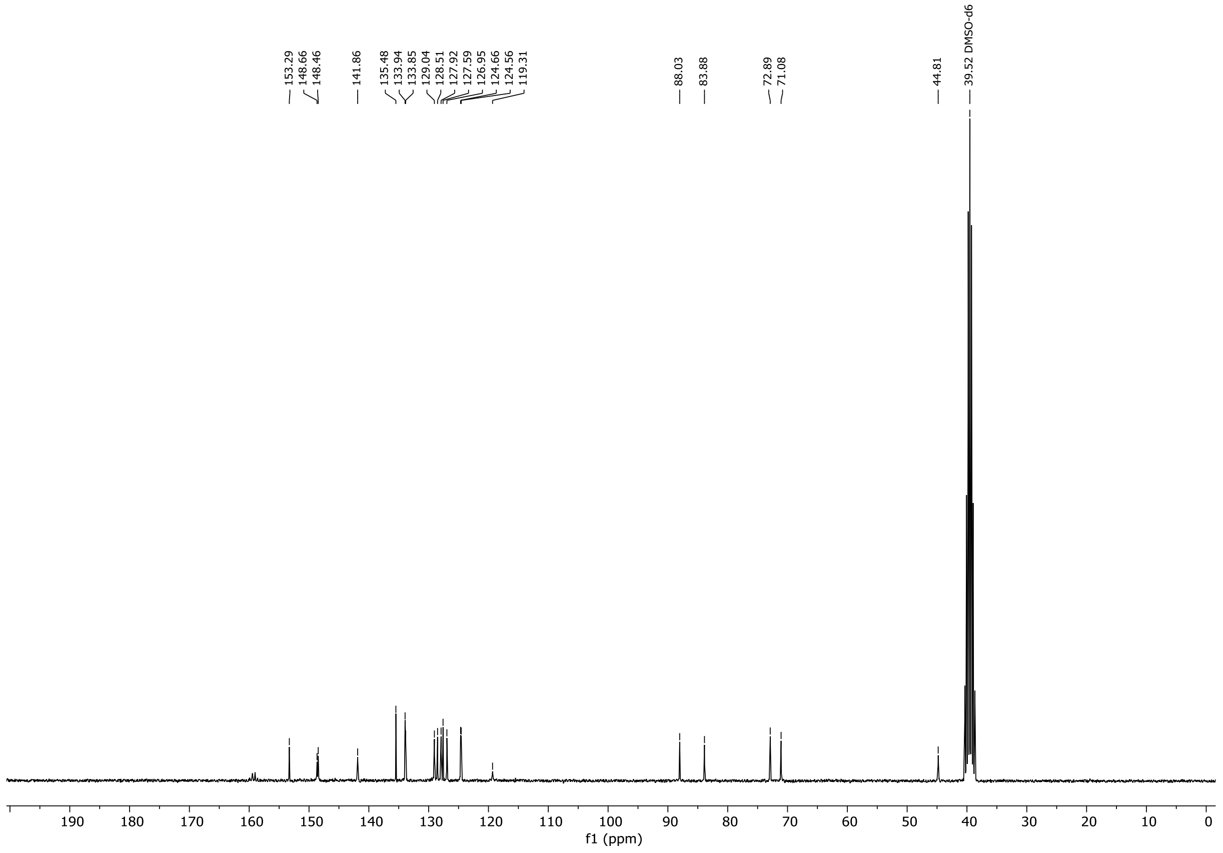

Figure S140. ^{13}C NMR of compound ***38***.

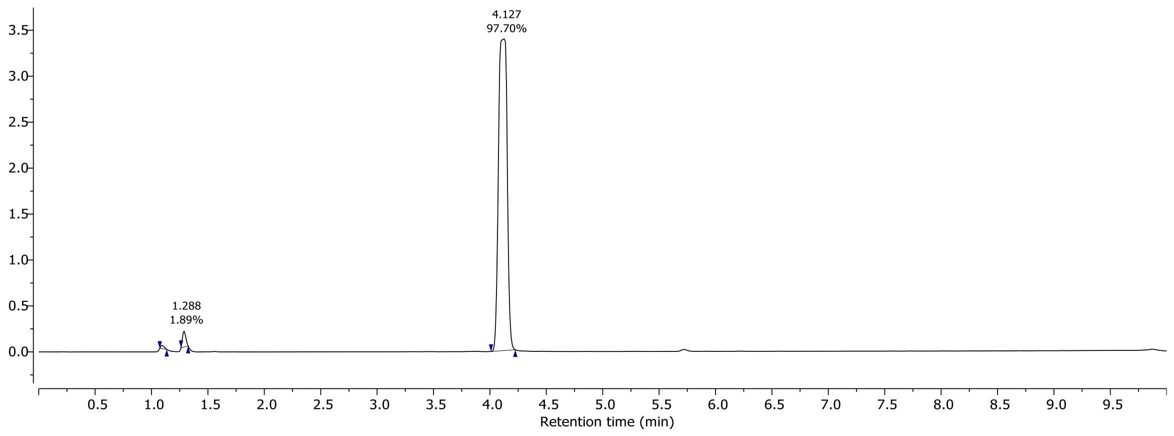

Figure S141. LCMS chromatogram of compound ***38*** at 254 nm.

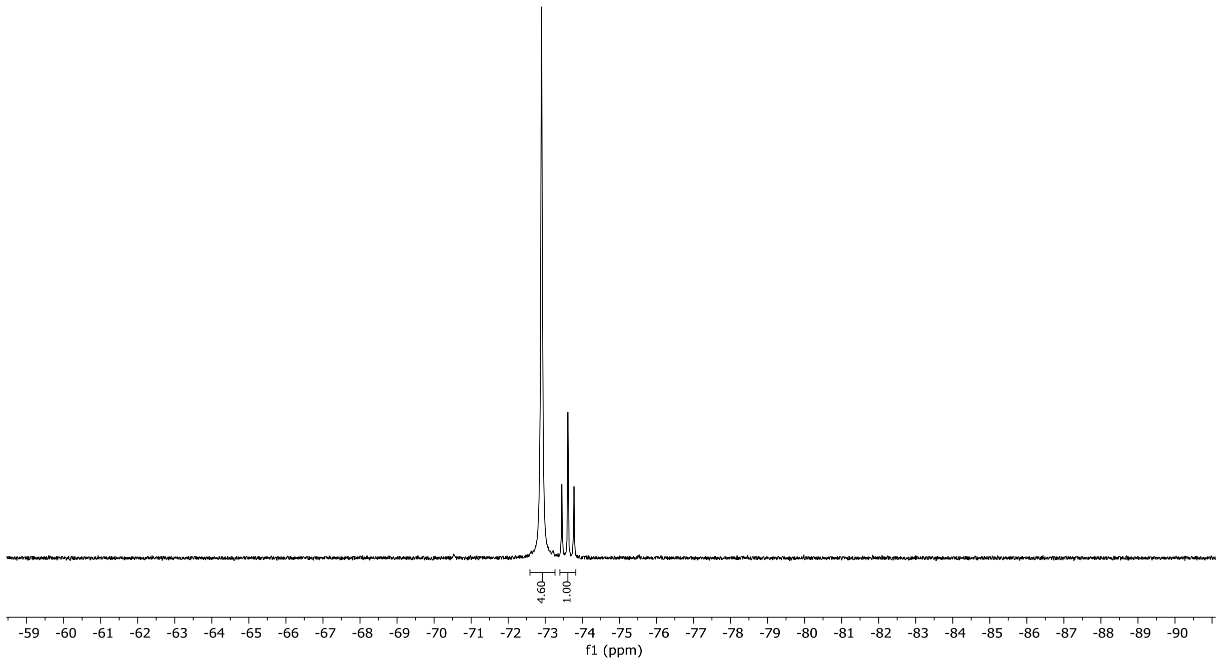

Figure S142. ^{19}F NMR of compound **38**.

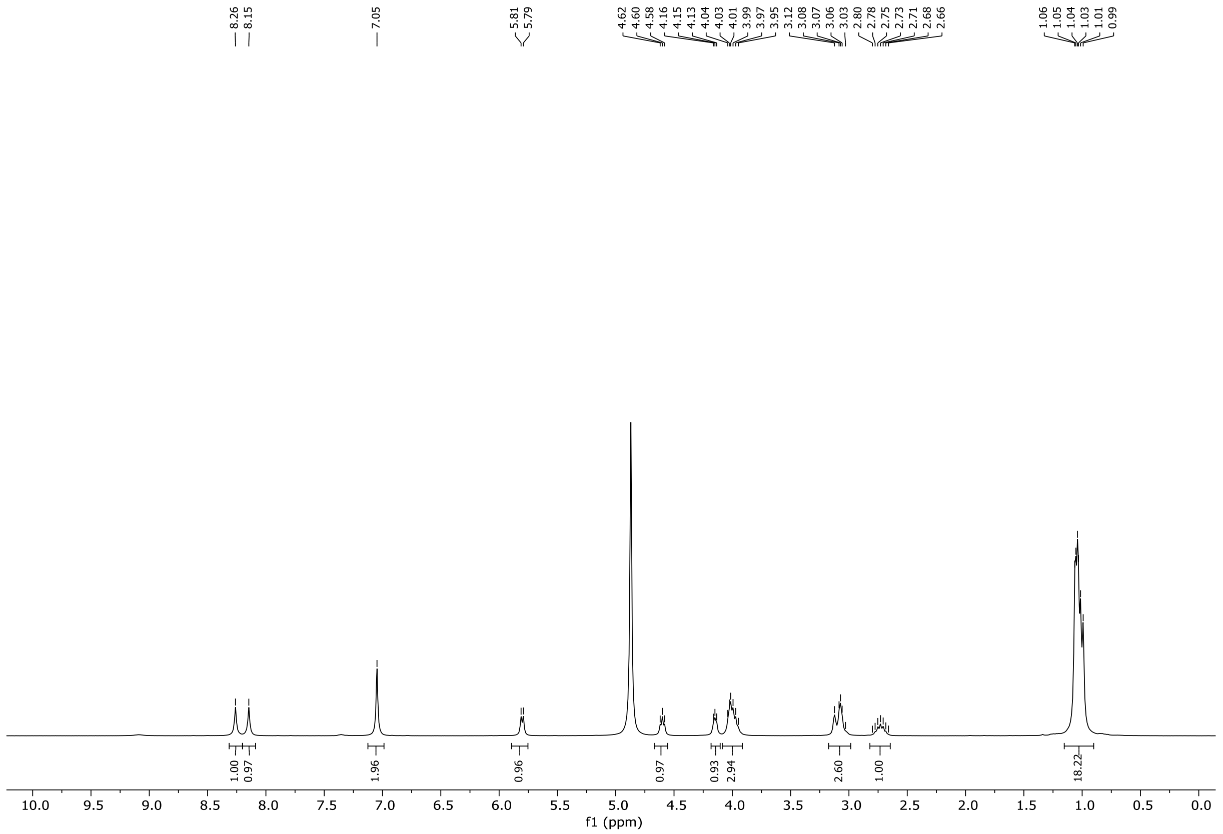

Figure S143. ^{1}H NMR of compound ***39***.

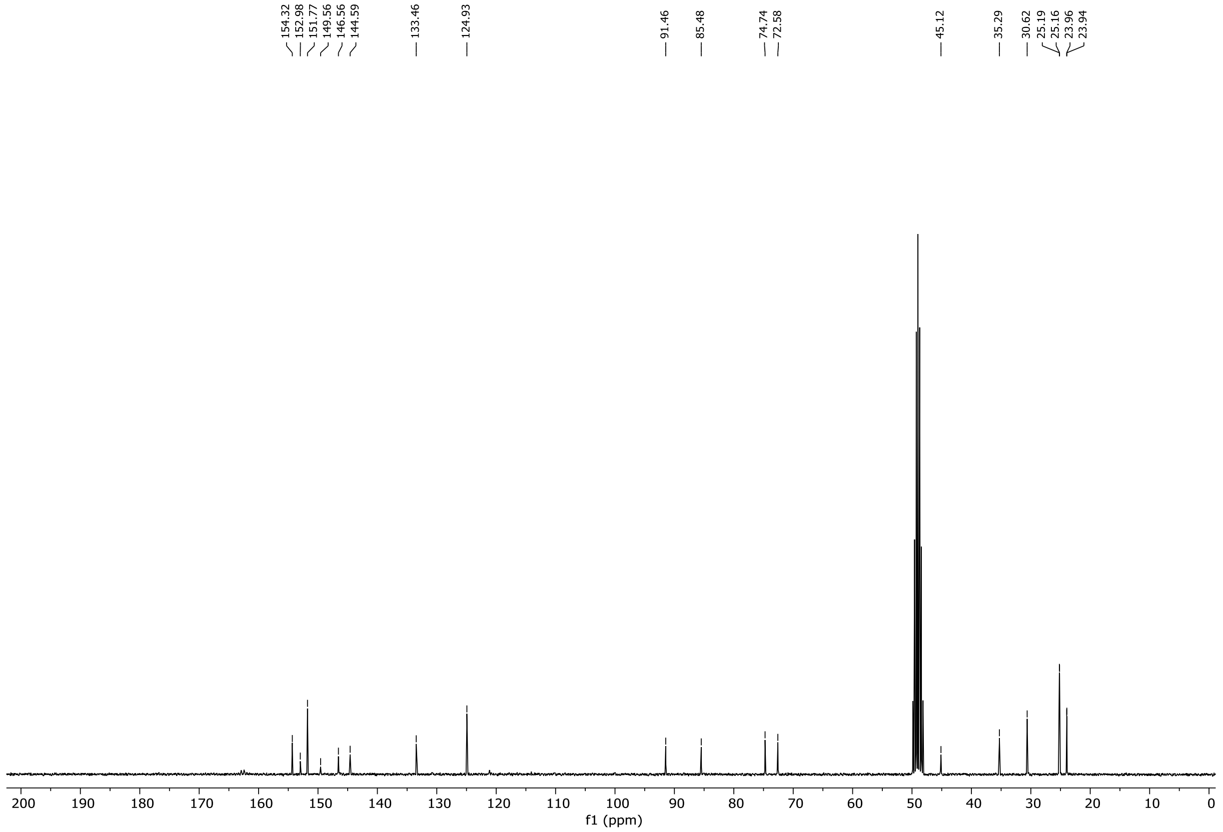

Figure S144.^{13}C NMR of compound ***39***.

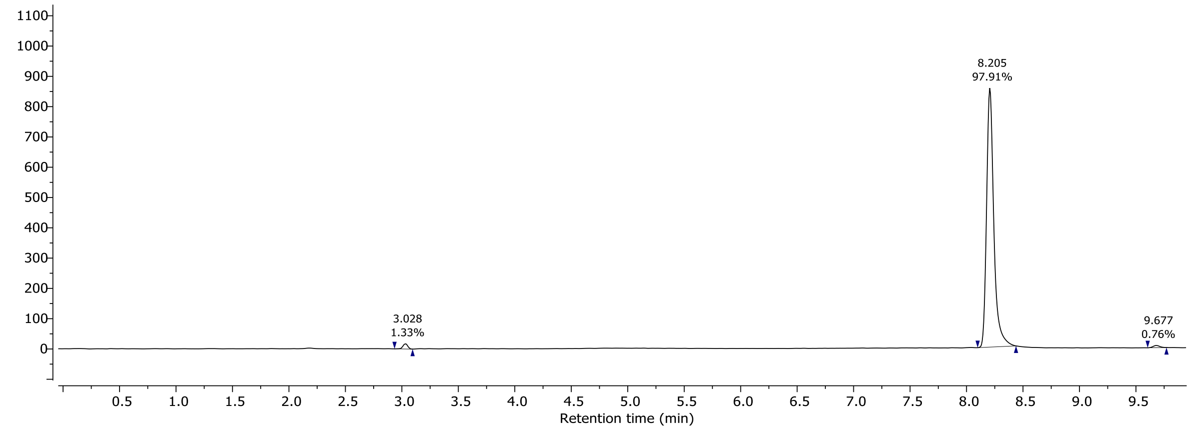

Figure S145. LCMS chromatogram of compound ***39*** at 254 nm.

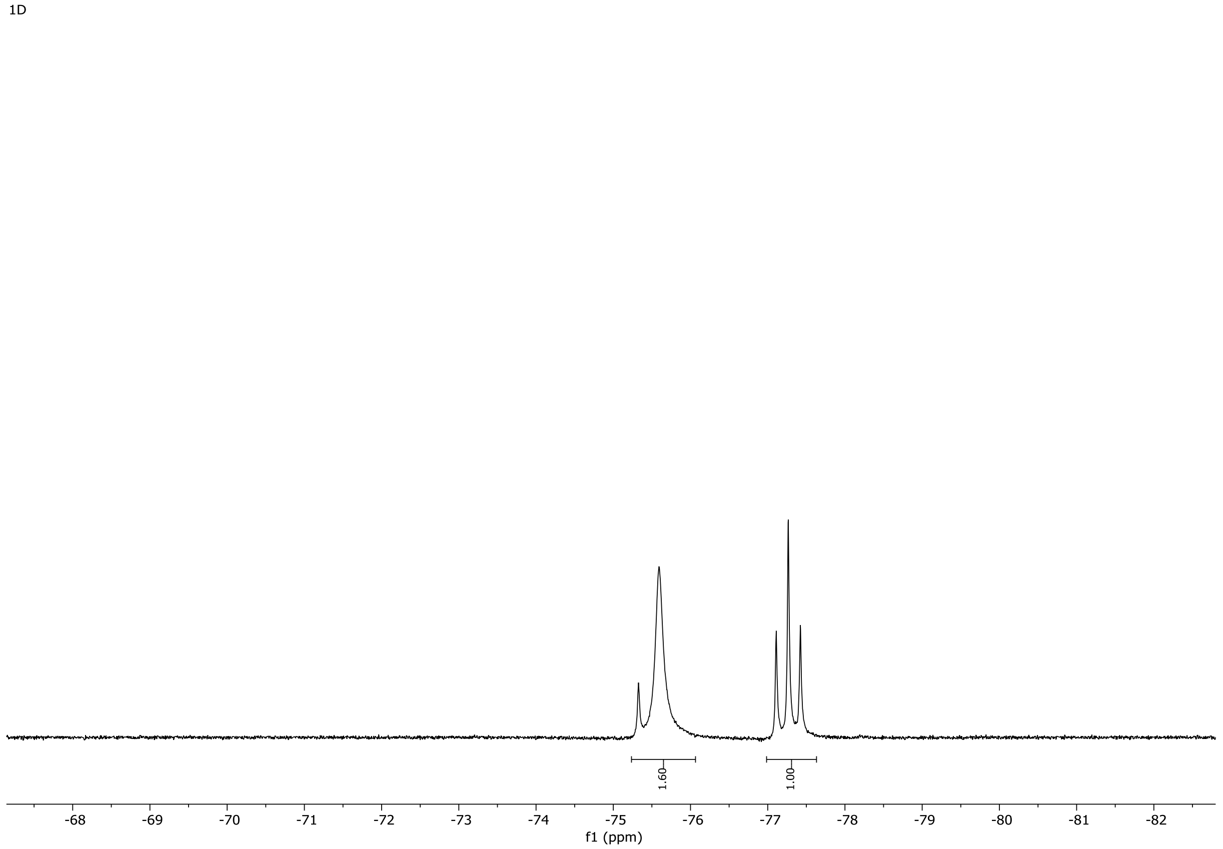

Figure S146.^{19}F NMR of compound ***39***.

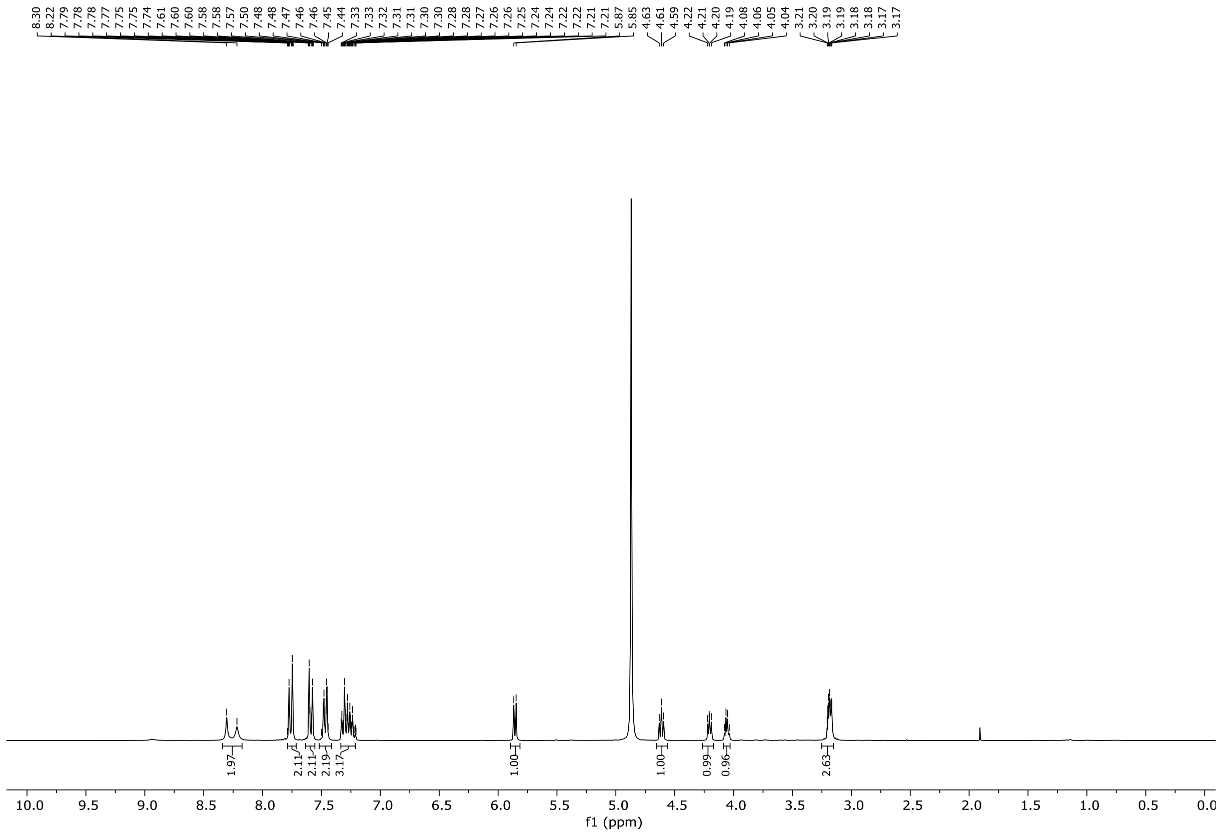

Figure S147. ^{1}H NMR of compound ***40***.

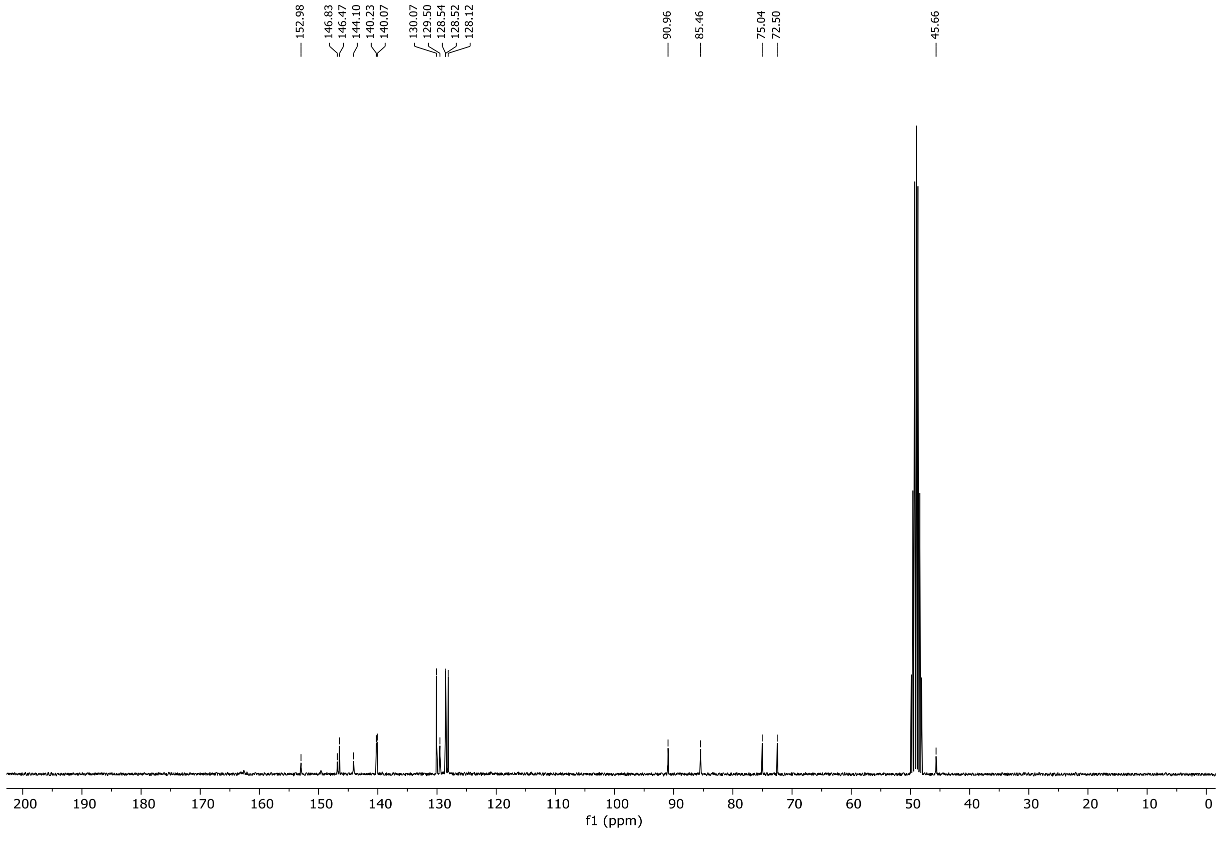

Figure S148. ^{13}**C** NMR of compound ***40***.

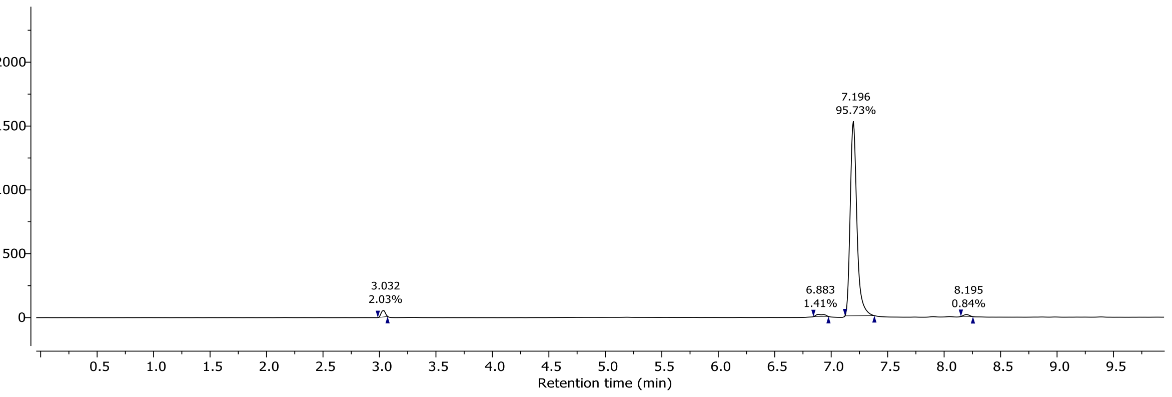

Figure S149. LCMS chromatogram of compound ***40*** at 254 nm.

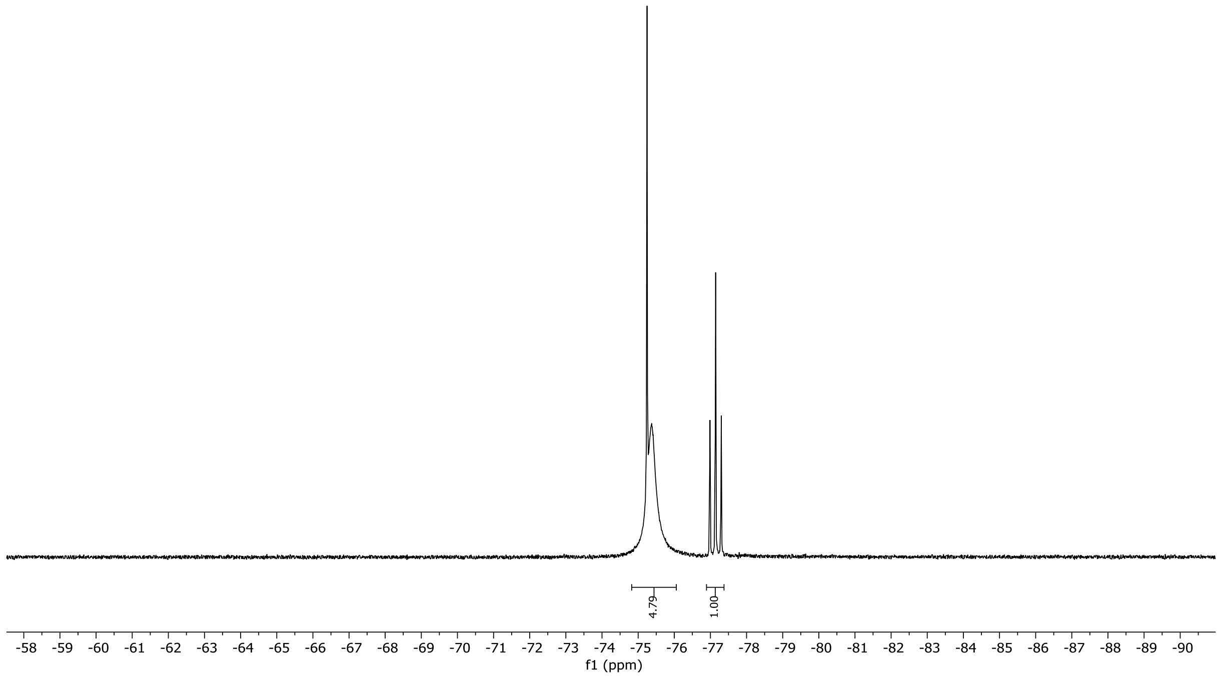

Figure S150. ^{19}F NMR of compound ***40.***

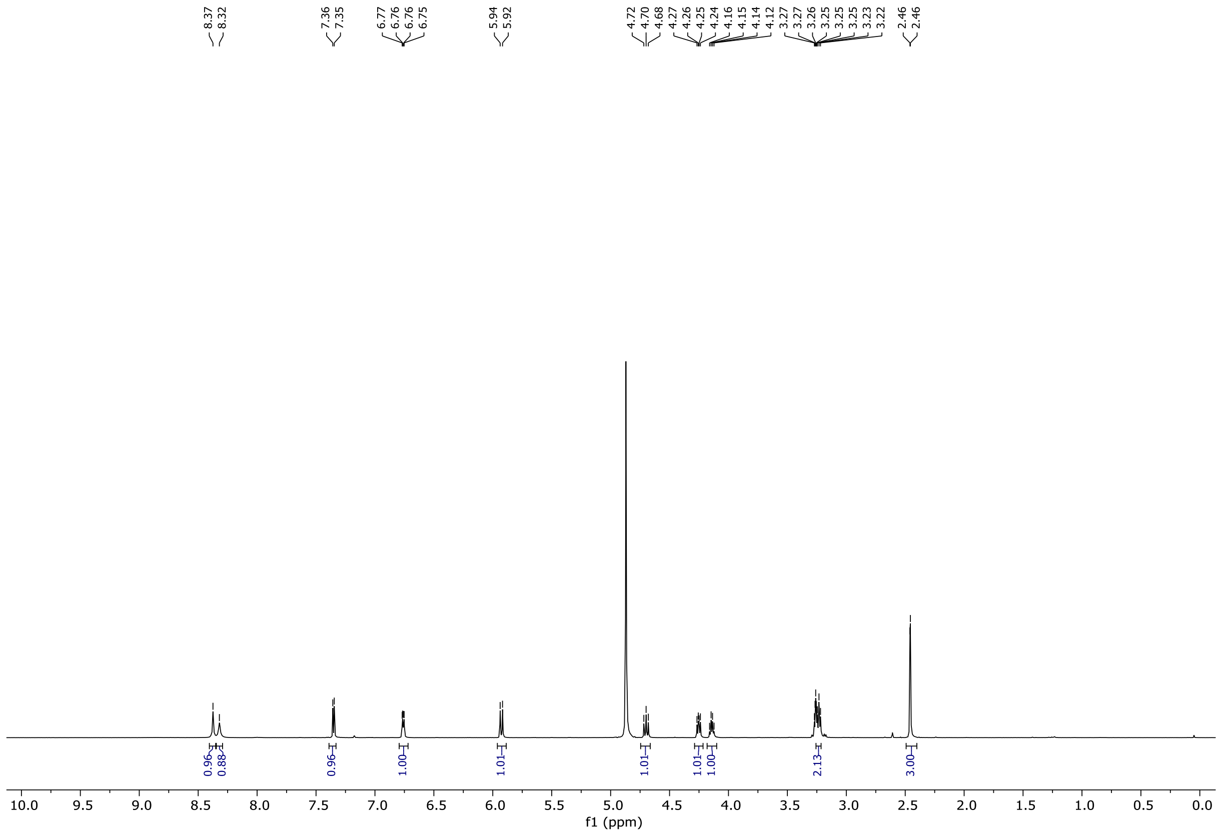

Figure S151. ^{1}H NMR of compound ***41***.

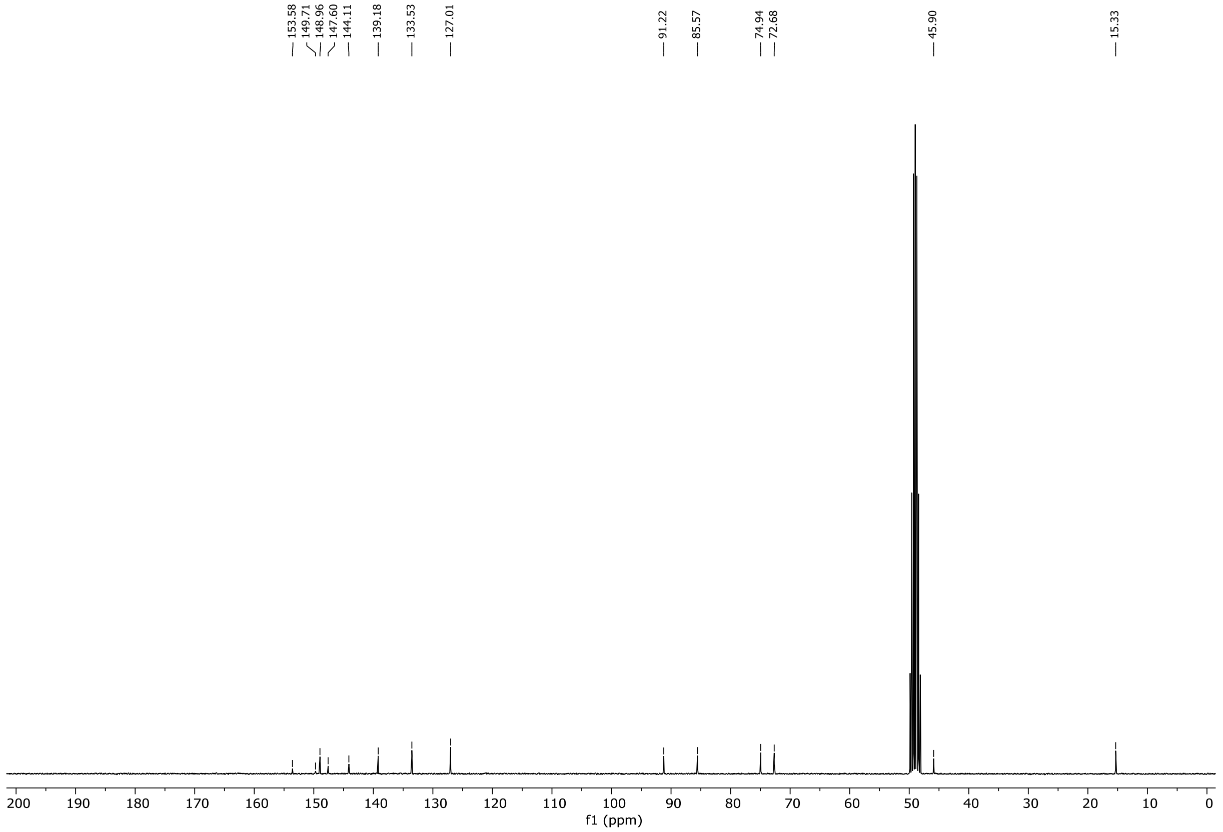

Figure S1152. ^{13}C NMR of compound ***41***.

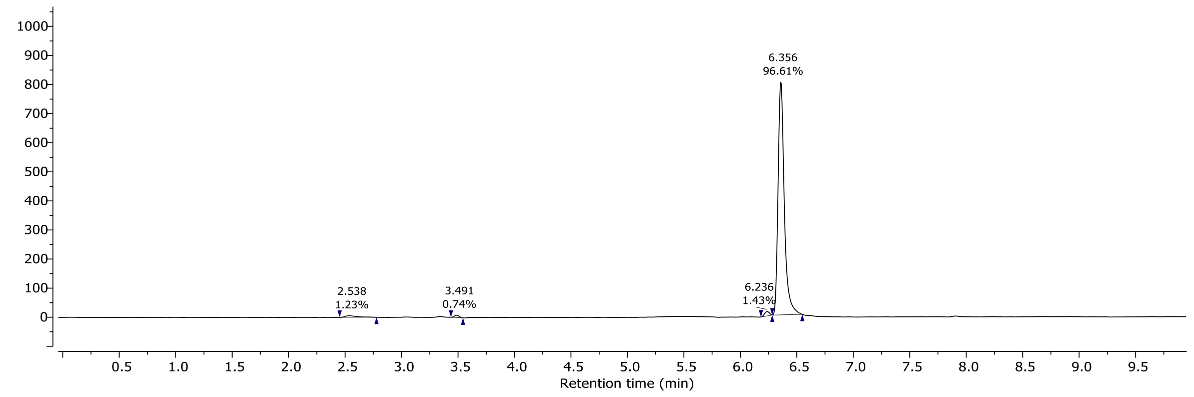

Figure S153. LCMS chromatogram of compound ***41*** at 254 nm.

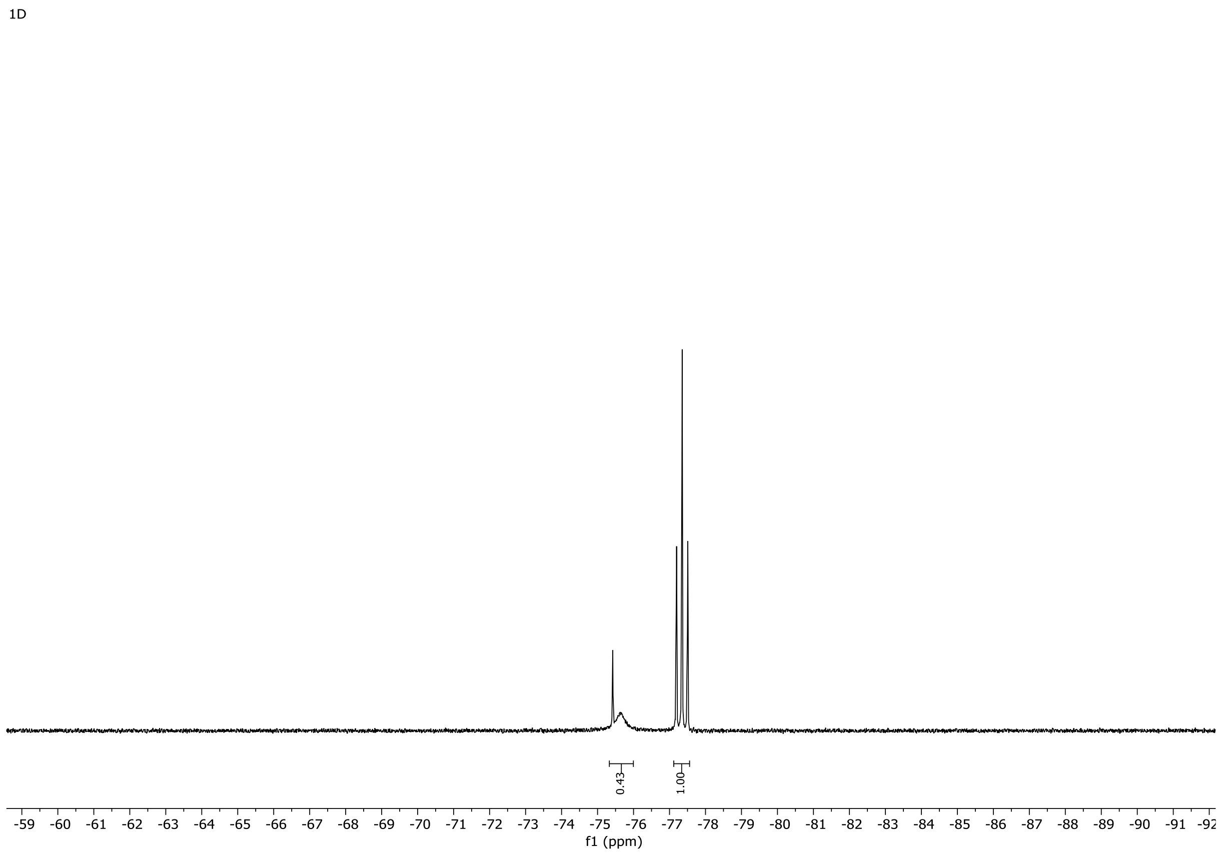

Figure S154. ^{19}F NMR of compound ***41*.**

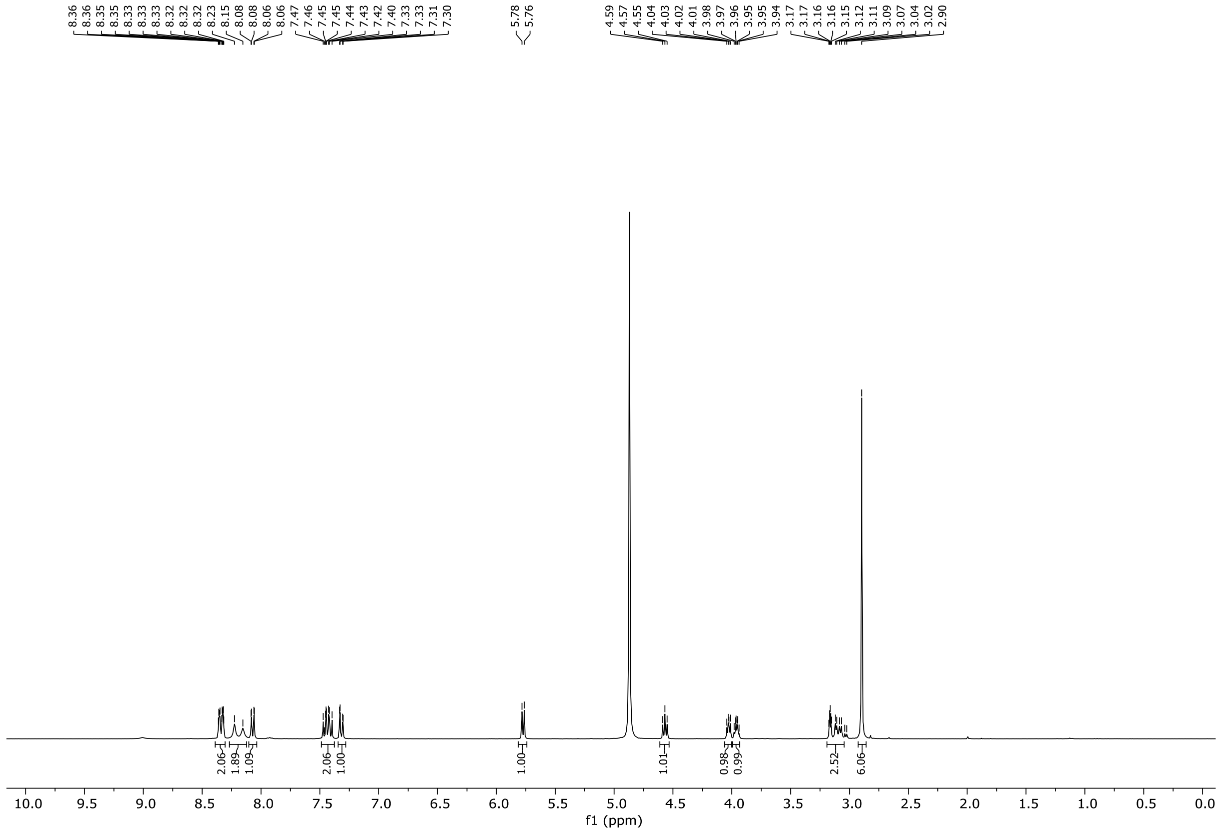

Figure S155. ^{1}H NMR of compound ***42***.

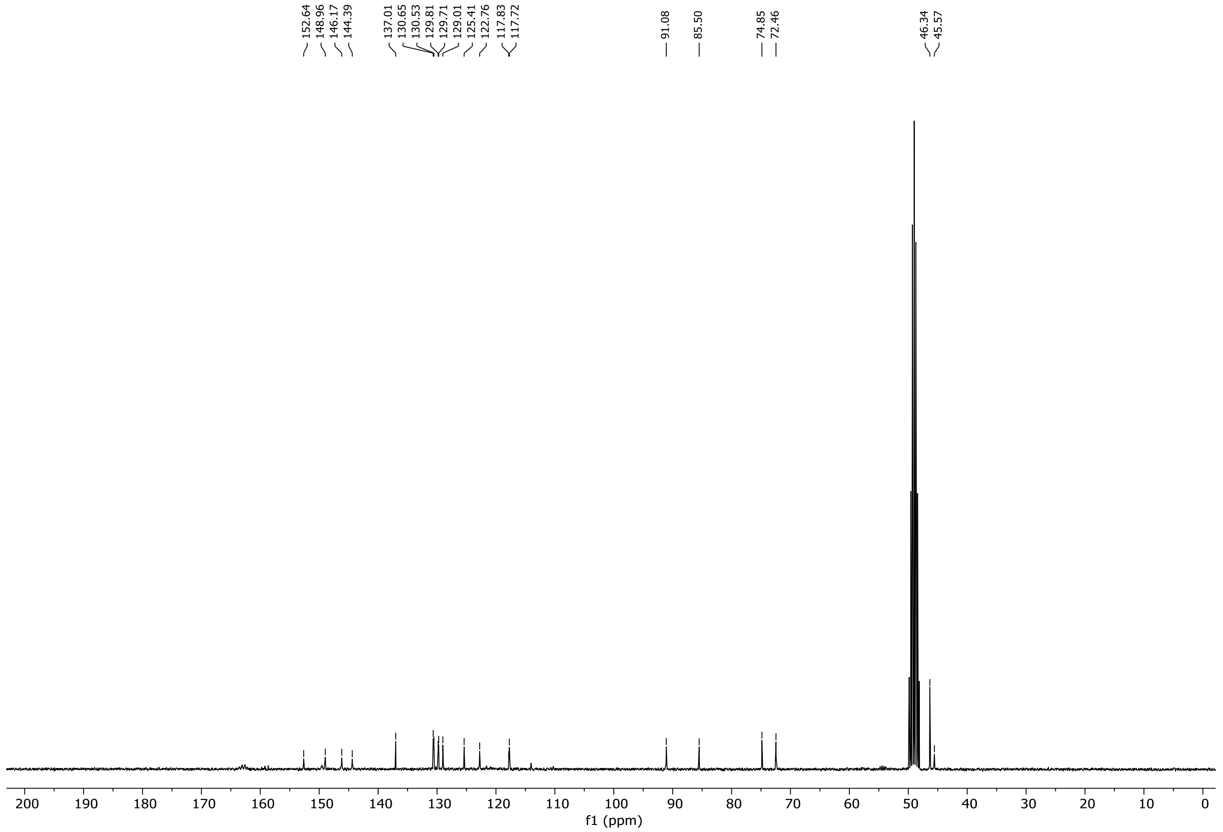

Figure S156. ^{13}C NMR of compound ***42***.

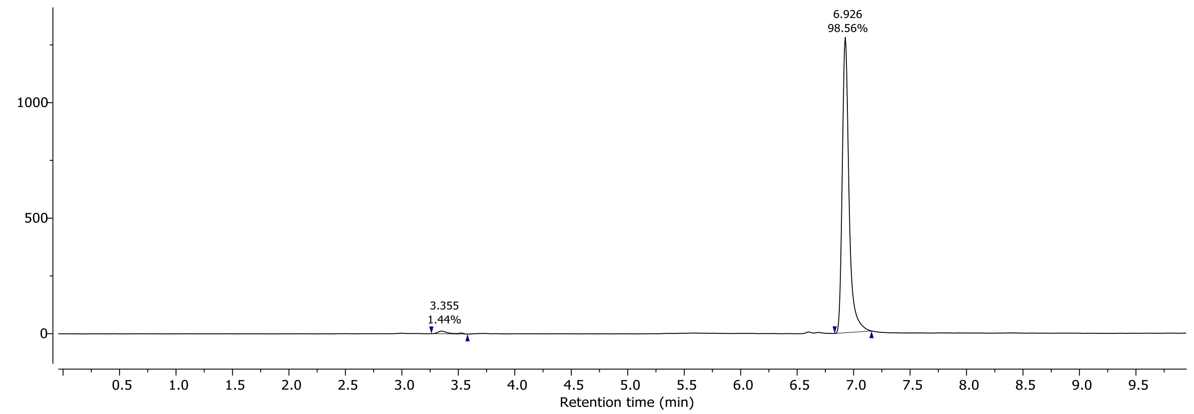

Figure S157. LCMS chromatogram of compound ***42*** at 254 nm.

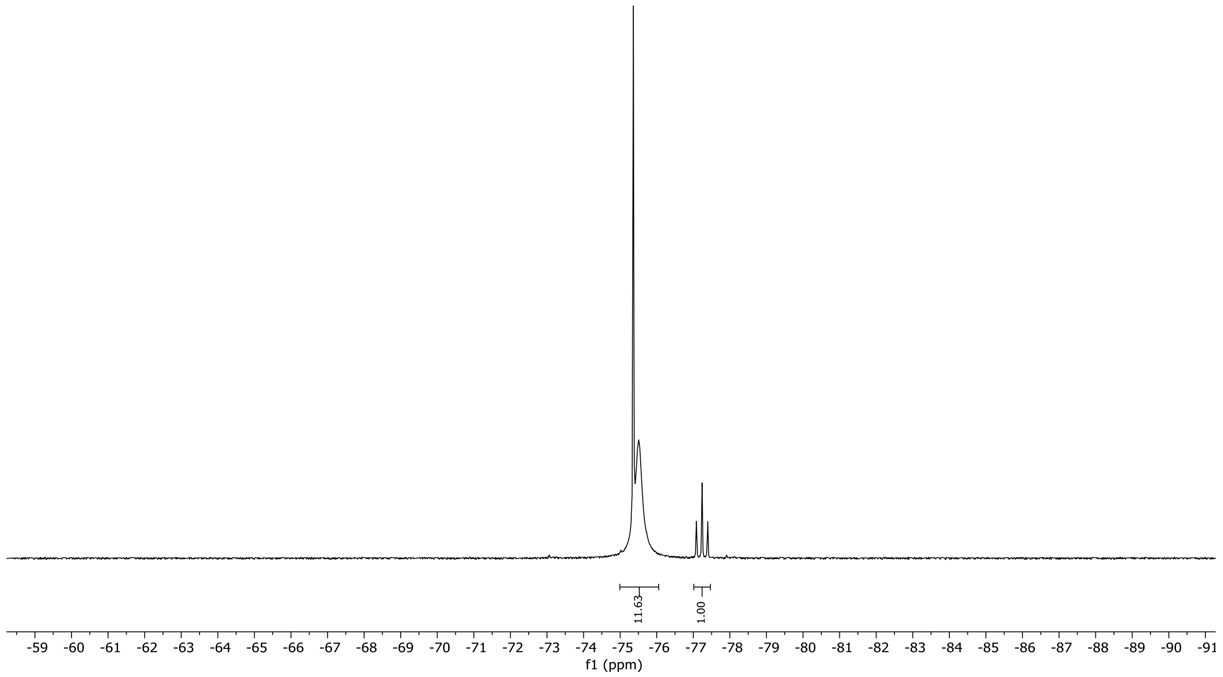

Figure S158. ^{19}F NMR of compound ***42***.

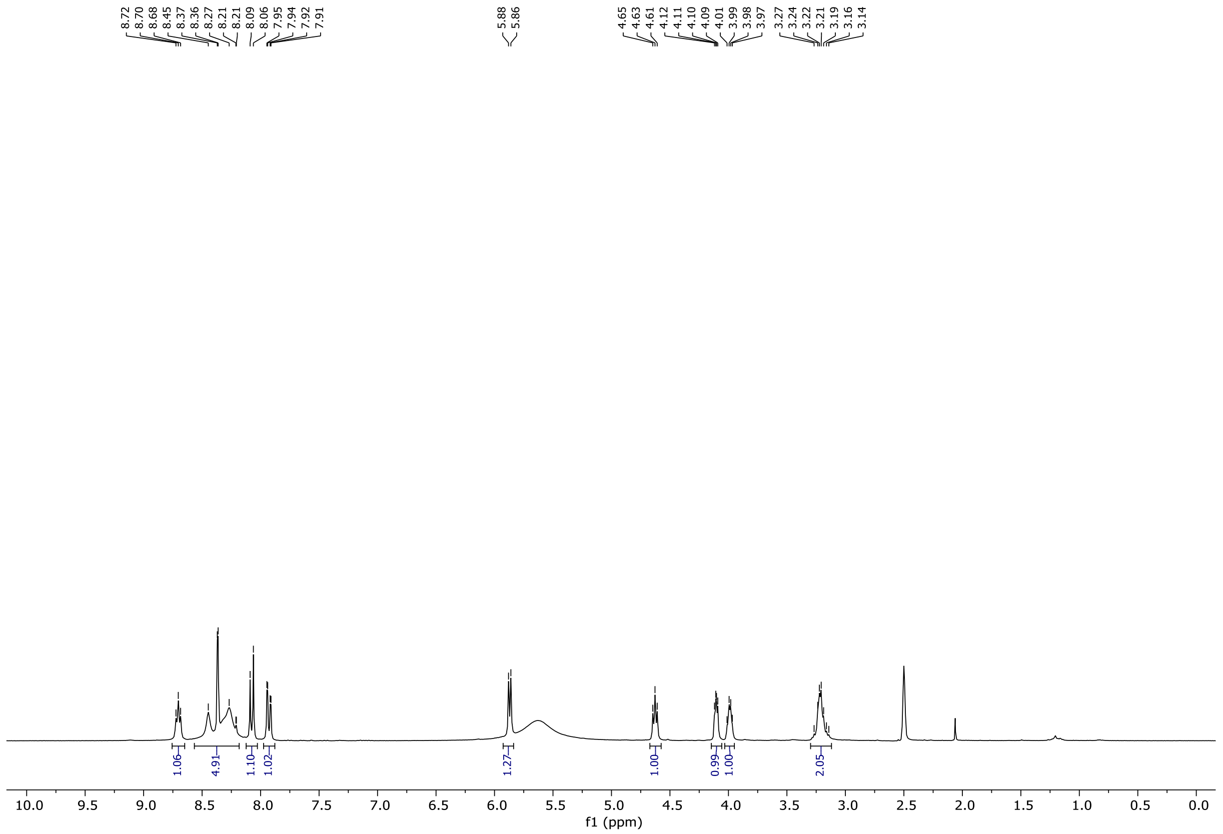

Figure S159. ^{1}H NMR of compound ***44***.

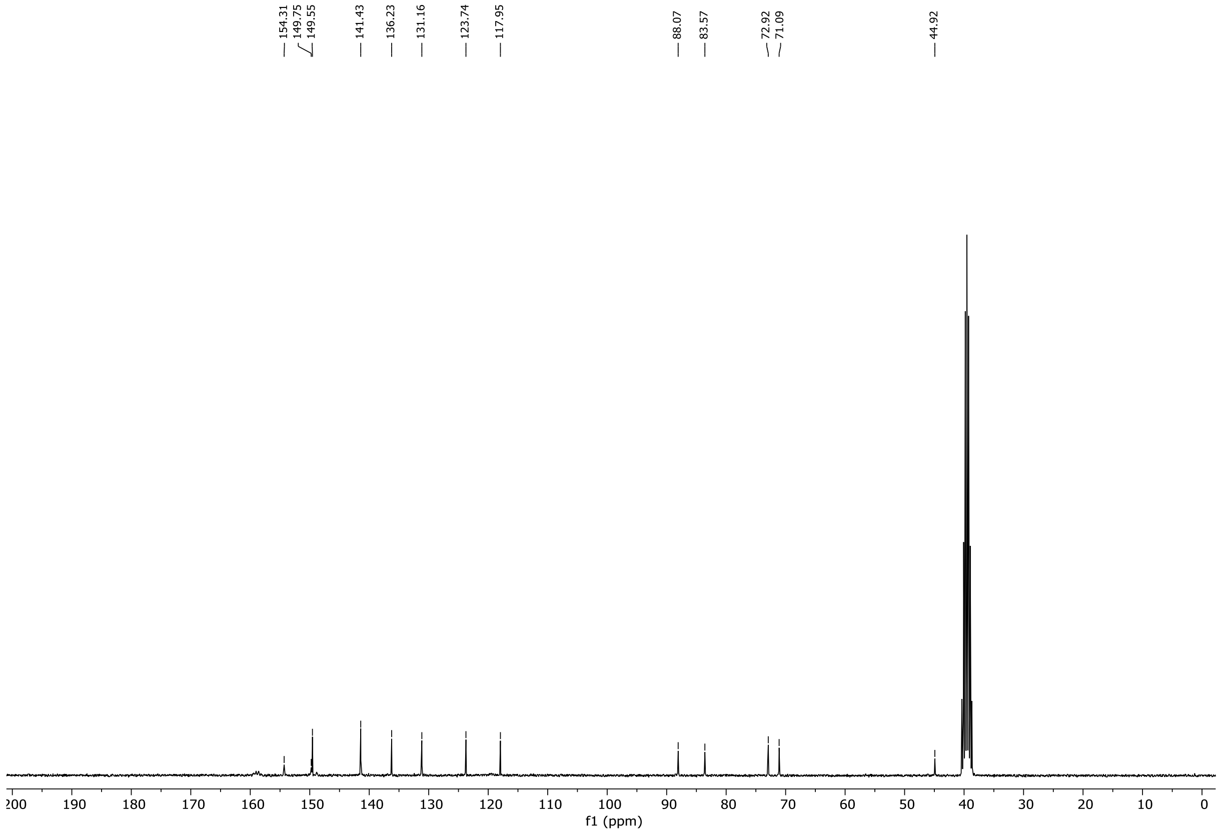

Figure S160. ^{13}C NMR of compound ***44***.

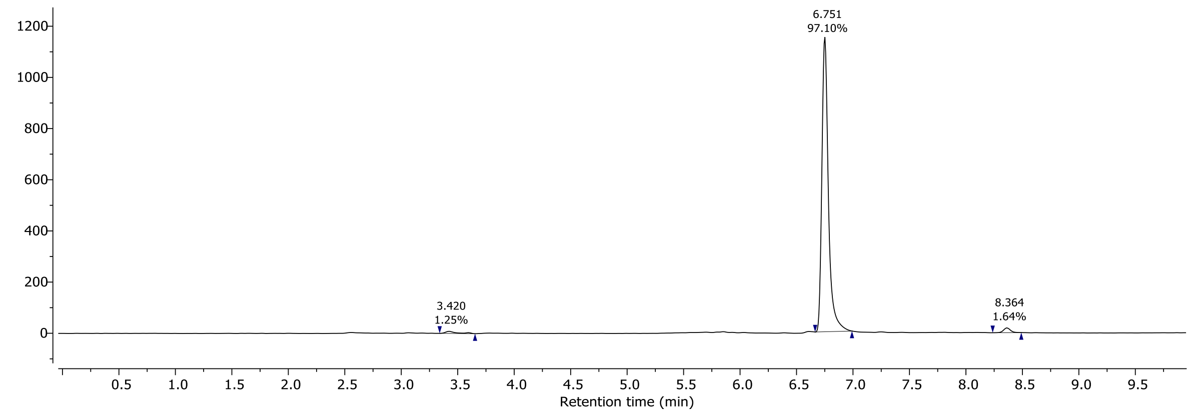

Figure S161. LCMS chromatogram of compound ***44*** at 254 nm.

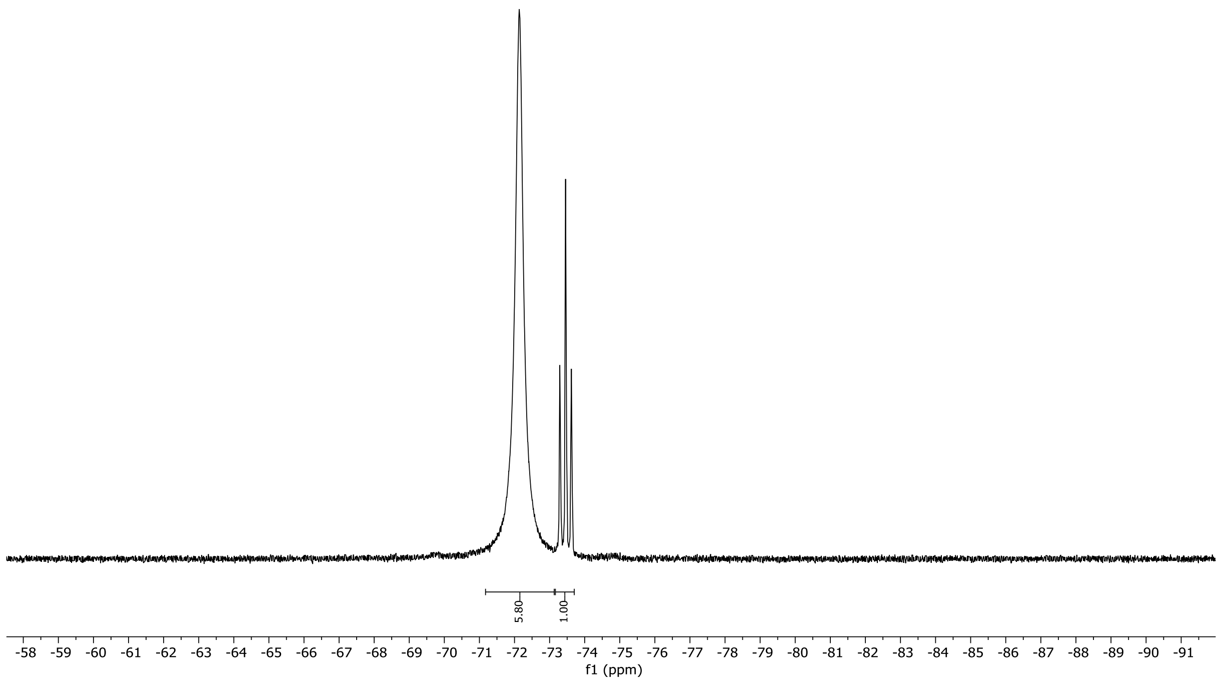

Figure S162. ^{19}F NMR of compound ***44.***

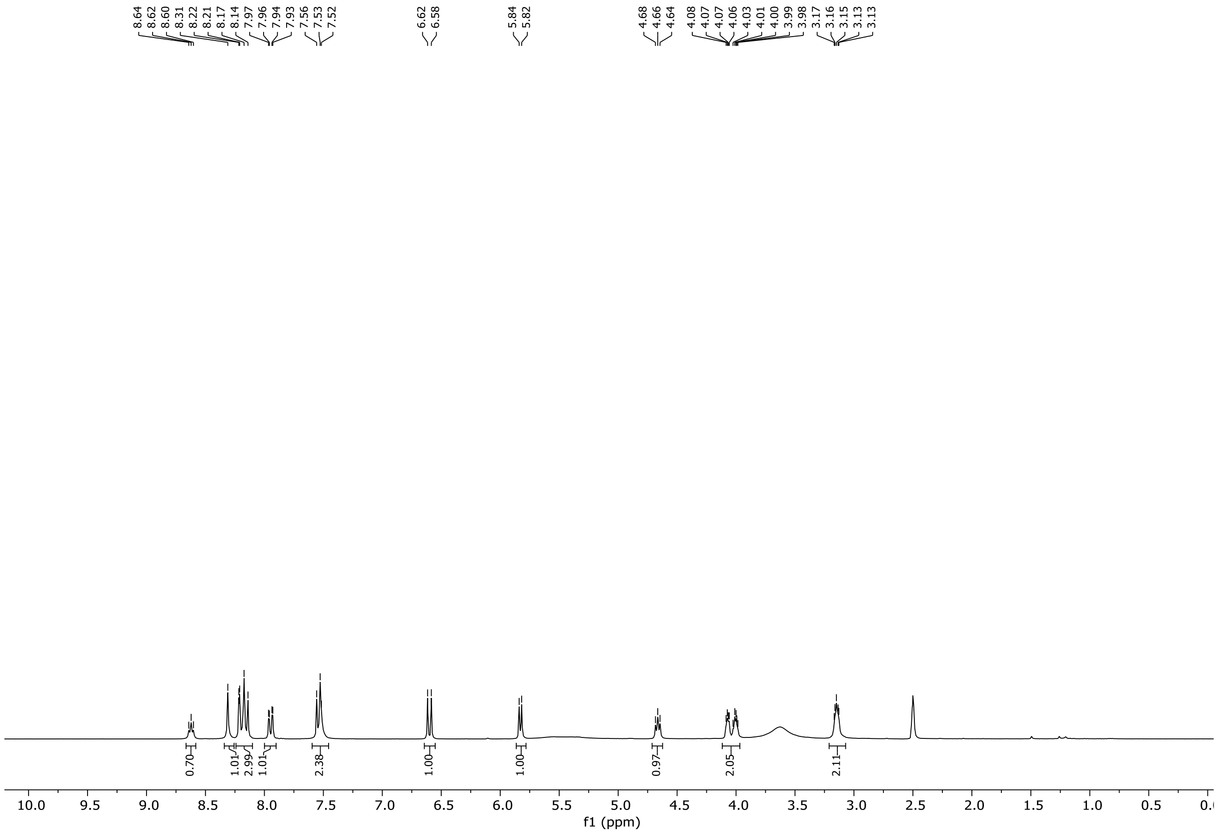

Figure S163. ^{1}H NMR of compound ***45***.

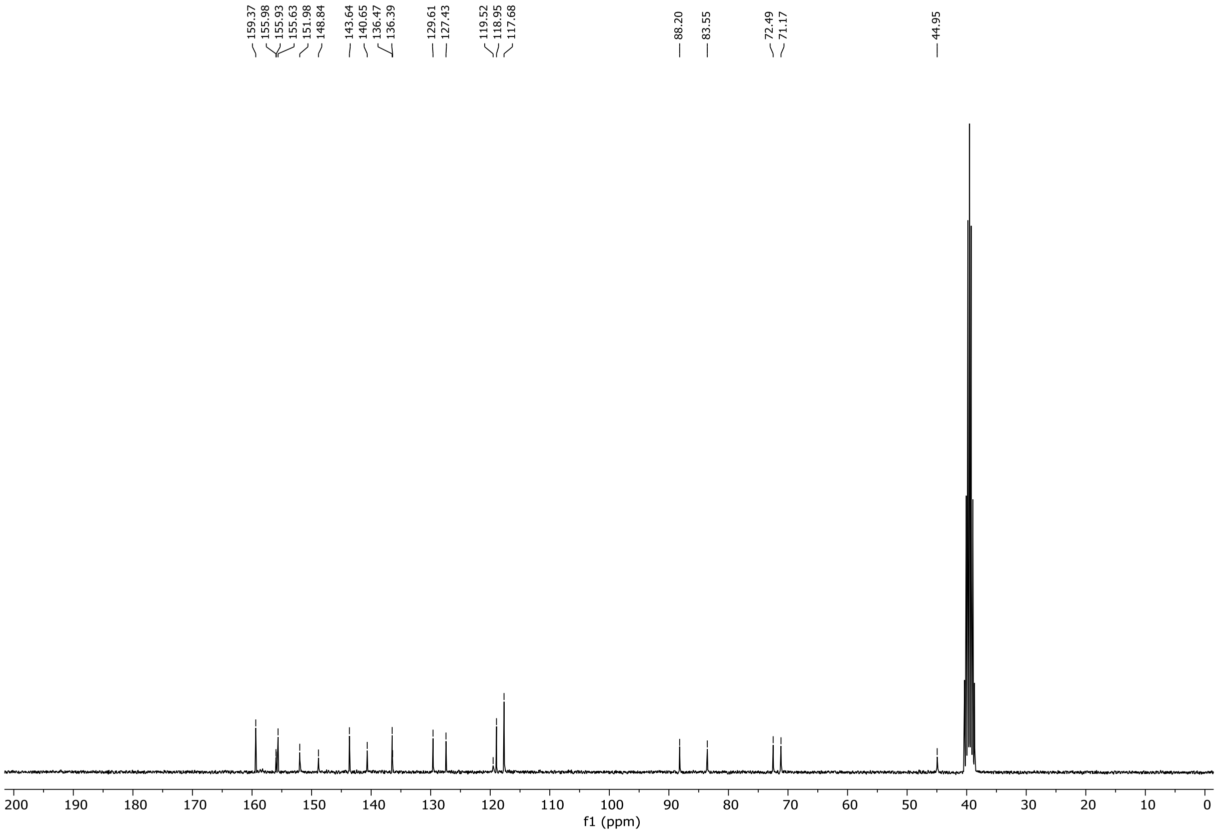

Figure S164. ^{13}C NMR of compound **45**.

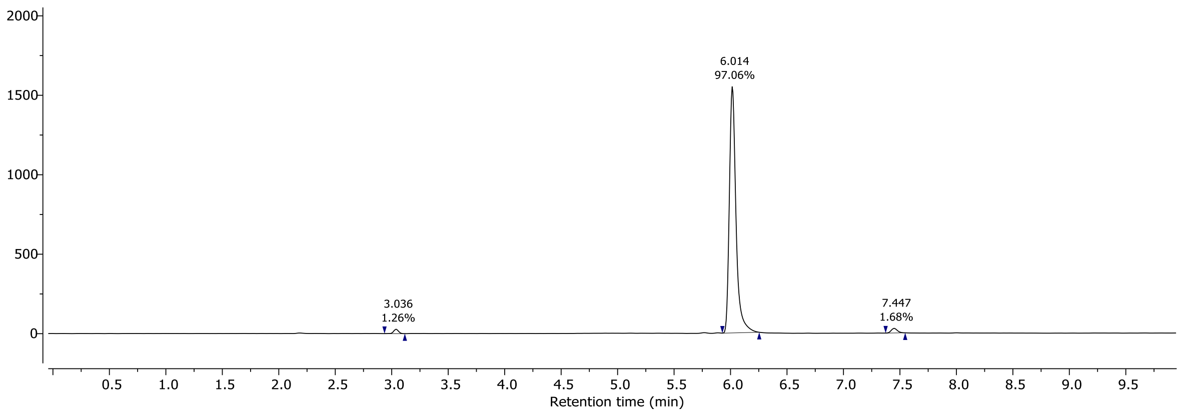

Figure S165.LCMS chromatogram of compound ***45*** at 254 nm.

1D

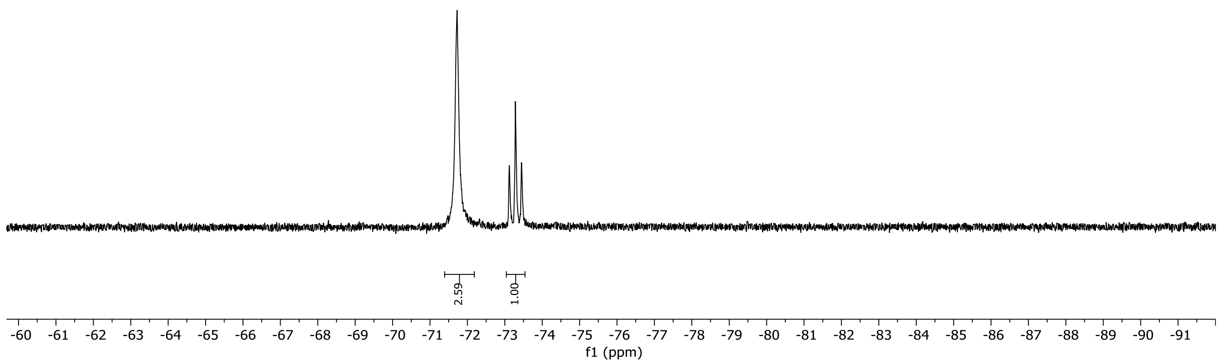

Figure S166. ^{19}F NMR of compound ***45*.**

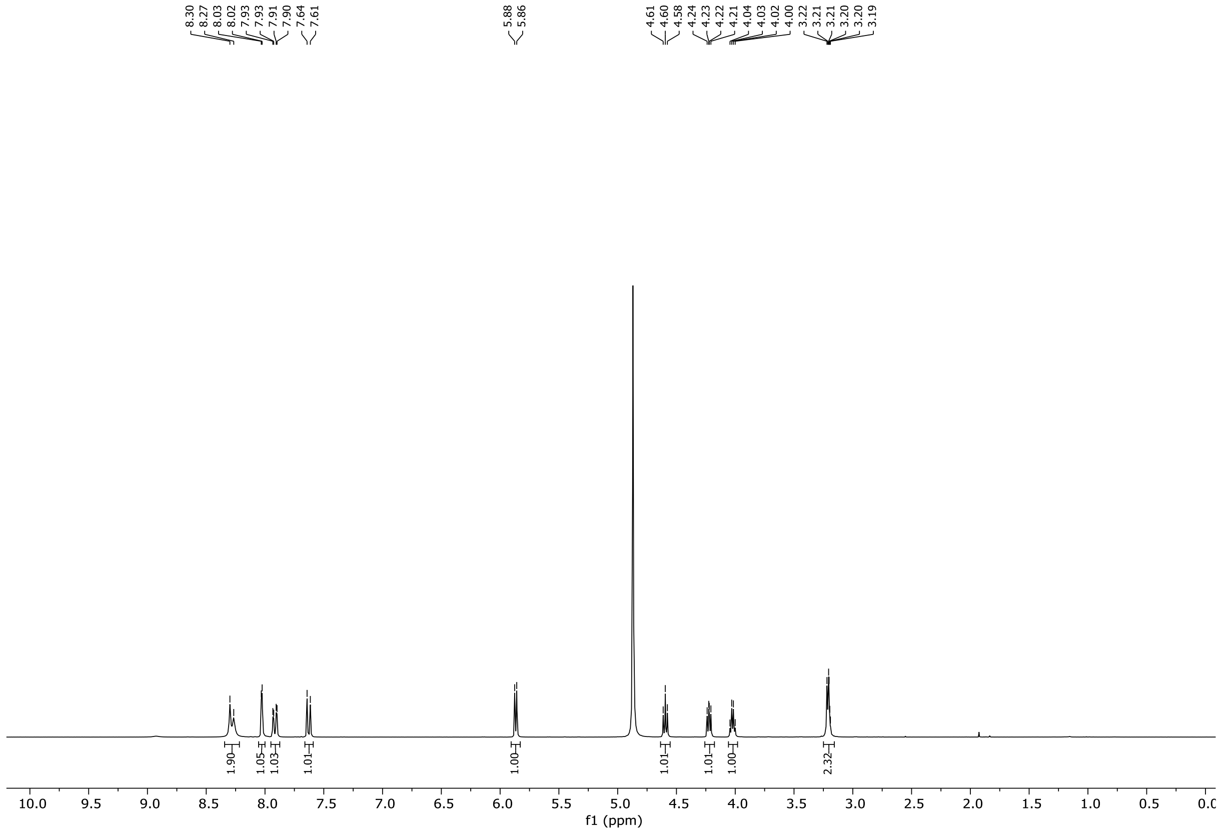

Figure S167. ^{1}H NMR of compound ***46***.

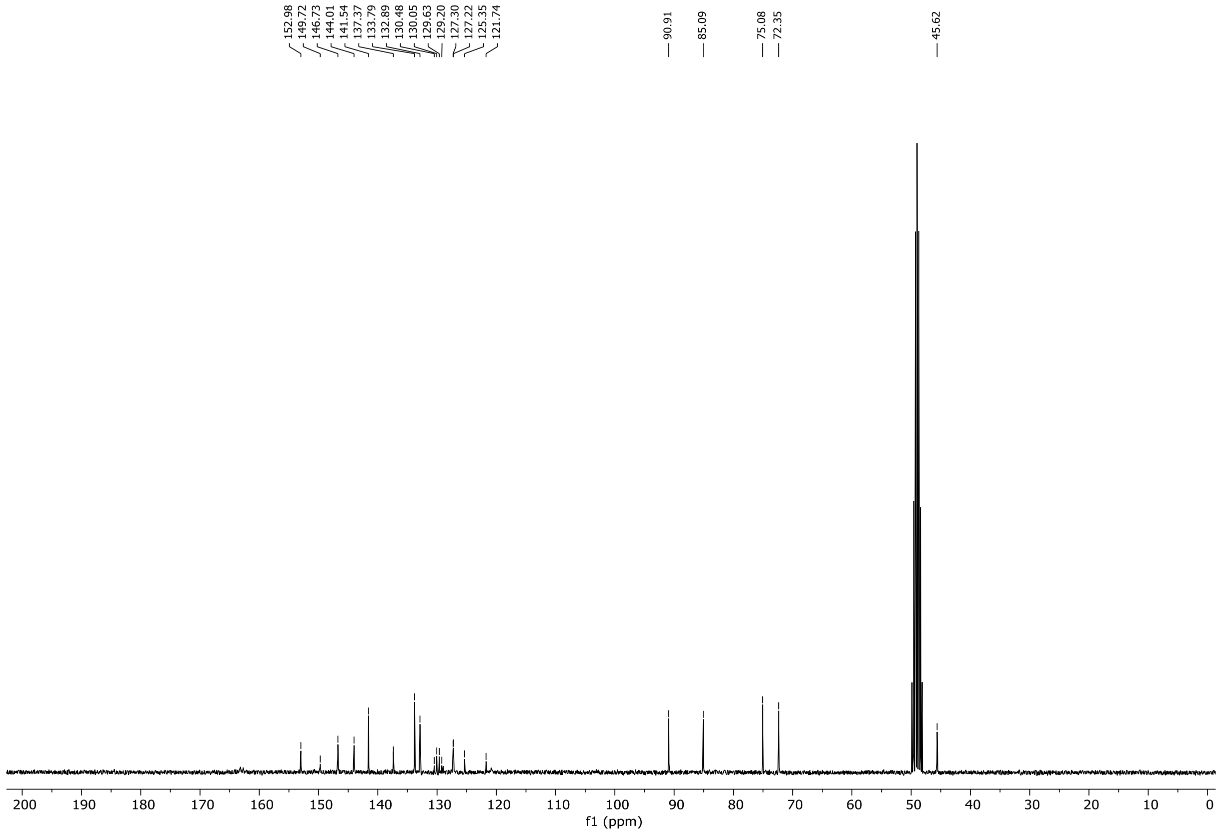

Figure S168. ^{13}C NMR of compound ***46.***

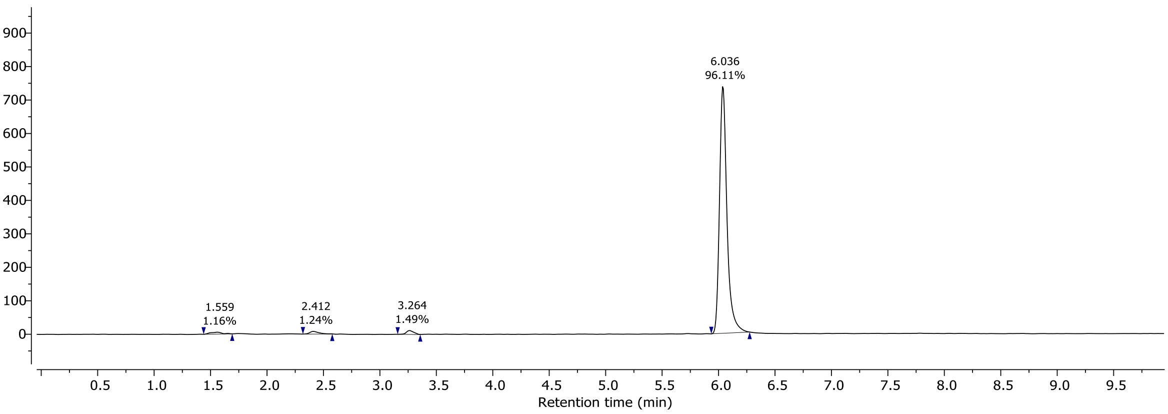

Figure S169. LCMS chromatogram of compound ***46*** at 254 nm.

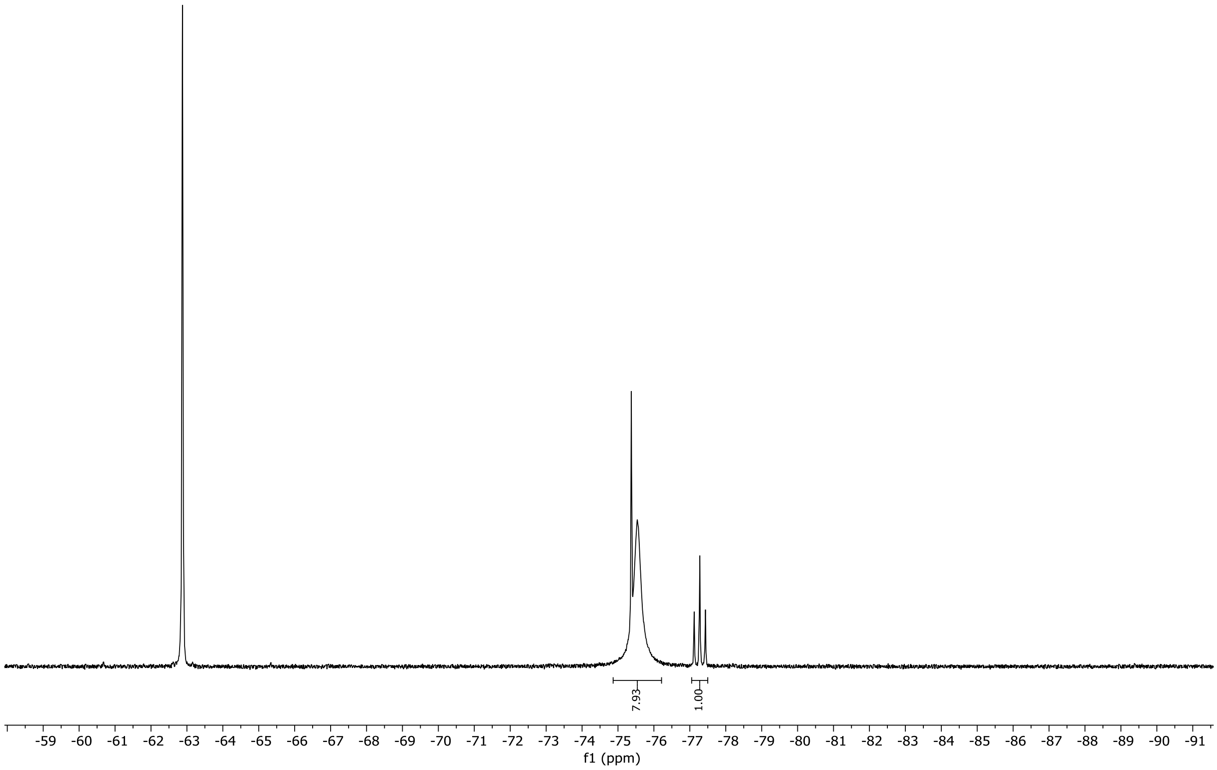

Figure S170. ^{19}F NMR of compound ***46*.**

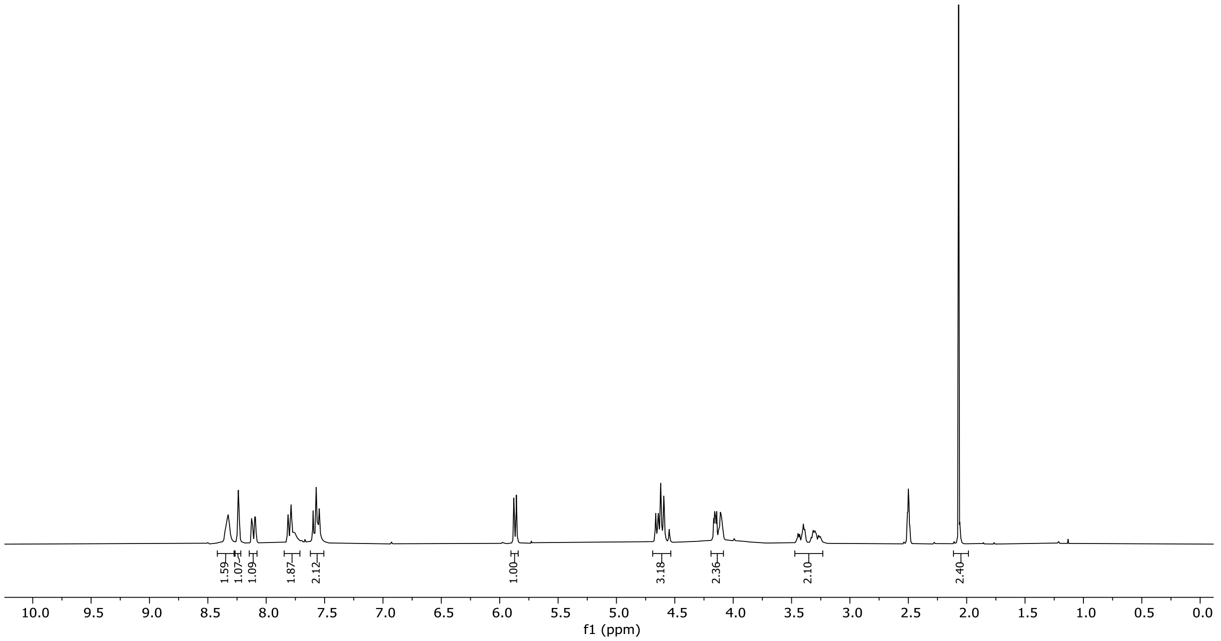

Figure S171. ^{1}H NMR of compound ***47***.

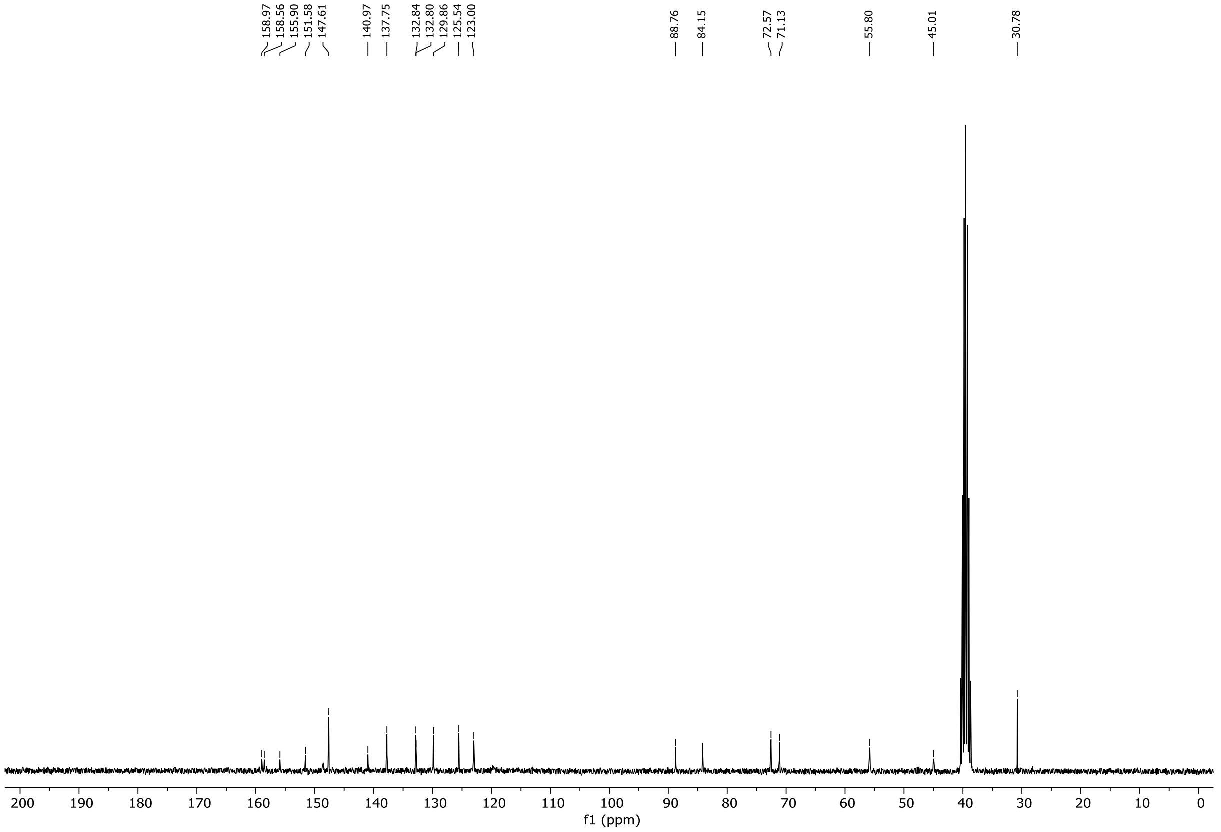

Figure S172. ^{13}C NMR of compound ***47***.

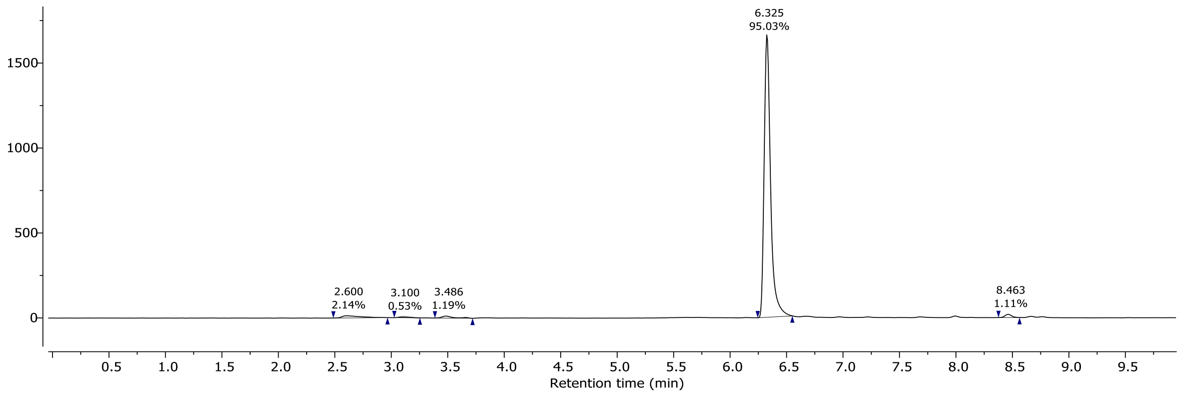

Figure S173. LCMS chromatogram of compound ***47*** at 254 nm.

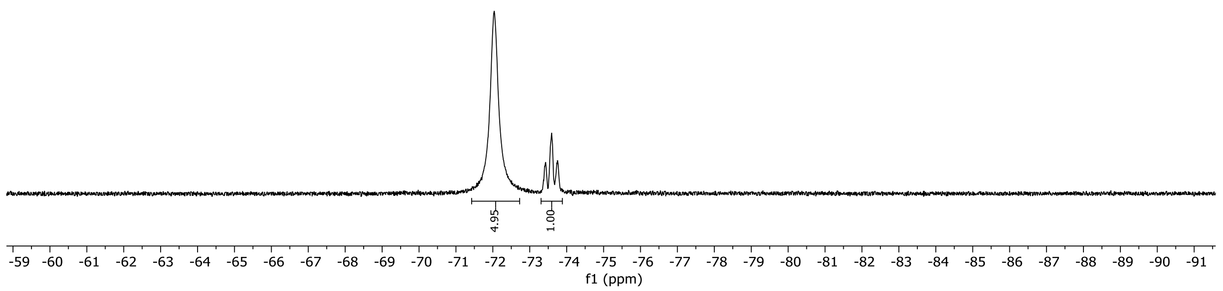

Figure S174. ^{19}F NMR of compound ***47***.

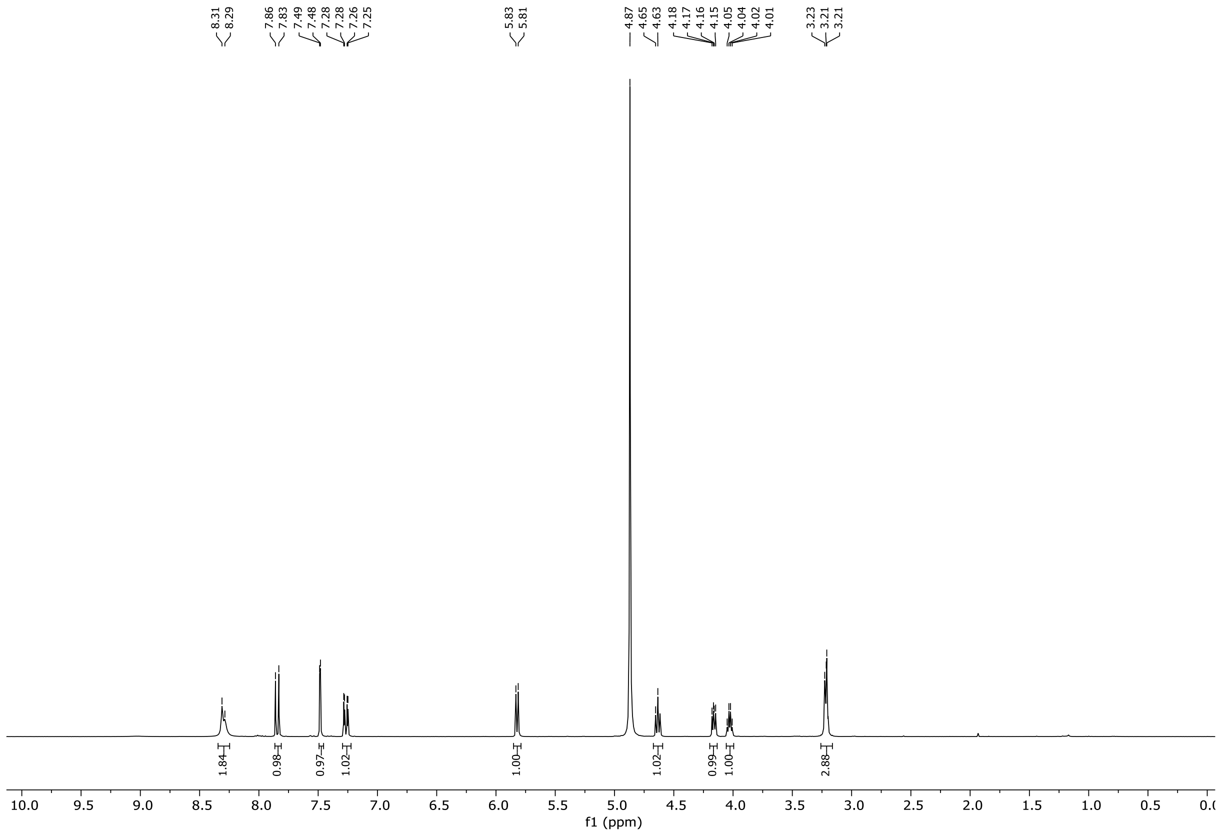

Figure S175. ^{1}H NMR of compound ***48***.

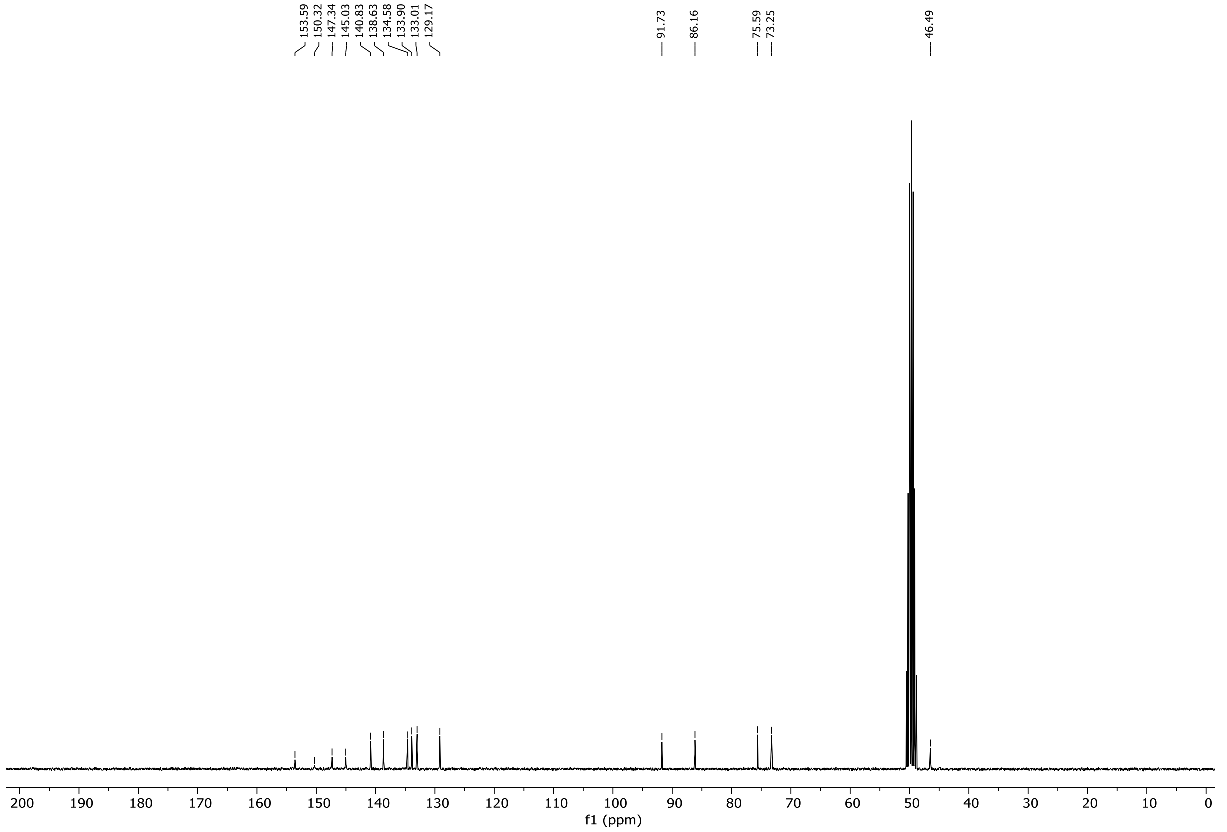

Figure S176. ^{13}C NMR of compound ***48***.

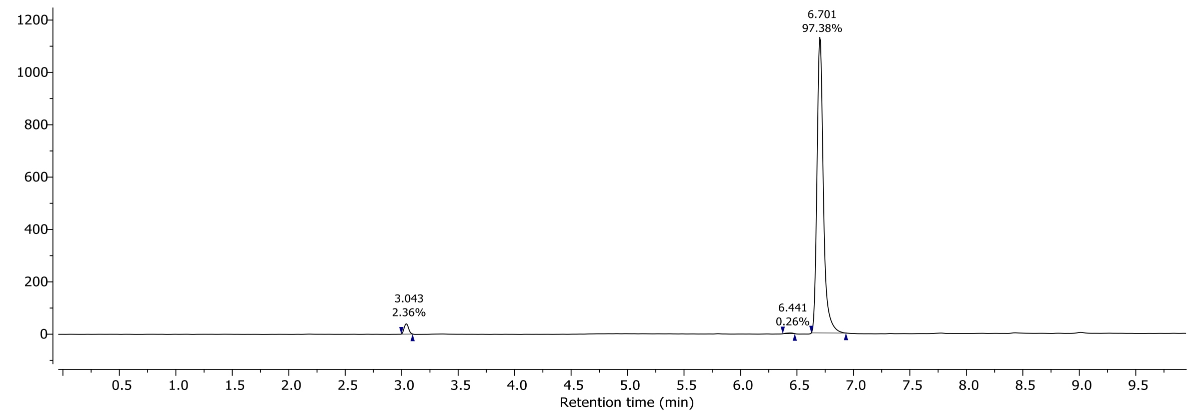

Figure S177.: LCMS chromatogram of compound ***48*** at 254 nm.

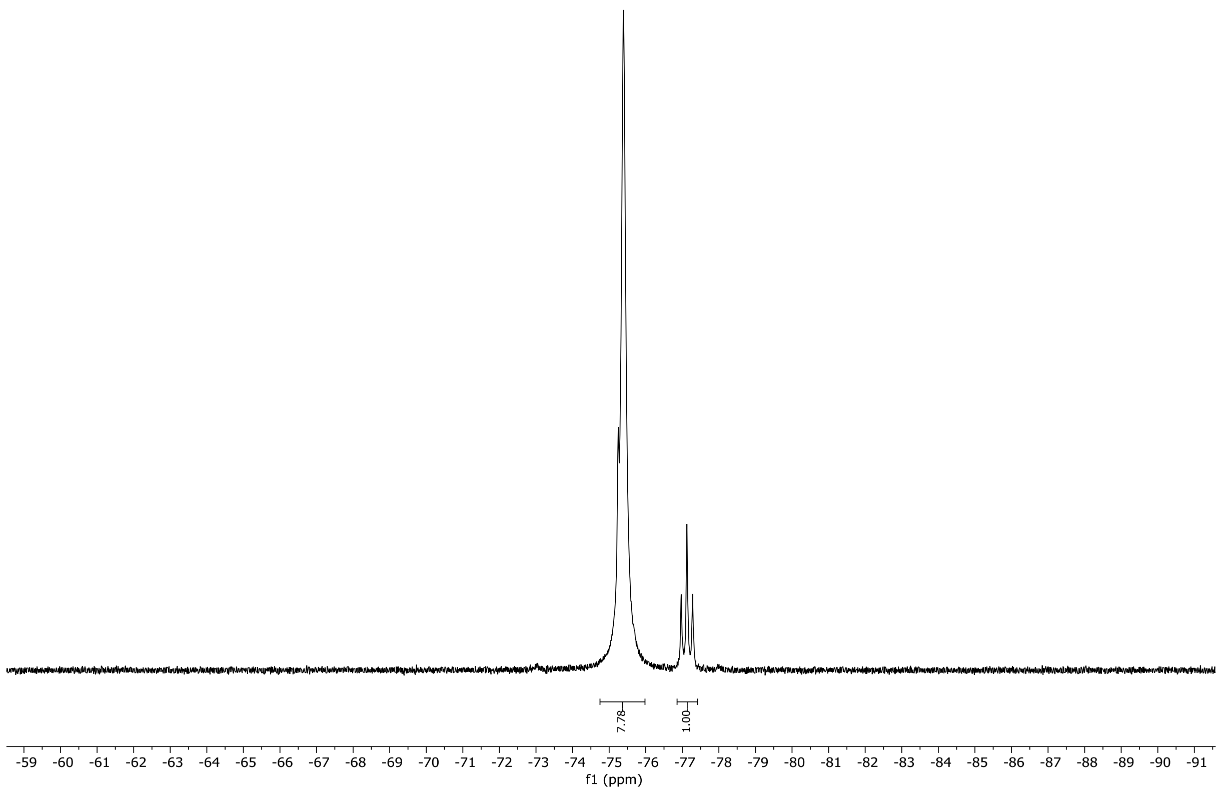

Figure S178. ^{19}F NMR of compound ***48***.

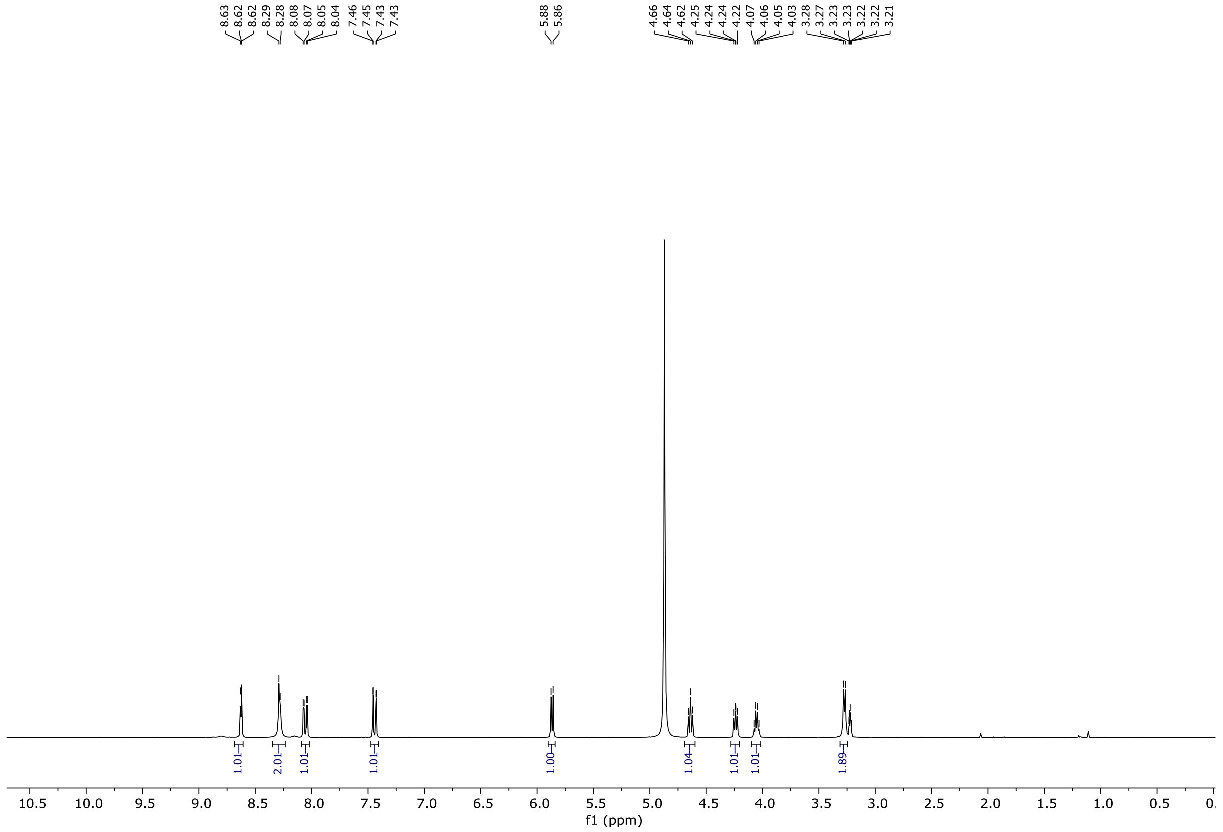

Figure S179. ^{1}H NMR of compound ***49***.

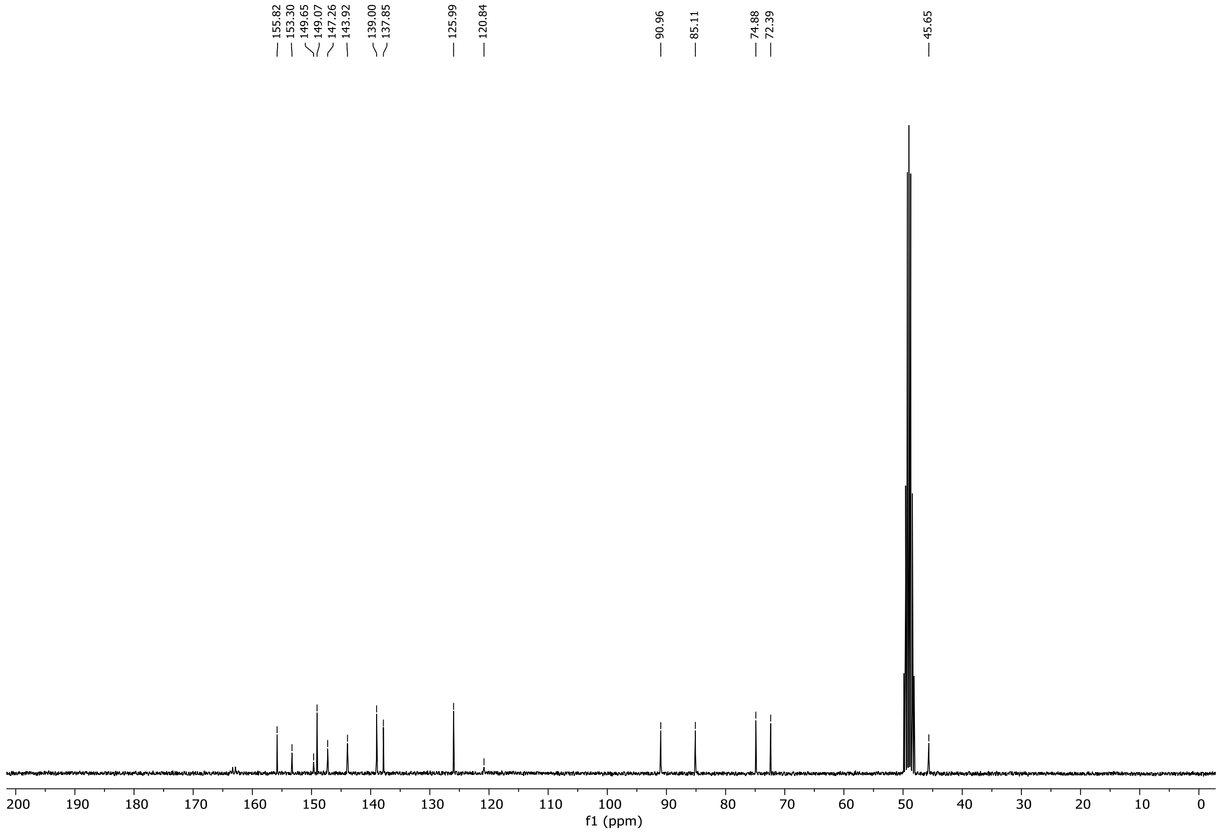

Figure S180. ^{13}C NMR of compound ***49***.

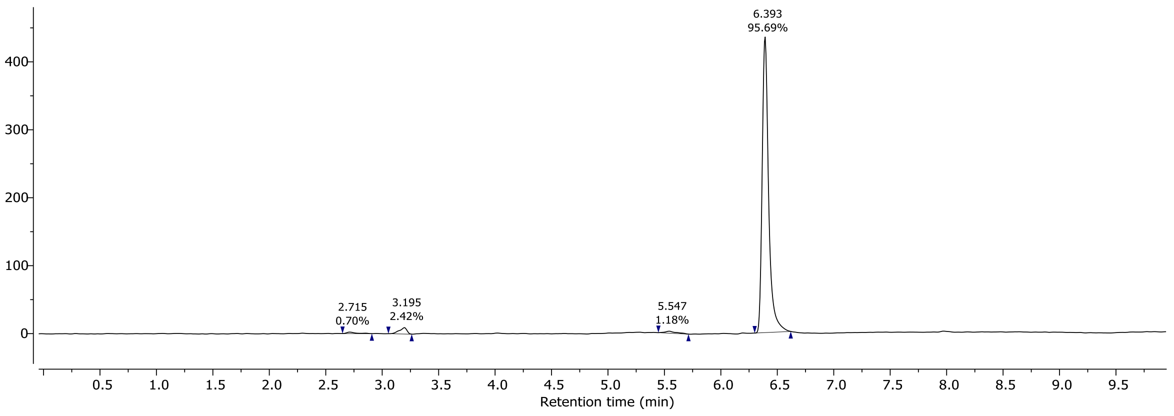

Figure S181. LCMS chromatogram of compound ***49*** at 254 nm.

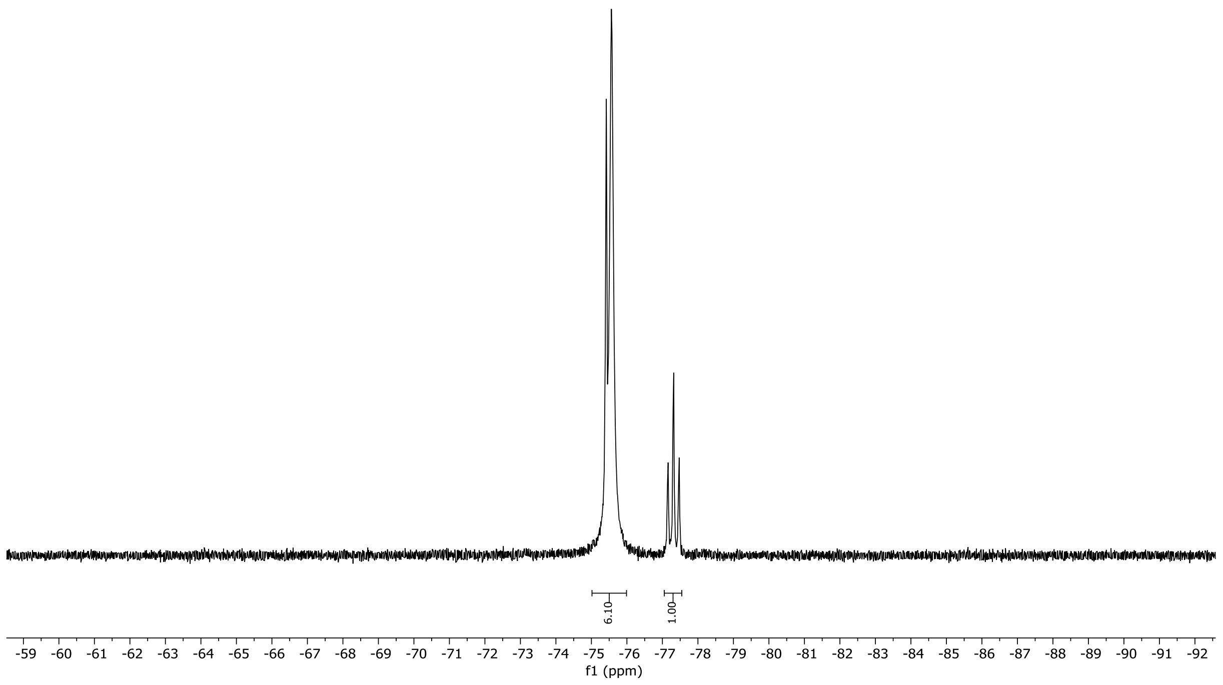

Figure S182. ^{19}F NMR of compound ***49*.**

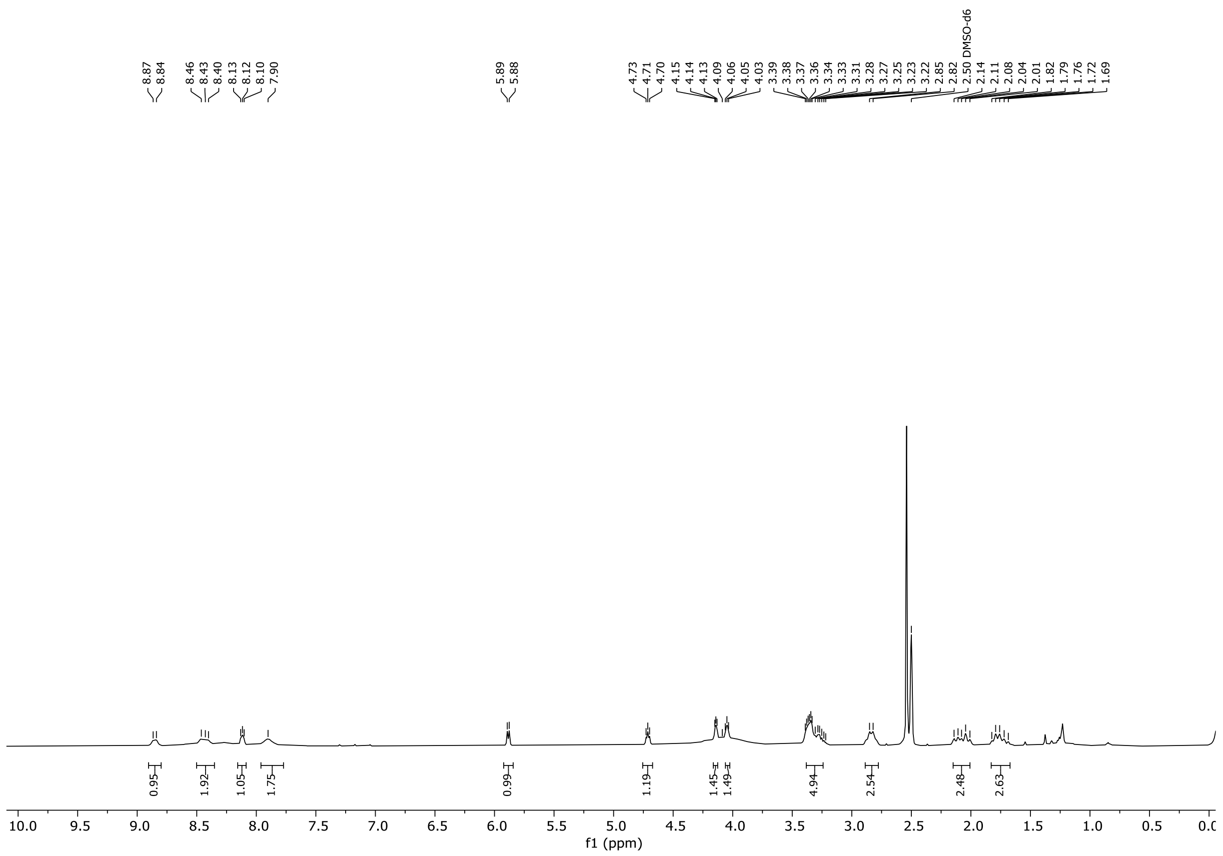

Figure S183. ^{1}H NMR of compound ***50***.

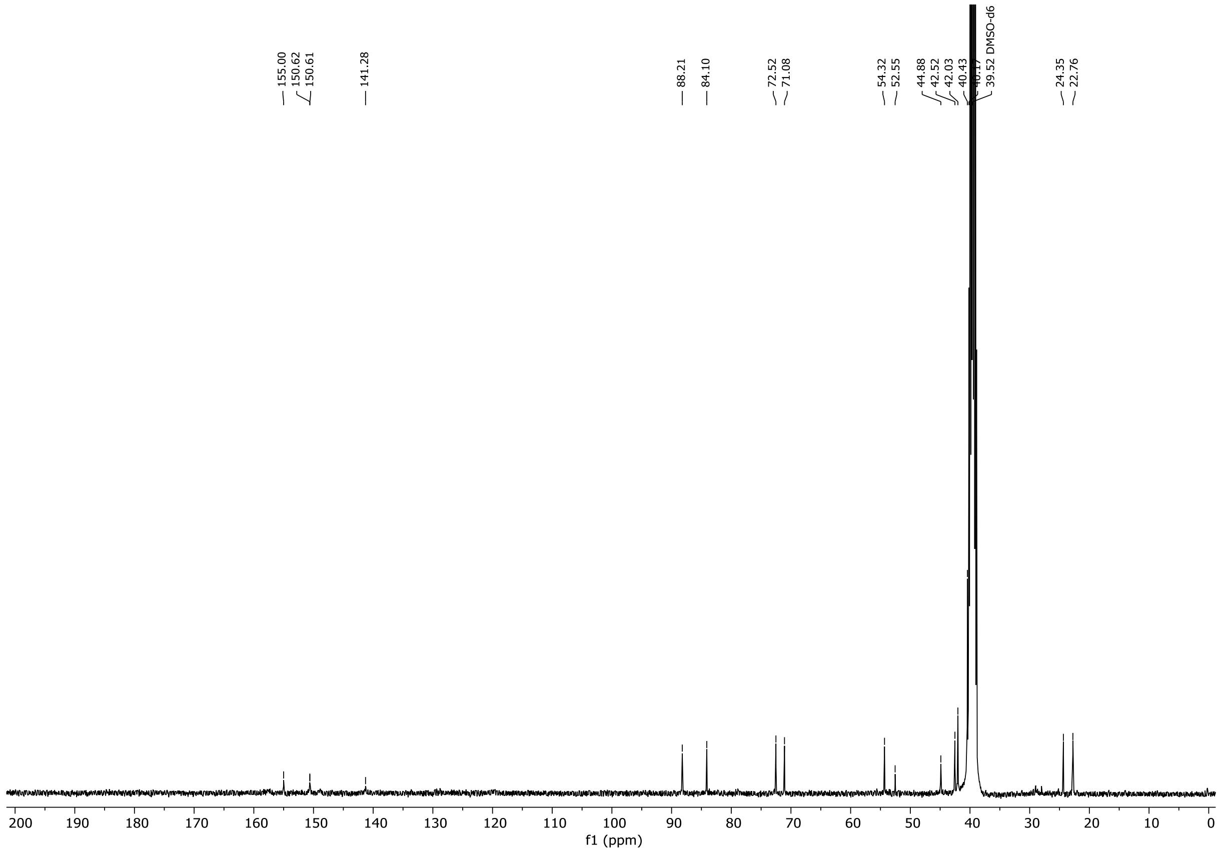

Figure S184. ^{13}C NMR of compound ***50***.

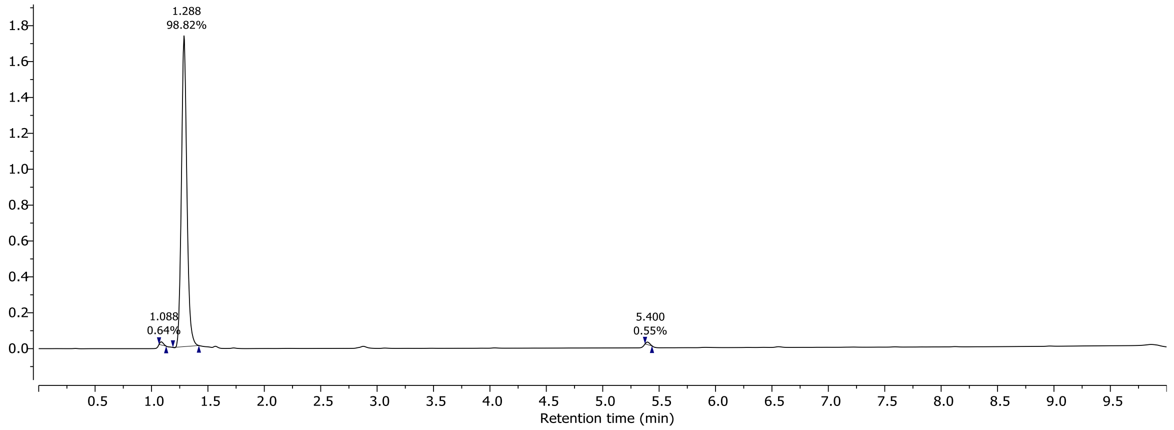

Figure S185. LCMS chromatogram of compound ***50*** at 254 nm.

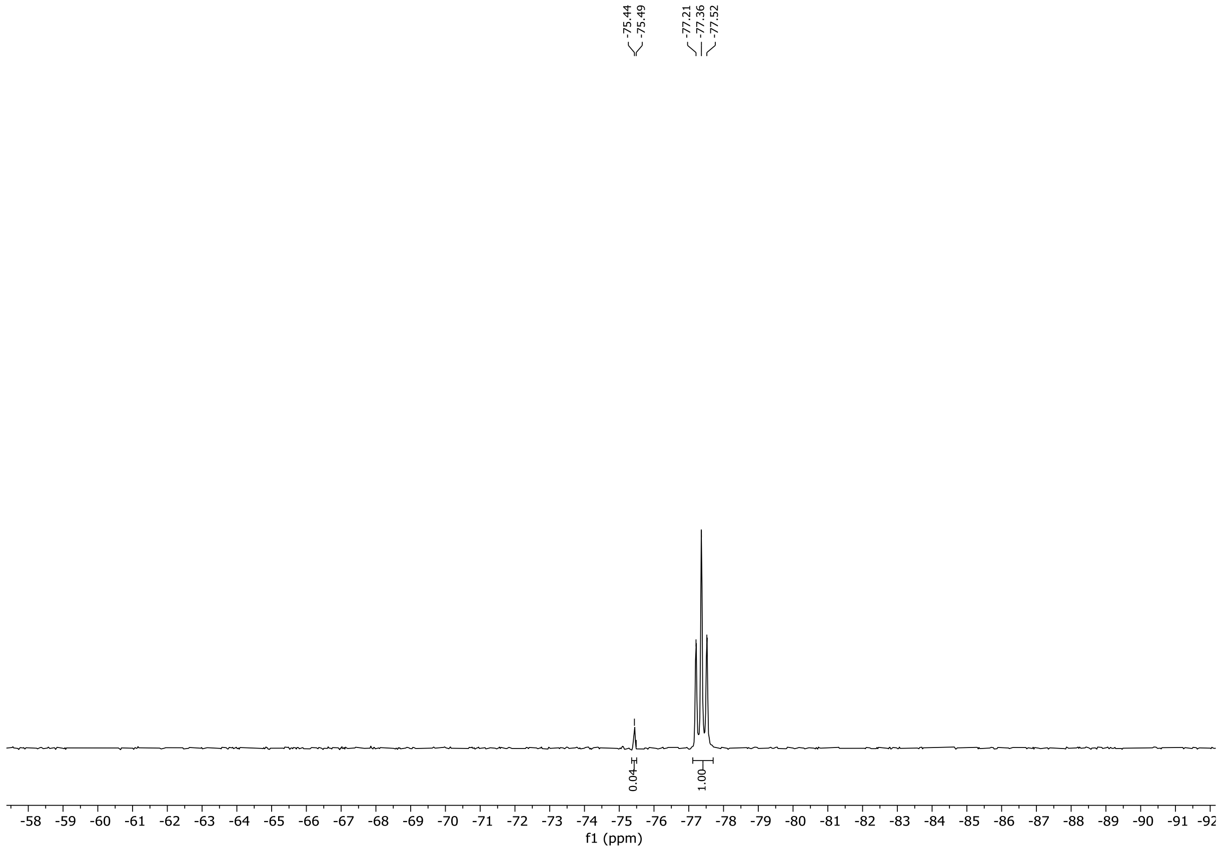

Figure S186. ^{19}F NMR of compound ***50*.**

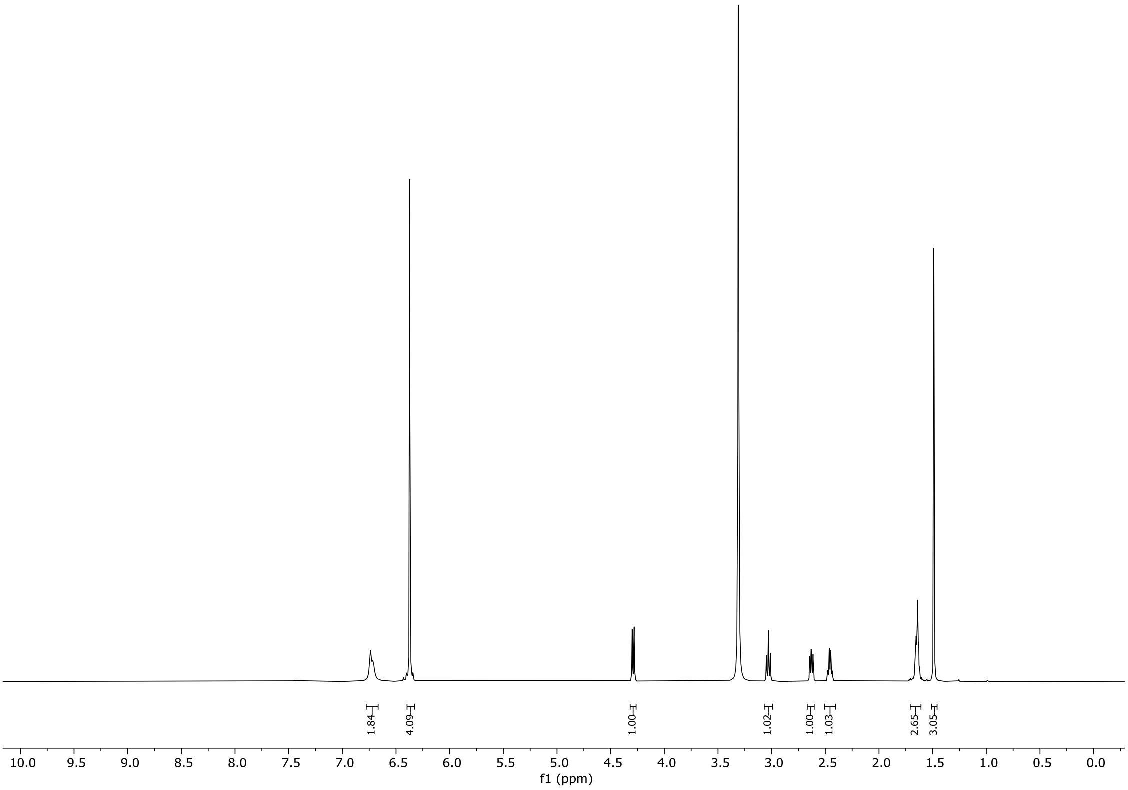

Figure S187. ^{1}H NMR of compound **51**.

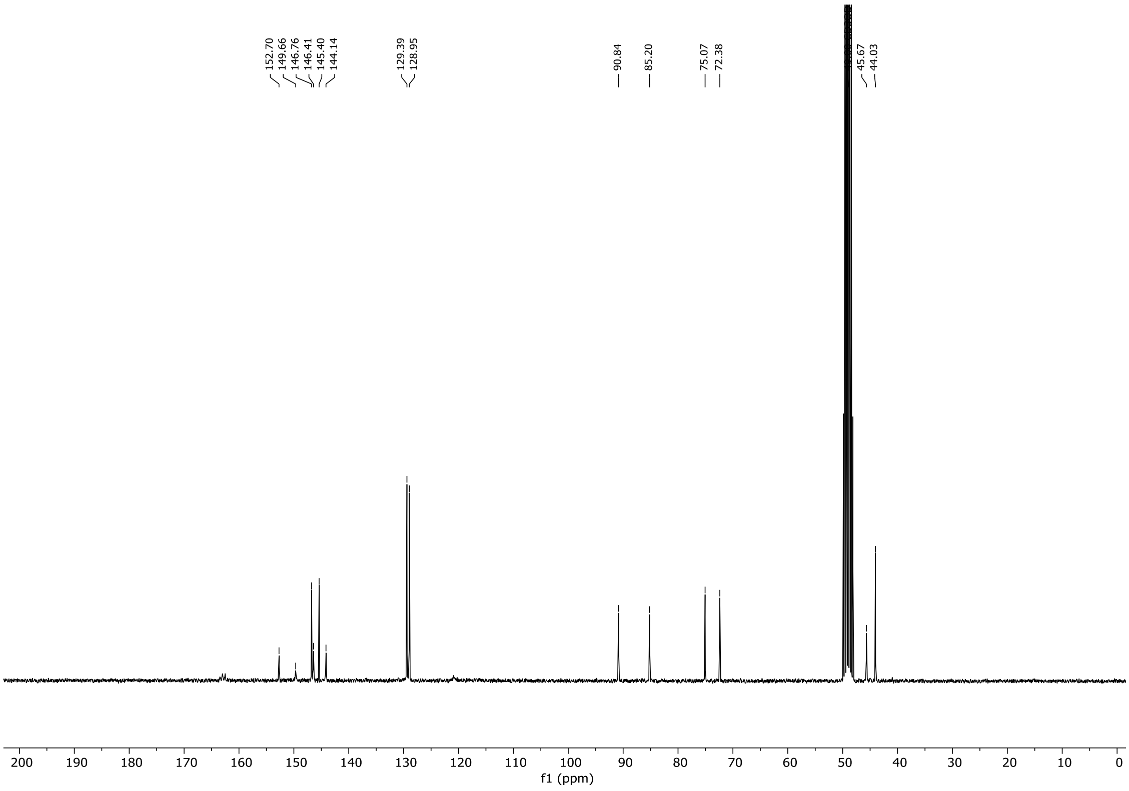

Figure S188. ^{13}C NMR of compound **51.**

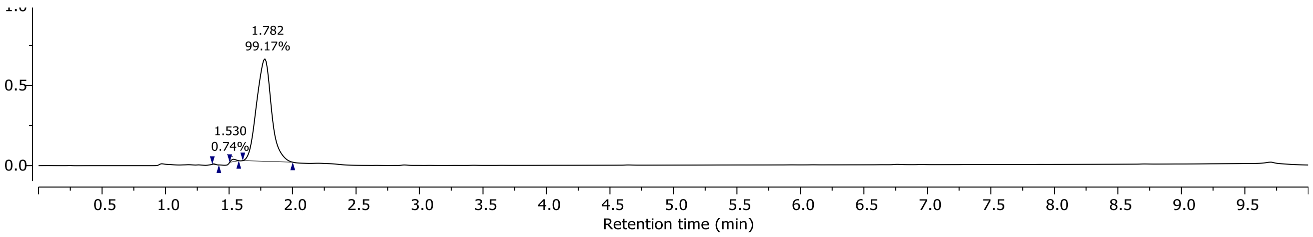

Figure S189. LCMS chromatogram of compound **51** at 254 nm.

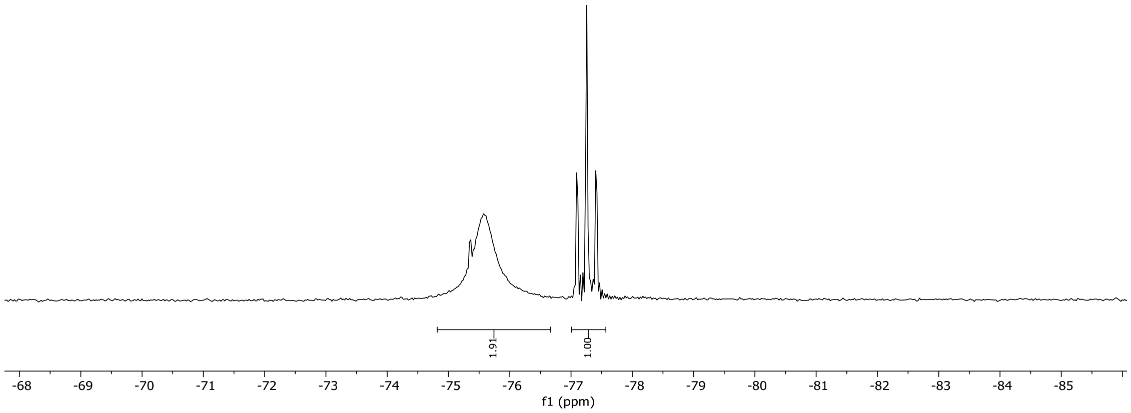

Figure S190. ^{19}F NMR of compound **51**.

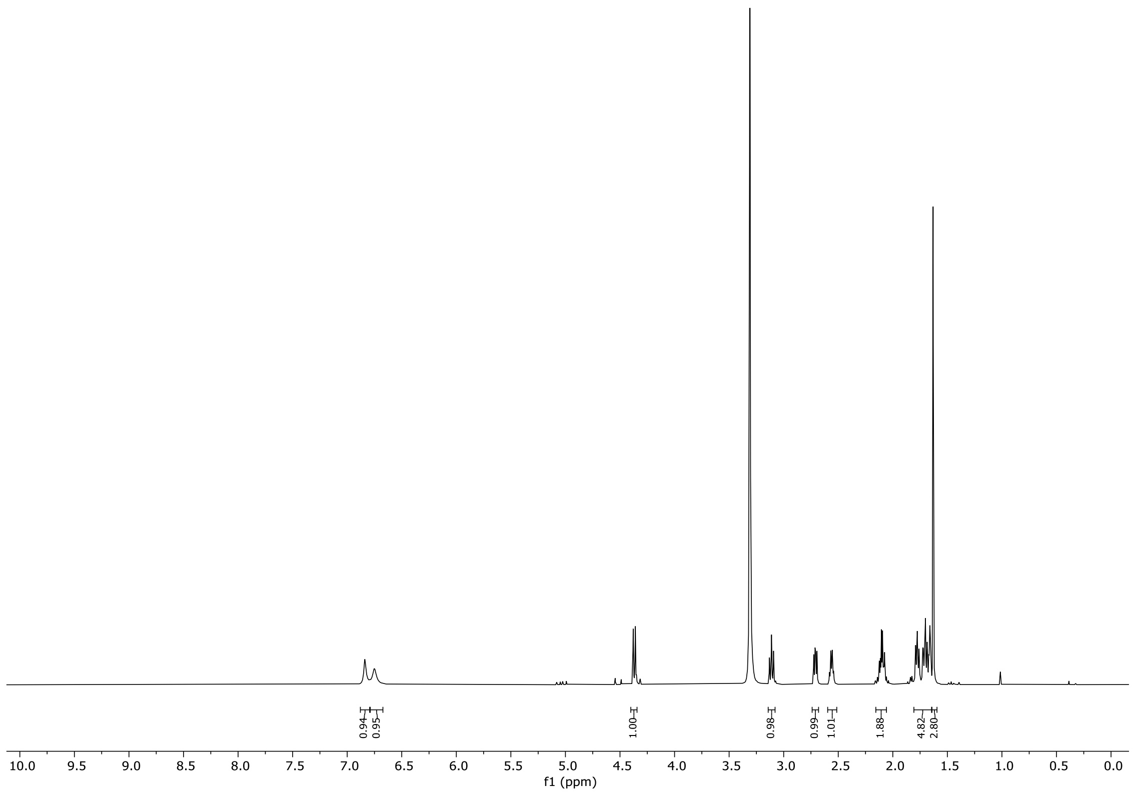

Figure S191. ^{1}H NMR of compound **52**.

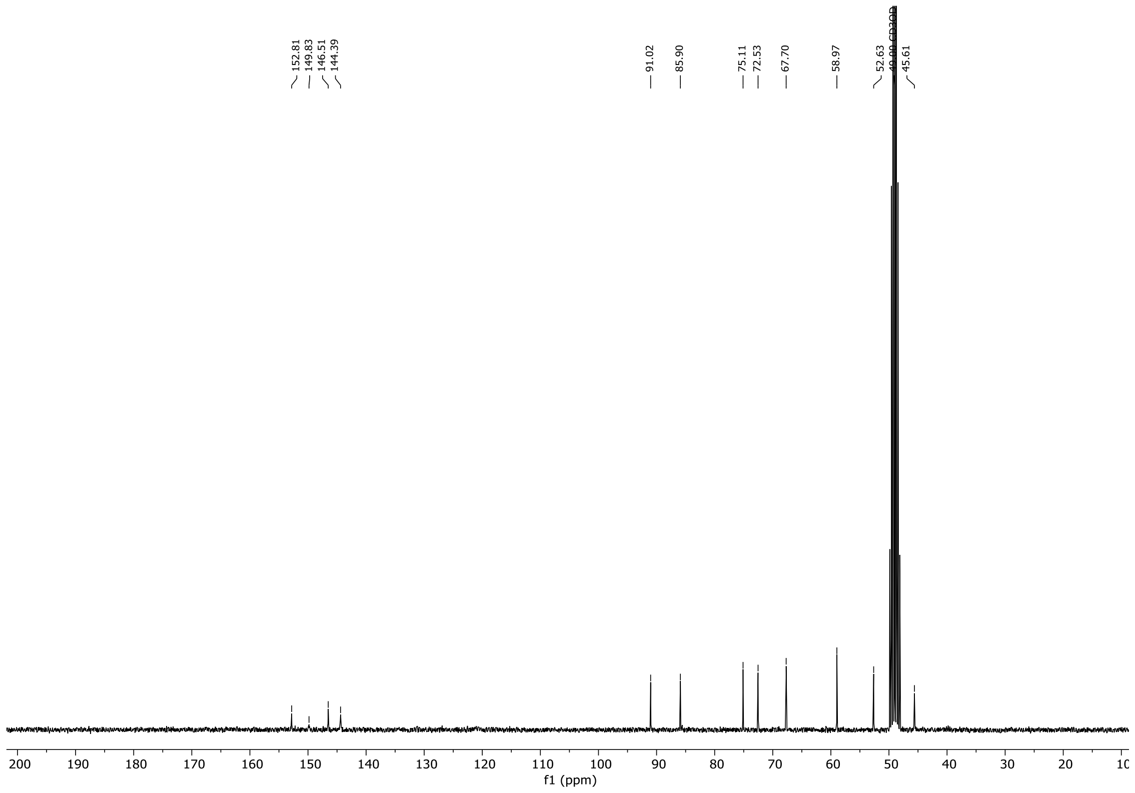

Figure S192. ^{13}C NMR of compound **53.**

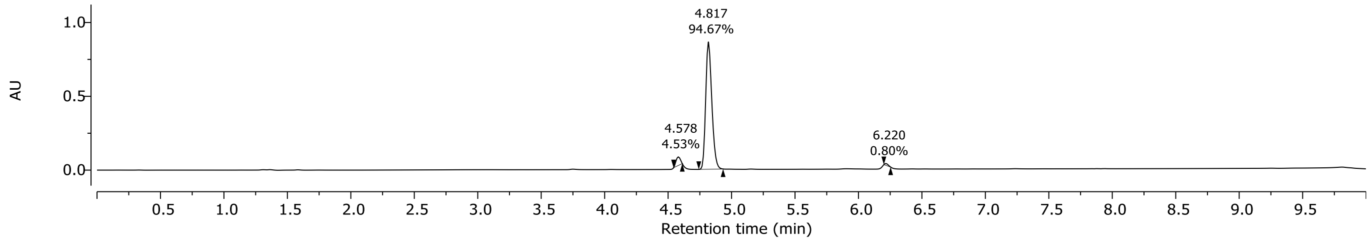

Figure S193. LCMS chromatogram of compound **53** at 254 nm.

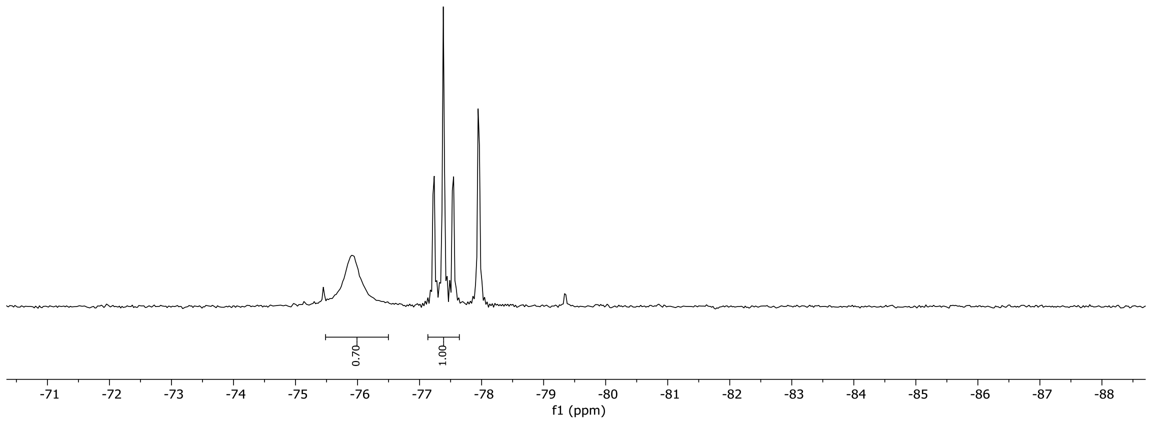

Figure S194. ^{19}F NMR of compound **53**.

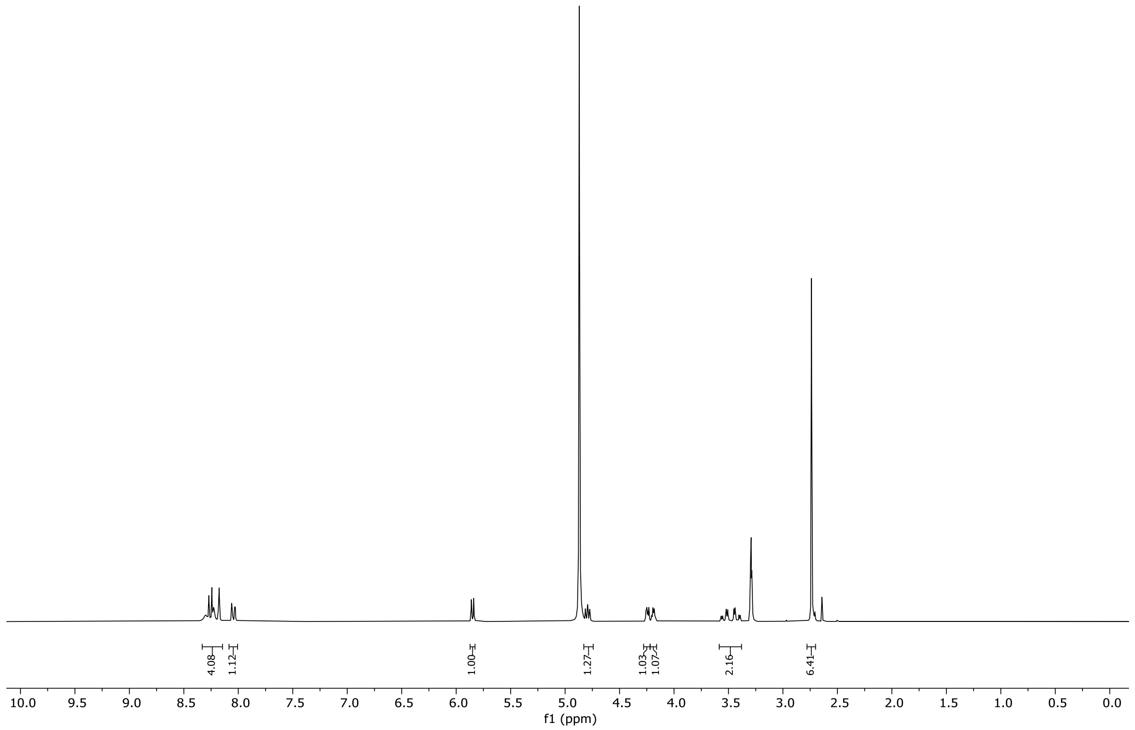

Figure S195. ^{1}H NMR of compound **54**.

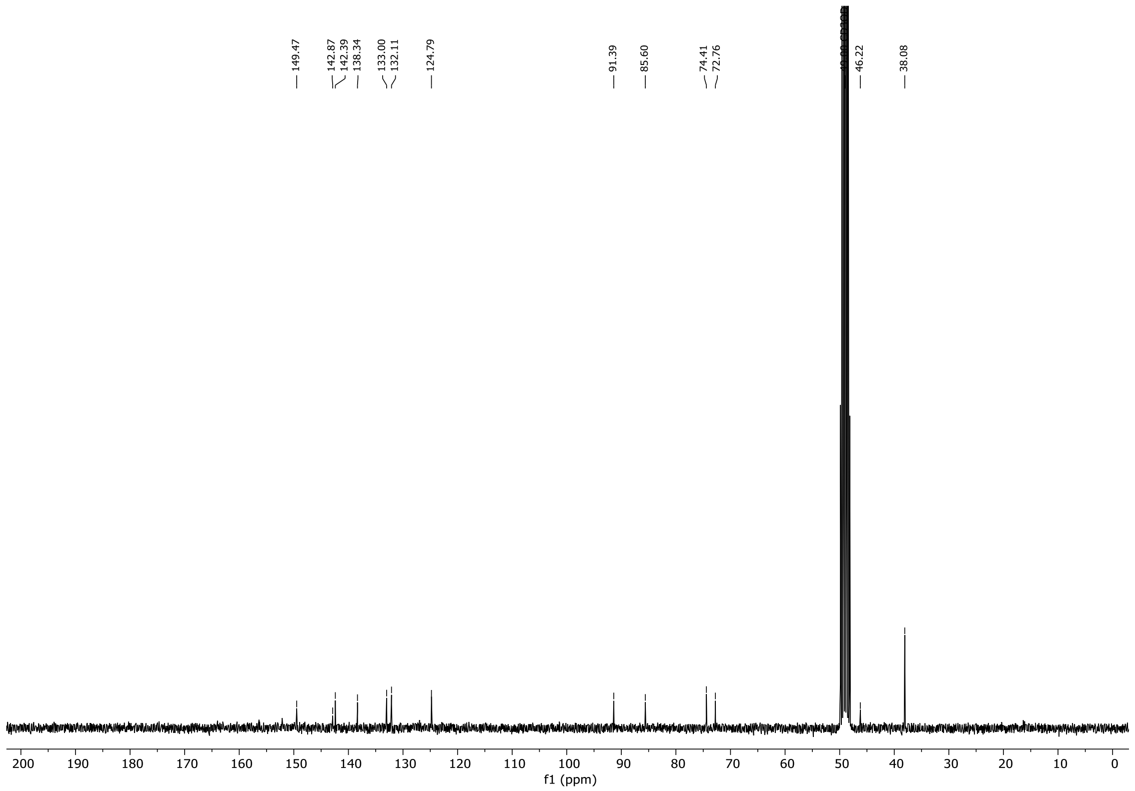

Figure S196. ^{13}C NMR of compound **54.**

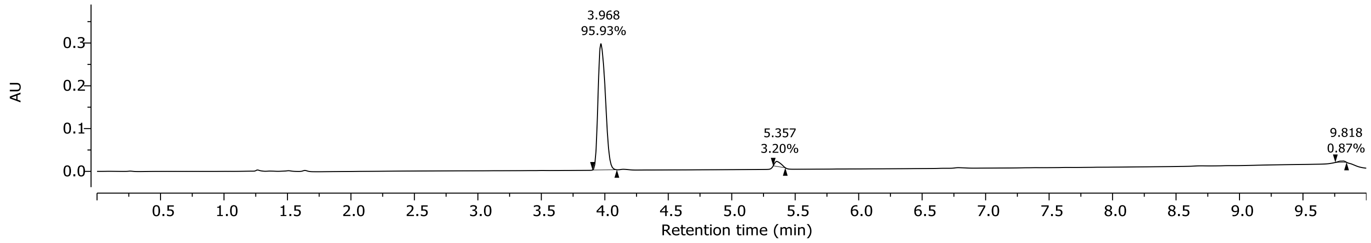

Figure S197. LCMS chromatogram of compound **54** at 254 nm.

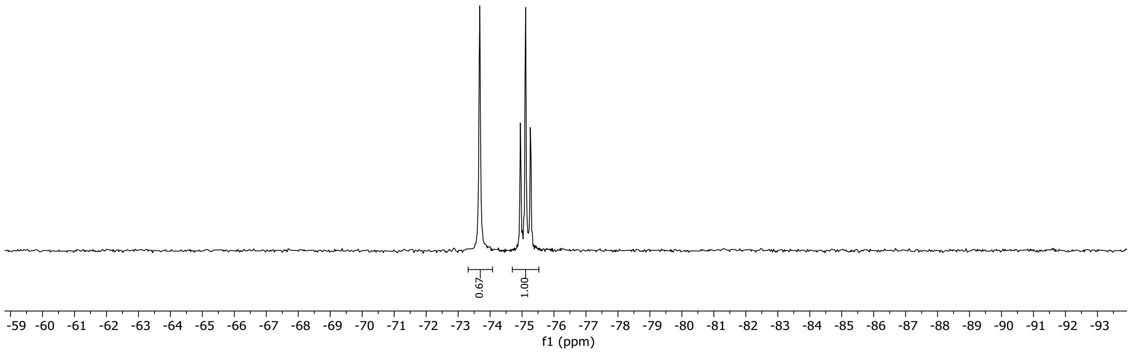

Figure S198. ^{19}F NMR of compound **54**

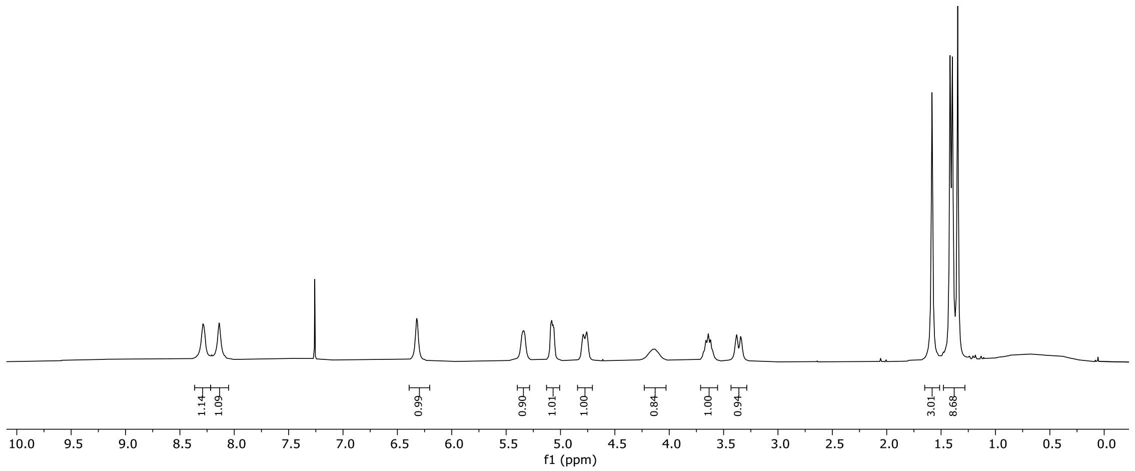

Figure S199. ^{1}H NMR of compound **55**.

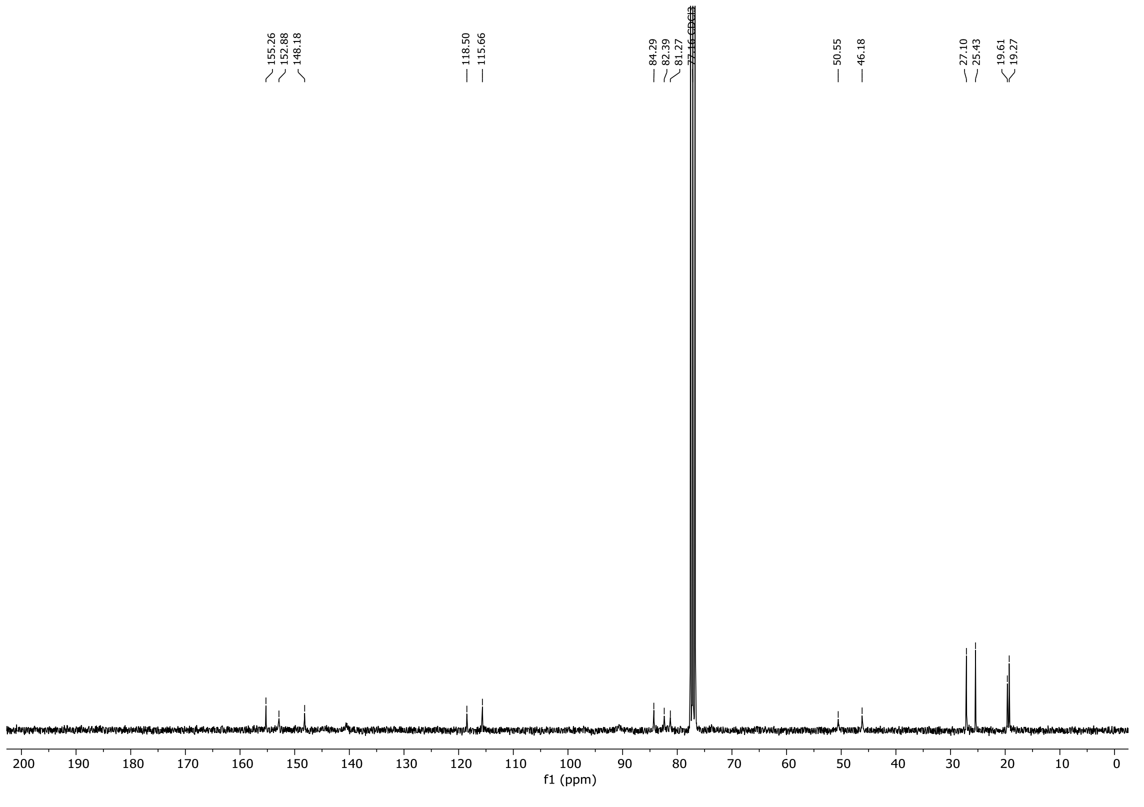

Figure S200. ^{13}C NMR of compound **55.**

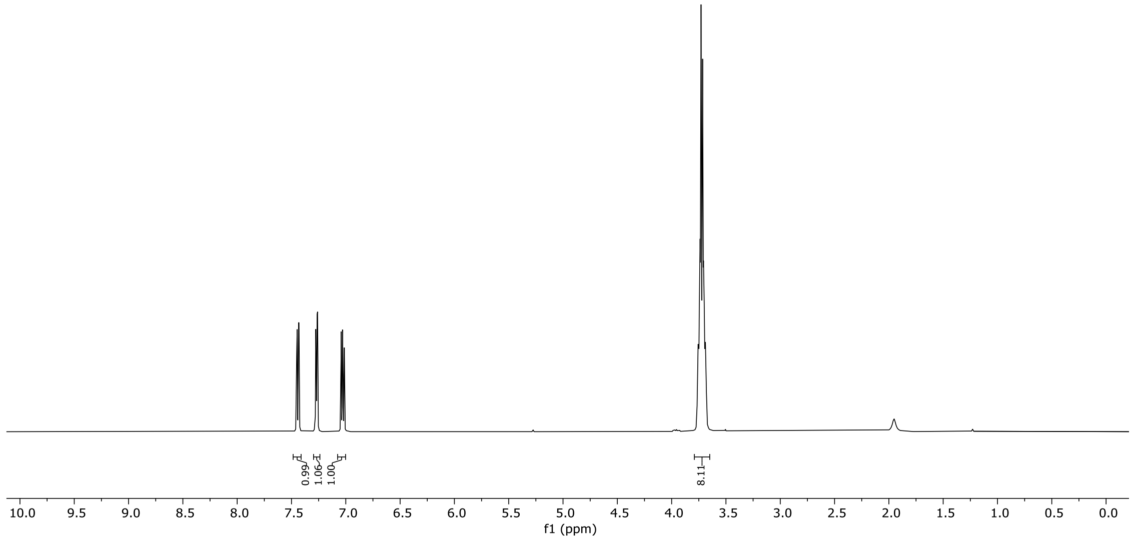

Figure S201. ^{1}H NMR of compound **58**.

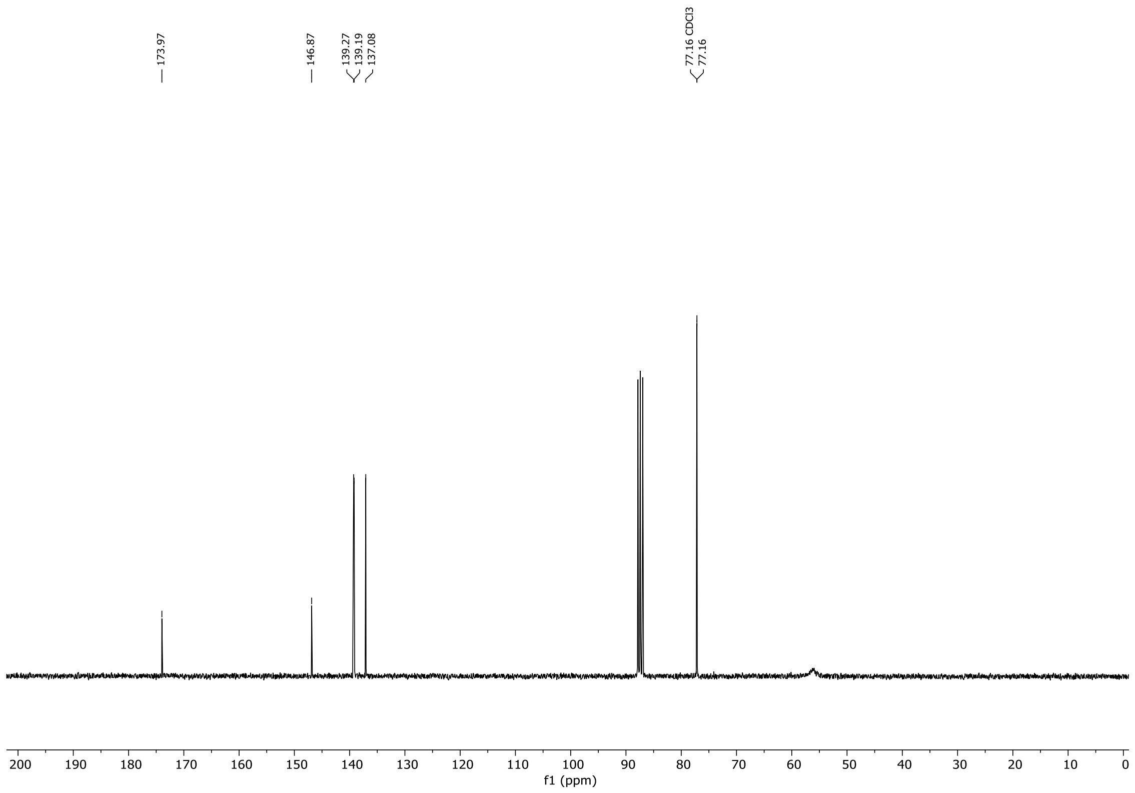

Figure S202. ^{13}C NMR of compound **58.**

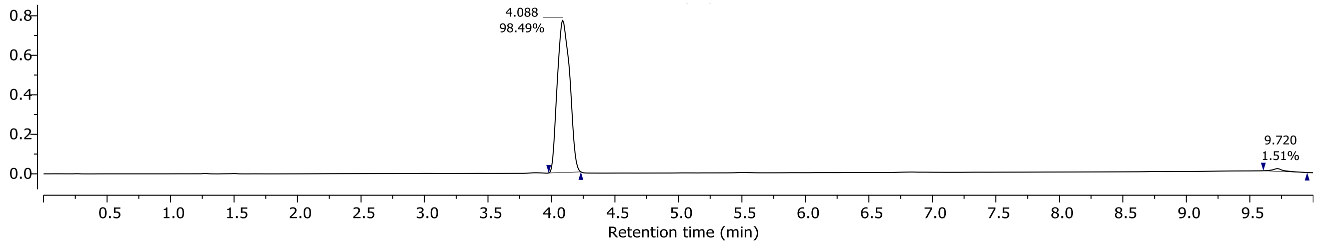

Figure S203. LCMS chromatogram of compound **58** at 254 nm.

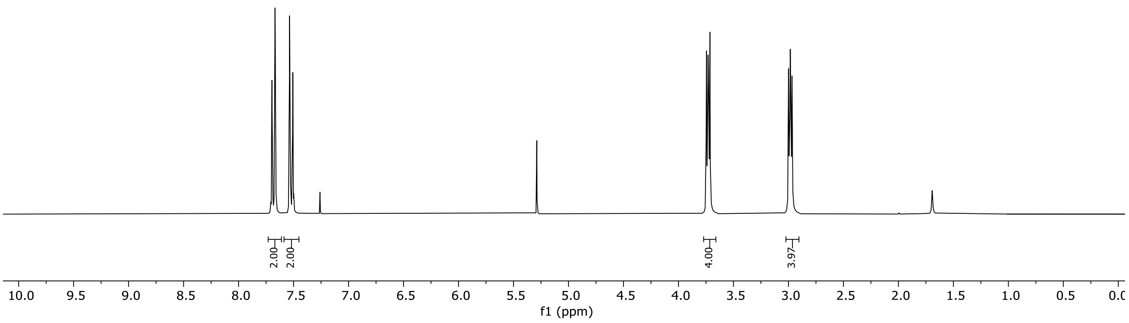

Figure S204. ^{1}H NMR of compound **59**.

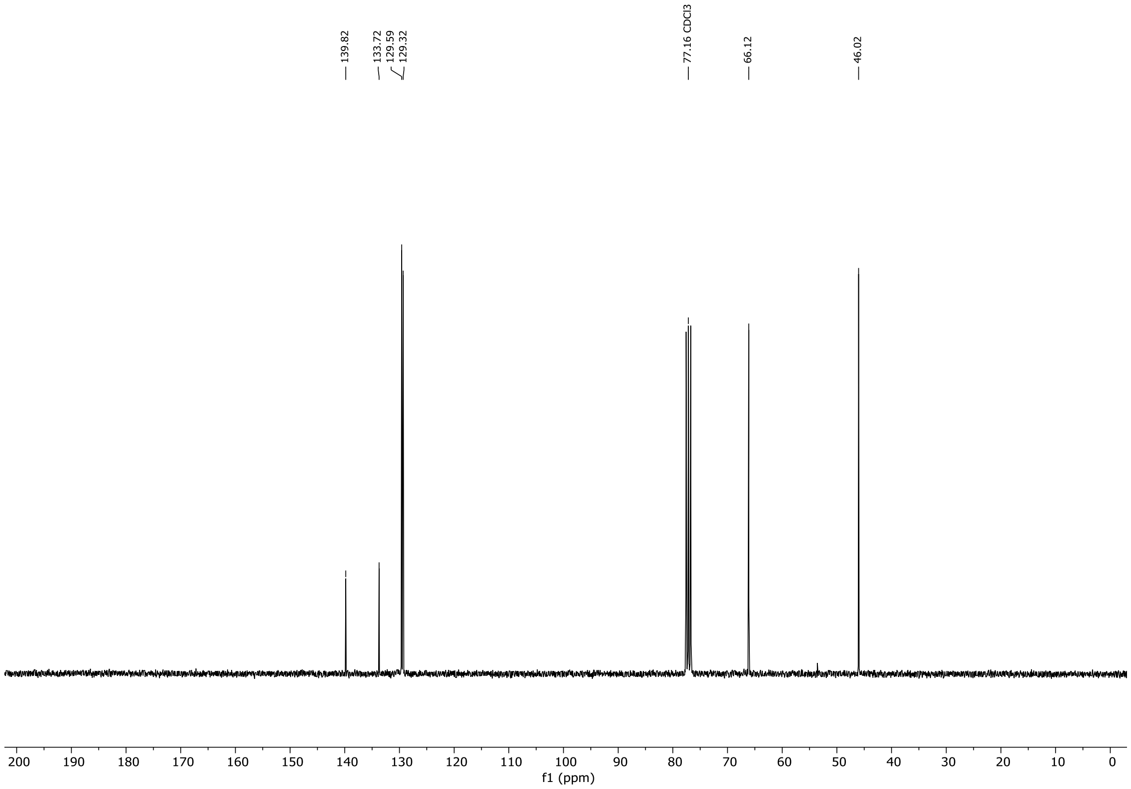

Figure S205. ^{13}C NMR of compound **59.**

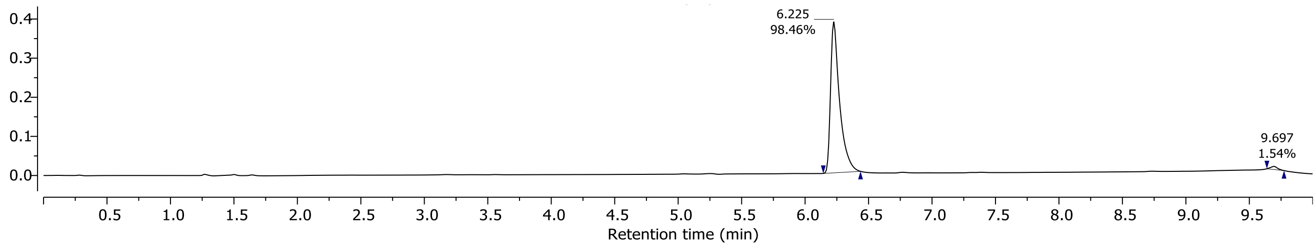

Figure S206. LCMS chromatogram of compound **59** at 254 nm.

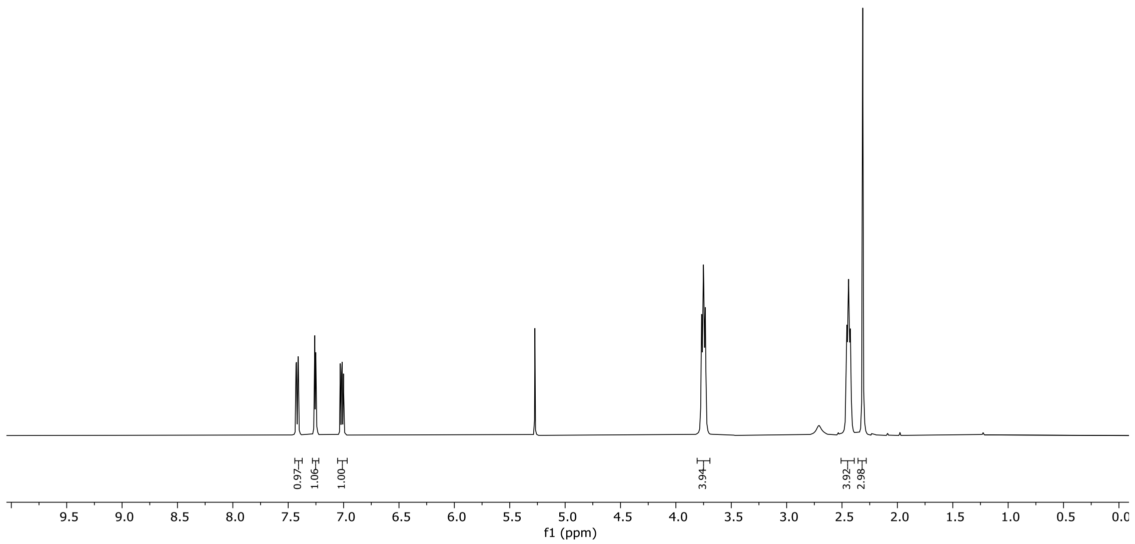

Figure S207. ^{1}H NMR of compound **60**.

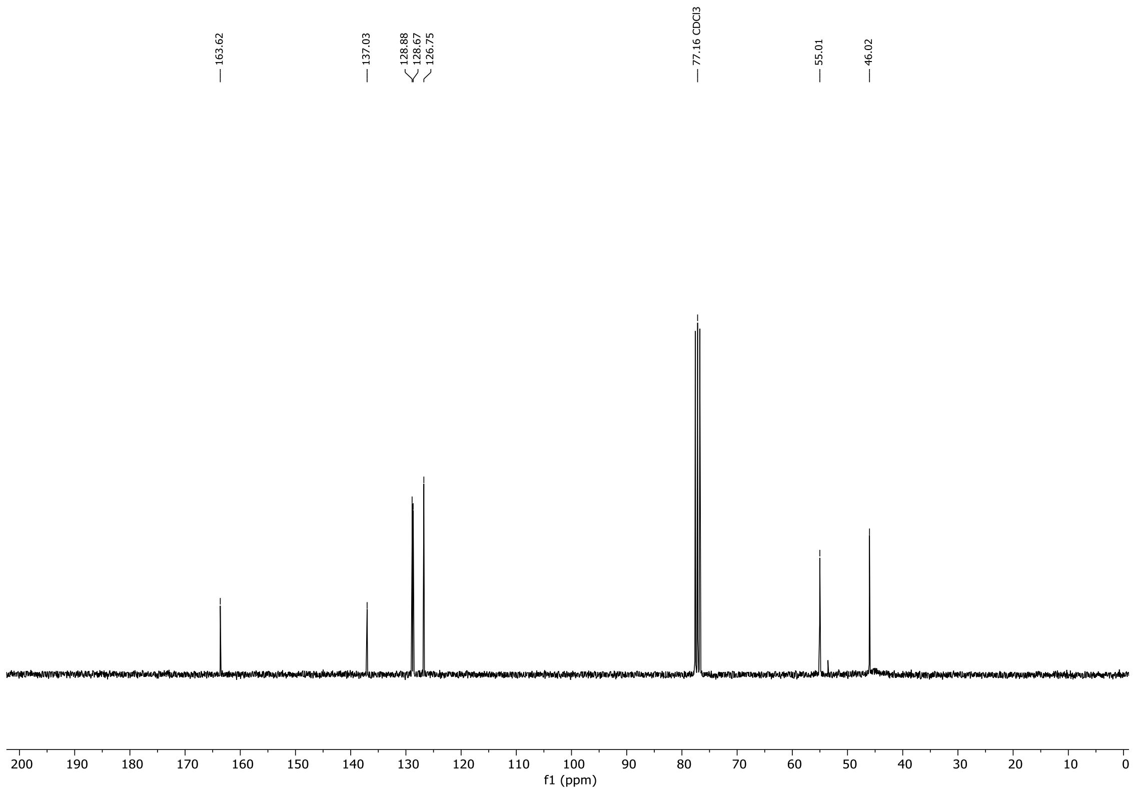

Figure S208. ^{13}C NMR of compound **60.**

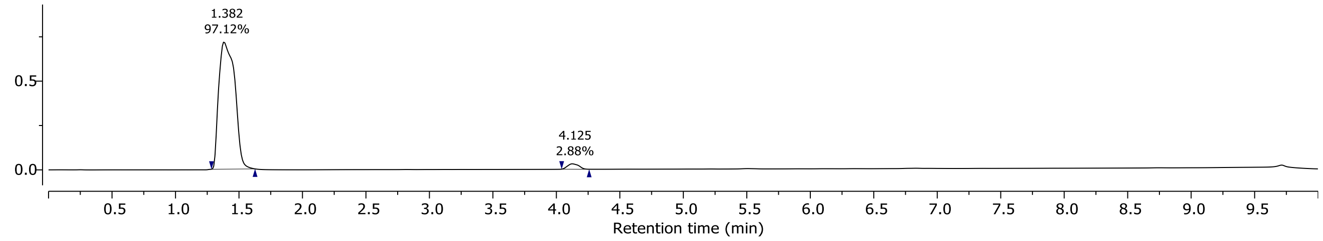

Figure S209. LCMS chromatogram of compound **60** at 254 nm.

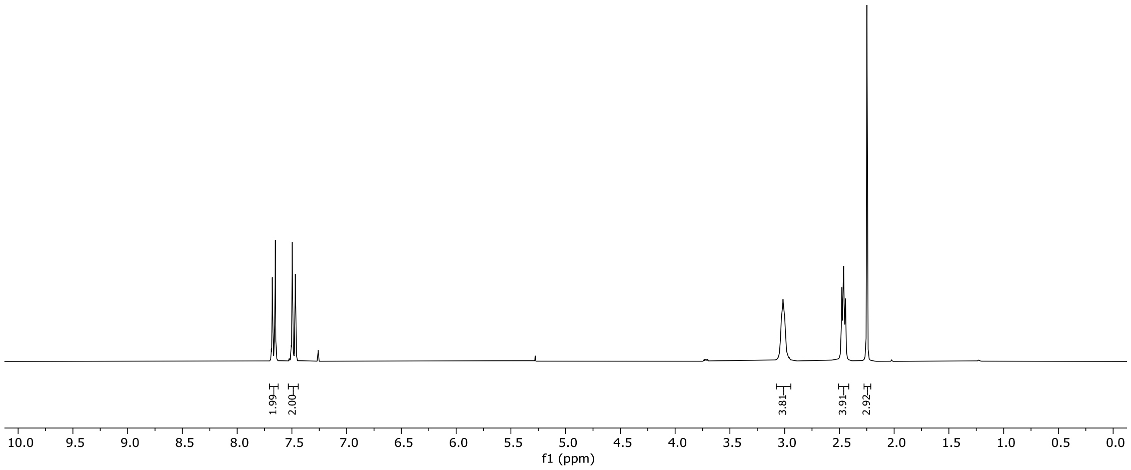

Figure S210. ^{1}H NMR of compound **61**.

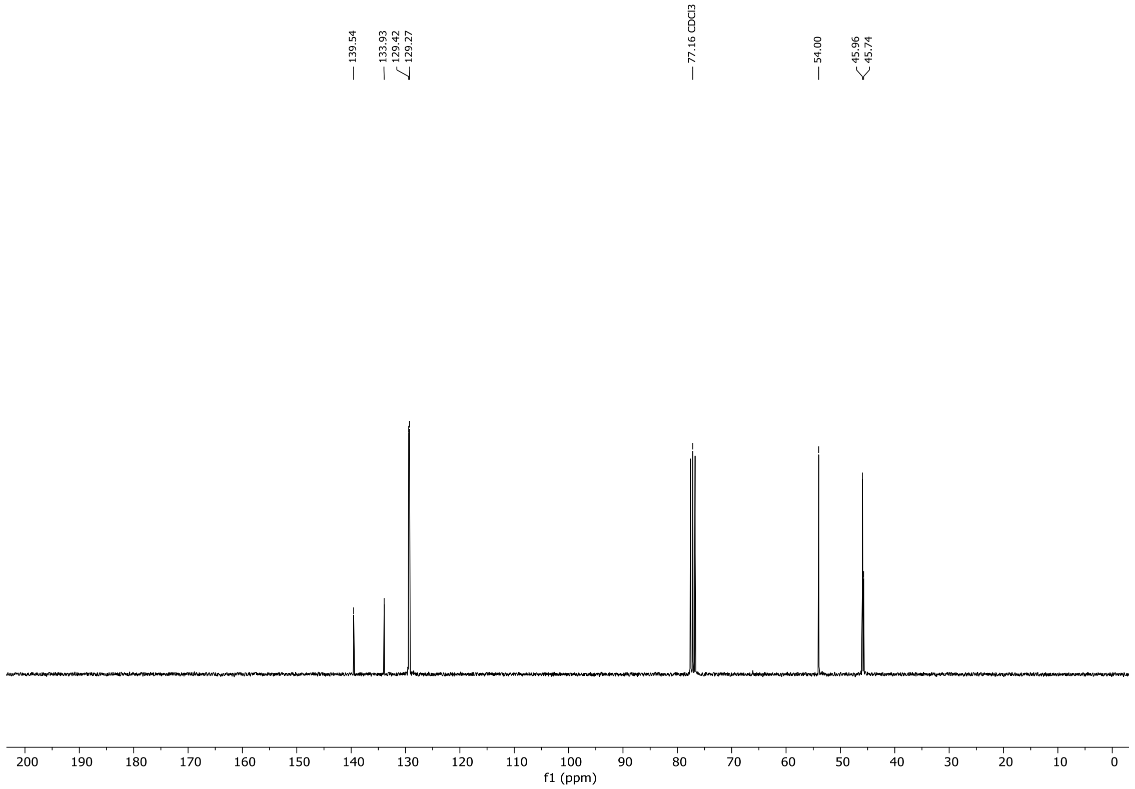

Figure S211. ^{13}C NMR of compound **61.**

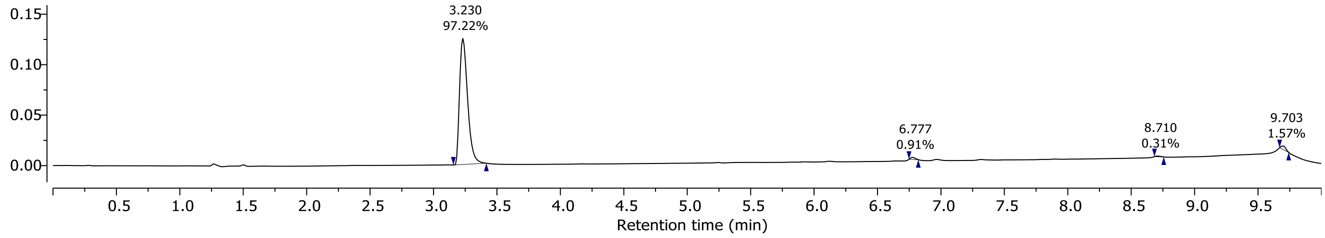

Figure S212. LCMS chromatogram of compound **61** at 254 nm.

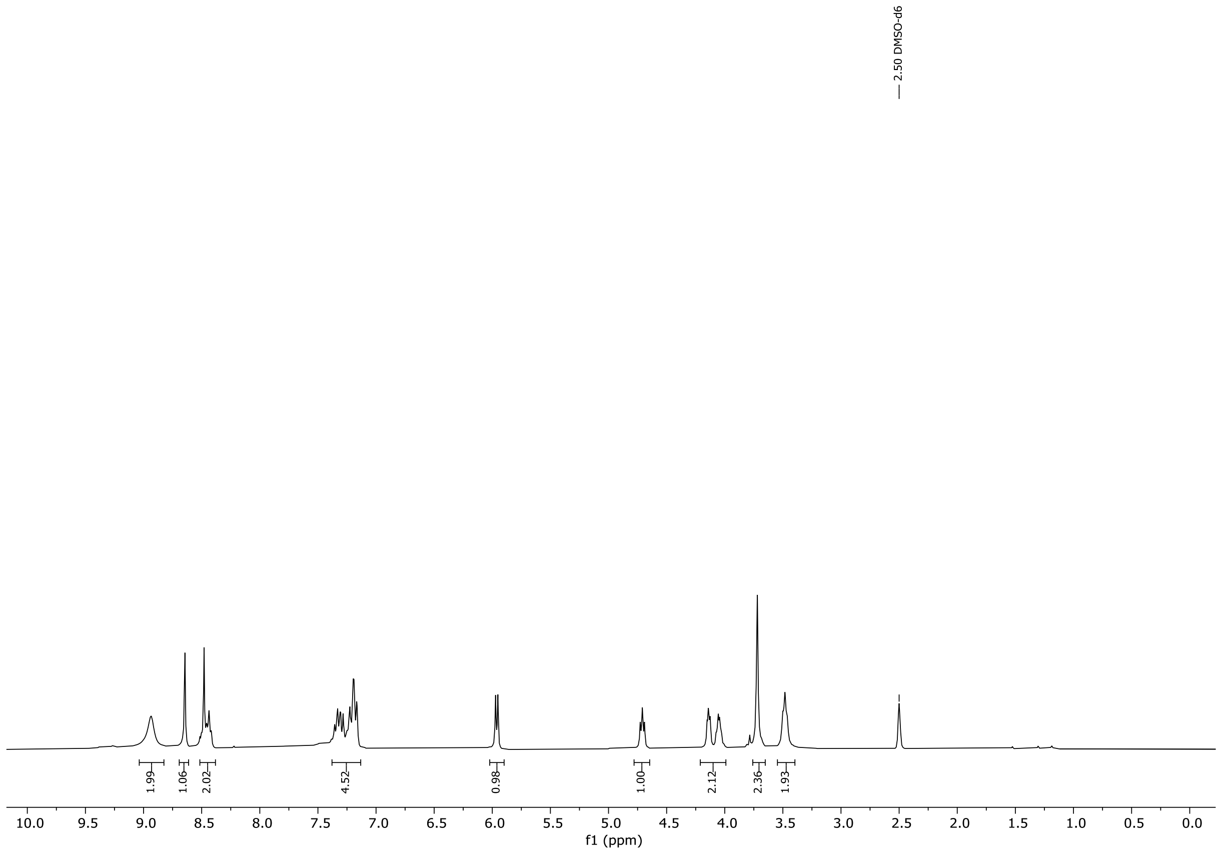

Figure S213. ^{1}H NMR of compound ***63***.

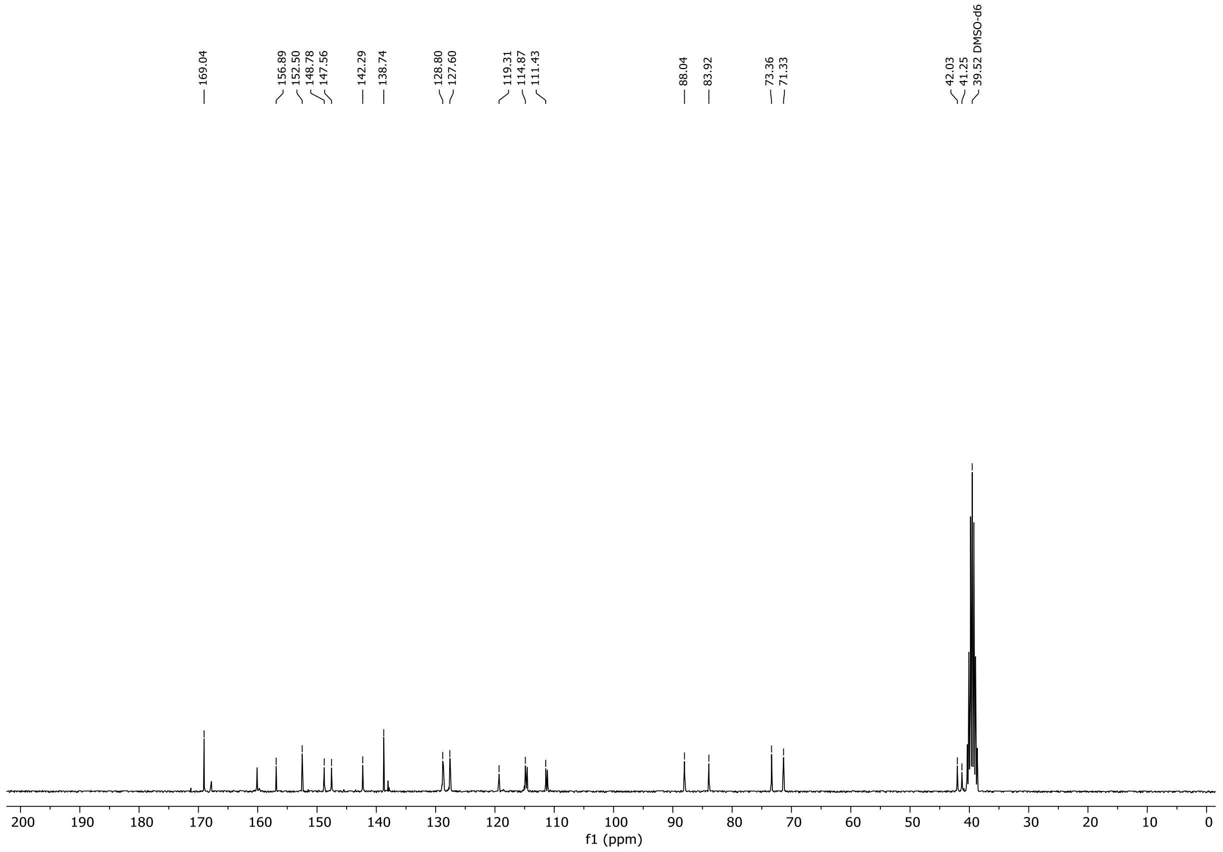

Figure S214. ^{13}C NMR of compound ***63.***

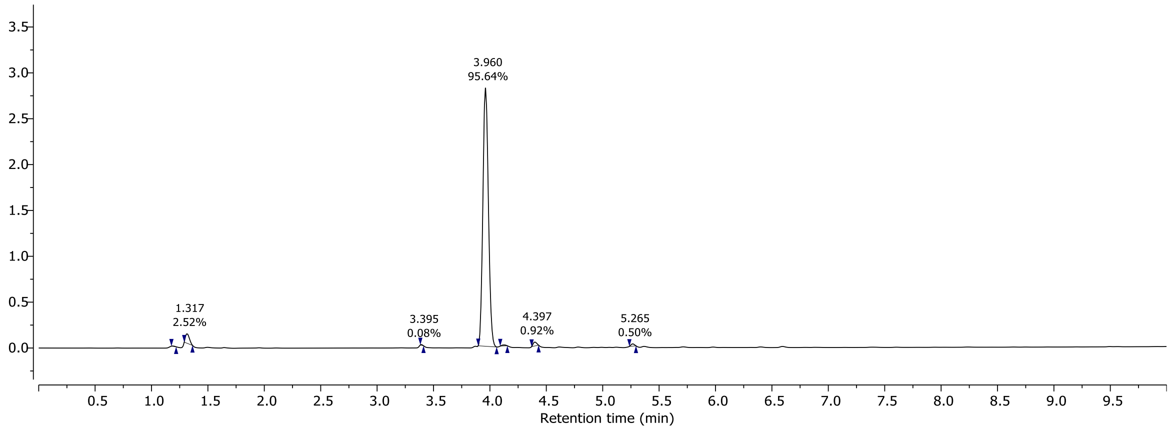

Figure S215. LCMS chromatogram of compound ***63*** at 254 nm.

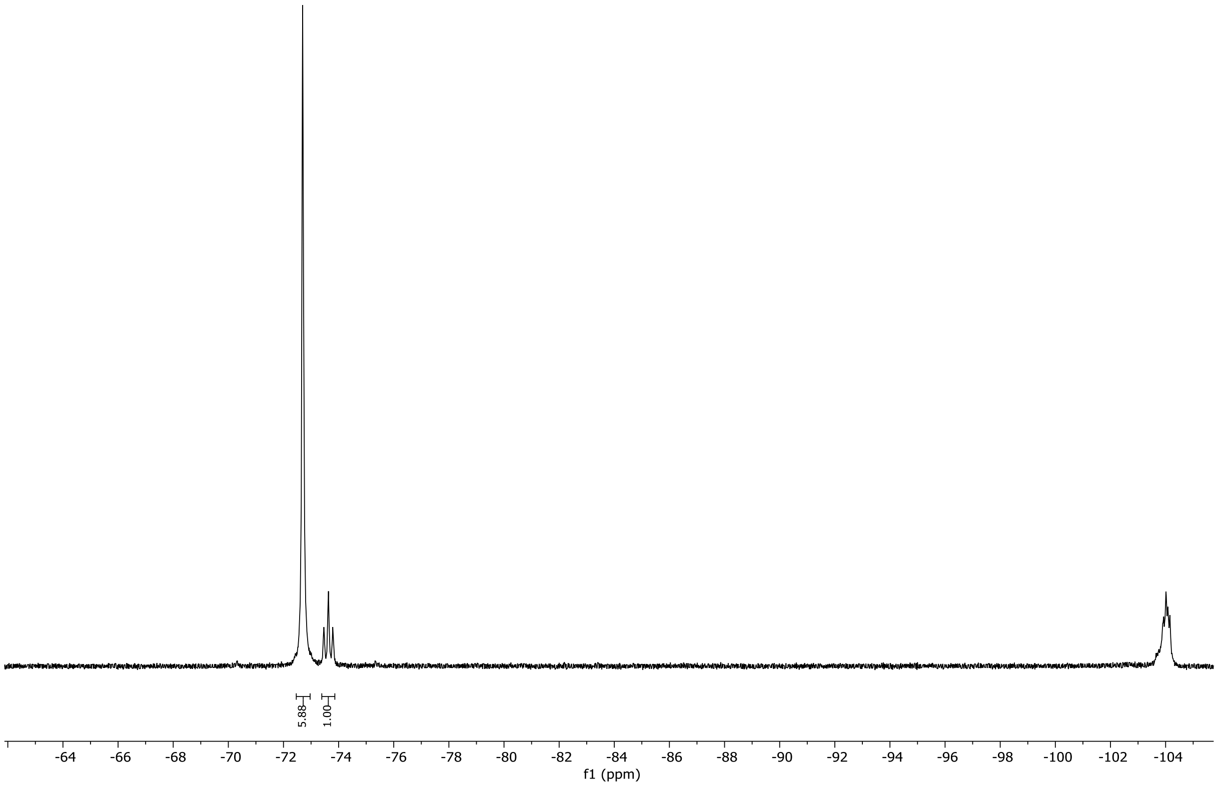

Figure S216. ^{19}F NMR of compound ***63***.

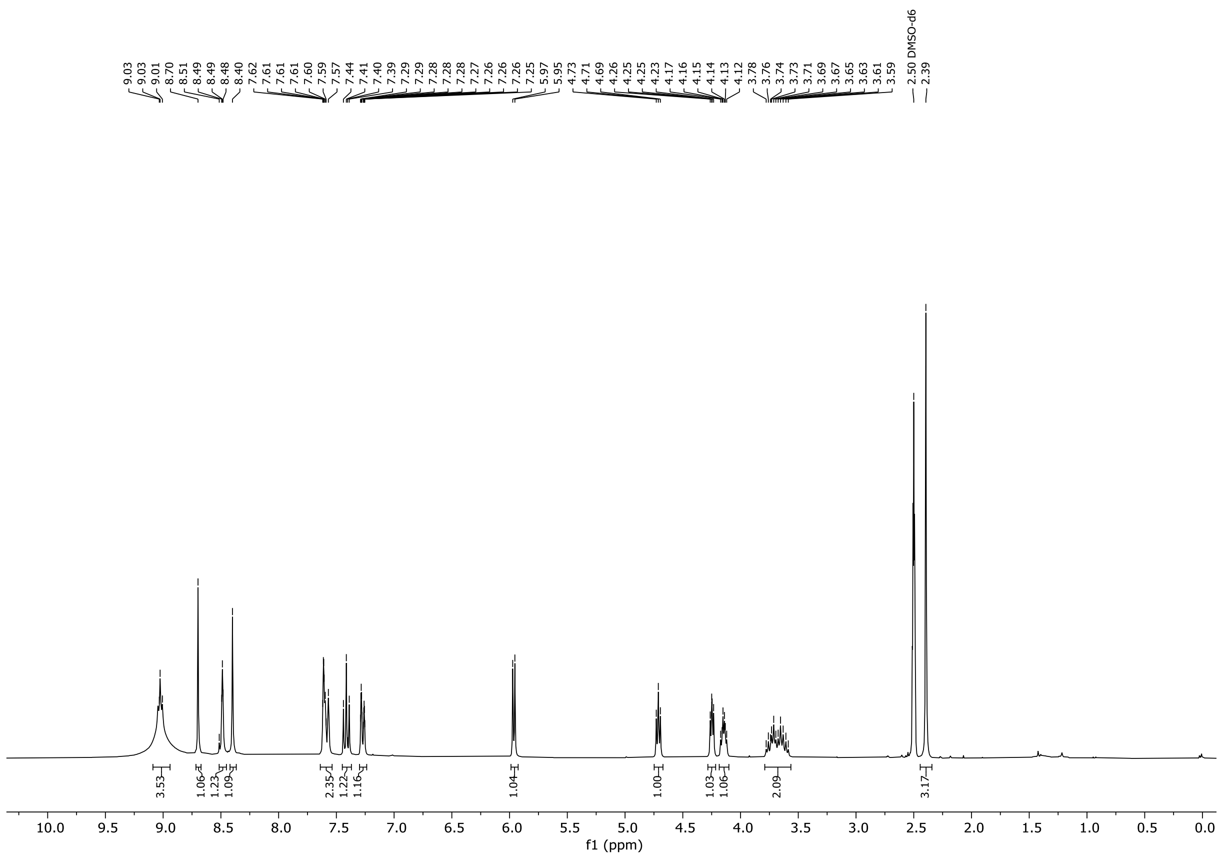

Figure S217. ^{1}H NMR of compound ***64***.

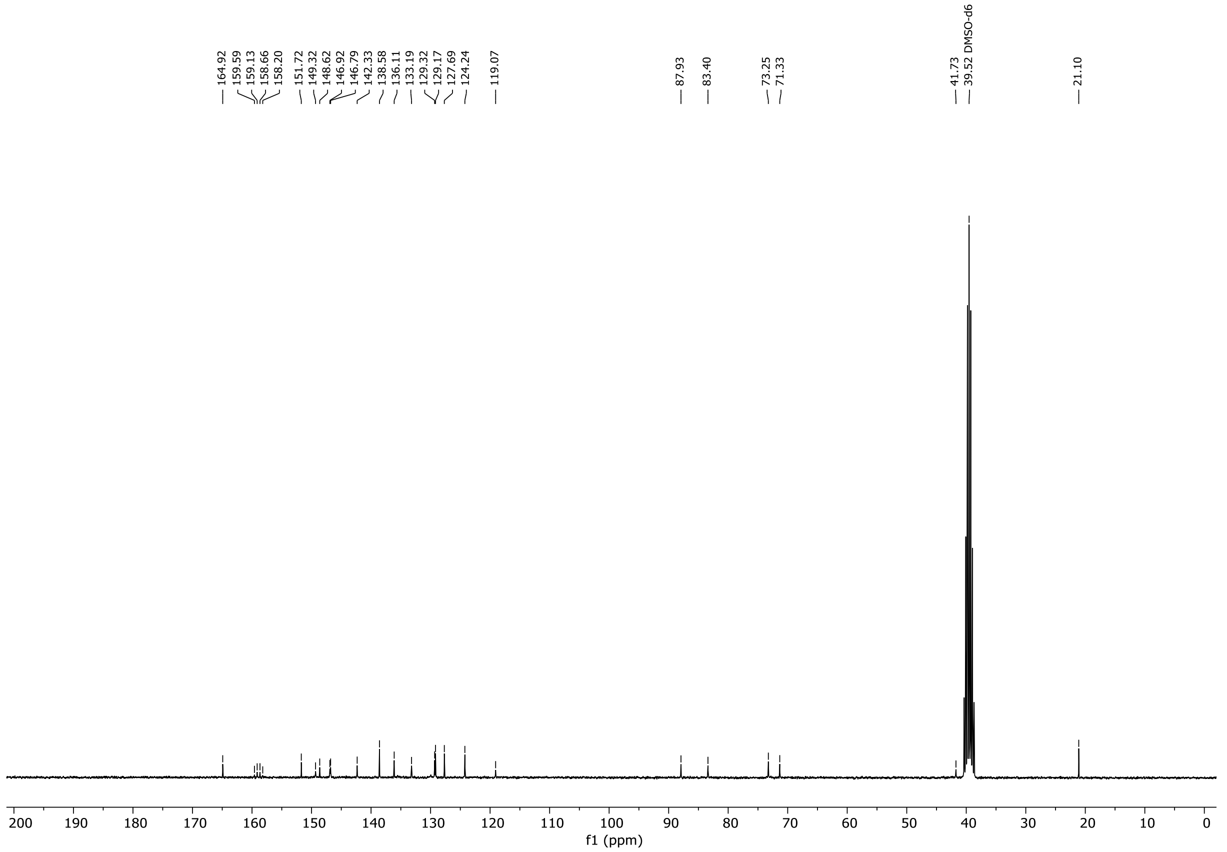

Figure S218. ^{13}C NMR of compound ***64***.

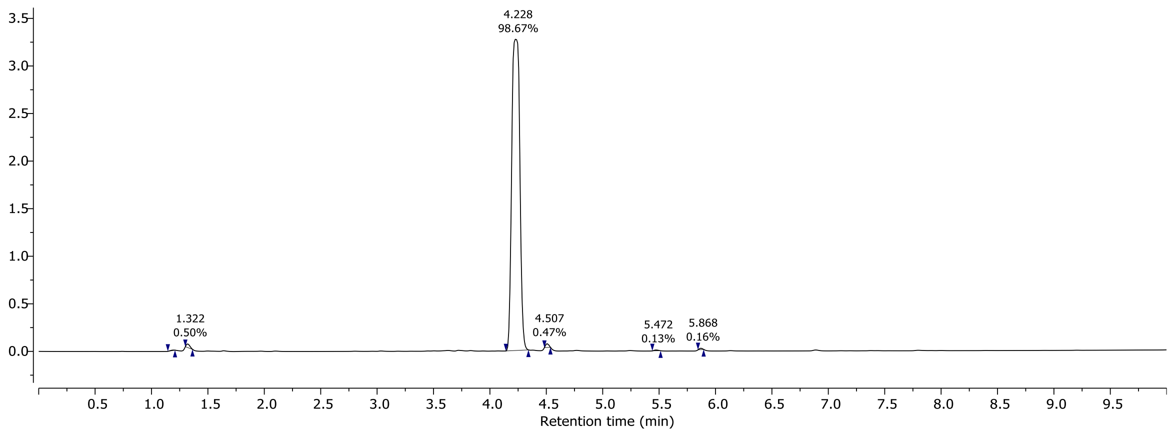

Figure S219. LCMS chromatogram of compound **64** at 254 nm.

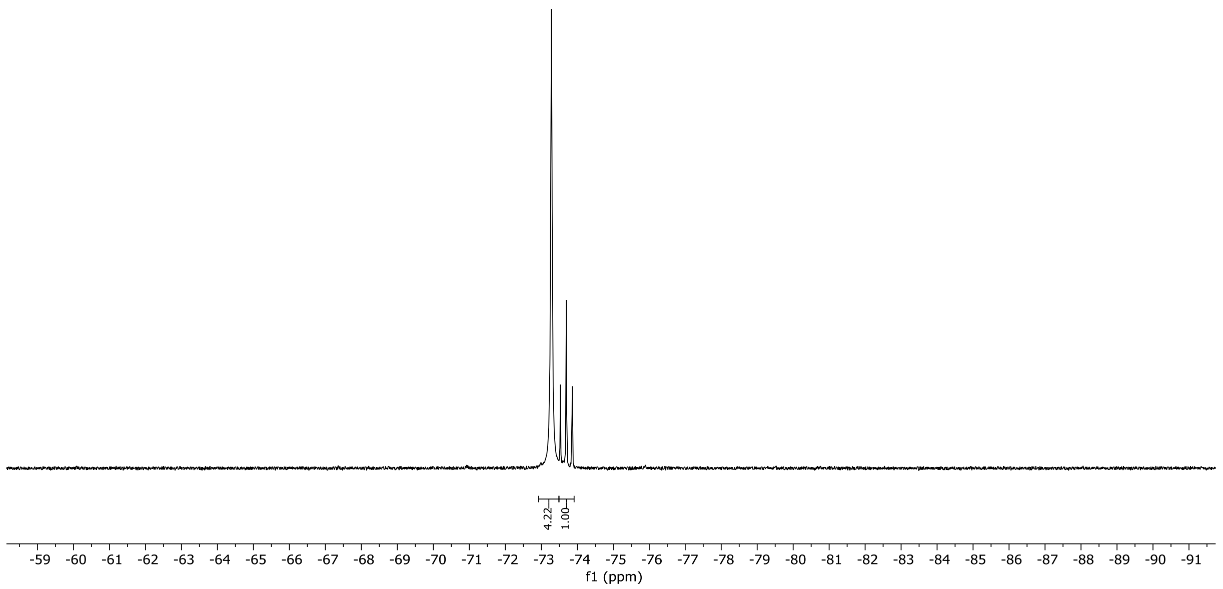

Figure S220. ^{19}F NMR of compound **64.**

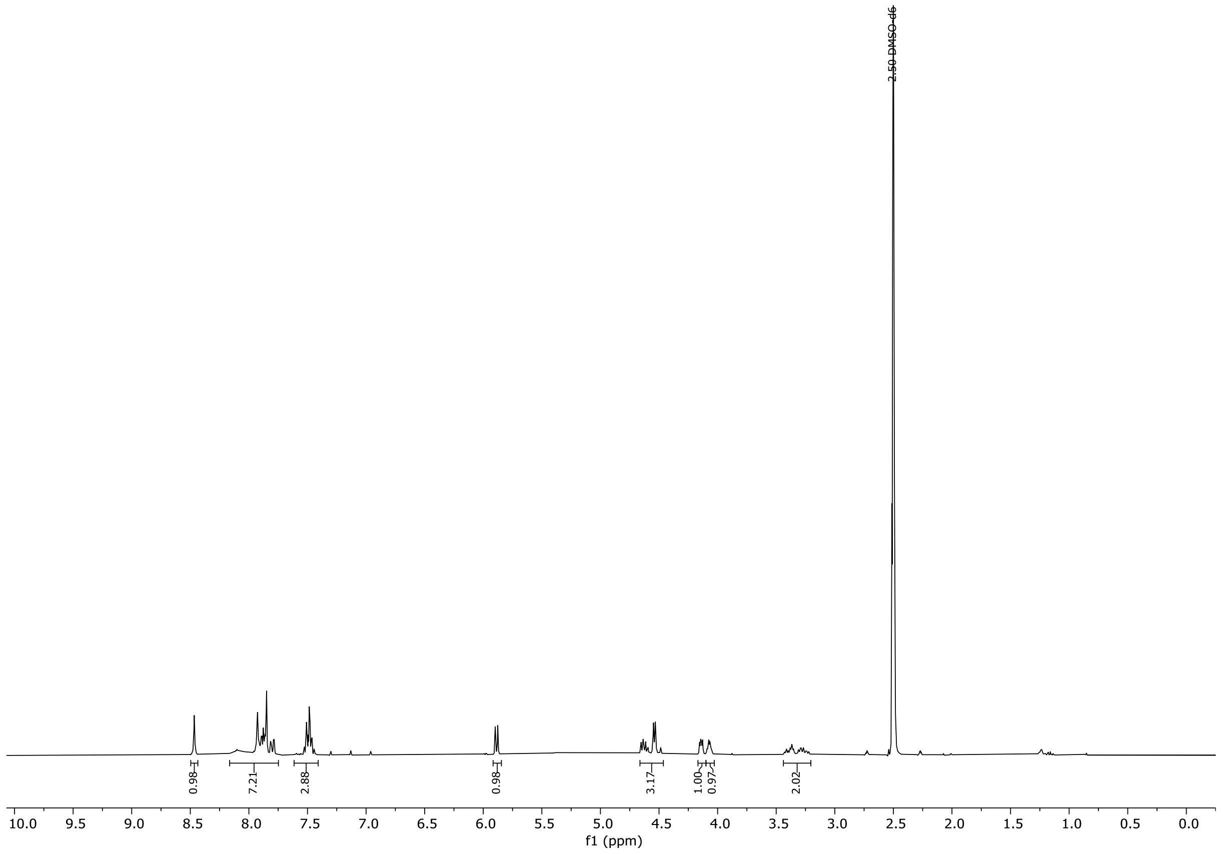

Figure S221. ^{1}H NMR of compound ***65***.

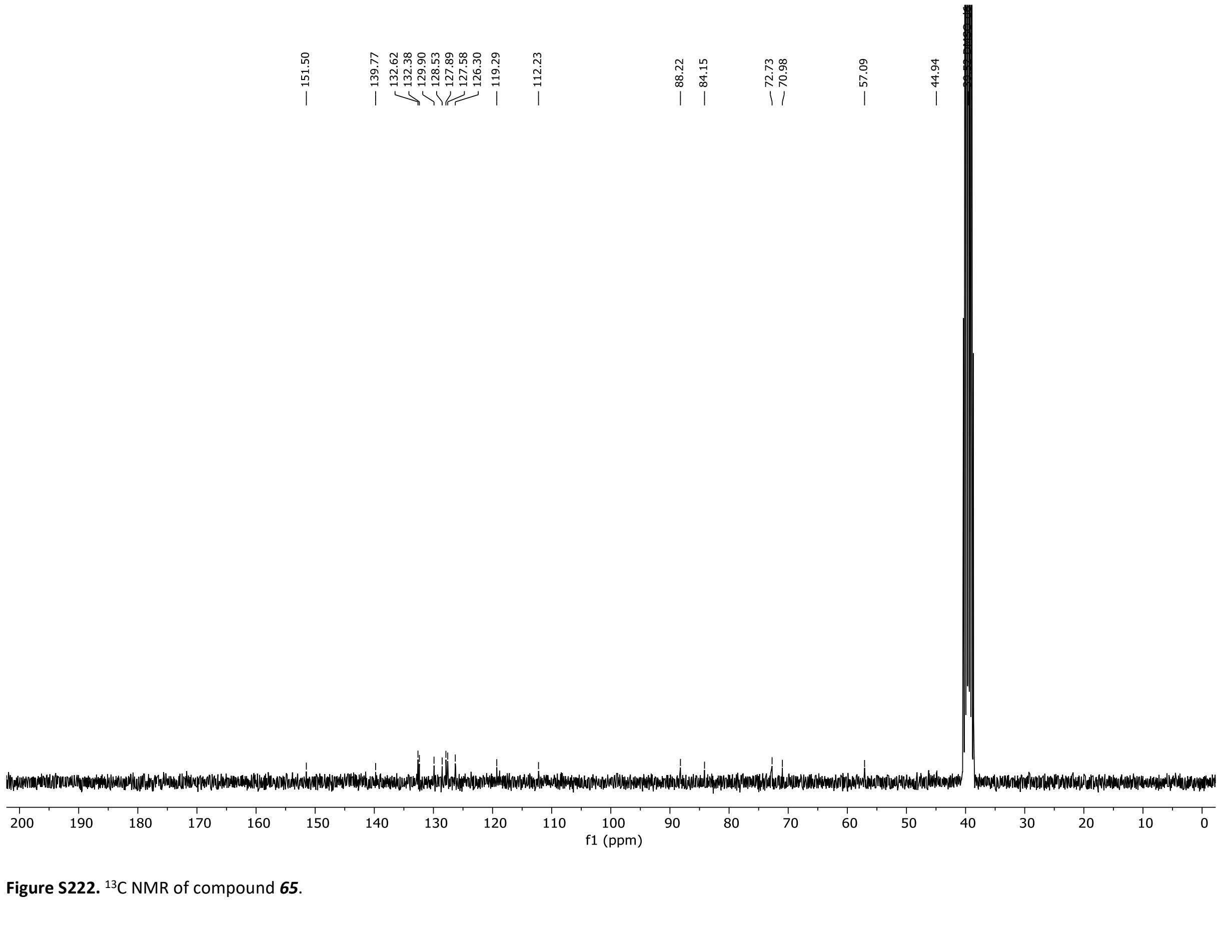

Figure S222. ^{13}C NMR of compound ***65***.

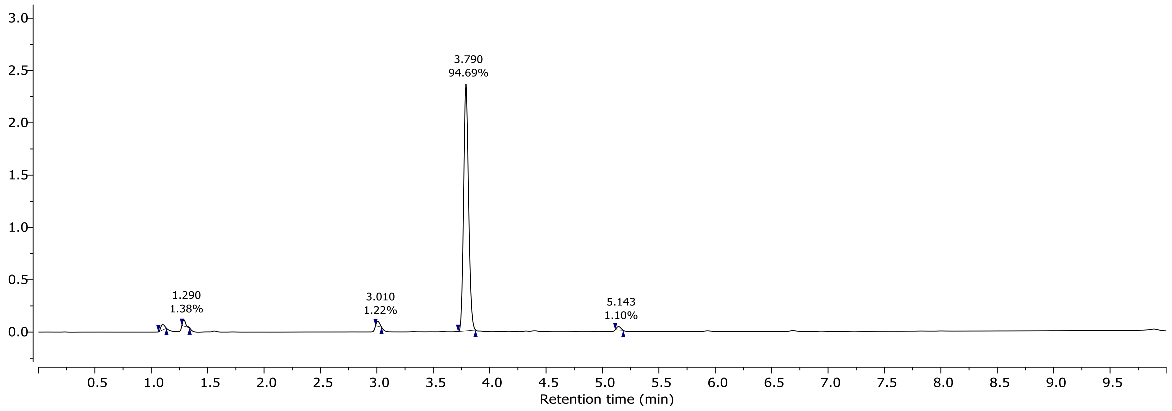

Figure S223. LCMS chromatogram of compound **65** at 254 nm.

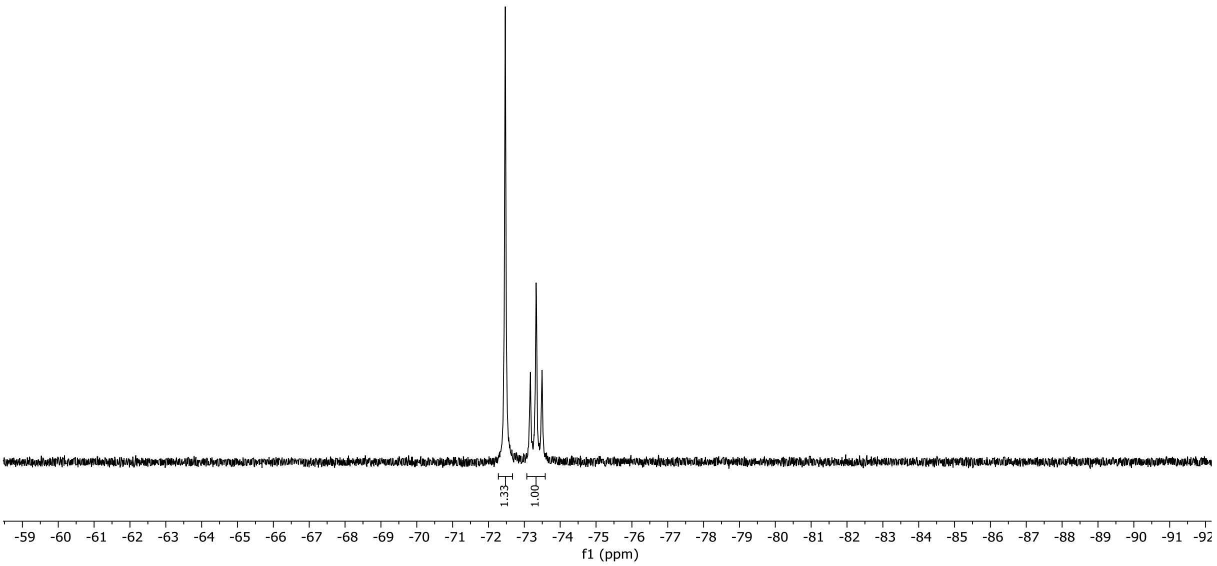

Figure S224. ^{19}F NMR of compound **65**.

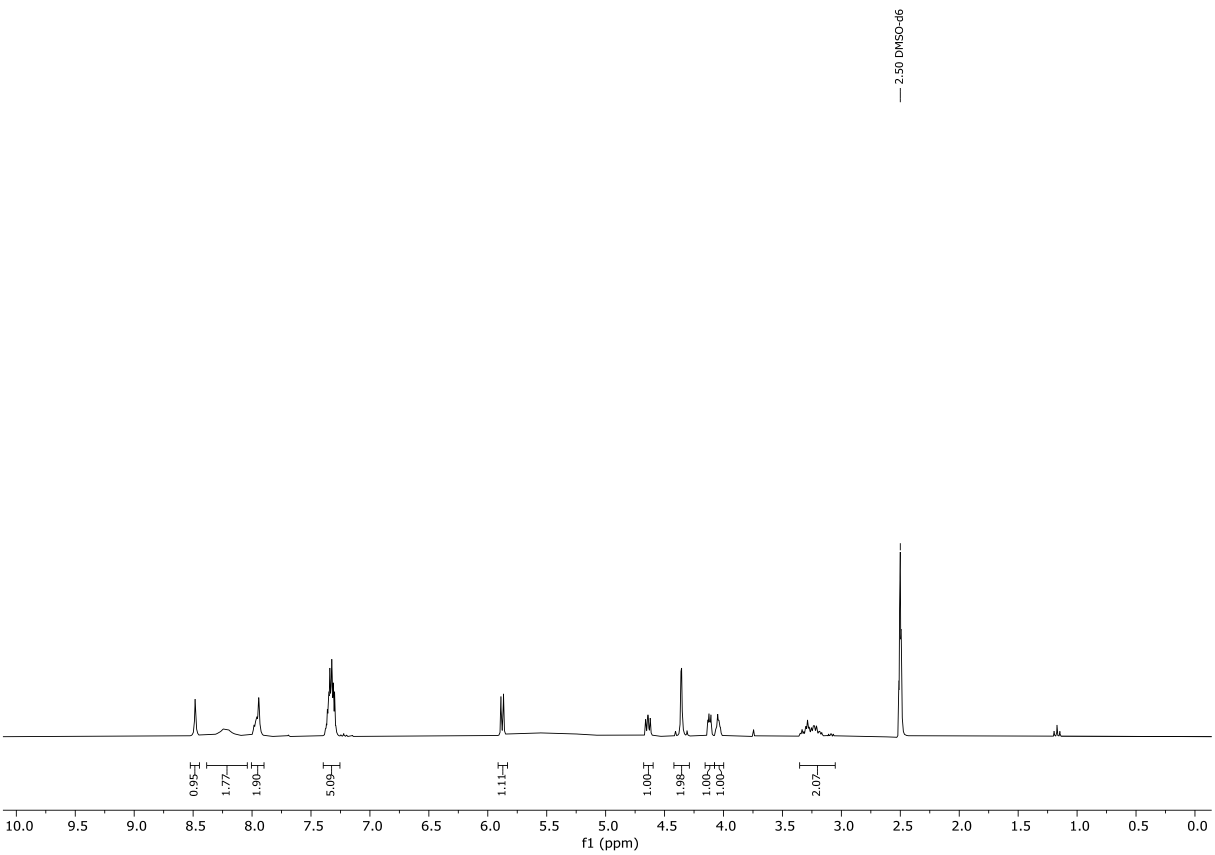

Figure S225. ^{1}H NMR of compound **66**.

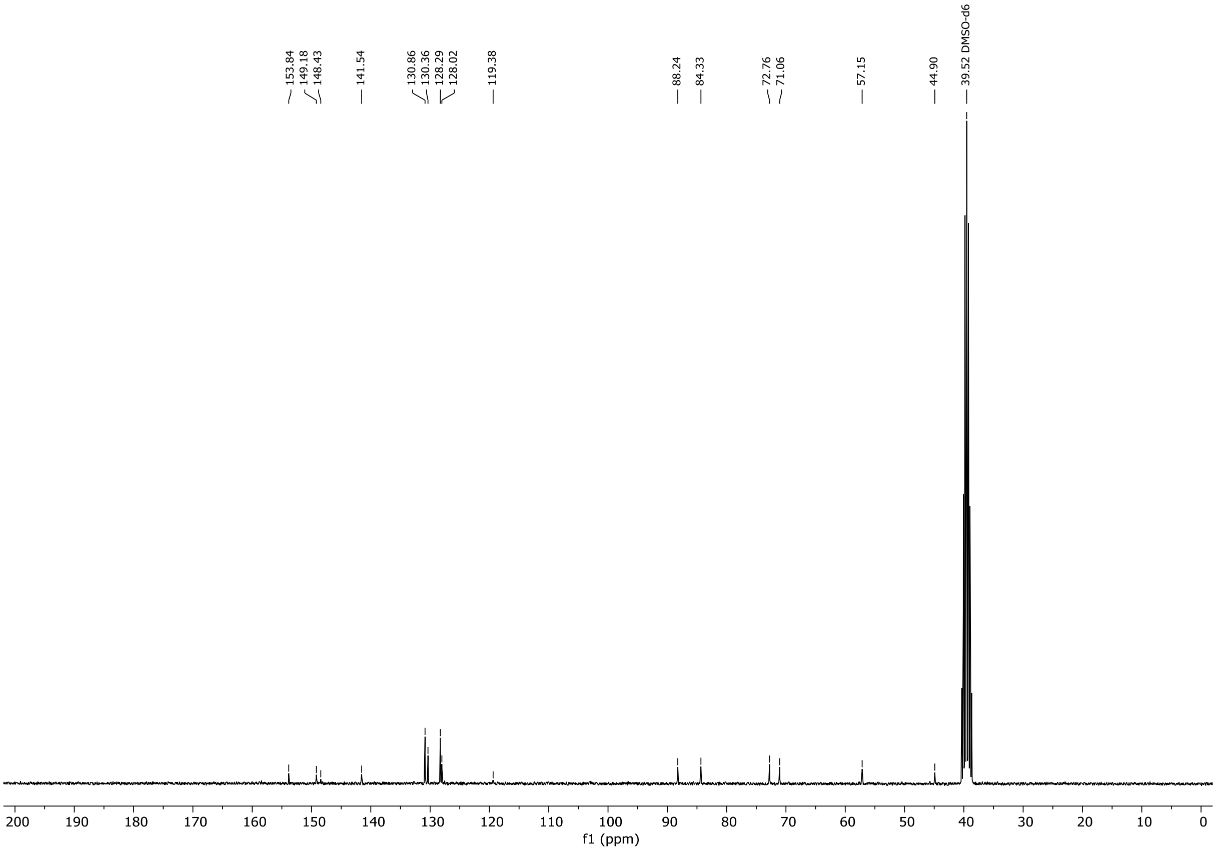

Figure S226. ^{13}C NMR of compound **66.**

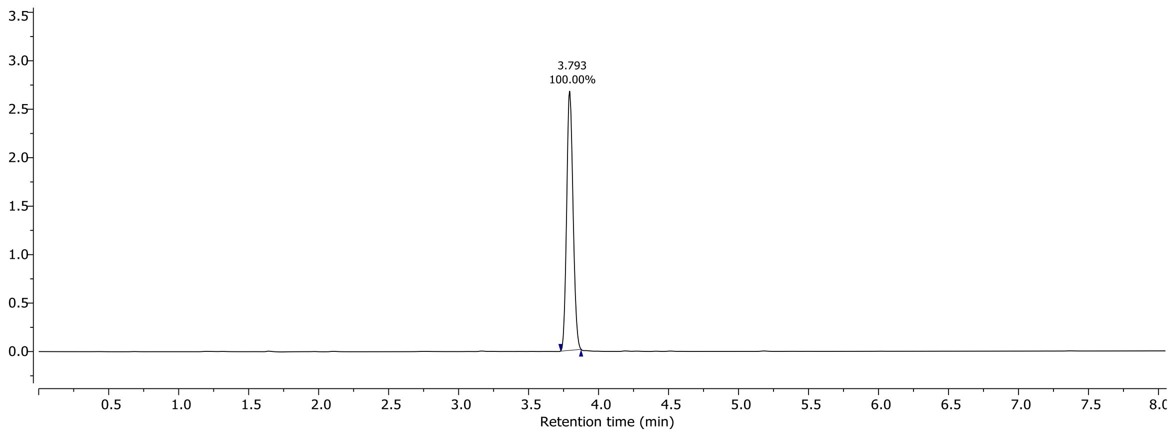

Figure S227. LCMS chromatogram of compound **66** at 254 nm.

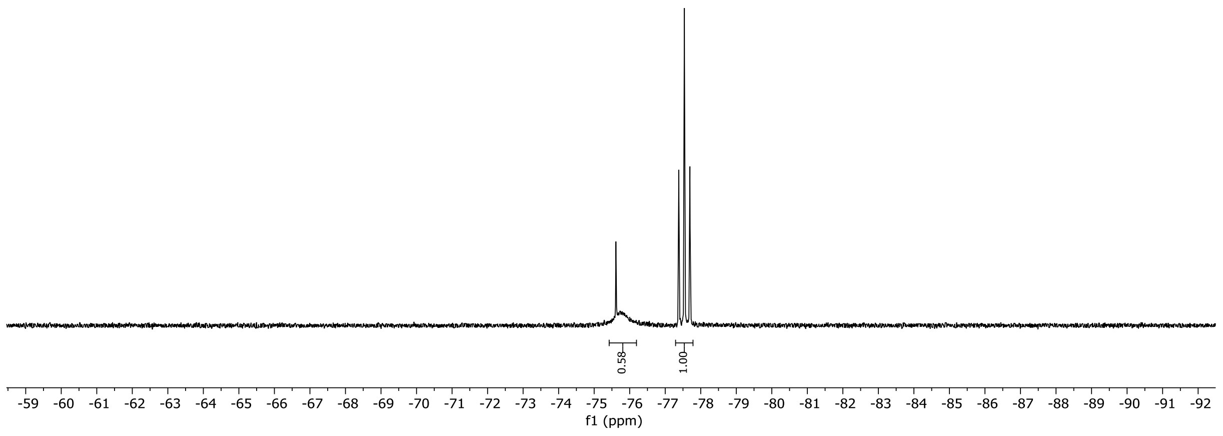

Figure S228. ^{19}F NMR of compound **66**.

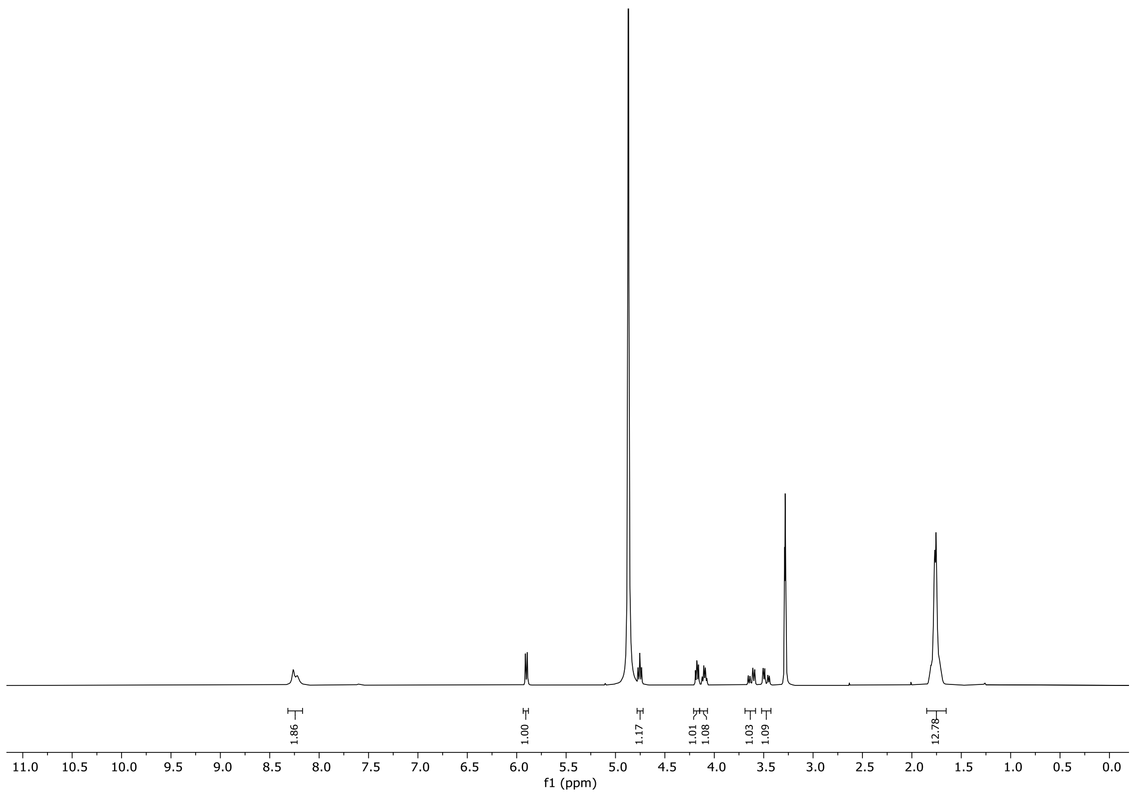

Figure S229. ^{1}H NMR of compound **67**.

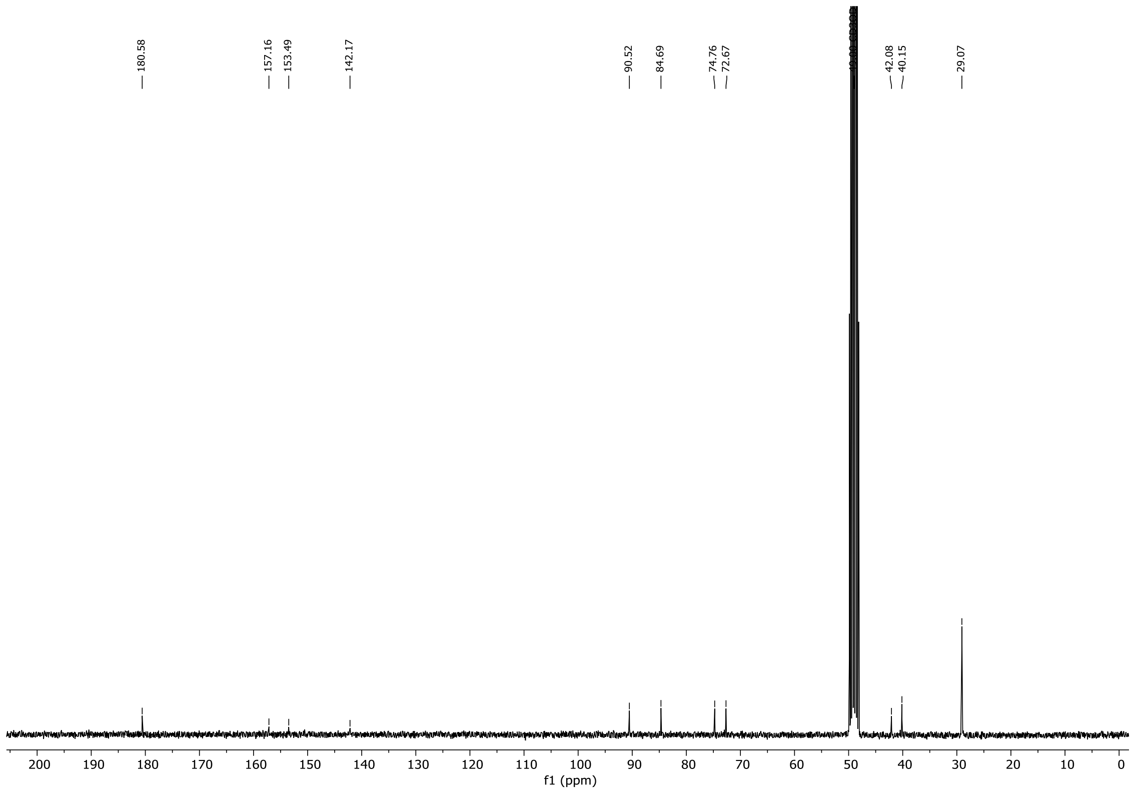

Figure S230. ^{13}C NMR of compound **67.**

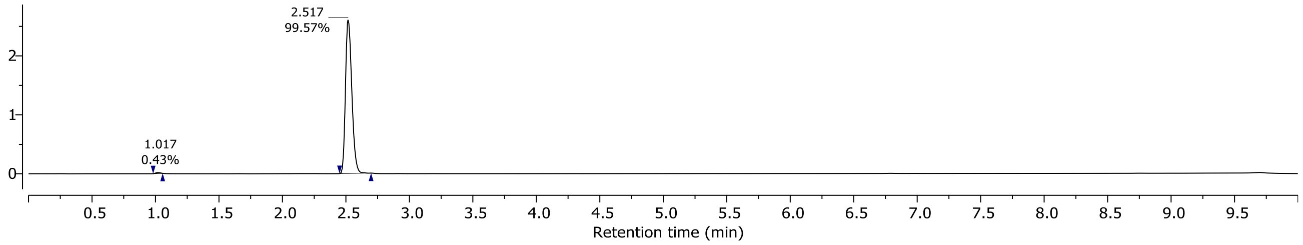

Figure S231. LCMS chromatogram of compound **67** at 254 nm.

15. References

1 S. A. Wildman and G. M. Crippen, *J Chem Inf Comput Sci*, 1999, **39**, 868–873.

2 P. Ertl, B. Rohde and P. Selzer, *J Med Chem*, 2000, **43**, 3714–3717.

3 P. R. Gerber, *J Comput Aided Mol Des*, 1998, **12**, 37–51.
